# Supplementary material for: Cap analogs with a hydrophobic photocleavable tag enable facile purification of fully capped mRNA with various cap structures
Source: Nat Commun. 2023 May 11;14:2657. doi: 10.1038/s41467-023-38244-8 (PMC10175277; doi:10.1038/s41467-023-38244-8)
Supplement: Supplementary file 1 — Supplementary Information [file 41467_2023_38244_MOESM1_ESM.pdf]

Supplementary Information File

Cap analogs with a hydrophobic photocleavable tag enable facile purification of fully capped mRNA with various cap structures

Masahito Inagaki[1+], Naoko Abe[1+], Zhenmin Li[1], Yuko Nakashima[1,2], Susit Acharyya[1], Kazuya Ogawa[1], Daisuke Kawaguchi[1], Haruka Hiraoka[1], Ayaka Banno[1], Zheyu Meng[1], Mizuki Tada[1], Tatsuma Ishida[1], Pingxue Lyu[1], Kengo Kokubo[1], Hirotaka Murase[1], Fumitaka Hashiya[2], Yasuaki Kimura[1], Satoshi Uchida[3,4,5], Hiroshi Abe[1,2,6,7*]

1 Department of Chemistry, Graduate School of Science, Nagoya University, Furo-cho, Chikusa-ku, Nagoya, Aichi 464-8602, Japan
2 Research Center for Materials Science, Nagoya University, Furo-cho, Chikusa-ku, Nagoya, Aichi 464-8602, Japan
3 Graduate School of Medical Science, Kyoto Prefectural University of Medicine, 1-5 Shimogamohangi-cho, Sakyo-ku, Kyoto 606-0823, Japan
4 Innovation Center of NanoMedicine (iCONM), Kawasaki Institute of Industrial Promotion, 3-25-14 Tonomachi, Kawasaki-ku, Kawasaki 210-0821, Japan
5 Medical Research Institute, Tokyo Medical and Dental University (TMDU), 1-5-45 Yushima, Bunkyo-ku Tokyo 113-8510, Japan
6 CREST, Japan Science and Technology Agency, 7, Gobancho, Chiyoda-ku, Tokyo 102-0076, Japan
7 Institute for Glyco-core Research (iGCORE), Nagoya University, Furo-cho, Chikusa-ku, Nagoya, Aichi 464-8601, Japan

*Corresponding author, h-abe@chem.nagoya-u.ac.jp
[+] *These authors contributed equally to this work*

Table of Contents

1. General Information and Instruments for Organic Synthesis
2. Experimental Details for Synthesis of Cap Analogs (with **Supplementary Figures 1 to 14**)
   - 2.1 Synthesis of Nitrobenzyl Alcohol Derivatives
   - 2.2 Synthesis of 2'-Nb-Di-PureCap Analog (**1**)
   - 2.3 Synthesis of 2'-Nb-Guanosine-5'-Diphosphate Imidazolide (**16**)
   - 2.4 Synthesis of Di-PureCap/2'OMe (**2**)
   - 2.5 Synthesis of Di-PureCap/3'OMe (**3**)
   - 2.6 Synthesis of Di-PureCap/N2 (**4**)
   - 2.7 Synthesis of Trinucleotide/Tetranucleotide PureCap Analogs (**5**–**8**)
   - 2.8 Synthesis of Trinucleotide/Tetranucleotide Cap Analogs with No Tag (**9**, **10**)
   - 2.9 Synthesis of More Hydrophobic 2'-Nb-Di-PureCap Analogs (**23**–**25**)
3. NMR Spectra of Synthesized Compounds
4. LC Profiles of Cap Analogs

Supplementary Table 1 MALDI TOF MS data of 5' fragments originated from RP-HPLC purified capped Nluc mRNAs after cleavage by DNAzyme 10-23.

Supplementary Figure 15. Removal of double-stranded RNA (dsRNA) impurity from transcribed Nluc mRNA (650-nt) using reversed-phase HPLC.

Supplementary Figure 16. IVT using dinucleotide PureCap cap analogs.

Supplementary Figure 17. RP-HPLC quantification of the capped/uncapped transcripts in the IVT reaction using PureCap analogs.

Supplementary Figure 18. MALDI-TOF MS analysis of 5' terminus of capped Nluc mRNAs prepared using tri/tetranucleotide cap analogs.

Supplementary Figure 19. Nluc mRNAs with different cap structures were compared in a HeLa S3-based cell-free translation system.

Supplementary Figure 20. Denaturing PAGE analysis of the IVT reaction with a PureCap analog **25.**

5. Supplementary References

1. General Information and Instruments for Organic Synthesis

Abbreviations: Standard abbreviations for the protecting groups are followed by the IUPAC-IUB Commission on Biochemical Nomenclature. All starting materials, reagents, and solvents of guaranteed grade were purchased from FUJIFILM Wako Chemicals, Tokyo Chemicals, Sigma-Aldrich, or Kanto Chemicals and used without further purification. All reactions involving moisture-sensitive reagents were performed under an argon atmosphere using oven-dried glassware. Column chromatography was performed on silica gel (63–210 mesh) purchased from Kanto Chemicals. All solid-phase nucleotide synthesis reagents were purchased from Chem Genes or Glen Research. All solvent compositions are reported in volume % unless specified otherwise. Syntheses of dinucleotides and trinucleotides were performed on a DNA/RNA synthesizer NR-2A_7MX or NRs-4A_10R7NP (Nihon Techno Service). NMR spectra were taken on JOEL NMR-ECS 400 (400 MHz for ^{1}H NMR, 101 MHz for ^{13}C NMR, and 163 MHz for ^{31}P NMR), JOEL NMR-ECS 600 (600 MHz for ^{1}H NMR, 151 MHz for ^{13}C NMR, and 243 MHz for ^{31}P NMR), or Magritek Spinsolve 60 Multi X ^{13}C-^{31}P (60 MHz for ^{1}H NMR, 15 MHz for ^{13}C NMR) instruments. The ^{1}H and ^{13}C{^{1}H} NMR chemical shifts (δ) are reported in parts per million (ppm) relative to residual solvents: $CHCl_3$ (7.26 ppm for ^{1}H NMR, 77.16 ppm for ^{13}C NMR), CD_3OD (3.31 ppm for ^{1}H NMR, 49.00 ppm for ^{13}C NMR), DMSO-d_6 (2.50 ppm for ^{1}H NMR, 39.52 ppm for ^{13}C NMR), and D_2O (4.79 ppm for ^{1}H NMR). ESI-TOF mass spectra were obtained on a micrOTOF-QII (Bruker Daltonics) instrument. MALDI-TOF mass spectra were obtained on an UltrafleXtreme (Bruker Daltonics) with 3-hydroxypicolinic acid as a matrix to detect the peaks of the synthesized RNAs.

2. Experimental Details for Synthesis of Cap Analogs (with Supplementary Figures 1 to 14)

2.1 Synthesis of Nitrobenzyl Alcohol Derivatives

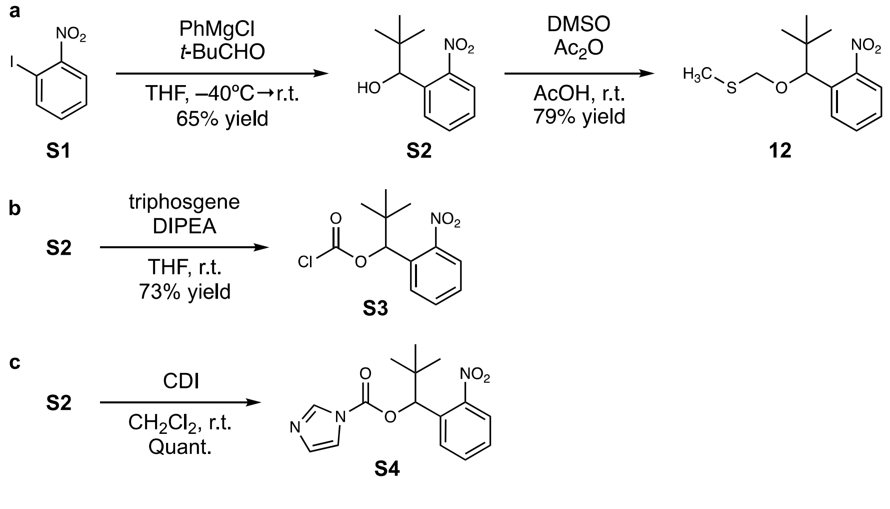

Supplementary Figure 1. Synthesis of Nitrobenzyl Alcohol Derivatives; (a) Synthesis of nitrobenzyl alcohol (**S2**) and its methylthioacetal derivative (**12**); (b) Synthesis of the chloroformate derivative (**S3**); Synthesis of the carbonylimidazole derivative (**S4**).

t**Bu-Nitrobenzyl Alcohol (S2)**: A mixture of 1-iodo-2-nitrobenzene (**S1**) (20 g, 80 mmol) in THF (150 mL) was cooled to -40 ºC. A solution of phenylmagnesium chloride in THF (2 M, 52 mL, 0.10 mol) was added to the mixture and stirred at -40 ºC for 1 hour. Trimethylacetaldehyde (12 mL, 9.6 g, 0.11 mol) was added to the reaction mixture and stirred at -40 ºC for 36 minutes. The reaction mixture was gradually warmed to room temperature. After being stirred for four hours, the reaction was quenched by adding an aqueous solution of *sat.* NH_4Cl. The mixture was extracted three times with ethyl acetate. The organic layer was dried over Na_2SO_4 and concentrated. The residue was purified by silica gel column chromatography eluted by hexane, followed by 17→25% ethyl acetate/hexane, to afford compound **S2** (11 g, 65% yield) as dark brown solid. All the spectral data of the product was consistent with the literature[1].

Methylthioacetal Derivative (12): To a solution of *t*Bu-nitrobenzyl alcohol (**S2**) (3.00 g, 14.3 mmol) in acetic acid (34.4 g, 33.0 mL, 572 mmol) was added DMSO (22.3 g, 20.0 mL, 286 mmol) and acetic anhydride (29.2 g, 27.0 mL, 286 mmol). After stirring at room temperature for 5 days, the reaction mixture was added dropwise to 10 M KOH *aq.* (130 mL, 1.29 mol) in an ice bath. The mixture was stirred at room temperature for 2 hours and then extracted with ethyl acetate. The organic layer was washed with brine and dried over Na_2SO_4. The residue was dissolved in hexane and subjected to silica gel column chromatography eluted by 0–6.3% ethyl acetate/hexane to afford the methylthioacetal **12** (3.1 g, 79% yield) as yellow oil. ^{1}H NMR (600 MHz, $CDCl_3$) δ 7.71 (dd, J = 8.2, 1.3 Hz, 1H), 7.64 (dd, J = 7.9, 1.5 Hz, 1H), 7.57–7.51 (m, 1H), 7.40–7.33 (m, 1H), 5.28 (s, 1H), 4.62 (d, J = 11.6 Hz, 1H), 4.27 (d, J = 11.6 Hz, 1H), 2.11 (s, 3H), 0.83 (s, 9H) ppm. ^{13}C NMR (151 MHz, $CDCl_3$) δ 151.20, 133.97, 131.94, 130.07, 128.35, 123.94, 79.15, 74.16, 36.41, 26.00, 14.58 ppm. ESI-TOF-MS calcd. for $C_{13}H_{19}NNaO_3S$, 292.0978 $[M + Na]^+$; found, 292.0981.

Chloroformate Derivative (S3): To a solution of *t*Bu-nitrobenzyl alcohol (**S2**) (639 mg, 3.05 mmol) in THF (10.2 mL), *N*,*N*-diisopropylethylamine (986 mg, 1.30 mL, 7.63 mmol) and triphosgene (997 mg, 3.36 mmol) were added successively. After being stirred for 23 hours at room temperature, triphosgene (200 mg) and DIPEA (1.00 mL) were added to the mixture and stirred at room temperature for 24 hours. The reaction mixture was diluted with dichloromethane and washed with water. The organic layer was dried over Na_2SO_4 and

concentrated by a rotary evaporator to afford the chloroformate as a dark brown oil (1.64 g, crude). The product was purified by silica gel column chromatography (hexane: AcOEt = 1.0 then 6:1, as eluent) to afford the chloroformate **S3** (601 mg, 72.6% yield) as yellow oil. ^{1}H NMR (600 MHz, $CDCl_3$) δ 7.90 (ddd, J = 8.2, 3.3, 1.4 Hz, 1H), 7.70–7.54 (m, 2H), 7.46 (dddd, J = 9.8, 7.1, 2.6, 1.5 Hz, 1H), 6.56 (d, J = 1.5 Hz, 1H), 0.94 (d, J = 2.5 Hz, 9H) ppm. ^{13}C NMR (151 MHz, $CDCl_3$) δ 149.97, 149.95, 149.04, 132.99, 131.13, 129.52, 128.80, 124.93, 84.30, 36.60, 25.56 ppm. ESI-TOF-MS calcd. for $C_{12}H_{14}ClNNaO_4$, 294.0504 $[M + Na]^+$; found, 294.0486.

Carbonylimidazole Derivative (S4): To a solution of *t*Bu-nitrobenzyl alcohol (**S2**) (5.8 g, 28 mmol) in dichloromethane (60 mL) was added 1,1'-carbonyldiimidazole (6.8 g, 42 mmol). After stirring at room temperature for five hours, the reaction mixture was diluted with dichloromethane and washed with brine. The organic layer was dried over Na_2SO_4 and concentrated. The residue was purified by silica gel column chromatography eluted by 50→67% ethyl acetate/hexane to afford compound **S3** (8.5 g, quant.) as brown oil. ^{1}H NMR (400 MHz, $CDCl_3$) δ 8.11 (t, J = 1.1 Hz, 1H), 7.87 (dd, J = 8.2, 1.3 Hz, 1H), 7.59–7.48 (m, 2H), 7.46–7.35 (m, 2H), 7.05–6.98 (m, 1H), 6.62 (s, 1H), 0.96 (s, 9H) ppm. ^{13}C NMR (101 MHz, $CDCl_3$) δ 149.22, 147.85, 136.97, 132.83, 131.71, 131.01, 129.35, 128.58, 124.97, 117.09, 80.39, 36.51, 25.84. ESI-TOF-MS calcd. for $C_{15}H_{17}N_3NaO_4$, 326.1111 $[M + Na]^+$; found, 326.1076.

2.2 Synthesis of 2'-Nb-Di-PureCap Analog (1)

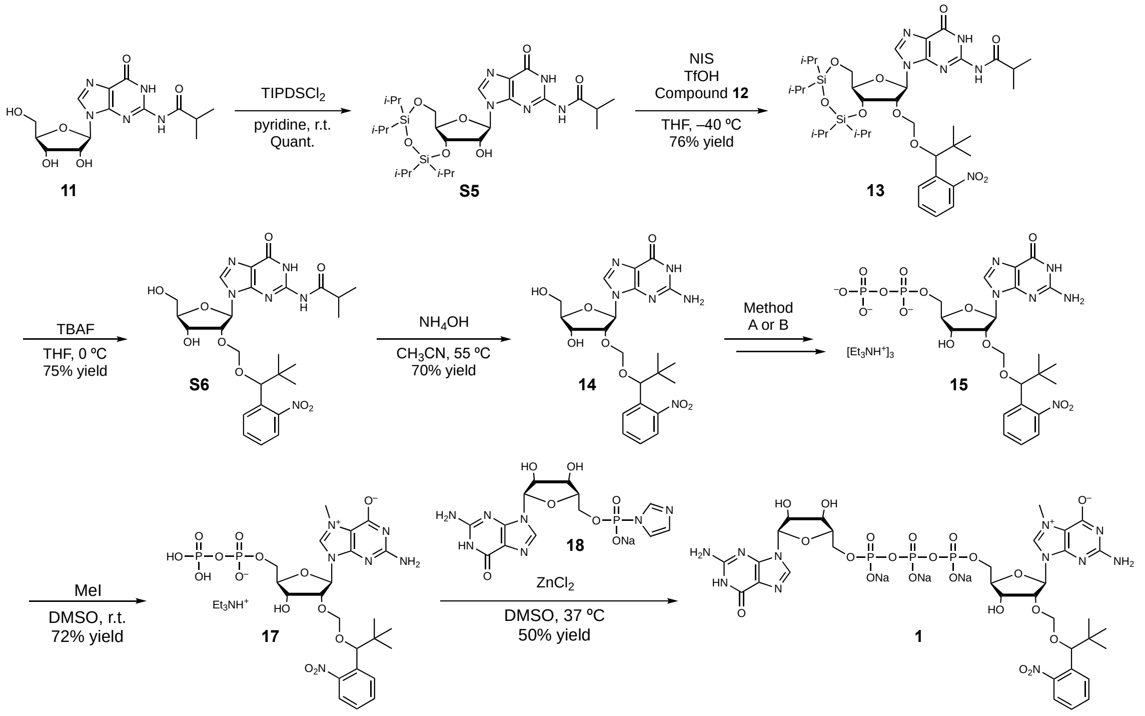

Supplementary Figure 2. Synthesis of 2'-Nb-Di-PureCap Analog (**1**).

***N*²-Isobutyryl-3',5'-*O*-TIPDS-Guanosine (S5):** *N*²-Isobutyryl-guanosine (**11**) (15.0 g, 42.5 mmol) was azeotroped with anhydrous pyridine (85.0 mL × 2). The residue was dissolved in anhydrous pyridine (85.0 mL) and cooled in an ice bath. To the mixture was added 1,3-dichloro-1,1,3,3-tetraisopropyldisiloxane (14.8 g, 14.6 mL, 46.8 mmol). The mixture was warmed to room temperature and stirred at room temperature for 5 hours. The reaction mixture was poured into stirred ice-cold water. The resulting precipitate was collected by filtration and washed (100 mL × 3) with water, followed by hexane (100 mL × 3). The obtained solid was transferred to the eggplant flask and azeotroped with benzene (100 mL× 3) to give compound **S5** as white solid (22.2 g, 88.0% yield). The product was used for the next reaction without further purification. The physical data matched the previously published data[2].

***N*²-Isobutyryl-3',5'-*O*-TIPDS-2'-Nb-guanosine (13):** To a suspension of *N*²-isobutyryl-3',5'-*O*-TIPDS-guanosine (**S5**) (2.96 g, 4.97 mmol), methylthioacetal derivative (**12**) (1.47 g, 5.47 mmol), and powdery molecular sieves 3Å (2.00 g), in tetrahydrofuran (50.0 mL), was added *N*-iodosuccinimide (1.23 g, 5.47 mmol) and then cooled to -40 ºC. To the cooled suspension was added trifluoromethanesulfonic acid (1.12 g, 662 μL, 7.46 mmol) and stirred at -40 ºC for 19 hours. The reaction mixture was quenched by adding triethylamine (24.0 mL) and then diluted with ethyl acetate. The mixture was washed with saturated *aq.* $NaHCO_3$ (×2), saturated *aq.* sodium thiosulfate (×1), and brine (×1), successively. The organic layer was

dried over Na_2SO_4 and concentrated. The residue was purified by silica gel column chromatography eluted by 0–3.2% methanol/dichloromethane to afford compound **13** (3.12 g, 76% yield) as yellow foam. ^{1}H NMR (600 MHz, $CDCl_3$) δ 12.08 (s, 1H), 9.04 (s, 1H), 7.94 (s, 1H), 7.74 (d, *J* = 8.4 Hz, 1H), 7.61–7.52 (m, 2H), 7.40 (dd, *J* = 8.3, 4.3 Hz, 1H), 5.92 (s, 1H), 5.19 (d, *J* = 6.7 Hz, 1H), 4.61 (d, *J* = 6.7 Hz, 1H), 4.52 (dd, *J* = 9.4, 4.4 Hz, 1H), 4.28 (d, *J* = 4.4 Hz, 1H), 4.16 (d, *J* = 13.4 Hz, 1.5H), 4.02 (dd, *J* = 9.5, 2.3 Hz, 1.5H), 3.93 (dd, *J* = 13.4, 2.5 Hz, 2H), 2.73 (p, *J* = 6.9 Hz, 1H), 1.23 (d, *J* = 6.8 Hz, 3H), 1.18 (d, *J* = 6.9 Hz, 3H), 1.12–0.91 (m, 78H), 0.91–0.82 (m, 7H), 0.65 (s, 9H) ppm. ^{13}C NMR (151 MHz, $CDCl_3$) δ 179.11, 155.65, 150.82, 147.80, 147.47, 136.43, 133.14, 132.35, 130.10, 128.64, 124.11, 121.15, 91.47, 88.01, 80.98, 77.42, 77.21, 76.99, 76.25, 70.30, 59.48, 50.81, 36.54, 36.45, 25.78, 19.13, 18.92, 17.51, 17.44, 17.39, 17.35, 17.31, 17.27, 17.24, 17.17, 17.13, 17.02, 16.89, 13.47, 13.25, 13.10, 12.94, 12.70, 12.57 ppm. ESI-TOF-MS calcd. for $C_{38}H_{61}N_6O_{10}Si_2$, 817.3982 $[M + H]^+$; found, 817.3921.

***N*^{2}-Isobutyryl-2'-Nb-guanosine (S6):** A solution of the protected guanosine **13** (850 mg, 1.04 mmol) in tetrahydrofuran (4.80 mL) was cooled in an ice bath, and 1 M TBAF/THF (10.0 mL, 10.4 mmol) was added to the solution. After stirring for 5 hours at 0 ºC, the reaction mixture was diluted with dichloromethane and washed with water. The organic layer was dried over Na_2SO_4 and concentrated. The residue was purified by silica gel column chromatography eluted by 0–9.1% methanol/dichloromethane to afford compound **S6** (450 mg, 75% yield) as colorless amorphous solid. ^{1}H NMR (600 MHz, $CDCl_3$, a mixture of stereoisomers) δ 12.40 (s, 0.5H), 12.36 (s, 0.5H), 10.27 (m, 0.5H), 10.21 (m, 0.5H), 8.21 (s, 0.5H), 8.00 (s, 0.5H), 7.66 (d, *J* = 8.1 Hz, 0.5H), 7.61 (d, *J* = 7.9 Hz, 0.5H), 7.53–7.45 (m, 2H), 7.35 (q, *J* = 8.3 Hz, 1.5H), 7.25 (t, *J* = 7.7 Hz, 0.5H), 5.98 (d, *J* = 4.7 Hz, 0.5H), 5.80 (d, *J* = 4.6 Hz, 0.5H), 5.10 (s, 0.5H), 4.99 (s, 0.5H), 4.84 – 4.71 (m, 2.5H), 4.67 (t, *J* = 4.8 Hz, 1H), 4.58 (t, *J* = 10.7 Hz, 1.5H), 4.13 (dd, *J* = 10.4, 3.8 Hz, 1H), 3.91–3.84 (m, 1H), 3.76 (t, *J* = 13.2 Hz, 1H), 3.39 (t, *J* = 4.1 Hz, 1H), 2.90 (h, *J* = 6.8 Hz, 1H), 1.24 (td, *J* = 4.3, 2.0 Hz, 6H), 0.71 (s, 4.5H), 0.59 (s, 4.5H) ppm. ^{13}C NMR (151 MHz, $CDCl_3$, a mixture of stereoisomers) δ 180.28, 180.22, 155.67, 155.60, 150.52, 149.79, 148.38, 148.27, 148.16, 147.86, 139.26, 133.87, 133.51, 132.14, 131.81, 130.01, 129.92, 128.51, 128.32, 123.95, 123.77, 121.00, 120.94, 95.02, 93.66, 88.27, 86.19, 86.05, 80.47, 79.49, 79.41, 79.23, 77.38, 77.17, 76.96, 70.68, 70.50, 70.25, 62.00, 36.66, 36.21, 36.16, 26.57, 25.65, 25.60, 19.14, 19.06, 19.03 ppm. ESI-TOF-MS calcd. for $C_{26}H_{33}N_6O_9$, 573.2315 $[M - H]^-$; found 573.2333.

2'-Nb-guanosine (14): To a solution of the compound **S6** (1.08 g, 1.88 mmol) in acetonitrile (14.4 mL) was added 28% ammonium hydroxide solution (36.0 mL). The mixture was heated

to 55 °C. After stirring at 55 °C for 6 hours, the reaction mixture was concentrated and azeotroped with benzene. The residue was suspended in a 1:3 mixture of dichloromethane and diethyl ether. The resulting precipitate was collected by filtration and rinsed with a 1:3 mixture of dichloromethane and diethyl ether. The obtained solid was dried in a desiccator over P_2O_5 under a vacuum to afford 2'-Nb-guanosine (**14**) (666 mg, 70% yield) as a white foam. ^{1}H NMR (600 MHz, DMSO-d_6, a mixture of stereoisomers) δ 10.61, 10.58 (2s, 1H), 7.88 (s, 0.5H), 7.79 (dd, *J* = 8.0, 1.3 Hz, 0.5H), 7.70–7.62 (m, 1.5H), 7.53–7.46 (m, 1H), 7.41 (m, 1H), 7.31 (ddd, *J* = 8.6, 6.6, 2.1 Hz, 0.5H), 6.37 (d, *J* = 10.1 Hz, 2H), 5.81 (d, *J* = 6.0 Hz, 0.5H), 5.59 (d, *J* = 5.6 Hz, 0.5H), 5.21 (d, *J* = 5.4 Hz, 0.5H), 5.15 (d, *J* = 5.3 Hz, 0.5H), 5.10 (t, *J* = 5.6 Hz, 0.5H), 5.03 (t, *J* = 5.5 Hz, 0.5H), 4.95 (s, 0.5H), 4.82 (s, 0.5H), 4.71–4.67 (m, 1H), 4.67–4.61 (m, 1H), 4.47 (t, *J* = 5.2 Hz, 0.5H), 4.38 (d, *J* = 7.2 Hz, 0.5H), 4.23 (td, *J* = 5.1, 3.8 Hz, 1H), 3.87 (q, *J* = 3.6 Hz, 0.5H), 3.82 (q, *J* = 3.7 Hz, 0.5H), 3.63–3.52 (m, 1H), 3.52–3.42 (m, 1H), 0.68 (s, 4.5H), 0.53 (s, 4.5H) ppm. ^{13}C NMR (151 MHz, DMSO-d_6, a mixture of stereoisomers) δ 157.25, 154.15, 154.11, 151.61, 151.48, 150.98, 150.00, 136.35, 135.70, 133.46, 133.00, 132.82, 132.39, 130.10, 129.99, 129.45, 128.93, 124.35, 124.11, 117.38, 117.18, 94.57, 92.51, 86.37, 86.04, 85.85, 85.39, 80.21, 78.95, 78.84, 78.72, 70.25, 70.06, 69.75, 62.05, 61.81, 40.46, 40.32, 40.19, 40.04, 39.91, 39.76, 39.62, 36.64, 36.04, 25.88, 25.83 ppm. ESI-TOF-MS calcd. for $C_{22}H_{28}N_6NaO_8$, 527.1861 [M + Na]$^+$; found 527.1758.

Synthesis of Diphosphate 15 (Method A)

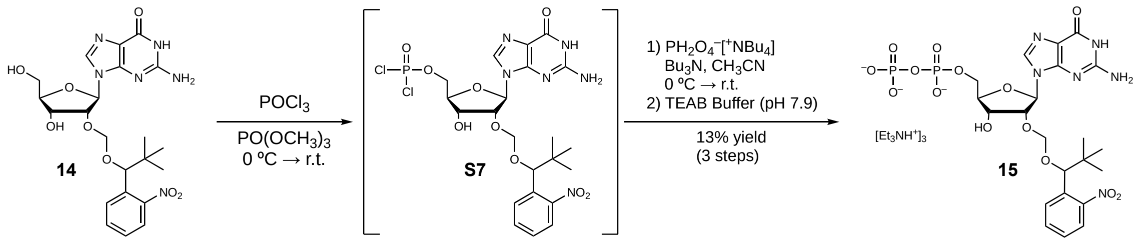

Supplementary Figure 3. Synthesis of Diphosphate 15 (Method A).

To a solution of 2'-Nb-guanosine (**14**) (200 mg, 0.396 mmol) in trimethyl phosphate (3.70 mL) was added phosphoryl chloride (48.0 µL, 79.0 mg, 0.515 mmol) in an ice bath. The mixture was gradually warmed to room temperature and stirred at room temperature. After being stirred at room temperature for 2 hours, the reaction mixture containing the phosphorodichloridate intermediate (**S7**) was added to a mixture of 0.44 M tetrabutylammonium dihydrogen phosphate/CH_3CN (1.80 mL, 0.792 mmol) and tributylamine (846 µL, 660 mg, 3.56 mmol) in an ice bath. The reaction mixture was warmed to room

temperature and stirred at room temperature for 19 hours. Then, the reaction mixture was quenched by adding 0.2 M TEAB buffer (pH 7.9, 5.0 mL). After being stirred at room temperature for 1 hour, the mixture was diluted with water and washed 5 times with dichloromethane. The aqueous layer was concentrated by a rotary evaporator at 50 ºC. The residue was purified by ion-exchange chromatography on QAE-Sephadex with a linear 0–1.5 M gradient of TEAB buffer containing 0–10% CH_3CN. 2'-Nb-Guanosine-5'-diphosphate (**15**) was obtained as triethylammonium salt (51.0 mg, 13% yield) as white solid.

Synthesis of Diphosphate 15 (Method B)

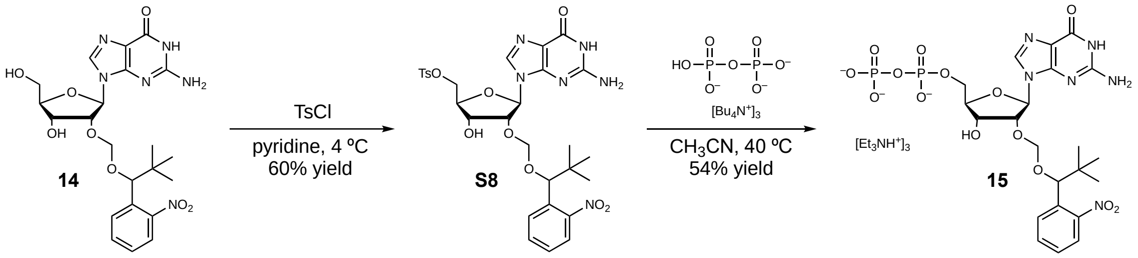

Supplementary Figure 4. Synthesis of Diphosphate 15 (Method B).

2'-Nb- 5'-*O*-Tosyl-guanosine (S8): To a solution of 2'-modified guanosine (**1**) (294 mg, 0.583 mmol) in pyridine (6.00 mL) was added dropwise *p*-toluenesulfonyl chloride (214 mg, 1.12 mmol) in pyridine (1.40 mL) in an ice bath. The mixture was stirred at 4 ºC for 3–4 days. The reaction mixture was quenched by adding ice-cold water and extracted 3 times with dichloromethane. The organic layer was dried over Na_2SO_4 and evaporated. The residue was subjected to silica gel column chromatography eluted by 2.4–9.1% methanol/dichloromethane to afford compound **S8** (260 mg, 68% yield) as yellow foam. ^{1}H NMR (600 MHz, $CDCl_3$, a mixture of stereoisomers) δ 11.92 (br, s), 7.76–7.63 (m, 4H), 7.57 (d, J = 11.9 Hz, 1.5H), 7.46–7.35 (m, 2H), 7.27–7.23 (m, 2.5H), 6.03–5.76 (m, 1H), 5.68 (s, 1H), 5.26–5.02 (m, 2H), 4.90 (d, J = 16.1 Hz, 2H), 4.76 (s, 2H), 4.47–4.30 (m, 1.5H), 4.21 (s, 2.5H), 2.37 (s, 3H), 0.77 (s, 4.5H), 0.74 (s, 4.5H) ppm. ^{13}C NMR (151 MHz, $CDCl_3$, a mixture of stereoisomers) δ 158.75, 154.18, 151.28, 151.01, 150.38, 145.30, 136.63, 133.76, 132.27, 131.92, 130.36, 130.10, 130.06, 129.95, 128.51, 128.02, 123.87, 117.14, 94.78, 81.80, 79.86, 78.56, 77.91, 70.70, 70.13, 69.94, 53.56, 36.68, 36.50, 29.78, 25.73, 25.67, 25.56, 21.72 ppm. ESI-TOF-MS calcd. for $C_{29}H_{34}N_6NaO_{10}S$, 681.1949 [M + Na]$^+$; found 681.1948.

2'-Nb-guanosine-5'-diphosphate (15): To a suspension of 2'-Nb-5'-*O*-tosyl-guanosine (812 mg, 1.23 mmol) and powdered 3A molecular sieves (1.00 mg) in CH_3CN (1.27 mL), was added 725 mM tris-tetrabutylammonium pyrophosphate in CH_3CN (2.55 mL, 1.85 mmol). After stirring for 2 days at 40 ºC, the reaction mixture was diluted with water. The resulting

suspension was centrifuged for 30 minutes at 4,500 rpm. The supernatant was collected, and the precipitate was resuspended in water. The resulting suspension was centrifuged for 30 minutes at 4,500 rpm. The supernatant was collected and purified by ion-exchange chromatography on QAE-Sephadex (φ = 4.5 cm, *h* = 8.4 cm, 140 cm^3, 12 mL/min) with a linear 0–1.5 M gradient of TEAB buffer (pH 7.9) containing 0–10% CH_3CN (270 minutes). The fractions containing the product were collected and concentrated to afford 2'-Nb guanosine diphosphate (**15**) (614 mg, 650 μmol, 52.8% yield). ^{1}H NMR (600 MHz, D_2O, a mixture of stereoisomers) *δ* 7.87 (s, 0.5H), 7.58 (s, 0.5H), 7.52 (d, *J* = 8.1 Hz, 0.5H), 7.47–7.38 (m, 1H), 7.38–7.29 (m, 1H), 7.28–7.17 (m, 1H), 7.01 (t, *J* = 7.8 Hz, 0.5H), 5.73 (d, *J* = 5.8 Hz, 0.5H), 5.48 (d, *J* = 5.0 Hz, 0.5H), 4.96, 4.94 (2s, 1H), 4.82 (d, *J* = 7.5 Hz, 0.5H), 4.76 (s, 0.5H), 4.71 (d, *J* = 7.3 Hz, 0.5H), 4.62 (dd, *J* = 18.0, 6.6 Hz, 1.5H), 4.52 (t, *J* = 5.1 Hz, 0-.5H), 4.45 (t, *J* = 5.0 Hz, 1H), 4.14 (d, *J* = 4.1 Hz, 0.5H), 4.10 – 4.03 (m, 1.5H), 3.99 (d, *J* = 4.8 Hz, 1H), 3.02 (q, *J* = 7.3 Hz, 15H), 1.10 (t, *J* = 7.3 Hz, 24H), 0.52 (s, 4.5H), 0.41 (s, 4.5H) ppm. ^{13}C NMR (151 MHz, D_2O, a mixture of stereoisomers) *δ* 158.95, 158.75, 153.89, 153.59, 151.59, 151.01, 149.74, 148.09, 138.02, 137.37, 133.49, 132.57, 132.47, 132.21, 129.86, 129.66, 128.65, 128.06, 123.80, 123.30, 116.33, 115.96, 95.44, 93.71, 86.20, 84.26, 84.20, 83.73, 83.67, 81.61, 80.72, 78.55, 76.97, 69.07, 68.94, 64.84, 64.81, 64.46, 46.63, 36.55, 35.65, 24.78, 24.68, 8.24 ppm. ^{31}P NMR (243 MHz, D_2O, a mixture of stereoisomers) *δ* -8.54 (1P), -10.61 (1P) ppm. ESI-TOF-MS calcd. for $C_{22}H_{29}N_6O_{14}P_2$, 663.1223 $[M - H]^-$; found 663.1378.

***N*^7-Methyl-2'-Nb-guanosine-5'-diphosphate (17):** To a solution of 2'-Nb-guanosine-5'-diphosphate (**15**) (0.564 mmol) in DMSO (4.70 mL) was added iodomethane (321 mg, 141 μL, 2.26 mmol). After stirring at room temperature for 13 hours, the reaction mixture was diluted with water and washed 4 times with dichloromethane. The crude was purified by ion-exchange chromatography on DEAE-Sephadex™ A-25 (φ = 4.5 cm, *h* = 8.4 cm, 140 cm^3, 12 mL/min) with a linear 0–0.8 M gradient of TEAA buffer (pH 6.0) containing 0–8% CH_3CN (270 minutes). After the removal of CH_3CN under reduced pressure, the product was desalted through Wakosil®25C18 (particle size, 15–30 μm (spherical); column size, φ = 4.80 cm, *h* = 10.2 cm, 184 cm^3 (12 mL/min); Solvent A, H_2O (20 minutes), then Solvent B, 90% CH_3CN/H_2O (20 minutes)), and the fractions containing product were concentrated to afford compound **17** (316 mg, 405 μmol, 71.8%). ^{1}H NMR (600 MHz, D_2O, 1:1 mixture of two stereoisomers) *δ* 9.12 (s, 0.5H), 8.93 (s, 0.5H), 7.61 (d, *J* = 8.1 Hz, 0.5H), 7.56–7.48 (m, 1H), 7.48–7.41 (m, 1.5H), 7.33 (t, *J* = 7.1 Hz, 0.5H), 7.29–7.23 (m, 0.5H), 5.97 (d, *J* = 4.6 Hz, 0.5H), 5.80 (d, *J* = 5.0 Hz, 0.5H), 4.97 (s, 0.5H), 4.92–4.87 (m, 1H), 4.80 (dd, *J* = 15.3, 7.1 Hz, 1H), 4.71–4.69 (m, 0.5H), 4.51 (q, *J* = 5.4 Hz, 1H), 4.39 (t, *J* = 5.0 Hz, 0.5H), 4.31 (t, *J* =

4.5 Hz, 0.5H), 4.22 (s, 1H), 4.17–4.08 (m, 1H), 4.05 (s, 1H), 3.98 (s, 1.5H), 3.93 (s, 1.5H), 3.04 (q, J = 7.4 Hz, 6H), 1.12 (t, J = 7.3 Hz, 8H), 0.54 (s, 4.5H), 0.51 (s, 4.5H) ppm. ^{31}P NMR (243 MHz, D_2O, 1:1 mixture of two stereoisomers) δ -9.94 (1P), -10.66 (1P) ppm. ^{1}H NMR (600 MHz, D_2O, single stereoisomer isolated by HPLC) δ 8.93 (s, 1H), 7.60 (d, J = 8.2 Hz, 1H), 7.49 (dd, J = 15.2, 8.1 Hz, 2H), 7.29 (t, J = 7.6 Hz, 1H), 5.82 (s, 1H), 5.00 (d, J = 4.1 Hz, 1H), 4.93 (d, J = 8.0 Hz, 1H), 4.86 (d, J = 7.2 Hz, 1H), 4.54 – 4.50 (m, 1H), 4.42 (t, J = 5.2 Hz, 1H), 4.25 (s, 1H), 4.10 (d, J = 11.2 Hz, 1H), 3.94 (d, J = 3.7 Hz, 3H), 3.05 (t, J = 7.3 Hz, 4H), 1.13 (t, J = 7.1 Hz, 6H), 0.61 (s, 10H) ppm. ^{13}C NMR (151 MHz, D_2O, single stereoisomer isolated by HPLC) δ 155.41, 154.99, 149.37, 148.61, 136.68, 133.90, 132.44, 130.24, 128.15, 123.51, 107.91, 95.16, 87.76, 85.95, 82.16, 79.32, 68.89, 64.78, 46.70, 36.52, 36.07, 24.76, 8.25 ppm. ^{31}P NMR (243 MHz, D_2O, single stereoisomer isolated by HPLC) δ -10.03 (1P), -10.71 (1P) ppm. ESI-TOF-MS calcd. for $C_{23}H_{31}N_6O_{14}P_2$, 677.1379 [M - H]$^-$; found 677.1374.

Guanosine 5'-monophosphate imidazolide monosodium salt (18): To a solution of guanosine 5'-monophosphate triethylammonium (300 mg, 0.53 mmol), imidazole (290 mg, 4.2 mmol), and 2,2'-dithiodipyridine (350 mg, 1.6 mmol) in *N*,*N*-dimethylformamide (7.5 mL), was added triethylamine (150 µL, 110 mg, 1.1 mmol) followed by triphenylphosphine (420 mg, 1.6 mmol). After stirring at room temperature for 2 hours, the reaction mixture was added dropwise to a sodium perchlorate solution (65 mg, 0.53 mmol) in acetone (60 mL) under stirring. The resulting suspension was transferred to a centrifuge tube and centrifuged for 10 minutes at 4,500 rpm. The supernatant was discarded, and the precipitate was rinsed 5 times with acetone. The solid was dried in a desiccator over P_2O_5 under reduced pressure to afford guanosine 5'-monophosphate imidazolide monosodium salt (**18**) (230 mg, quant.) as white solid. ^{31}P NMR (243 MHz, DMSO-d_6) δ 1.80 ppm.

2'-Nb-Di-PureCap Analog (1): N^7-Methyl-2'-Nb-guanosine-5'-diphosphate triethylammonium salt (**17**) (63.5 mg, 81.4 µmol) and guanosine-5'-monophosphate imidazolide (**18**) (106 mg, 244 µmol) were dissolved in 0.50 M zinc chloride/DMSO solution (2.66 mL, $ZnCl_2$: 1.33 mmol). The mixture was incubated for 34 hours at 37 ºC. The reaction mixture was quenched by adding a mixture of 100 mM *aq.* EDTA (16.0 mL, 1.60 mmol) and 0.2 M TEAB buffer (pH 7.9, 10.0 mL, adjusted the pH 5–6). The mixture was diluted with water to 100 mL of the final volume. The mixture was purified by ion-exchange chromatography on DEAE-Sephadex™ A-25 (φ = 4.5 cm, h = 8.4 cm, 140 cm^3, 12 mL/min) with a linear 0–1.0 M gradient of TEAA buffer (pH 6.0) containing 0–10% CH_3CN. After the removal of CH_3CN under reduced pressure, the purity of the product was further purified by reversed-phase MPLC (instrument, ISCO; column, Wakosil@25C18 (φ = 4.80 cm, h = 10.2

cm (184 cm^3)); Solvent A, 50 mM TEAA buffer (pH 6.0) containing 0.5% CH_3CN; Solvent B, CH_3CN; linear gradient 0–80%B for 40 min; flow rate, 12 mL/min; detection wavelength, 260 nm; column temperature, r.t.). The fractions containing the product were collected and concentrated. The product was desalted through Wakosil®25C18 (particle size, 15–30 µm (spherical); column size, φ = 4.80 cm, h = 10.2 cm, 184 cm^3 (12 mL/min); Solvent A, H_2O (20 minutes), then Solvent B, 90% CH_3CN/H_2O (20 minutes)), and the fractions containing product were concentrated to afford the target compound as triethylammonium salt (46.1 µmol, 56.6% yield). The cap analog triethylammonium salt (46.1 µmol) was dissolved in methanol (4.00 mL). A solution of 0.19 M $NaClO_4$ in acetone (6.00 mL) was added to the methanol solution of cap analog to give a white precipitate. The suspension was diluted with acetone to a total volume of 30 mL. The mixture was centrifuged for 15 minutes at 4,500 rpm. The supernatant was discarded, and the precipitate was resuspended in acetone. The suspending-centrifugation processes were repeated additional 3 times. The precipitate was dried under reduced pressure to afford the target cap analog (**1**) (44.2 mg, 40.6 µmol, 50.0%) as a white solid. The yield was calculated by using the absorbance of the product at 260 nm measured by a NanoDrop 2000 spectrophotometer. Extinction coefficient (ε_{260}) = 26,300 M^{-1}•cm^{-1} was used for calculation[3, 4]. ^1H NMR (400 MHz, D_2O, a mixture of stereoisomers) δ 7.83 (d, J = 11.1 Hz, 1H), 7.53 (dd, J = 8.2, 1.4 Hz, 0.4H), 7.44 (dd, J = 9.1, 7.7 Hz, 1H), 7.42–7.30 (m, 1.6H), 7.30–7.24 (m, 0.4H), 7.17 (ddd, J = 8.4, 6.4, 2.3 Hz, 0.6H), 5.80 (d, J = 4.9 Hz, 0.4H), 5.62 (dt, J = 10.1, 5.1 Hz, 1.6H), 4.92 (s, 0.6H), 4.83 (d, J = 7.5 Hz, 1H), 4.79 (s, 0.4H), 4.75 (d, J = 7.5 Hz, 1H), 4.62 (d, J = 6.8 Hz, 1H), 4.52–4.43 (m, 1.6H), 4.33 (ddd, J = 11.2, 5.3, 3.6 Hz, 1.4H), 4.31–4.02 (m, 7H), 3.94 (s, 1.1H), 3.90 (s, 1.9H), 0.56–0.37 (m, 9H) ppm. ^{13}C NMR (101 MHz, D_2O, a mixture of stereoisomers) δ 158.53, 153.75, 151.33, 149.67, 148.92, 148.37, 137.22, 133.78, 132.19, 129.95, 128.78, 127.91, 123.47, 115.90, 107.78, 95.01, 87.26, 86.74, 85.56, 83.60, 82.00, 80.55, 79.32, 73.87, 70.27, 68.85, 65.52, 36.39, 36.05, 35.67, 24.78, 24.68 ppm. ^{31}P NMR (162 MHz, D_2O, a mixture of stereoisomers) δ -10.87 (dd, 2P, J = 30.1, 19.3 Hz), -22.31 (td, 1P, J = 19.3, 6.4 Hz) ppm. ESI-TOF-MS calcd. for $C_{33}H_{43}N_{11}O_{21}P_3$, 1022.1853 [M - H]$^-$; found 1022.1851.

2.3 Synthesis of 2’-Nb-Guanosine-5’-Diphosphate Imidazolide (16)

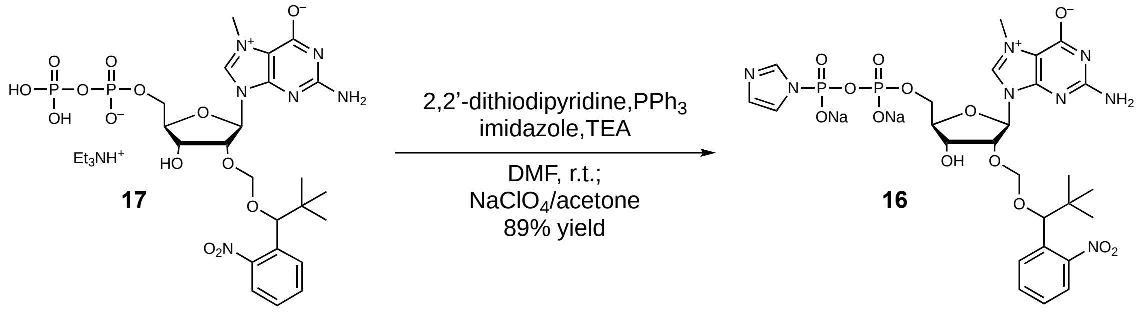

Supplementary Figure 5. Synthesis of 2’-Nb-N^7-Methylguanosine-5’-Diphosphate Imidazolide (**16**).

2’-Nb-*N*⁷-Methylguanosine-5’-Diphosphate Imidazolide (16): To a solution of N^7-methyl-guanosine-5’-diphosphate triethylammonium salt (**17**) (3.74 g, 4.80 mmol), imidazole (2.61 g, 38.4 mmol), and 2,2’-dithiodipyridine (3.17 g, 14.4 mmol) in *N*,*N*-dimethylformamide (57.1 mL) was added triethylamine (0.971 g, 1.34 mL, 9.60 mmol) followed by triphenylphosphine (3.78 g, 14.4 mmol). After being stirred at room temperature for 4 hours, the reaction mixture was added dropwise to a sodium perchlorate (3.53 g, 28.8 mmol) in 4% triethylamine/ dry acetone (720 mL) under stirring. The resulting precipitate was collected by filtration and washed with dry acetone. The solid was dried under reduced pressure to afford N^7-methyl-guanosine 5’-diphosphophosphate phosphorimidazolide disodium salt (**16**) (3.30 g, 88.9% yield) as yellow solid. ^{1}H NMR (600 MHz, DMSO-d_6) δ 9.59, 9.40 (2s, 1H), 7.79 (dd, J = 8.2, 1.4 Hz, 0.5H), 7.73–7.65 (m, 2H), 7.63–7.57 (m, 1.5H), 7.52–7.46 (m, 0.5H), 7.37 (ddd, J = 8.5, 5.9, 2.9 Hz, 0.5H), 7.17 (dd, J = 2.8, 1.4 Hz, 1H), 6.78 (s, 1H), 5.96 (d, J = 4.3 Hz, 0.5H), 5.76 (d, J = 5.4 Hz, 0.5H), 5.72 (d, J = 3.4 Hz, 0.5H), 5.65 (d, J = 5.1 Hz, 0.5H), 5.05 (s, 0.5H), 4.93 (s, 0.5H), 4.87 (d, J = 7.1 Hz, 0.5H), 4.82 (d, J = 7.0 Hz, 0.5H), 4.75 (d, J = 7.1 Hz, 0.5H), 4.57 (t, J = 4.5 Hz, 0.5H), 4.53 (d, J = 7.0 Hz, 0.5H), 4.43–4.34 (m, 1H), 4.27 (q, J = 4.7 Hz, 0.5H), 3.97 (d, J = 11.8 Hz, 4H), 3.92–3.82 (m, 1H), 3.79 (d, J = 9.3 Hz, 1H), 0.71 (s, 5H), 0.64 (s, 4H) ppm. ^{13}C NMR (151 MHz, D_2O, a mixture of stereoisomers) δ 160.10, 149.89, 149.72, 149.38, 148.32, 139.89, 135.18, 133.89, 132.79, 132.64, 132.20, 130.05, 128.78, 128.51, 128.46, 127.89, 123.80, 123.52, 120.82, 120.42, 108.77, 108.48, 95.30, 93.71, 87.22, 87.05, 85.73, 85.32, 82.27, 80.85, 79.71, 79.19, 69.16, 68.78, 65.12, 64.91, 36.54, 35.90, 35.84, 35.74, 30.26, 24.77, 24.69 ppm. ^{31}P NMR (243 MHz, DMSO-d_6) δ -10.73 (m, 1P), -19.22 (m, 1P) ppm. ESI-TOF-MS calcd. for $C_{26}H_{33}N_8O_{13}P_2$, 727.1648 [M - H]$^-$; found 727.1658

2.4 Synthesis of Di-PureCap/ 2'OMe (2)

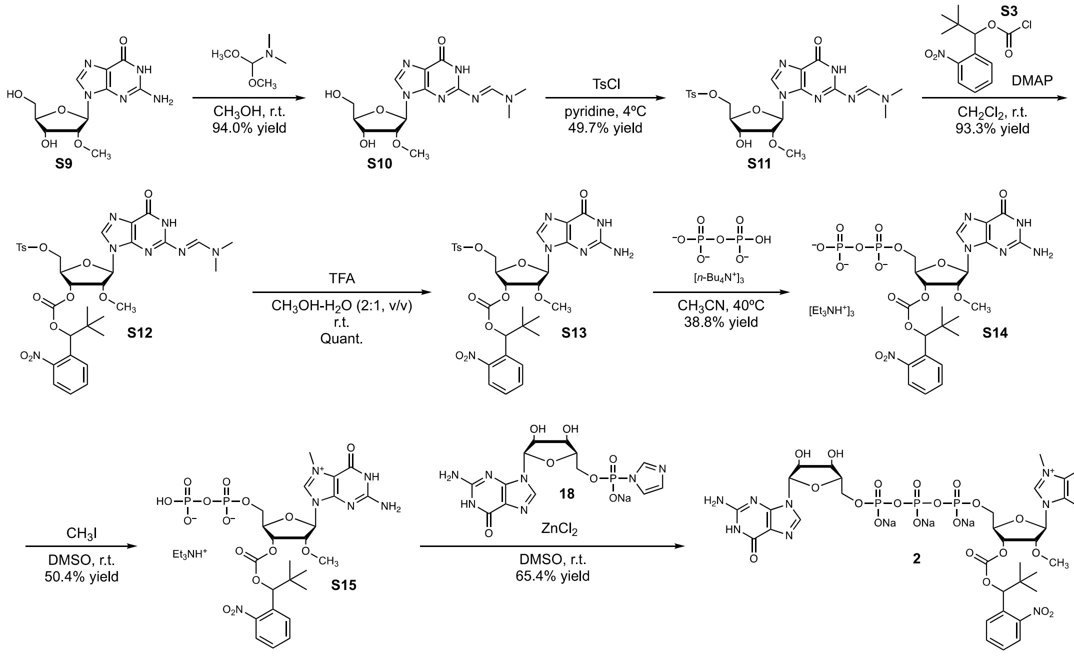

Supplementary Figure 6. Synthesis of Di-PureCap/2'OMe (**2**).

***N*²-Dimethylaminomethylene-2'-*O*-methylguanosine (S10):** To a suspension of 2'-*O*-methylguanosine (3.63 g, 12.2 mmol) in methanol (61.0 mL), *N*,*N*-dimethylformamide dimethyl acetal (7.27 g, 8.17 mL, 61.0 mmol) was added. After being stirred for 21 hours at room temperature, the reaction mixture was concentrated by a rotary evaporator. The oily residue was dissolved in methanol (10.0 mL), and a few drops of ether were added. The mixture was sonicated and then suspended by adding ether (150 mL). The resulting precipitate was collected by filtration and washed with ether. The solid was dried under reduced pressure to give the compound **S10** (4.08 g, 94.0) as white powder. All the physical data matched the previously reported data[5].

***N*²-Dimethylaminomethylene-2'-*O*-methyl-5'-*O*-Tosyl-guanosine (S11):** To a cooled solution of the compound **S10** (1.00 g, 2.84 mmol) in pyridine (22.7 mL), a solution of *p*-toluenesulfonyl chloride (812 mg, 4.26 mmol) in pyridine (5.70 mL) was added dropwise in an ice bath. The mixture was left in a refrigerator at 4 °C for 17 hours, and then a solution of *p*-toluenesulfonyl chloride (271 mg, 1.42 mmol) in pyridine (1.00 mL) was added. The mixture was left in a refrigerator at 4 °C for 17 hours. The reaction mixture was quenched by pouring it into water. The mixture was extracted 2 times with dichloromethane. The organic layer was dried over Na_2SO_4 and evaporated. The residue was subjected to silica gel column chromatography eluted by 1.6–9.1% methanol/dichloromethane to afford the 5'-*O*-tosylated

compound **S11** (682 mg, 47.4% yield) as white foam and the 3',5'-bis-tosylated side-product (167 mg, 8.91% yield) as white solid. ^{1}H NMR (400 MHz, $CDCl_3$) δ 10.21–10.00 (m, 1H), 8.46 (s, 1H), 7.70 (s, 1H), 7.63 (d, J = 8.4 Hz, 2H), 7.17 (d, J = 8.4 Hz, 2H), 5.93 (d, J = 4.1 Hz, 1H), 5.24 (s, 1H), 4.60 (t, J = 5.4 Hz, 1H), 4.33–4.15 (m, 4H), 3.44 (s, 3H), 3.08 (s, 3H), 2.99 (s, 3H), 2.30 (s, 3H) ppm. ^{13}C NMR (101 MHz, $CDCl_3$) δ 158.44, 158.38, 157.14, 150.29, 145.36, 136.69, 132.27, 129.94, 127.88, 120.31, 86.32, 83.03, 81.66, 69.41, 69.02, 58.82, 58.59, 53.62, 41.66, 41.48, 35.27, 31.00, 21.75, 21.65 ppm. ESI-TOF-MS calcd. for $C_{21}H_{27}N_6O_7S$, 507.1656 [M + H]$^+$; found 507.1675, calcd for $C_{21}H_{26}N_6NaO_7S$, 529.1476 [M + Na]$^+$; found 529.1495.

***N*^{2}-Dimethylaminomethylene-2'-*O*-methyl-3'-Nb-5'-*O*-Tosyl-guanosine (S12):** To a cooled solution of the chloroformate derivative (**S3**) (107 mg, 0.394 mmol) in dichloromethane (985 µL), 5'-*O*-Tosylated guanosine analog (**S11**) (100 mg, 0.197 mmol) and DMAP (48.1 mg, 0.394 mmol) were added in an ice bath. After being stirred for 20 hours at room temperature, the reaction mixture was diluted with dichloromethane and washed with saturated *aq.* NH_4Cl. The organic layer was dried over Na_2SO_4 and evaporated. The residue was subjected to silica gel column chromatography eluted by 2.0–4.8% methanol/dichloromethane to afford compound **S12** (126 mg, 93.3% yield) as white solid. ^{1}H NMR (600 MHz, $CDCl_3$) δ 9.73–9.60 (m, 1H), 8.61 (s, 0.5H), 8.58 (s, 0.5H), 7.88 (ddd, J = 8.8, 4.0, 1.2 Hz, 1H), 7.67–7.49 (m, 7H), 7.49–7.40 (m, 1H), 7.40–7.27 (m, 1H), 7.21–7.09 (m, 3H), 6.41 (s, 0.5H), 6.32 (s, 0.5H), 5.71 (td, J = 11.0, 4.5 Hz, 1H), 5.67–5.61 (m, 1H), 4.68 (dd, J = 5.7, 3.1 Hz, 0.5H), 4.58 (dd, J = 5.7, 3.5 Hz, 0.5H), 4.33–4.13 (m, 4H), 3.37 (s, 2H), 3.21 (s, 1.5H), 3.08 (d, J = 2.1 Hz, 3H), 3.03 (s, 1.5H), 2.92 (s, 1.5H), 2.33 (d, J = 9.0 Hz, 3H), 0.95 (s, 4.5H), 0.93 (s, 4.5H) ppm. ^{13}C NMR (151 MHz, $CDCl_3$) δ 158.71, 158.66, 158.47, 158.15, 158.09, 157.24, 154.28, 154.02, 149.90, 149.31, 149.09, 145.91, 145.62, 145.51, 137.64, 132.93, 132.72, 132.59, 132.37, 132.13, 130.19, 129.93, 129.80, 129.76, 129.68, 129.26, 129.04, 129.00, 128.77, 128.01, 127.86, 127.80, 127.74, 124.81, 124.58, 121.22, 120.92, 88.19, 88.01, 87.36, 81.02, 80.93, 80.83, 80.78, 80.69, 80.60, 80.49, 80.16, 79.10, 78.94, 78.52, 78.20, 78.11, 75.84, 75.59, 75.20, 74.58, 74.07, 73.89, 67.70, 67.61, 67.55, 66.08, 65.30, 59.58, 59.40, 59.20, 58.94, 58.88, 53.58, 41.56, 41.43, 41.36, 41.05, 36.59, 36.34, 36.29, 36.25, 35.40, 35.34, 25.78, 25.74, 25.71, 25.60, 21.79, 21.68 ppm. ESI-TOF-MS calcd for $C_{33}H_{40}N_7O_{11}S$, 742.2502 [M + H]$^+$; found 742.2562, calcd. for $C_{33}H_{39}N_7NaO_{11}S$, 764.2321 [M + Na]$^+$; found 764.2313.

2'-*O*-methyl-3'-Nb-5'-*O*-Tosyl-guanosine (S13): To a suspension of the protected guanosine (**S12**) (591 mg, 0.797 mmol) in methanol (13.3 µL) and water (6.63 mL) was added

trifluoroacetic acid (6.82 g, 4.68 mL, 59.8 mmol). After being stirred for 23 hours at room temperature, the reaction mixture was concentrated to remove methanol. The residue was diluted with dichloromethane and washed with water. The aqueous layer was dried over Na_2SO_4 and evaporated to give compound **S13** (576 mg, quant.) as gray foam. ^1H NMR (600 MHz, $CDCl_3$) *δ* 7.91–7.86 (m, 1H), 7.75–7.66 (m, 3H), 7.66–7.60 (m, 2H), 7.29–7.21 (m, 3H), 6.43 (s, 0.5H), 6.30 (s, 0.5H), 5.75 (dd, *J* = 6.3, 3.7 Hz, 1H), 5.29–5.21 (m, 1H), 4.73 (t, *J* = 5.8 Hz, 0.5H), 4.63 (t, *J* = 5.7 Hz, 0.5H), 4.40–4.35 (m, 1H), 4.31 (dd, *J* = 7.9, 3.9 Hz, 1H), 4.29–4.20 (m, 1H), 3.31 (s, 1.5H), 2.97 (s, 1.5H), 2.36 (d, *J* = 9.7 Hz, 3H), 0.95 (d, *J* = 11.2 Hz, 9H) ppm. ^{13}C NMR (151 MHz, $CDCl_3$) *δ* 159.21, 154.10, 153.77, 151.66, 149.39, 149.25, 145.58, 136.77, 132.81, 132.70, 132.56, 132.41, 132.09, 130.17, 130.13, 130.10, 129.69, 129.27, 128.97, 128.89, 128.82, 128.07, 128.02, 127.99, 127.96, 126.45, 124.66, 120.91, 117.56, 86.95, 86.86, 80.88, 80.67, 80.54, 80.16, 80.10, 79.75, 79.69, 74.36, 74.21, 68.40, 59.66, 59.39, 59.21, 36.59, 36.32, 36.24, 35.44, 25.81, 25.73, 25.70, 25.61, 21.80, 21.71, 21.68 ppm. ESI-TOF-MS calcd. for $C_{30}H_{35}N_6O_{11}S$, 687.2079 $[M + H]^+$; found 687.2091.

2'-*O*-methyl-3'-Nb-guanosine-5'-diphosphate (S14): To a suspension of 5'-*O*-Tosyl-guanosine (**S13**) (53.3 mg, 71.9 μmol) and powdered 3A molecular sieves (55.0 mg) in CH_3CN (80.0 μL), 616 mM tris-tetrabutylammonium pyrophosphate in CH_3CN (175 μL, 108 μmol) was added. After stirring for 19 hours at 40 ºC, the reaction mixture was diluted with water. The resulting suspension was centrifuged for 30 minutes at 4,500 rpm. The supernatant was collected, and the precipitate was resuspended in water. The resulting suspension was centrifuged for 30 minutes at 4,500 rpm. The supernatant was collected, and the combined supernatant was purified by ion-exchange chromatography on QAE-Sephadex (φ = 3.0 cm, *h* = 10 cm, 70 cm^3, 6 mL/min) in a linear 0–1.5 M gradient of TEAB buffer (pH 7.9) containing 0–10% CH_3CN (270 minutes). The fractions containing the product were collected and concentrated to afford the modified guanosine diphosphate (**S14**) (56.9 mg, 57.1 mol, 79.7% yield) as triethylammonium salt. ^1H NMR (400 MHz, CD_3OD) *δ* 8.23–8.02 (m, 1H), 7.92 (d, *J* = 8.2 Hz, 1H), 7.69 (q, *J* = 6.3 Hz, 2H), 7.62–7.47 (m, 1H), 6.36 (s, 0.5H), 6.23 (s, 0.5H), 5.83 (dd, *J* = 7.6, 2.9 Hz, 1H), 5.49 (dd, *J* = 15.8, 5.0 Hz, 1H), 4.48–4.08 (m, 4H), 3.32 (s, 3H), 3.15 (q, *J* = 7.3 Hz, 18H), 1.28 (q, *J* = 11.2 Hz, 40H), 0.97 (d, *J* = 8.8 Hz, 9H) ppm. ^{13}C NMR (101 MHz, CD_3OD) *δ* 158.15, 154.23, 153.93, 153.86, 149.52, 132.55, 132.16, 129.13, 129.05, 128.85, 126.03, 125.62, 124.27, 122.87, 85.84, 81.96, 80.80, 80.70, 80.23, 79.94, 75.54, 64.94, 58.41, 58.14, 58.03, 45.98, 42.24, 41.75, 36.10, 35.71, 30.37, 24.94, 24.77, 19.24, 10.25, 10.05, 7.75, 6.88 ppm. ^{31}P NMR (162 MHz, CD_3OD) *δ* -9.04 (1P), -9.44 (0.5P), -10.75 (0.5P) ppm. ESI-TOF-MS calcd. for $C_{23}H_{29}N_6O_{15}P_2$, 691.1172 $[M - H]^-$; found 691.1270.

***N*^7-Methyl-2'-*O*-methyl-3'-Nb-guanosine-5'-diphosphate (S15):** To a solution of modified-guanosine-5'-diphosphate (**S14**) (180 μmol) in DMSO (1.50 mL) was added iodomethane (204 mg, 89.6 μL, 1.44 mmol). After stirring at room temperature for 13 hours, the reaction mixture was diluted with water and washed 3 times with dichloromethane. The crude was purified by ion-exchange chromatography on QAE-Sephadex™ A-25 (φ = 4.5 cm, *h* = 8.4 cm, 140 cm^3, 12 mL/min) with a linear 0–0.8 M gradient of TEAA buffer (pH 6.0) containing 0–8% CH_3CN (270 minutes). After the removal of CH_3CN under reduced pressure, the product was desalted through Wakosil® 25C18 (particle size, 15–30 μm (spherical); column size, φ = 4.80 cm, *h* = 10.2 cm, 184 cm^3 (12 mL/min); Solvent A, H_2O (20 minutes), then Solvent B, 90% CH_3CN/H_2O (20 minutes)), and the fractions containing product were concentrated to afford compound **S15** (73.3 mg, 90.8 μmol, 50.4% yield) as triethylammonium salt. ^1H NMR (600 MHz, D_2O) δ 7.89–7.70 (m, 1H), 7.66–7.33 (m, 3H), 6.18 (s, 0.5H), 6.00 (d, *J* = 11.2 Hz, 1.5H), 5.47 (s, 0.5H), 4.62 (d, *J* = 5.6 Hz, 0.5), 4.67 (m, 0.5H), 4.55 (s, 0.5H), 4.50–4.38 (m, 1H), 4.18 (s, 0.5H), 4.06 (s, 1.5H), 3.99 (s, 3H), 3.30 (s, 1.5H), 3.07 (qd, *J* = 7.4, 2.1 Hz, 6H), 2.99 (s, 1.5H), 1.14 (td, *J* = 7.3, 2.2 Hz, 9H), 0.74 (d, *J* = 12.1 Hz, 9H) ppm. ^{13}C NMR (151 MHz, D_2O) δ 155.70, 155.63, 155.09, 154.94, 154.35, 153.94, 150.05, 149.87, 148.91, 148.65, 133.38, 133.05, 131.87, 131.18, 129.43, 129.22, 128.59, 124.70, 124.63, 108.16, 108.01, 87.55, 86.87, 83.55, 83.02, 82.51, 82.20, 81.55, 81.15, 75.27, 74.81, 69.65, 64.65, 64.44, 59.06, 58.99, 46.71, 36.20, 36.13, 35.58, 24.95, 24.92, 8.28 ppm. ^{31}P NMR (243 MHz, D_2O) δ -9.64 (1P), -10.72 (1P) ppm. ESI-TOF-MS calcd. for $C_{24}H_{31}N_6O_{15}P_2$, 705.1328 $[M - H]^-$; found 705.1404.

2'-*O*-methyl-3'-Nb-PureCap Analog, DiPure/ 2'OMe (2): *N*^7-Methyl-2'-*O*-methyl-3'-Nb-guanosine-5'-diphosphate triethylammonium salt (**S15**) (7.60 mg, 9.40 μmol) and guanosine 5'-monophosphate imidazolide (**18**) (12.3 mg, 28.2 μmol) were dissolved in 0.59 M zinc chloride/DMSO solution (493 μL, $ZnCl_2$: 291 μmol). After stirring at 37 ºC for 2 days, the reaction mixture was quenched by adding 200 mM EDTA *aq.* (pH 7.0) (1.60 mL, EDTA: 320 μmol) and 2.0 M TEAB buffer (pH 7.9, 100 μL). The mixture was purified by reverse-phase HPLC using a YMC-Triart-C8 column (250 × 4.6 mm I.D., S-5 μm, 12 nm, flow rate 1.0 mL/min, temperature: 50 ºC) with a linear gradient of 5–80% of CH_3CN in 0.1 M triethylammonium bicarbonate buffer (pH 7.9) in 25 minutes. The fractions containing the product were concentrated to afford the target compound (8.33 mg, 6.15 μmol, 65.4% yield) as triethylammonium salt. The product was lyophilized, and the solid was redissolved in methanol (2.0 mL). 190 mM $NaClO_4$ in acetone (10 mL) was added to the mixture, and the resulting suspension was centrifuged (4,000 rpm, 20 minutes). The supernatant was

discarded, and the precipitate was resuspended in acetone. The suspending-centrifugation processes were repeated additional four times. The precipitate was dried under reduced pressure to afford the target cap analog (**2**) (3.46 mg, 3.10 μmol, 33.0% yield) as the sodium salt. The yields were calculated by using the absorbance of the product at 260 nm measured by a NanoDrop 2000 spectrophotometer. Extinction coefficient (ε_{260}) = 26,300 M^{-1}•cm^{-1} was used for calculation[3, 4].

[TEA salt] ^{1}H NMR (600 MHz, CD_3OD, triethylammonium salt) *δ* 7.99 (d, *J* = 5.4 Hz, 1H), 7.92 (q, *J* = 8.0 Hz, 1H), 7.77–7.61 (m, 2H), 7.59–7.49 (m, 1H), 6.41–6.29 (m, 0.5H), 6.27–6.17 (m, 0.5H), 6.04–5.93 (m, 1H), 5.76 (s, 1H), 5.30 (s, 1H), 4.76–4.67 (m, 1H), 4.61 (s, 1H), 4.58–4.38 (m, 4H), 4.27 (s, 2H), 4.23–4.13 (m, 3H), 4.13–3.99 (m, 3H), 3.53–3.43 (m, 1.5H), 3.08 (d, *J* = 7.9 Hz, 1.5H), 0.96 (d, *J* = 8.4 Hz, 9H) ppm. ^{13}C NMR (151 MHz, CD_3OD, triethylammonium salt) *δ* 159.34, 157.66, 155.50, 155.17, 155.02, 153.03, 150.94, 150.78, 138.22, 133.97, 133.82, 133.27, 130.47, 130.35, 130.12, 125.63, 117.77, 108.69, 89.71, 89.29, 85.29, 84.35, 84.11, 84.02, 81.73, 81.48, 75.66, 75.20, 71.92, 66.63, 65.32, 60.04, 59.78, 47.43, 37.51, 37.09, 36.97, 26.28, 26.13, 23.46, 9.14 ppm. ^{31}P NMR (243 MHz, CD_3OD, triethylammonium salt) *δ* -10.53 to -11.77 (m, 2P), -22.19 (s, 1P) ppm.

[Na salt] ^{1}H NMR (600 MHz, D_2O, sodium salt) *δ* 7.87–7.80 (m, 2H), 7.68–7.54 (m, 2H), 7.45 (t, *J* = 7.7 Hz, 1H), 6.19 (s, 0.5H), 6.04 (s, 0.5H), 5.78 (d, *J* = 5.4 Hz, 1H), 5.62 (t, *J* = 6.4 Hz, 1H), 5.27–5.22 (m, 0.5H), 5.17 (t, *J* = 4.3 Hz, 0.5H), 4.54–4.47 (m, 1H), 4.45–4.38 (m, 1H), 4.35 (t, *J* = 5.4 Hz, 0.5H), 4.31 (q, *J* = 5.4 Hz, 1H), 4.25–4.04 (m, 5.5H), 3.95 (d, *J* = 5.4 Hz, 3H), 3.21 (s, 1.5H), 2.95 (s, 1.5H), 0.82 (d, *J* = 13.4 Hz, 9H) ppm. ^{31}P NMR (243 MHz, D_2O, sodium salt) *δ* -10.97 (dd, *J* = 36.2, 19.7 Hz), -22.56 (t, *J* = 19.7 Hz) ppm. ESI-TOF-MS calcd. for $C_{34}H_{43}N_{11}O_{22}P_3$, 1050.1802 $[M - H]^-$; found 1050.1836.

2.5 Synthesis of Di-PureCap/ 3'OMe (3)

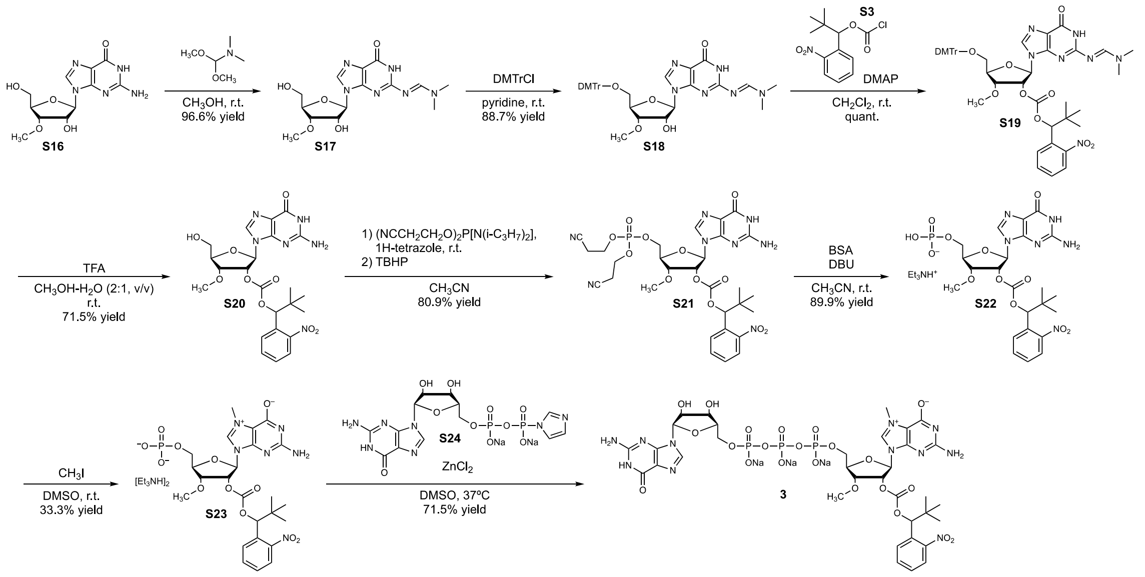

Supplementary Figure 7. Synthesis of Di-PureCap/ 3'OMe (**3**).

***N*²-Dimethylaminomethylene-3'-*O*-Methyl-Guanosine (S17):** To a suspension of 3'-*O*-methylguanosine (**S16**) (1.50 g, 5.05 mmol) in methanol (25.3 mL), *N*,*N*-dimethylformamide dimethyl acetal (3.01 g, 3.39 mL, 25.3 mmol) was added. After being stirred for 18 hours at room temperature, the reaction mixture was concentrated by a rotary evaporator. The oily residue was dissolved in dichloromethane (10.0 mL) and suspended by adding ether. The resulting precipitate was collected by filtration and washed with ether. The solid was dried under reduced pressure to give compound **S17** (1.72 g, 96.6% yield) as white powder. ^1H NMR (600 MHz, DMSO-d_6) δ 11.35 (s, 1H), 8.54 (s, 1H), 8.04 (s, 1H), 5.78 (d, J = 6.3 Hz, 1H), 5.52 (s, 1H), 5.15 (s, 1H), 4.68–4.63 (m, 1H), 3.99 (q, J = 4.0 Hz, 1H), 3.84 (dd, J = 5.0, 3.3 Hz, 1H), 3.66–3.60 (m, 1H), 3.53 (d, J = 13.3 Hz, 1H), 3.41 (s, 3H), 3.16 (s, 3H), 3.03 (s, 3H) ppm. ^{13}C NMR (151 MHz, DMSO-d_6) δ 157.97, 157.45, 150.02, 136.87, 119.85, 86.88, 82.96, 79.86, 73.02, 64.93, 61.47, 57.61, 40.64, 34.65 ppm. ESI-TOF-MS calcd. for $C_{14}H_{20}N_6NaO_5$, 375.1388 $[M + Na]^+$; found 375.1353.

***N*²-Dimethylaminomethylene-3'-*O*-Methyl-5'-*O*-DMTr-Guanosine (S18):** To a solution of the *N*²-protected-3'-*O*-methylguanosine (**S17**) (195 mg, 0.553 mmol) in pyridine (2.77 mL), DMTrCl (244 mg, 0.719 mmol) was added. After stirring for 12 hours at room temperature, DMTrCl (93.9 mg, 0.277 mmol) was added. The reaction mixture was stirred at room temperature for 3.5 hours and then quenched by adding methanol (1.00 mL). The mixture was diluted with dichloromethane and washed with saturated *aq.* $NaHCO_3$. The organic layer was dried over Na_2SO_4 and concentrated by a rotary evaporator. The crude material was subjected to silica gel column chromatography eluted by 1.6–4.8%

methanol/dichloromethane containing 1.0% triethylamine to afford compound **S18** (337 mg, 93.1% yield) as white foam. ^1H NMR (400 MHz, $CDCl_3$, containing triethylamine) *δ* 8.37 (s, 1H), 7.66 (s, 1H), 7.36–7.29 (m, 2H), 7.25–7.04 (m, 7H), 6.75–6.67 (m, 4H), 5.91 (d, *J* = 5.9 Hz, 1H), 4.73 (t, *J* = 5.7 Hz, 1H), 4.17 (q, *J* = 4.1 Hz, 1H), 3.93–3.87 (m, 1H), 3.66 (d, *J* = 1.2 Hz, 6H), 3.37 (s, 3H), 3.32 (dd, *J* = 10.6, 4.0 Hz, 1H), 3.22 (dd, *J* = 10.6, 4.5 Hz, 1H), 2.87 (d, *J* = 8.6 Hz, 6H), 2.43 (q, *J* = 7.2 Hz, 15H), 0.92 (t, *J* = 7.2 Hz, 22H) ppm. ^{13}C NMR (101 MHz, $CDCl_3$, containing triethylamine) *δ* 158.56, 158.16, 156.99, 150.69, 144.65, 136.21, 135.69, 130.04, 128.10, 127.95, 126.93, 120.26, 113.21, 87.65, 86.46, 81.54, 80.25, 73.84, 63.68, 58.29, 55.20, 46.15, 41.27, 35.09, 11.51 ppm. ESI-TOF-MS calcd. for $C_{35}H_{39}N_6O_7$, 655.2875 $[M + H]^+$; found 655.2976.

***N*^2^-Dimethylaminomethylene-2'-Nb-3'-*O*-Methyl-5'-*O*-DMTr-Guanosine (S19):** To a cooled solution of the protected-guanosine (**S18**) (531 mg, 0.811 mmol) and the chloroformate derivative **S3** (439 mg, 1.62 mmol) in dichloromethane (4.06 mL), DMAP (198 mg, 1.62 mmol) was added in an ice bath. After being stirred for 18 hours at room temperature, the reaction mixture was diluted with dichloromethane and washed with saturated *aq.* NH_4Cl. The organic layer was dried over Na_2SO_4 and basified by adding triethylamine (4.00 mL, 4%). The solvents were evaporated. The residue was subjected to silica gel column chromatography eluted by 2.4–6.3% methanol/dichloromethane containing triethylamine to afford compound **S19** (745 mg, quant.) as white amorphous solid. ^1H NMR (600 MHz, $CDCl_3$) *δ* 10.20 (s, 1H), 8.56 (s, 0.4H), 8.47 (s, 0.6H), 7.87–7.80 (m, 1H), 7.70 (s, 0.4H), 7.66 (s, 0.6H), 7.60–7.53 (m, 0.8H), 7.52–7.49 (m, 1.2H), 7.42–7.34 (m, 3H), 7.28–7.24 (m, 4H), 7.23–7.19 (m, 2H), 7.17–7.12 (m, 1H), 6.79–6.73 (m, 4H), 6.45 (s, 0.6H), 6.30 (s, 0.4H), 6.03 (dd, *J* = 6.8, 4.3 Hz, 1H), 6.02–5.94 (m, 1H), 4.23 (dt, *J* = 5.6, 3.7 Hz, 1H), 4.13 (t, *J* = 5.1 Hz, 0.6H), 4.00 (dd, *J* = 6.1, 4.9 Hz, 0.4H), 3.72 (d, *J* = 1.1 Hz, 6H), 3.45–3.40 (m, 1H), 3.33 (s, 1.8H), 3.27 (td, *J* = 11.3, 4.1 Hz, 1H), 3.09 (s, 1.2H), 3.06–3.05 (m, 1.2H), 3.01–2.99 (m, 1.8H), 2.97 (s, 1.2H), 2.94 (s, 1.8H), 0.91 (d, *J* = 5.8 Hz, 9H) ppm. ^{13}C NMR (151 MHz, $CDCl_3$) *δ* 158.75, 158.63, 158.51, 158.29, 158.26, 157.13, 157.08, 153.75, 153.56, 150.03, 149.80, 149.10, 148.91, 144.28, 136.42, 136.19, 135.48, 132.67, 132.45, 132.05, 129.96, 129.74, 129.08, 128.95, 128.71, 128.56, 128.07, 127.88, 126.90, 124.55, 124.35, 120.84, 120.76, 113.16, 86.47, 86.44, 86.15, 85.55, 81.74, 81.58, 80.77, 80.41, 78.23, 77.94, 76.31, 76.21, 62.84, 62.68, 58.83, 58.65, 55.17, 46.12, 41.33, 41.14, 36.51, 36.11, 35.12, 35.05, 25.72, 25.64, 25.44, 11.47 ppm. ESI-TOF-MS calcd. for $C_{47}H_{51}N_7NaO_{11}$, 912.3539 $[M + Na]^+$; found 912.3644.

2'-Nb-3'-*O*-Methyl-Guanosine (S20): To a suspension of the protected-guanosine (**S19**)

(56.9 mg, 63.9 µmol) in methanol (1.07 mL) and water (533 µL) was added trifluoroacetic acid (546 mg, 367 µL, 4.79 mmol). After being stirred for 19 hours at room temperature, the reaction mixture was diluted with a mixture of methanol/water (2:1, v/v, 5.00 mL). The mixture was concentrated and purified by silica gel column chromatography eluted by 1.9–9.1% methanol/dichloromethane to afford compound **S20** (24.3 mg, 71.5% yield) as white solid. ^{1}H NMR (600 MHz, DMSO-d_6) δ 10.73 (d, J = 9.5 Hz, 1H), 8.01–7.93 (m, 2H), 7.81–7.68 (m, 1H), 7.64–7.52 (m, 2H), 6.54 (s, 2H), 6.18 (s, 0.6H), 6.02 (s, 0.4H), 5.96 (d, J = 5.5 Hz, 1H), 5.48 (q, J = 5.1 Hz, 1H), 4.22 (t, J = 4.7 Hz, 0.5H), 4.08–3.95 (m, 1.5H), 3.68–3.60 (m, 1H), 3.58–3.50 (m, 2H), 3.35 (s, 2H), 2.98 (s, 1H), 0.87 (d, J = 3.7 Hz, 9H) ppm. ^{13}C NMR (151 MHz, DMSO-d_6) δ 156.61, 156.57, 153.91, 153.88, 153.10, 152.99, 150.98, 150.92, 149.08, 148.86, 135.66, 135.55, 133.05, 131.36, 130.78, 129.60, 129.47, 128.89, 128.51, 124.51, 124.48, 116.54, 116.47, 84.03, 82.96, 82.89, 79.93, 79.65, 77.90, 77.75, 77.27, 77.23, 60.91, 60.86, 58.05, 57.71, 36.02, 35.59, 25.44, 25.21 ppm. ESI-TOF-MS calcd. for $C_{23}H_{29}N_6O_9$, 533.1991 [M + H]$^+$; found 533.2020.

2’-Nb-3’-*O*-Methyl-Guanosine 5’-bis(cyanoethyl)monophosphate (S21): 2’-Nb-3’-*O*-methyl-guanosine (**S20**) (290 mg, 0.545 mmol) was added to a solution of bis(2-cyanoethyl) *N*,*N*-diisopropylphosphoramidite (222 mg, 0.818 mmol) in CH_3CN (5.45 mL). 1*H*-Tetrazole (57.3 mg, 0.818 mmol) was added to the mixture. After stirring at room temperature for 1.5 hours, the reaction mixture was added to 5–6 M *tert*-butyl hydroperoxide in decane solution (218 µL, 1.09–1.31 mmol). The reaction mixture was stirred at room temperature for 6 minutes and diluted with dichloromethane. The mixture was washed with saturated *aq.* $NaHCO_3$. The organic layer was dried over Na_2SO_4 and concentrated. The residual material was purified by silica gel column chromatography eluted by 4.8–17% methanol/dichloromethane to afford compound **S21** (317 mg, 80.9% yield) as brown amorphous solid. ^{1}H NMR (600 MHz, $CDCl_3$) δ 12.25 (s, 0.5H), 7.88 (d, J = 8.2 Hz, 0.5H), 7.81 (d, J = 8.0 Hz, 0.5H), 7.74 (d, J = 16.2 Hz, 1H), 7.66–7.60 (m, 1H), 7.59–7.50 (m, 1H), 7.46 (ddd, J = 8.5, 5.9, 2.9 Hz, 0.5H), 7.40 (t, J = 7.8 Hz, 0.6H), 6.60 (s, 1.5H), 6.35 (s, 0.5H), 6.22 (s, 0.5H), 5.95 (d, J = 4.4 Hz, 1H), 5.73 (dd, J = 10.3, 5.4 Hz, 1H), 4.53–4.32 (m, 2.5H), 4.25 (tq, J = 11.5, 5.4 Hz, 5.5H), 3.44 (s, 1.5H), 3.03 (s, 1.5H), 2.75 (dq, J = 12.6, 6.8 Hz, 4H), 0.94 (s, 9H) ppm. ^{13}C NMR (151 MHz, $CDCl_3$) δ 158.89, 154.01, 153.97, 153.81, 153.60, 151.23, 149.48, 149.26, 137.08, 132.74, 132.58, 131.98, 129.35, 129.10, 128.90, 124.62, 124.52, 117.55, 117.50, 117.12, 117.00, 86.69, 86.57, 80.98, 80.74, 78.01, 77.77, 76.37, 67.07, 62.85, 62.81, 59.18, 58.94, 36.52, 36.16, 25.83, 25.63, 19.69, 19.63 ppm. ^{31}P NMR (243 MHz, $CDCl_3$) δ -1.83 and 1.87 ppm. ESI-TOF-MS calcd. for $C_{29}H_{35}N_8NaO_{12}P$, 741.2005 [M + Na]$^+$; found 741.1980.

2'-Nb-3'-*O*-Methyl-Guanosine 5'-Monophosphate (S22): To a solution of the protected nucleoside monophosphate (**S21**) (317 mg, 0.441 mmol) in acetonitrile (4.41 mL), *N*,*O*-bis(trimethylsilyl)acetamide (897 mg, 1.08 mL, 4.41 mmol) and 1,8-diazabicyclo[5.4.0]undec-7-ene (268 mg, 263 µL, 1.76 mmol) were successively added. After stirring at room temperature for 3 hours, the reaction mixture was quenched by adding 1 M TEAA buffer (pH 6.0 1.50 mL). The mixture was diluted with water (25.0 mL) and washed 3 times with dichloromethane (20.0 mL). The aqueous layer was purified by using Wakosil® 25C18 (particle size, 15–30 µm (spherical); column size, φ = 4.80 cm, h = 10.2 cm, 184 cm^3 (12 mL/min); Solvent A, 50 mM TEAA buffer (pH 6.0) containing 0.5% CH_3CN; Solvent B, CH_3CN; linear gradient 0–80%B over 60 minutes). The fractions containing the target product were combined, concentrated, and lyophilized to afford compound **S22** (347 mg, 89.9% yield, mono-triethylammonium salt contains three equivalents of TEAA salt) as brown powder. ^1H NMR (600 MHz, CD_3OD) δ 8.11–8.01 (m, 1H), 7.96–7.85 (m, 1H), 7.73 (t, J = 7.7 Hz, 0.5H), 7.65 (dt, J = 8.0, 5.8 Hz, 1H), 7.60–7.49 (m, 1.5H), 6.26 (s, 0.5H), 6.17 (s, 0.5H), 6.07 (dd, J = 11.3, 5.6 Hz, 1H), 5.61 (q, J = 5.1 Hz, 1H), 4.37 (t, J = 4.4 Hz, 0.5H), 4.33–4.21 (m, 1.5H), 4.16 (q, J = 7.3 Hz, 1H), 4.12–4.00 (m, 1H), 3.47 (s, 1.5H), 3.15 (q, J = 7.4 Hz, 24H, triethylammonium), 3.11 (s, 1.5H), 1.93 (s, 9H, Acetate), 1.27 (t, J = 7.4 Hz, 36H, triethylammonium), 0.92 (d, J = 6.3 Hz, 9H) ppm. ^{13}C NMR (151 MHz, CD_3OD) δ 179.20, 159.53, 159.46, 155.91, 155.84, 154.88, 154.79, 152.96, 152.92, 150.76, 150.55, 138.06, 137.93, 133.90, 133.52, 133.15, 130.40, 130.34, 130.05, 130.03, 125.56, 125.51, 117.74, 117.53, 86.77, 86.50, 83.77, 83.72, 81.68, 81.53, 80.21, 79.86, 78.97, 78.80, 65.80, 65.77, 65.64, 59.17, 59.01, 47.10, 47.07, 37.24, 36.99, 26.19, 26.09, 23.81, 23.79, 9.05 ppm. ^{31}P NMR (243 MHz, CD_3OD) δ -1.53 and 1.48 ppm. ESI-TOF-MS calcd. for $C_{23}H_{28}N_6O_{12}P$, 611.1508 $[M - H]^-$; found 611.1504.

***N*^{7}-Methyl-2'-Nb-3'-*O*-Methyl-Guanosine 5'-Monophosphate (S23):** To a solution of the modified guanosine 5'-monophosphate (**S22**) (61.6 mg, 75.6 µmol) in DMSO (630 µL) was added iodomethane (85.9 mg, 37.7 µL, 605 µmol). After stirring at room temperature for 7 hours, the reaction mixture was diluted with water and washed 3 times with dichloromethane. The aqueous layer was purified by using Wakosil® 25C18 (particle size, 15–30 µm (spherical); column size, φ = 4.80 cm, h = 10.2 cm, 184 cm^3 (12 mL/min); Solvent A, 50 mM TEAA buffer (pH 6.0) containing 0.5% CH_3CN; Solvent B, CH_3CN; linear gradient 0–80%B over 60 minutes). The fractions containing the target product were combined, concentrated, and lyophilized to afford compound **S23** (18.3 mg, 33.3% yield) as triethylammonium salt. ^1H NMR (600 MHz, D_2O, triethylammonium salt) δ 7.86 (dd, J = 8.2, 1.3 Hz, 0.4H), 7.79 (dd, J

= 8.2, 1.4 Hz, 0.6H), 7.73–7.67 (m, 0.4H), 7.64–7.59 (m, 1H), 7.57–7.44 (m, 1.6H), 6.19 (d, *J* = 3.7 Hz, 0.4H), 6.09 (d, *J* = 5.7 Hz, 0.6H), 6.04 (s, 0.5H), 5.96 (s, 0.5H), 5.62–5.55 (m, 1H), 4.44 (dt, *J* = 8.8, 3.1 Hz, 1H), 4.35 (dd, *J* = 5.0, 3.0 Hz, 1H), 4.27 (t, *J* = 5.1 Hz, 0.5H), 4.16–4.10 (m, 0.5H), 4.08–4.02 (m, 3H), 3.98–3.92 (m, 1H), 3.49 (s, 2H), 3.17 (q, *J* = 7.4 Hz, 18H triethylammonium), 3.14 (s, 1H), 1.89 (s, 3H, acetate), 1.25 (t, *J* = 7.4 Hz, 27H, triethylammonium), 0.86 (d, *J* = 7.0 Hz, 9H) ppm. ^{13}C NMR (151 MHz, D_2O, triethylammonium salt) δ 181.47, 160.00, 159.51, 154.23, 153.75, 149.74, 149.55, 149.03, 148.80, 133.35, 132.98, 131.59, 131.05, 129.58, 129.39, 128.73, 128.50, 124.58, 124.16, 108.63, 108.02, 86.78, 85.62, 83.82, 83.76, 83.25, 81.89, 81.60, 78.86, 78.80, 78.09, 77.64, 63.23, 62.59, 58.52, 58.39, 46.68, 36.01, 35.95, 35.62, 24.92, 24.87, 23.32, 8.26 ppm. ^{31}P NMR (243 MHz, D_2O, triethylammonium salt) δ 4.17, 4.13 ppm. ESI-TOF-MS calcd. for $C_{24}H_{30}N_6O_{12}P$, 625.1664 [M - H]$^-$; found 625.1693.

Guanosine 5'-Diphosphate Imidazolide (S24): To a solution of guanosine-5'-diphosphate triethylammonium salt (1.00 g, 1.55 mmol), imidazole (844 mg, 12.4 mmol), and 2,2'-dithiodipyridine (1.02 g, 4.65 mmol) in *N*,*N*-dimethylformamide (18.0 mL), was added triethylamine (433 µL, 314 mg, 3.10 mmol) followed by triphenylphosphine (1.22 g, 4.65 mmol). After stirring at room temperature for 12 hours, the reaction mixture was added dropwise to a sodium perchlorate solution (550 mg, 4.49 mmol) in acetone (173 mL) under stirring. The resulting precipitate was collected by filtration and washed with acetone. The solid was dried under reduced pressure to afford guanosine 5'-diphosphophate imidazolide disodium salt (**S24**) (673 mg, 80.8%) as yellow solid. ^{1}H NMR (600 MHz, D_2O) δ 7.85 (d, *J* = 0.9 Hz, 1H), 7.78 (s, 1H), 7.17–7.13 (m, 1H), 6.86 (dt, *J* = 2.4, 1.1 Hz, 1H), 5.73 (d, *J* = 6.4 Hz, 1H), 4.59–4.54 (m, 1H), 4.24–4.20 (m, 1H), 4.11 (d, *J* = 3.1 Hz, 1H), 3.94–3.86 (m, 2H), 3.03 (q, *J* = 7.4 Hz, 0.5H), 2.85 (s, 0.5H), 2.70 (q, *J* = 1.2 Hz, 0.5H), 2.55 (s, 0.2H), 2.07 (d, *J* = 1.2 Hz, 1.5H), 1.11 (t, *J* = 7.4 Hz, 1H) ppm. ^{31}P NMR (243 MHz, D_2O) δ -11.28 (d, *J* = 19.7 Hz), -19.38 (d, *J* = 23.0 Hz) ppm.

Di-PureCap/ 3'OMe (3): To a solution of N^7-methyl-2'-Nb-3'-*O*-methyl-guanosine 5'-monophosphate (**S23**) (18.4 mg, 18.6 µmol) in DMSO (372 µL) was added guanosine 5'-diphosphate imidazolide (**S24**) (30.0 mg, 55.8 µmol) and zinc chloride (50.7 mg, 372 µmol). After stirring at 37 ºC for 1.5 days, the reaction mixture was quenched by adding 500 mM EDTA *aq.* (pH 8.0) (968 µL, EDTA: 484 mmol). The mixture was diluted with water (14.0 mL) and purified by reverse-phase HPLC using (instrument, Shimadzu; column, YMC-Actus Triart C8 (Preparative, 250 × 20.0 mm I.D.); Solvent A, 50 mM TEAA buffer (pH 6.0) containing 0.5% CH_3CN; Solvent B, CH_3CN; linear gradient 5–80%B (25 min); flow rate, 10 mL/min;

detection wavelength, 254 nm). The fractions containing the target product were collected, concentrated, and lyophilized to afford the PureCap analog (**3**) as triethylammonium salt. The product was redissolved in methanol (2.00 mL). 190 mM $NaClO_4$ in acetone (12.0 mL) was added to the mixture, and the resulting suspension was centrifuged (4,000 rpm, 20 minutes). The supernatant was discarded, and the precipitate was resuspended in acetone. The suspending-centrifugation processes were repeated additional 4 times. The precipitate was dried under reduced pressure to afford the PureCap Analog (**3**) (14.9 mg, 13.3 μmol, 71.5% yield) as sodium salt. The yield was calculated by using the absorbance of the product at 260 nm measured by a NanoDrop 2000 spectrophotometer. Extinction coefficient (ε_{260}) = 26,300 $M^{-1}\cdot cm^{-1}$ was used for calculation. ^1H NMR (600 MHz, D_2O) δ 7.93 (s, 1H), 7.80 (d, *J* = 8.2 Hz, 1H), 7.68–7.40 (m, 3H), 5.99 (s, 1H), 5.92 (d, *J* = 5.7 Hz, 0.5H), 5.79 (s, 0.5H), 5.72 (d, *J* = 5.9 Hz, 0.5H), 5.43 (s, 0.5H), 4.60 (s, 1H), 4.48–4.41 (m, 1H), 4.39–4.14 (m, 8H), 4.08 (s, 1H), 4.01 (s, 2H), 3.51–3.39 (m, 2H), 0.93–0.77 (m, 9H) ppm. ^{13}C NMR (151 MHz, D_2O) δ 158.67, 154.06, 153.84, 151.39, 149.23, 148.87, 137.33, 133.00, 131.59, 130.94, 129.55, 129.27, 128.77, 124.21, 115.98, 108.52, 107.89, 86.67, 85.49, 83.68, 83.62, 82.51, 81.65, 78.20, 77.97, 73.79, 70.33, 65.53, 64.94, 58.55, 36.05, 35.67, 30.29, 24.80 ppm. ^{31}P NMR (243 MHz, D_2O) δ -10.96 (2P, dt, *J* = 88.8, 23.0 Hz), -22.44 (1P, t, *J* = 19.7 Hz) ppm. ESI-TOF-MS calcd. for $C_{34}H_{43}N_{11}O_{22}P_3$, 1050.1802 $[M - H]^-$; found 1050.1825.

2.6 Synthesis of Di-PureCap/ N2 (4)

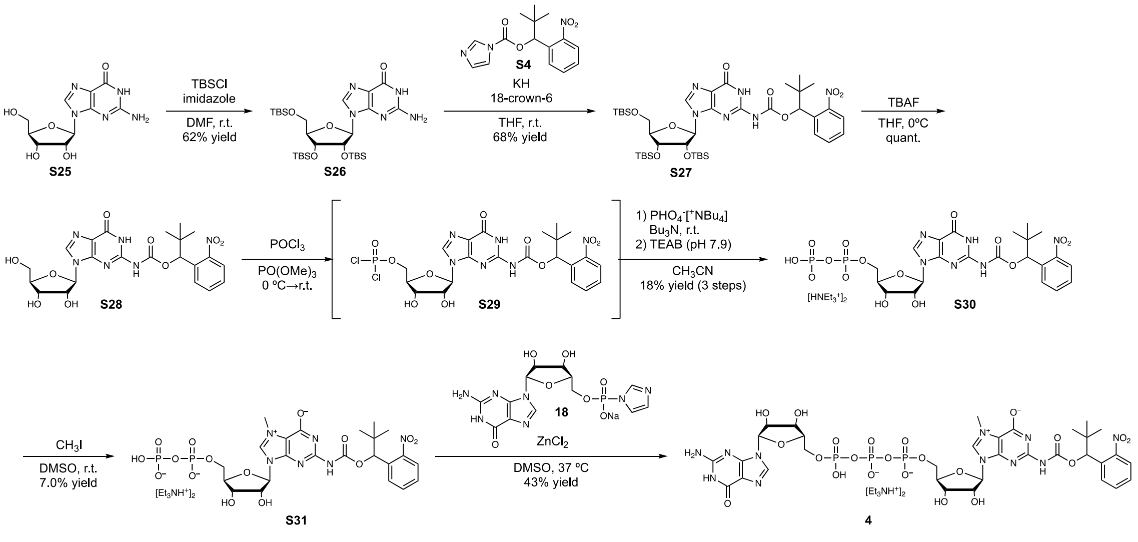

Supplementary Figure 8. Synthesis of Di-PureCap/N2 (**4**).

2',3',5'-*O*-Tris(TBDMS)-guanosine (S26): To a suspension of guanosine (**S25**) (10 g, 35 mmol) and imidazole (19 g, 0.28 mol) in *N*,*N*-dimethylformamide (70 mL), was added *tert*-butyldimethylsilyl chloride (21 g, 0.14 mol). After being stirred at room temperature for 2 days,

the reaction mixture was diluted with ethyl acetate and washed 2 times with an aqueous solution of *sat.* NH_4Cl and 2 times with brine. The organic layer was dried over Na_2SO_4 and concentrated. The residue was purified by silica gel column chromatography eluted by dichloromethane, followed by 1→3% methanol/dichloromethane, to afford compound **S26** (14 g, 62% yield) as white solid. All the spectral data of the product was consistent with the literature[6].

***N*²-Nb-2',3',5'-*O*-Tris(TBDMS)-guanosine (S27)**: To a solution of 2',3',5'-*O*-tris(TBDMS)-guanosine (**S26**) (4.9 g, 7.9 mmol), the carbonylimidazole derivative **S4** (2.7 g, 8.7 mmol), and 18-crown-6 (2.8 g, 11 mmol) in THF (40 mL) was added 30% potassium hydride in mineral oil (1.4 g, 11 mmol). After stirring at room temperature for 5 hours, the reaction mixture was diluted with ethyl acetate and washed with a 1:1 mixture of brine and aqueous *sat.* NH_4Cl. The organic layer was dried over Na_2SO_4 and concentrated. The residue was purified by silica gel column chromatography eluted by 25→50% ethyl acetate/hexane to afford compound **S27** (4.6 g, 68% yield) as brown foam. ^1H NMR (600 MHz, $CDCl_3$) δ 11.17, 11.15 (2s, 1H), 8.22, 8.15 (2s, 1H), 8.00 (d, J = 2.4 Hz, 1H), 7.90 (d, J = 8.2 Hz, 1H), 7.62–7.50 (m, 2H), 7.49–7.42 (m, 1H), 6.53 (d, J = 6.2 Hz, 1H), 5.83 (dd, J = 5.9, 1.6 Hz, 1H), 4.37 (ddd, J = 5.8, 4.3, 1.6 Hz, 1H), 4.22 (dd, J = 4.4, 2.7 Hz, 1H), 4.06–4.03 (m, 1H), 3.87 (dt, J = 11.5, 3.0 Hz, 1H), 3.74 (dd, J = 11.4, 2.5 Hz, 1H), 0.96–0.88 (m, 28H), 0.75 (d, J = 12.9 Hz, 9H), 0.12–0.04 (m, 13H), -0.08 (d, J = 6.9 Hz, 3H), -0.30 (d, J = 14.6 Hz, 3H) ppm. ^{13}C NMR (151 MHz, $CDCl_3$) δ 155.39, 152.80, 149.27, 149.23, 148.54, 146.38, 137.13, 132.65, 132.60, 132.11, 129.16, 128.77, 128.73, 124.90, 120.86, 87.24, 86.22, 79.15, 77.02, 72.57, 62.99, 60.52, 36.32, 26.15, 25.92, 25.77, 25.69, 21.17, 18.59, 18.16, 17.91, 14.27, -4.35, -4.50, -4.58, -5.11, -5.30, -5.38 ppm. ESI-TOF-MS calcd. for $C_{40}H_{68}N_6NaO_9Si_3$, 883.4248 $[M + Na]^+$; found, 883.4555.

***N*²-Nb-guanosine (S28)**: To an ice-cold solution of fully protected-guanosine **S27** (2.0 g, 2.3 mmol) in THF (11 mL), 1 M tetrabutylammonium fluoride/THF (23 mL, 23 mmol) was added. After stirring at 0 ºC for 3 hours in an ice bath, the reaction mixture was diluted with dichloromethane and washed 3 times with water. The organic layer was dried over Na_2SO_4 and concentrated. The residue was purified by silica gel column chromatography eluted by dichloromethane, followed by 1→17% methanol/dichloromethane. The product was suspended in dichloromethane, and the solution was added dropwise to ether under stirring. The resulting precipitate was collected by filtration and rinsed with ether to afford compound **S28** (1.2 g, quant.) as white solid. ^1H NMR (400 MHz, DMSO-d_6) δ 11.65 (s, 1H), 11.13 (s, 1H), 8.20 (s, 1H), 7.96 (dd, J = 8.0, 1.3 Hz, 1H), 7.77 (td, J = 7.6, 1.3 Hz, 1H), 7.65 – 7.52

(m, 2H), 6.23 (s, 1H), 5.75 (d, *J* = 5.7 Hz, 1H), 5.46 (dd, *J* = 5.8, 3.1 Hz, 1H), 5.16 (d, *J* = 4.6 Hz, 1H), 5.01 (t, *J* = 5.4 Hz, 1H), 4.39 (q, *J* = 5.5 Hz, 1H), 4.09 (q, *J* = 4.3 Hz, 1H), 3.86 (q, *J* = 4.0 Hz, 1H), 3.60 (dt, *J* = 12.0, 4.8 Hz, 1H), 3.50 (dt, *J* = 12.0, 4.6 Hz, 1H), 0.88 (s, 9H) ppm. ^{13}C NMR (101 MHz, DMSO-d_6) δ 155.56, 154.36, 149.53, 149.31, 147.68, 138.15, 133.69, 132.07, 130.01, 129.33, 125.10, 120.42, 87.15, 85.83, 77.67, 74.46, 70.72, 61.67, 36.46, 25.83 ppm. ESI-TOF-MS calcd. for $C_{22}H_{26}N_6NaO_9$, 541.1654 [M + Na]$^+$; found, 541.1887.

***N*²-Nb-Guanosine 5'-diphosphate (S30):** To a solution of N^2-Nb-guanosine (**S30**) (400 mg, 0.770 mmol) in trimethyl phosphate (7.00 mL), phosphoryl chloride (83.0 µL, 153 mg, 1.00 mmol) was added. The mixture was gradually warmed to room temperature and stirred at room temperature. After being stirred at room temperature for 4 hours, the reaction mixture containing the phosphorodichloridate intermediate (**S29**) was added to a mixture of 0.5 M tetrabutylammonium dihydrogen phosphate/CH_3CN (4.60 mL, 2.30 mmol) and tributylamine (1.60 mL, 1.30 g, 6.90 mmol) in an ice bath. The reaction mixture was warmed to room temperature and stirred at room temperature for 16 hours. Then, the reaction mixture was quenched by adding 0.2 M TEAB buffer (pH 7.9, 10 mL). After stirring at room temperature for 1–2 hours, the clear solution was diluted with water and washed 5 times with dichloromethane. The aqueous layer was concentrated by a rotary evaporator at 50 ºC. The residue was purified by ion-exchange chromatography on QAE-Sephadex in a linear 0–1.5 M gradient of TEAB buffer containing 0–10% CH_3CN. N^2-Nb-Guanosine 5'-diphosphate (**S30**) was obtained as triethylammonium salt (121 mg, 18% yield) as white solid. ^{1}H NMR (600 MHz, D_2O) δ 8.15 (s, 1H), 7.84 (d, *J* = 8.1 Hz, 1H), 7.60 (dd, *J* = 7.3, 3.6 Hz, 2H), 7.44 (dt, *J* = 8.4, 4.3 Hz, 1H), 6.25 (s, 1H), 5.92 (dd, *J* = 6.1, 2.1 Hz, 1H), 4.73–4.68 (m, 1H), 4.43 (dt, *J* = 6.1, 3.1 Hz, 1H), 4.26–4.22 (m, 1H), 4.09 (q, *J* = 4.0 Hz, 2H), 3.06 (q, *J* = 7.4 Hz, 11H), 1.14 (t, *J* = 7.3 Hz, 17H), 0.82 (s, 9H) ppm. ^{13}C NMR (151 MHz, D_2O) δ 157.47, 154.72, 149.98, 148.55, 147.48, 139.55, 133.45, 133.41, 131.75, 129.33, 129.05, 124.69, 119.49, 87.22, 87.14, 84.11, 84.05, 79.23, 73.94, 73.85, 70.51, 65.17, 58.68, 46.71, 35.78, 24.80, 8.26 ppm. ^{31}P NMR (243 MHz, D_2O) δ -10.25 (1P), -10.69 (1P) ppm. ESI-TOF-MS calcd. for $C_{22}H_{27}N_6O_{15}P_2$, 677.1015 [M - H]$^-$; found, 677.1162.

***N*²-Nb-*N*⁷-Methyl-Guanosine 5'-diphosphate (S31):** To a solution of N^2-Nb-guanosine 5'-diphosphate (**S30**) (72.0 mg, 82.0 µmol) in DMSO (1.82 mL) was added iodomethane (88.0 mg, 39.0 µL, 623 µmol). After being stirred at room temperature for 24 hours, the reaction mixture was diluted with water and washed 5 times with ether. The aqueous phase was concentrated, and the residue was dissolved in water. The crude was purified by reversed-

phase HPLC using YMC-Triart C8 column (250 × 10.0 mm I.D., S-5 µm, 12 nm, flow rate 3 mL/min, temperature 50 ºC) with a linear gradient of 10–80% of CH_3CN in 0.1 M triethylammonium bicarbonate buffer (pH 7.9) in 25 minutes. The collected fractions containing the product were acidified to pH 4.0 by adding a few drops of acetic acid. After the removal of CH_3CN under reduced pressure, the product was desalted by Wakosil®25C18 (15–30 µm, spherical) and lyophilized to afford compound (**S31**) (5.10 mg, 7.0% yield) as a white solid. ^{1}H NMR (600 MHz, D_2O) δ 7.84 (d, J = 8.2 Hz, 1H), 7.60 (s, 2H), 7.44 (d, J = 7.2 Hz, 1H), 6.21 (s, 1H), 6.03 (d, J = 4.9 Hz, 1H), 4.52 (d, J = 15.6 Hz, 1H), 4.37 (s, 1H), 4.27 (s, 1H), 4.19 (d, J = 11.7 Hz, 1H), 4.08 (s, 1H), 4.02 (s, 3H), 3.05 (q, J = 7.1 Hz, 13H), 1.13 (t, J = 7.3 Hz, 20H), 0.81 (s, 9H) ppm. ^{13}C NMR (151 MHz, D_2O) δ 154.19, 148.63, 148.05, 133.34, 131.98, 129.23, 129.00, 124.64, 111.87, 89.95, 84.20, 78.79, 75.14, 69.16, 63.98, 46.69, 36.06, 35.73, 24.83, 8.25 ppm. ^{31}P NMR (243 MHz, D_2O) δ -9.24 (1P), -10.67 (1P) ppm. ESI-TOF-MS calcd. for $C_{23}H_{29}N_6O_{15}P_2$, 691.1172 $[M – H]^-$; found, 691.1395.

Di-PureCap/ N2 (4): N^2-Nb-N^7-Methyl-guanosine 5’-diphosphate (**S31**) (1.30 mg, 1.45 µmol) and guanosine 5’-phosphate imidazolide (**18**) (2.10 mg, 4.79 µmol) were dissolved in 0.59 M zinc chloride DMSO solution (80 µL, $ZnCl_2$: 47.2 µmol). The mixture was incubated for 2 days at 37 ºC. The addition of 38 mM aqueous EDTA (1.6 mL, 60 µmol) quenched the reaction mixture*,* and it was diluted with 0.2 M TEAB buffer (pH 7.9, 600 µL, adjusted the pH around 4.0). The mixture was purified by reversed-phase HPLC using a YMC-Triart C8 column (250 × 4.6 mm I.D., S-5 µm, 12 nm, flow rate 1 mL/min, temperature 50 ºC) with a linear gradient of 5–80% of CH_3CN in 0.1 M triethylammonium bicarbonate buffer (pH 7.9) in 25 minutes. The collected fractions containing the product were acidified to pH 4.0 by adding a few drops of acetic acid. After the removal of CH_3CN under reduced pressure, the product was desalted by Wakosil® 25C18 (15–30 µm, spherical) and lyophilized to afford Di-PureCap/ N2 (**4**) (0.50 mg, 43% yield, triethylammonium salt) as white solid. ^{1}H NMR (600 MHz, D_2O) δ 7.86–7.81 (m, 2H), 7.60 (d, J = 5.4 Hz, 2H), 7.43 (dt, J = 8.5, 4.5 Hz, 1H), 6.23 (d, J = 2.8 Hz, 1H), 5.90 (t, J = 2.9 Hz, 1H), 5.64 (t, J = 7.1 Hz, 1H), 4.57–4.51 (m, 1H), 4.44 (dt, J = 13.6, 4.1 Hz, 1H), 4.31 (tt, J = 9.6, 5.1 Hz, 2H), 4.25–4.20 (m, 2H), 4.18–4.04 (m, 5H), 3.95 (s, 3H), 3.06 (q, J = 7.4 Hz, 16H), 1.13 (t, J = 7.3 Hz, 24H), 0.82 (s, 9H) ppm. ^{13}C NMR (151 MHz, D_2O) δ 158.52, 154.16, 153.87, 151.55, 148.53, 147.63, 137.35, 133.35, 131.77, 129.27, 129.01, 124.60, 115.94, 111.35, 89.88, 87.44, 86.60, 84.06, 83.81, 79.01, 75.14, 73.65, 70.46, 69.16, 65.47, 64.30, 46.67, 36.18, 35.76, 24.82, 8.24 ppm. ^{31}P NMR (243 MHz, D_2O) δ -11.02 (2P), -22.60 (1P) ppm. ESI-TOF-MS calcd. for $C_{33}H_{41}N_{11}O_{22}P_3$, 1036.1646 $[M - H]^-$; found, 1036.1643.

1.7 Synthesis of Trinucleotide/Tetranucleotide PureCap Analogs (5 - 8)

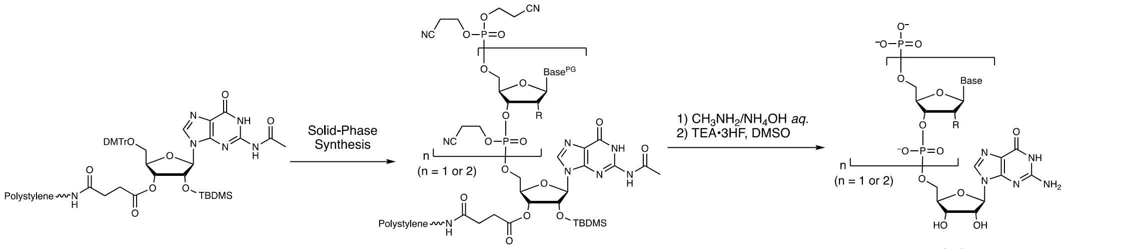

19^{PG}: n = 1, Base^{PG} = N^6-Bz-Ade, R = OTOM
20^{PG}: n = 1, Base^{PG} = N^6-Bz-Ade, R = OCH_3
21^{PG}: n = 2, Base^{PG} = N^6-Bz-Ade and N^2-*i*-Bu-Gua R = OCH_3
22^{PG}: n = 2, Base^{PG} = N^6-CH_3-Bz-Ade and N^2-*i*-Bu-Gua R = OCH_3

19: n = 1, Base = Ade, R = OTOM, 21.5% yield
20: n = 1, Base = Ade, R = OCH_3, 55.0% yield
21: n = 2, Base = Ade and Gua R = OCH_3, 34.0% yield
22: n = 2, Base = N^6-CH_3-Ade and Gua R = OCH_3, 41.0% yield

Supplementary Figure 9. Solid-phase synthesis of dinucleotides and trinucleotides (**19 – 22**).

General Procedure: Syntheses of dinucleotides and trinucleotides were performed by DNA/RNA synthesizer NR-2A_7MX (Nihon Techno Service) on Primer Support 5G riboG 300 (Cytiva, 15.0 µmol scale synthesis) according to the literature[7]. In the coupling steps, 100 mM 2'-*O*-methyl-Adenosine(n-Benzoyl)-CE-phosphoramidite (ChemGenes) in CH_3CN, 100 mM 2'-*O*-methyl-Adenosine(n-Benzoyl)-CE-phosphoramidite in CH_3CN, 100 mM 2'-*O*-methyl-Guanosine(n-*i*-Bu)-CE-phosphoramidite (ChemGenes) in CH_3CN, 50–70 mM 5'-DMTr-2'-TOM-ribo Adenosine (n-acetyl) OP (ChemGenes) in CH_3CN, 150 mM bis(2-cyanoethyl)-*N*,*N*-diisopropylphosphoramidite in CH_3CN, and 0.3 M 5-(benzylthio)-1*H*-tetrazole in CH_3CN (activator) were circulated through the column. A solution of 184 mM trichloroacetic acid/dichloromethane was used as detritylation reagent, and 0.05 M I_2 in pyridine/H_2O (9:1, v/v) for the oxidation, 10% Ac_2O in THF/pyridine (8:1, v/v) as Cap-A, 10% 1-methylimidazole in THF as Cap-B. The synthesis was performed using a 10 µmol scale standard RNA synthesis protocol. After the last cycle of the synthesis, the solid support was treated by a 1:1 mixture of 28% ammonium hydroxide/40% methylamine *aq.* (1.00 mL/15.0 µmol of solid support) at 65 °C for 1 hour to remove the protecting groups and cleave the nucleotides from the solid support. The nucleotides were concentrated to dryness and redissolved in DMSO (1.00 mL/60.0 µmol). To the solution was added TEA•3HF (1.00 mL/60.0 µmol) and incubated at 65 °C for 3–5 hours to remove 2'-*O*-silyl protecting groups. The reaction mixture was neutralized by the addition of 250 mM Na_2CO_3 *aq.* (10.0 mL/60.0 µmol). The crude was purified by ion-exchange chromatography on DEAE-Sephadex (φ = 4.5 cm, *h* = 8.4 cm, 140 cm^3, 12.0 mL/min) in a linear 0–1.2 M gradient of TEAB buffer (pH 7.9) containing 0–8% CH_3CN (270 minutes). The fractions containing the products were collected and concentrated to give the di-/trinucleotides. The yields were calculated by using the absorbance of the product at 260 nm measured by a NanoDrop 2000 spectrophotometer. Extinction coefficients (ε_{260}) = 25,000 M^{-1}•cm^{-1} for dinucleotides, and 35,100 M^{-1}•cm^{-1} for

trinucleotides, were used for the calculation.

pApG (19) (Triethylammonium salt, ε_{260} = 25,000 M^{-1}•cm^{-1}): ^{1}H NMR (400 MHz, D_2O) δ 8.30 (s, 1H), 8.06 (s, 1H), 7.80 (s, 1H), 5.89 (d, J = 4.4 Hz, 1H), 5.70 (d, J = 5.2 Hz, 1H), 4.75–4.70 (m, 2H), 4.57 (q, J = 4.8 Hz, 1H), 4.36 (d, J = 4.7 Hz, 2H), 4.27–4.11 (m, 2H), 4.11–3.94 (m, 3H), 3.42 (q, J = 7.2 Hz, 4H), 3.09 (q, J = 7.4 Hz, 14H), 1.36–0.99 (m, 27H) ppm. ^{31}P NMR (243 MHz, D_2O) δ 0.76, -0.09 ppm. ESI-TOF-MS *m/z* calcd. for $C_{20}H_{25}N_{10}O_{14}P_2$ $[M - H]^-$: 691.1032; found: 691.1071.

pA(2'OCH$_3$)pG (20) (Triethylammonium salt, ε_{260} = 25,000 M^{-1}•cm^{-1}): ^{1}H NMR (600 MHz, D_2O) δ 8.39–8.23 (m, 1H), 8.12–7.94 (m, 1H), 7.89–7.73 (m, 1H), 5.95 (t, J = 4.5 Hz, 1H), 5.69 (t, J = 4.7 Hz, 1H), 4.82–4.75 (m, 1H, overlapped with water), 4.61 (d, J = 5.2 Hz, 1H), 4.41–4.28 (m, 3H), 4.28–4.15 (m, 1H), 4.15–4.02 (m, 2H), 4.02–3.88 (m, 2H), 3.43–3.31 (m, 3H), 3.31–3.18 (m, 3H), 3.06 (tt, J = 11.1, 6.5 Hz, 12H), 1.22–1.08 (m, 22H) ppm. ^{31}P NMR (162 MHz, D_2O) δ 0.87, -0.27 ppm. ^{13}C NMR (151 MHz, D_2O) δ 158.59, 155.26, 153.67, 152.50, 151.40, 148.67, 139.66, 137.60, 118.65, 116.36, 87.70, 85.41, 83.42, 82.79, 82.13, 73.29, 72.47, 70.14, 65.17, 63.84, 58.87, 57.84, 46.71, 8.26, 7.39 ppm. ESI-TOF-MS *m/z* calcd. for $C_{21}H_{27}N_{10}O_{14}P_2$ $[M - H]^-$: 705.1189; found: 705.1250.

pA(2'OCH$_3$)pG(2'OCH$_3$)pG (21) (Triethylammonium salt, ε_{260} = 35,100 M^{-1}•cm^{-1}): ^{1}H NMR (600 MHz, D_2O) δ 8.45–8.34 (m, 1H), 8.21–8.06 (m, 1H), 7.95 (d, J = 13.8 Hz, 1H), 7.80 (d, J = 11.0 Hz, 1H), 6.02–5.91 (m, 1H), 5.82–5.71 (m, 1H), 5.69–5.57 (m, 1H), 4.79 (s, overlapped with water), 4.49 (d, J = 5.8 Hz, 0.4H, overlapped with water), 4.39–4.17 (m, 5H), 4.17–3.89 (m, 7H), 3.56–3.40 (m, 5H), 3.40–3.16 (m, 7H), 3.16–3.00 (m, 5H), 1.31–1.02 (m, 17H) ppm. ^{31}P NMR (243 MHz, D_2O) δ 0.60, -0.25, -0.46 ppm. ESI-TOF-MS *m/z* calcd. for $C_{32}H_{41}N_{15}O_{21}P_3$ $[M - H]^-$: 1064.1819; found: 1064.1941.

p^{m6}A(2'OCH$_3$)pG(2'OCH$_3$)pG (22) (Triethylammonium salt, ε_{260} = 35,100 M^{-1}•cm^{-1}): ^{1}H NMR (600 MHz, D_2O) δ 8.37 (d, J = 1.9 Hz, 1H), 8.04 (s, 1H), 7.93 (d, J = 2.0 Hz, 1H), 7.82 (d, J = 1.9 Hz, 1H), 6.05 (d, J = 5.0 Hz, 1H), 5.80 (dd, J = 6.0, 2.0 Hz, 1H), 5.76 (d, J = 5.1 Hz, 1H), 4.88 (p, J = 4.5 Hz, 3H), 4.50 (t, J = 5.3 Hz, 1H), 4.46 – 4.40 (m, 4H), 4.31 (s, 1H), 4.18 – 4.13 (m, 4H), 4.05 – 4.00 (m, 2H), 3.49 – 3.37 (m, 6H), 3.18 – 3.14 (m, 24H), 3.03 (d, J = 7.2 Hz, 3H), 1.24 (td, J = 7.5, 2.1 Hz, 36H) ppm. ^{13}C NMR (151 MHz, D_2O) δ 158.50, 158.41, 154.77, 153.59, 153.52, 152.62, 151.45, 151.21, 138.94, 137.61, 137.38, 116.34, 116.29, 87.43, 86.12, 85.37, 83.55, 83.05, 82.53, 82.13, 80.74, 73.26, 72.57, 72.44, 70.31, 65.05, 64.85, 63.70, 58.99, 57.97, 57.74, 46.68, 8.25, 7.49 ppm. ^{31}P NMR (243 MHz, D_2O) δ 1.65 (1P), -0.29 (1P), -0.42 (1P) ppm. ESI-TOF-MS *m/z* calcd. for $C_{33}H_{42}N_{15}NaO_{21}P_3$ $[M - 2H + Na]^-$: 1100.1795; found: 1100.1149.

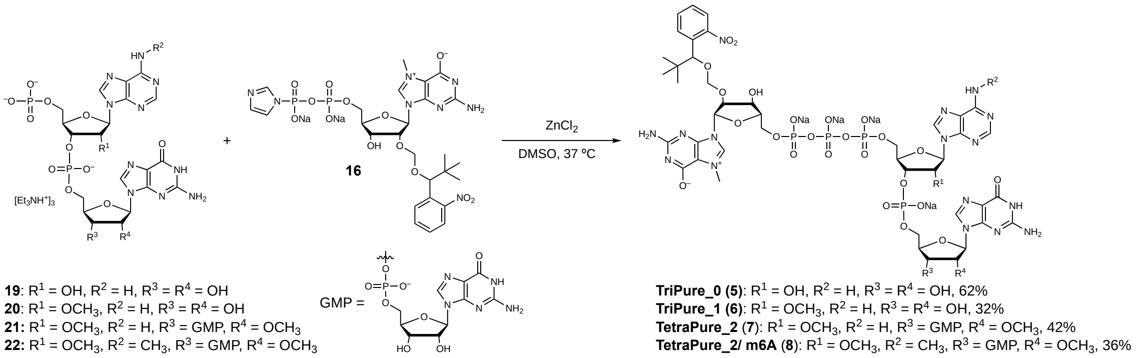

Supplementary Figure 10. Synthesis of Trinucleotide and Tetranucleotide PureCap Analogs (**5 - 8**).

General Procedure for the Synthesis of Trinucleotide and Tetranucleotide PureCap Analogs: To a solution of the dinucleotides or trinucleotides triethylammonium salt (c.a.106 μmol) in DMSO (50 mM), N^7-methyl-2'-Nb-guanosine-5'-diphosphate imidazolide disodium salt (**16**) (3.00 eq.) and zinc chloride (20.0 eq.) were added. After stirring at 37 °C for 3 days, the reaction mixture was quenched by adding 500 mM EDTA *aq.* (pH 8.0) (EDTA: 1.30 eq. per molar amount of $ZnCl_2$). The mixture was diluted with 5.24 volumes of water and purified by reverse-phase HPLC (instrument, Shimadzu; column, YMC-Actus Triart C8 (Preparative, 250 × 20.0 mm I.D.); Solvent A, 50 mM TEAA buffer (pH 6.0) containing 0.5% CH_3CN; Solvent B, CH_3CN; linear gradient 5–80%B (25 min); flow rate, 10 mL/min; detection wavelength, 254 nm). The fractions containing the target compound were collected, concentrated, and lyophilized to afford the target tri-/tetra-nucleotide PureCap analogs as triethylammonium salt. The product was redissolved in methanol (2.00 mL). 190 mM $NaClO_4$ in acetone (24.0 mL) was added to the mixture, and the resulting suspension was centrifuged (4,000 rpm, 20 minutes). The supernatant was discarded, and the precipitate was resuspended in acetone. The suspending-centrifugation processes were repeated additional 3–4 times. The precipitate was dried under reduced pressure to afford the target tri-/tetra-nucleotide PureCap analogs as the sodium salt. The yields were calculated by using the absorbance of the product at 260 nm measured by a NanoDrop 2000 spectrophotometer. Extinction coefficients (ε_{260}) = 40,100 M^{-1}•cm^{-1} for trinucleotide PureCap analogs, and 50,200 M^{-1}•cm^{-1} for tetranucleotide PureCap analogs, were used for the calculation.

TriPure_0, Nb-^{m7}GpppApG (5) (Sodium salt, ε_{260} = 40,100 M^{-1}•cm^{-1}): ^{1}H NMR (600 MHz, D_2O) δ 8.36 (s, 1H), 8.14–7.85 (m, 2H), 7.62–7.36 (m, 4H), 7.22 (s, 1H), 5.98–5.87 (m, 1.5H), 5.77 (s, 1H), 5.71 (s, 0.5H), 5.04–4.83 (m, 3H), 4.71 (s, 3H), 4.57 (s, 1H), 4.49 (d, *J* = 14.2 Hz, 3H), 4.39–4.21 (m, 10H), 4.17 (s, 1H), 4.10 (t, *J* = 7.0 Hz, 1H), 4.05–3.96 (m, 3H), 0.49 (s, 9H) ppm. ^{13}C NMR (151 MHz, D_2O) δ 158.30, 155.43, 155.24, 154.54, 154.35, 153.63,

151.85, 151.26, 149.67, 149.25, 149.06, 148.85, 148.72, 148.50, 139.66, 137.26, 133.59, 132.70, 132.51, 132.21, 129.97, 129.80, 128.71, 128.09, 123.75, 123.34, 118.19, 116.01, 107.57, 107.29, 94.61, 93.70, 87.76, 87.40, 87.10, 86.99, 85.08, 84.43, 83.41, 82.79, 81.24, 80.16, 79.56, 79.10, 74.53, 73.82, 70.19, 68.48, 68.13, 65.21, 64.98, 64.71, 64.37, 61.76, 48.96, 36.24, 36.20, 36.15, 35.68, 30.31, 25.10, 24.95, 24.84, 20.58, 13.32, 8.30 ppm. ^{31}P NMR (243 MHz, D_2O) *δ* 0.07 (1P), -10.70 (2P), -21.98 (1P) ppm. ESI-TOF-MS *m/z* calcd. for $C_{43}H_{54}N_{16}O_{27}P_4$ [M - 2H]$^{2-}$: 675.1153; found: 675.1165.

TriPure_1, Nb-^{m7}GpppA(2'OCH$_3$)pG (6) (Sodium salt, ε_{260} = 40,100 M^{-1}•cm^{-1}): ^{1}H NMR (600 MHz, D_2O) *δ* 8.51–8.28 (m, 1H), 8.17–7.88 (m, 2H), 7.63 (s, 0.5H), 7.54 (d, *J* = 7.1 Hz, 1H), 7.44 (s, 1.5H), 7.37 (s, 0.5H), 7.28 (s, 0.5H), 5.98 (dd, *J* = 17.3, 6.4 Hz, 1H), 5.88 (s, 1H), 5.79 (d, *J* = 6.6 Hz, 1H), 5.70 (s, 1H), 5.00–4.87 (m, 3H), 4.72 (s, 1H), 4.61–4.55 (m, 0.5H), 4.49 (s, 2.5H), 4.42–4.14 (m, 10H), 4.11 (dt, *J* = 15.6, 7.5 Hz, 1H), 4.08–3.94 (m, 3H), 3.39 (d, *J* = 10.7 Hz, 3H), 0.72–0.46 (2s, 9H) ppm. ^{13}C NMR (151 MHz, D_2O) *δ* 158.52, 155.75, 155.19, 154.97, 153.66, 152.74, 149.69, 149.27, 148.83, 148.62, 139.49, 133.63, 132.61, 132.25, 129.99, 129.86, 128.77, 128.08, 123.78, 123.35, 118.27, 116.26, 107.62, 107.30, 94.76, 93.54, 87.62, 87.37, 85.31, 85.03, 84.92, 84.67, 83.59, 83.53, 83.13, 82.16, 82.03, 81.66, 80.38, 79.49, 79.16, 73.53, 72.84, 70.32, 68.60, 68.24, 65.11, 64.84, 64.46, 61.77, 57.97, 36.31, 36.15, 36.12, 35.67, 35.54, 30.29, 24.87, 24.82, 24.74, 24.66, 20.56, 13.29 ppm. ^{31}P NMR (243 MHz, D_2O) *δ* -0.23 (1P), -10.81 (2P), -22.01 (1P) ppm. ESI-TOF-MS *m/z* calcd. for $C_{44}H_{56}N_{16}O_{27}P_4$ [M - 2H]$^{2-}$: 682.1231; found: 682.1225.

TetraPure_2, Nb-^{m7}GpppA(2'OCH$_3$)pG(2'OCH$_3$)pG (7) (Sodium salt, ε_{260} = 50,200 M^{-1}•cm^{-1}): ^{1}H NMR (600 MHz, D_2O) *δ* 8.42–8.34 (m, 1H), 8.06 (d, *J* = 12.4 Hz, 1.5H), 7.97 (s, 1H), 7.84 (s, 1H), 7.67 (s, 0.5H), 7.57 (d, *J* = 7.6 Hz, 1H), 7.51–7.36 (m, 2H), 7.29 (s, 1H), 6.88 (s, 1H), 5.94 (dd, *J* = 11.6, 5.1 Hz, 2H), 5.88–5.80 (m, 1.5H), 5.73 (s, 1H), 5.68 (d, *J* = 4.3 Hz, 0.5H), 4.89 (s, 4H), 4.58 (s, 2.5H), 4.52 (s, 0.5H), 4.46 (s, 3H), 4.38 (s, 3H), 4.34–4.24 (m, 8H), 4.21–4.08 (m, 11H), 4.05 (s, 1.5H), 4.00 (s, 1.5H), 3.83–3.47 (m, 6H), 0.64–0.46 (m, 9H) ppm. ^{13}C NMR (151 MHz, D_2O) *δ* 159.70–158.2 (m), 153.71–150.8 (m), 149.70–148.00 (m), 132.07, 129.36, 128.17, 123.14, 117.26, 114.91, 107.66, 87.66, 84.99, 82.62, 80.56, 79.37, 75.68, 72.44, 68.13, 65.01, 61.77, 58.27, 57.91, 56.82, 48.94, 46.69, 36.13, 35.36, 30.30, 28.25, 24.76, 20.57, 16.84, 13.30, 8.32 ppm. ^{31}P NMR (243 MHz, D_2O) *δ* -0.26 (2P), -10.84 (2P), -22.09 (1P) ppm. ESI-TOF-MS *m/z* calcd. for $C_{55}H_{70}N_{21}O_{34}P_5$ [M - 2H]$^{2-}$: 861.6546; found: 861.6557.

TetraPure_2/m6A, Nb-^{m7}Gppp^{m6}A(2'OCH$_3$)pG(2'OCH$_3$)pG (8) (Sodium salt, ε_{260} = 50,200 M^{-1}•cm^{-1}): ^{1}H NMR (600 MHz, D_2O) *δ* 8.36 (s, 0.5H), 8.32 (s, 0.5H), 8.09 (s, 0.5H), 8.06 (s, 0.5H), 8.01 (d, *J* = 2.4 Hz, 1H), 7.88 (s, 1H), 7.73 (d, *J* = 8.1 Hz, 0.5H), 7.63 (t, *J* = 7.4 Hz, 0.5H), 7.59 (d, *J* = 8.1 Hz, 0.5H), 7.54 – 7.45 (m, 2.5H), 7.32 (t, *J* = 7.4 Hz, 1H), 6.00 (d, *J* =

5.6 Hz, 0.5H), 5.97 (d, *J* = 5.3 Hz, 0.5H), 5.91 (d, *J* = 5.1 Hz, 0.5H), 5.86 (d, *J* = 6.0 Hz, 1H), 5.79 (t, *J* = 5.2 Hz, 1H), 5.71 (d, *J* = 5.1 Hz, 0.5H), 5.04 (s, 0.5H), 4.96 (d, *J* = 7.4 Hz, 0.5H), 4.92 – 4.90 (m, 2H), 4.62 (d, *J* = 5.2 Hz, 1H), 4.55 (d, *J* = 4.7 Hz, 1H), 4.50 (s, 1H), 4.48 (s, 1.5H), 4.44 (d, *J* = 3.7 Hz, 1.5H), 4.39 (t, *J* = 5.1 Hz, 2.5H), 4.33 (s, 1.5H), 4.29 (d, *J* = 9.1 Hz, 4H), 4.22 (s, 3H), 4.18 (s, 3H), 4.08 (s, 1.5H), 4.03 (s, 1.5H), 3.40 (dd, *J* = 9.2, 2.7 Hz, 6H), 2.71 (d, *J* = 2.3 Hz, 3H), 0.65 (s, 5H), 0.58 (s, 4H) ppm. ^{13}C NMR (151 MHz, D_2O) δ 158.55, 154.70, 153.74, 151.32, 149.00, 138.73, 137.52, 133.75, 132.22, 129.94, 123.46, 116.34, 107.90, 95.04, 93.46, 87.19, 84.99, 80.36, 73.40, 72.49, 70.41, 68.94, 65.07, 57.76, 46.69, 38.91, 38.49, 38.44, 38.35, 38.21, 38.06, 37.93, 37.78, 37.64, 36.45, 36.02, 24.81, 23.54, 8.39 ppm. ^{31}P NMR (243 MHz, D_2O) δ -0.31 (d, *J* = 19.7 Hz, 2P), -10.81 (d, *J* = 19.7 Hz, 2P), -21.89 (1P) ppm. ESI-TOF-MS *m/z* calcd. for $C_{56}H_{72}N_{21}O_{34}P_5$ $[M - 2H]^{2-}$: 868.6645; found: 868.6625.

2.8 Synthesis of Trinucleotide/Tetranucleotide Cap Analogs with No Tag (9, 10)

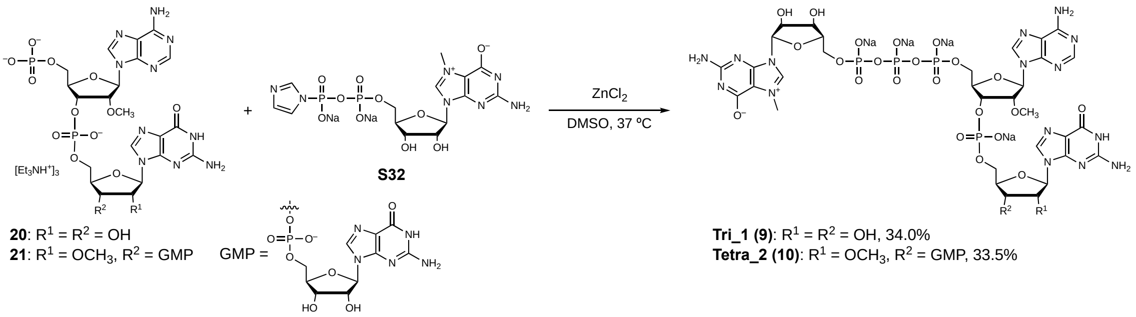

Supplementary Figure 11. Synthesis of Trinucleotide and Tetranucleotide Cap Analogs (**9**, **10**).

General Procedure for the Synthesis of Trinucleotide and Tetranucleotide Cap Analogs (with No Tag): To a solution of the dinucleotides or trinucleotides triethylammonium salt (43.4–62.4 μmol) in DMSO (50 mM), *N*^{7}-methylguanosine-5'-diphosphate imidazolide disodium salt (**S32**) (3.00 eq.) and zinc chloride (20.0 eq.) were added. After stirring at 37 ºC for 3 days, the reaction mixture was quenched by adding 500 mM EDTA *aq.* (pH 8.0) (EDTA: 1.30 eq. per molar amount of $ZnCl_2$). The crude was purified by ion-exchange chromatography on QAE-Sephadex (φ = 3.0 cm, *h* = 10 cm, 70 cm^3, 6 mL/min) in a linear 0–1.0 M gradient of TEAA buffer (pH 6.0) containing 0–10% CH_3CN (270 minutes). The fractions containing the product were collected and lyophilized to afford the target tri-/tetranucleotide cap analogs as triethylammonium salt. The product was redissolved in methanol (2.0 mL). 190 mM $NaClO_4$ in acetone (12 mL) was added to the mixture, and the resulting suspension was centrifuged (4,500 rpm, 20 minutes). The supernatant was

discarded, and the precipitate was resuspended in acetone. The suspending-centrifugation processes were repeated additional 3–4 times. The precipitate was dried under reduced pressure to afford the target tri-/tetranucleotide cap analogs as sodium salt. The yields were calculated by using the absorbance of the product at 260 nm measured by a NanoDrop 2000 spectrophotometer. Extinction coefficients (ε_{260}) = 36,400 $M^{-1}\cdot cm^{-1}$ for trinucleotide cap analogs and 46,500 $M^{-1}\cdot cm^{-1}$ for tetranucleotide cap analogs were used for the calculation.

Tri_1, ^{m7}GpppA(2'OCH$_3$)pG (9) (Sodium salt, ε_{260} = 36,400 $M^{-1}\cdot cm^{-1}$): ^{1}H NMR (600 MHz, D_2O) δ 8.19 (s, 1H), 7.89 (s, 1H), 7.78 (s, 1H), 5.84 (d, J = 5.5 Hz, 1H), 5.70 (d, J = 3.5 Hz, 1H), 5.65 (d, J = 5.4 Hz, 1H), 4.77 (dt, J = 8.3, 4.3 Hz, 1.5H), 4.56 (t, J = 5.3 Hz, 1.5H), 4.53–4.48 (m, 0.5H), 4.46 (t, J = 4.3 Hz, 0.5H), 4.36 (dq, J = 11.4, 3.9 Hz, 4H), 4.29 (t, J = 5.3 Hz, 1H), 4.28–4.22 (m, 4H), 4.19 (dq, J = 4.8, 2.5 Hz, 2H), 4.15 (ddt, J = 8.8, 5.7, 3.2 Hz, 2H), 4.12–4.02 (m, 5H), 3.98–3.92 (m, 3H), 3.86 (s, 3H), 3.31 (s, 3H) ppm. ^{13}C NMR (151 MHz, D_2O) δ 158.44, 157.57, 157.27, 155.16, 153.65, 152.77, 151.31, 149.09, 148.45, 139.34, 137.39, 118.23, 116.08, 108.28, 107.98, 89.39, 89.29, 87.41, 85.02, 84.05, 83.77, 83.49, 82.81, 81.94, 74.86, 73.56, 72.52, 70.20, 69.29, 69.14, 65.11, 64.40, 57.99, 48.95, 36.03, 30.30, 23.33 ppm. ^{31}P NMR (243 MHz, D_2O) δ -0.30 (1P), -10.71 (2P), -21.99 (1P). ESI-TOF-MS calcd. for $C_{32}H_{41}N_{15}O_{24}P_4$, 571.5705 $[M - 2H]^{2-}$; found 571.5679, calcd. for $C_{32}H_{40}N_{15}NaO_{24}P_4$, 582.5615 $[M + Na - 3H]^{3-}$; found 582.5588.

Tetra_2, ^{m7}GpppA(2'OCH$_3$)pG(2'OCH$_3$)pG (10) (Sodium salt, ε_{260} = 46,500 $M^{-1}\cdot cm^{-1}$): ^{1}H NMR (600 MHz, D_2O) δ 11.41 (br), 8.73–8.21 (m, 2H), 7.92 (s, 1H), 6.78 (s, 1H), 5.98–5.72 (m, 2H), 5.05 (s, 1H), 4.48 (s, 1H), 4.40–3.82 (m, 14H), 3.68–3.51 (m, 4H), 3.46 (s, 2H) ppm. ^{13}C NMR (151 MHz, D_2O) δ 159.13, 154.60, 153.72, 152.51, 150.98, 149.69, 148.57, 133.54, 132.04, 117.23, 115.57, 114.88, 107.60, 89.31, 87.44, 85.13, 83.09, 80.87, 79.23, 75.86, 74.86, 74.48, 72.60, 69.66, 68.45, 67.91, 65.97, 63.93, 62.63, 58.36, 56.82, 48.95, 46.71, 35.93, 30.31, 23.33, 8.31 ppm. ^{31}P NMR (243 MHz, D_2O) δ -0.14 (2P), -10.68 (2P), -22.04 (1P) ppm. ESI-TOF-MS calcd. for $C_{43}H_{54}N_{20}O_{31}P_5$, 500.3989 $[M - 3H]^{3-}$; found 500.3974, calcd. for $C_{43}H_{55}N_{20}O_{31}P_5$, 751.1020 $[M - 2H]^{2-}$; found 751.1009.

2.9 Synthesis of More Hydrophobic 2'-Nb-Di-PureCap Analogs (23–25)

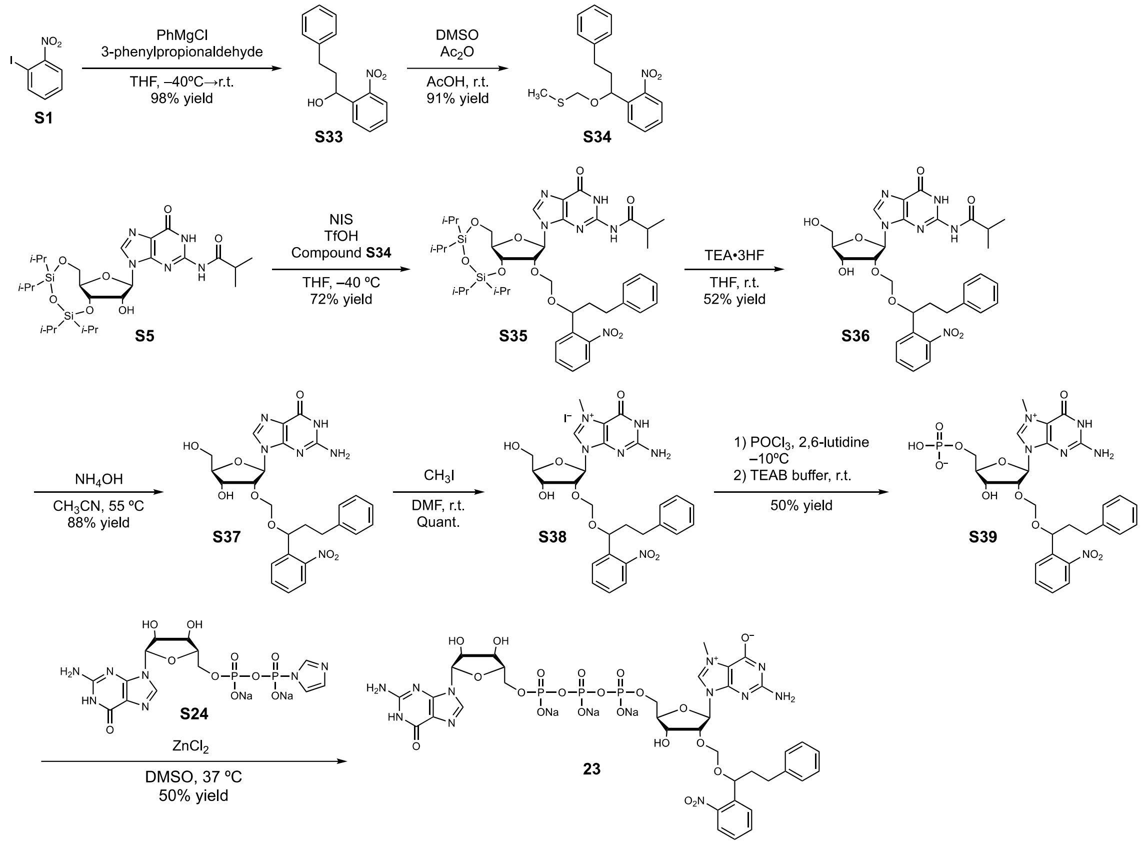

Supplementary Figure 12. Synthesis of 2'-Nb-Di-PureCap Analog with Phenylethyl Group (**23**)

<u>(Phenylethyl)nitrobenzyl Alcohol (S33):</u> A mixture of 1-iodo-2-nitrobenzene (**S1**) (5.00 g, 20.1 mmol) in THF (41.9 mL) was cooled to –40 °C. A solution of phenylmagnesium chloride in THF (2 M, 13.1 mL, 26.1 mmol) was added to the mixture and stirred at –40 °C for 1.5 hours. 3-Phenylpropionaldehyde (3.77 g, 3.70 mL, 28.1 mmol) was added to the reaction mixture and stirred at –40 °C for 2.5 hours. The reaction mixture was quenched by the addition of aqueous solution of *sat.* NH_4Cl (20.0 mL). The mixture was extracted 3 times with dichloromethane (20.0 mL). The organic layer was dried over Na_2SO_4 and concentrated. The residue was purified by silica gel column chromatography eluted by 9.1→50% ethyl acetate/hexane, to afford compound **S33** (5.08 g, 98.3% yield) as brown liquid. ^1H NMR (600 MHz, $CDCl_3$) δ 7.90 (dd, J = 8.2, 1.3 Hz, 1H), 7.82 (dd, J = 7.9, 1.5 Hz, 1H), 7.65 – 7.60 (m, 1H), 7.40 (ddd, J = 8.2, 7.3, 1.4 Hz, 1H), 7.32 – 7.29 (m, 2H), 7.25 – 7.19 (m, 3H), 5.26 (dt, J = 8.8, 3.7 Hz, 1H), 2.93 (ddd, J = 13.6, 10.4, 5.1 Hz, 1H), 2.87 – 2.84 (m, 1H), 2.80 (ddd, J = 13.7, 10.3, 6.3 Hz, 1H), 2.15 (dddd, J = 13.9, 10.1, 6.4, 3.4 Hz, 1H), 2.05 (dddd, J = 13.9, 10.2, 8.8, 5.2 Hz, 1H) ppm. ^{13}C NMR (151 MHz, $CDCl_3$) δ 147.71, 141.44, 140.20, 133.55,

128.48, 128.46, 128.12, 128.07, 126.01, 124.37, 68.95, 39.81, 32.56 ppm. ESI-TOF-MS $C_{15}H_{15}NNaO_3$ (M + Na)$^+$ calcd. *m/z* 280.0944, found *m/z* 280.0845.

(Phenylethyl)nitrobenzyl alcohol methylthioacetal Derivative (S34): To a solution of phenylethyl-nitrobenzyl alcohol (**S33**) (5.08 g, 19.7 mmol) in acetic acid (47.3 g, 45.1 mL, 788 mmol) was added DMSO (30.8 g, 28.0 mL, 394 mmol) and acetic anhydride (40.2 g, 37.2 mL, 394 mmol). After being stirred at room temperature for 2 days, the reaction mixture was added dropwise to 10 M KOH *aq.* (177 mL, 1.77 mol) in an ice-bath. The mixture was stirred at room temperature for 1 hour and then extracted with ethyl acetate (200 mL). The organic layer was washed with brine and dried over Na_2SO_4. The residue was dissolved in hexane and subjected to silica gel column chromatography eluted by 4.8–17% ethyl acetate/hexane, to afford the methylthioacetal **S34** (5.67 g, 90.7% yield) as brown oil. ^{1}H NMR (600 MHz, $CDCl_3$) δ 7.93 (dd, *J* = 8.2, 1.3 Hz, 1H), 7.77 (dd, *J* = 7.9, 1.5 Hz, 1H), 7.64 (td, *J* = 7.5, 1.4 Hz, 1H), 7.42 (ddd, *J* = 8.7, 7.3, 1.5 Hz, 1H), 7.31 – 7.27 (m, 2H), 7.26 – 7.23 (m, 2H), 7.21 – 7.17 (m, 1H), 5.35 (dd, *J* = 9.1, 3.1 Hz, 1H), 4.66 (d, *J* = 11.6 Hz, 1H), 4.31 (d, *J* = 11.6 Hz, 1H), 2.98 (ddd, *J* = 13.7, 11.2, 4.9 Hz, 1H), 2.83 (ddd, *J* = 13.7, 10.8, 5.8 Hz, 1H), 2.21 – 2.18 (m, 0.5H), 2.17 (s, 3H), 2.16 – 2.14 (m, 0.5H), 2.07 (dddd, *J* = 13.9, 10.9, 9.0, 4.9 Hz, 1H) ppm. ^{13}C NMR (151 MHz, $CDCl_3$) δ 148.63, 141.57, 138.05, 133.47, 128.42, 128.39, 128.30, 128.28, 125.93, 124.46, 74.01, 73.72, 68.15, 39.67, 32.66, 14.38 ppm. ESI-TOF-MS $C_{17}H_{19}NNaO_3S$ (M + Na)$^+$ calcd. *m/z* 340.0978, found *m/z* 340.0876.

***N*²-Isobutyryl-3’,5’-*O*-TIPDS-2’-(phenylethyl)Nb-guanosine (S35):** To a suspension of *N*^{2}-Isobutyryl-3’,5’-*O*-(1,1,3,3-tetraisopropyldisiloxane-1,3-diyl) guanosine (**S5**) (2.00 g, 3.35 mmol), nitrobenzyl alcohol methylthioacetal (**S34**) (1.38 g, 4.36 mmol), and powdery molecular sieves 3Å (1.50 g), in tetrahydrofuran (33.5 mL), was added *N*-iodosuccinimide (981 mg, 4.36 mmol) and then cooled to –40 ºC. To the cooled suspension was added trifluoromethanesulfonic acid (755 mg, 447 µL, 5.03 mmol) and stirred at –40ºC for 12 hours. The reaction mixture was quenched by the addition of triethylamine (16.8 mL) and then diluted with ethyl acetate (100 mL). The mixture was washed with saturated *aq.* $NaHCO_3$ (100 mL x1), saturated *aq.* sodium thiosulfate (100 mL x1), and brine (100 mL x1), successively. The organic layer was dried over Na_2SO_4 and concentrated. The residue was purified by silica gel column chromatography eluted by 0–4.8% methanol/dichloromethane, to afford compound **S35** (2.10 g, 72.4% yield) as brown foam. ^{1}H NMR (600 MHz, $CDCl_3$, not pure) δ 12.39 – 12.07 (m, 1H), 10.77 (s, 0.7H), 9.55 – 9.28 (m, 0.5H), 8.02 (s, 0.3H), 7.93 (s, 1H), 7.88 (s, 0.3H), 7.76 – 7.60 (m, 1H), 7.47 – 7.41 (m, 0.5H), 7.30 – 7.25 (m, 0.4H), 7.19 (t, *J* = 7.5 Hz, 1H), 7.16 – 7.12 (m, 1H), 7.09 (td, *J* = 7.3, 1.6 Hz, 1.2H), 7.05 – 7.00 (m, 0.3H),

6.94 – 6.90 (m, 0.5H), 5.84 – 5.71 (m, 1H), 5.65 (s, 0.3H), 5.38 (dd, *J* = 9.1, 3.0 Hz, 0.3H), 5.28 (dd, *J* = 8.3, 3.7 Hz, 0.3H), 5.24 (s, 0.1H), 5.20 (d, *J* = 6.6 Hz, 0.3H), 5.11 (d, *J* = 6.5 Hz, 0.3H), 4.63 (d, *J* = 6.7 Hz, 0.6H), 4.53 (dd, *J* = 9.4, 4.2 Hz, 0.3H), 4.47 – 4.37 (m, 1H), 4.30 – 4.22 (m, 1H), 4.22 – 4.06 (m, 2.5H), 4.06 – 3.90 (m, 2.5H), 2.92 (p, *J* = 6.9 Hz, 1H), 2.84 – 2.68 (m, 1.4H), 2.65 – 2.47 (m, 0.6H), 2.19 – 1.96 (m, 1.4H), 1.22 – 1.19 (m, 6H), 1.06 – 0.91 (m, 35H) ppm. ^{13}C NMR (151 MHz, $CDCl_3$, not pure) *δ* 179.97, 179.46, 179.03, 156.01, 155.76, 155.67, 148.36, 148.29, 147.96, 147.90, 147.82, 147.50, 147.40, 141.30, 140.98, 138.09, 137.50, 136.55, 136.39, 136.33, 133.96, 133.48, 128.57, 128.52, 128.42, 128.38, 128.30, 128.27, 128.22, 127.91, 126.00, 125.85, 124.59, 124.16, 121.37, 121.32, 121.18, 93.73, 91.29, 88.97, 87.94, 87.35, 81.59, 81.17, 81.00, 79.63, 76.76, 75.24, 74.09, 72.08, 70.56, 70.02, 69.49, 60.52, 59.49, 59.42, 39.52, 39.39, 36.35, 36.18, 36.00, 32.22, 31.98, 19.16, 19.02, 18.90, 18.81, 17.43, 17.40, 17.34, 17.27, 17.20, 17.13, 17.05, 17.00, 16.98, 16.92, 16.90, 16.84, 16.79, 13.44, 13.31, 13.20, 12.96, 12.92, 12.84, 12.57, 12.46 ppm. ESI-TOF-MS $C_{42}H_{60}N_6NaO_{10}Si_2$ (M + Na)$^+$ calcd. *m/z* 887.3802, found *m/z* 887.3832.

***N*^{2}-Isobutyryl-2'-(phenylethyl)Nb-guanosine (S36):** To a solution of the protected guanosine **S35** (2.10 g, 2.43 mmol) in THF (9.95 mL) was added triethylamine trihydrofluoride (1.97 g, 1.99 mL, 12.2 mmol). After being stirred at room temperature for 14 hours, the reaction mixture was concentrated by using rotary evaporator. The residue was diluted with dichloromethane (100 mL) and washed with saturated *aq.* $NaHCO_3$ (100 mL). The aqueous layer was extracted with dichloromethane (100 mL x2). The organic layer was dried over Na_2SO_4 and concentrated. The residue was purified by silica gel column chromatography eluted by 2.4–9.1% methanol/dichloromethane, to afford compound **S36** (780 mg, 52.0% yield) as white foam. ^{1}H NMR (600 MHz, $CDCl_3$) *δ* 12.43 – 12.18 (m, 1H), 10.25 – 9.95 (m, 1H), 8.37 (s, 0.5H), 8.16 (s, 0.5H), 7.79 (d, *J* = 7.3 Hz, 0.5H), 7.73 (d, *J* = 7.4 Hz, 0.5H), 7.55 (dq, *J* = 16.0, 7.9 Hz, 1.5H), 7.42 (t, *J* = 7.8 Hz, 0.5H), 7.33 (t, *J* = 8.2 Hz, 0.5H), 7.27 (t, *J* = 8.3 Hz, 0.5H), 7.17 – 7.03 (m, 4H), 6.95 (d, *J* = 7.2 Hz, 1H), 6.06 (d, *J* = 4.8 Hz, 0.5H), 5.89 – 5.85 (m, 0.5H), 5.26 – 5.17 (m, 1H), 5.12 – 5.02 (m, 1H), 4.91 – 4.75 (m, 2H), 4.74 – 4.61 (m, 2H), 4.56 (s, 0.5H), 4.25 – 4.14 (m, 1H), 3.99 – 3.73 (m, 2H), 3.43 (s, 0.5H), 2.92 – 2.76 (m, 1H), 2.74 – 2.51 (m, 1.5H), 2.21 (d, *J* = 13.1 Hz, 0.5H), 2.08 – 1.78 (m, 2H), 1.22 (dt, *J* = 9.8, 6.3 Hz, 6H) ppm. ^{13}C NMR (151 MHz, $CDCl_3$) *δ* 180.14, 179.82, 155.74, 155.66, 148.28, 148.17, 148.09, 148.02, 147.49, 141.19, 141.06, 139.28, 139.02, 137.77, 137.46, 133.63, 133.39, 128.39, 128.35, 128.28, 128.06, 125.98, 125.94, 124.36, 124.21, 121.08, 120.99, 94.30, 93.02, 87.99, 87.82, 86.22, 86.08, 79.99, 79.39, 74.86, 74.00, 70.48, 70.20, 61.83, 53.54, 39.48, 39.29, 36.15, 36.11, 32.21, 31.95, 30.98, 19.07, 19.01, 18.94 ppm. ESI-TOF-MS $C_{30}H_{34}N_6NaO_9$ (M + Na)$^+$ calcd. *m/z* 645.2280, found *m/z* 645.2270.

2’-(Phenylethyl)Nb-guanosine (S37): To a solution of the protected guanosine **S36** (780 mg, 1.25 mmol) in acetonitrile (8.20 mL) was added 28% ammonium hydroxide solution (16.4 mL). The mixture was heated to 55 ºC. After being stirred at 55 ºC for 11 hours, the reaction mixture was concentrated and azeotroped with benzene (x1). The residue was dissolved in dichloromethane (20.0 mL) and suspended by the addition of diethyl ether (200 mL). The resulting precipitate was collected by filtration and rinsed with diethyl ether. The obtained solid was dried under vacuum to afford compound **S37** (611 mg, 88.4% yield) as white powder. ^{1}H NMR (600 MHz, DMSO-d_6) δ 10.02 (s, 1H), 7.99 (s, 0.5H), 7.91 (dd, J = 8.2, 1.4 Hz, 0.5H), 7.86 (dd, J = 8.2, 1.3 Hz, 0.5H), 7.82 (s, 0.5H), 7.72 (td, J = 7.6, 1.4 Hz, 0.5H), 7.65 – 7.58 (m, 1H), 7.54 (dtd, J = 9.9, 7.3, 1.4 Hz, 1H), 7.41 (ddd, J = 8.5, 7.2, 1.5 Hz, 0.5H), 7.26 – 7.23 (m, 1H), 7.21 – 7.11 (m, 3H), 7.03 – 6.99 (m, 1H), 6.44 (s, 2H), 5.88 (d, J = 6.5 Hz, 0.5H), 5.69 (d, J = 5.8 Hz, 0.5H), 5.29 (d, J = 4.9 Hz, 0.5H), 5.22 – 5.14 (m, 1H), 5.11 (dd, J = 8.2, 3.9 Hz, 1H), 4.86 (dd, J = 7.4, 4.9 Hz, 0.5H), 4.72 – 4.66 (m, 1.5H), 4.63 (d, J = 7.0 Hz, 0.5H), 4.55 (t, J = 5.4 Hz, 0.5H), 4.37 (d, J = 7.2 Hz, 0.5H), 4.28 (q, J = 4.3 Hz, 0.5H), 4.22 (d, J = 3.7 Hz, 0.5H), 3.92 (q, J = 3.7 Hz, 0.5H), 3.87 (q, J = 3.8 Hz, 0.5H), 3.66 – 3.58 (m, 1H), 3.53 (dt, J = 12.2, 5.9 Hz, 1H), 2.70 (ddd, J = 13.8, 11.0, 5.1 Hz, 0.5H), 2.59 (ddd, J = 13.7, 10.5, 5.6 Hz, 0.5H), 2.20 (ddd, J = 13.9, 10.5, 6.4 Hz, 0.5H), 2.01 – 1.94 (m, 1H), 1.90 (dddd, J = 16.0, 13.5, 7.3, 3.2 Hz, 0.5H), 1.83 – 1.77 (m, 1H) ppm. ^{13}C NMR (151 MHz, DMSO-d_6) δ 156.76, 153.80, 153.69, 151.20, 151.05, 148.41, 147.61, 141.33, 141.16, 136.98, 136.20, 135.53, 135.22, 133.59, 133.34, 128.89, 128.49, 128.38, 128.31, 128.25, 128.17, 128.01, 125.80, 125.71, 124.17, 124.03, 116.86, 116.68, 93.35, 91.44, 86.04, 85.61, 84.87, 84.81, 78.95, 78.18, 74.01, 72.70, 69.57, 69.04, 61.50, 61.25, 38.86, 38.69, 31.42, 31.35 ppm. ESI-TOF-MS $C_{26}H_{28}N_6NaO_8$ (M + Na)$^+$ calcd. *m/z* 575.1861, found *m/z* 575.1855.

2’-(Phenylethyl)Nb-*N*^{7}-methylguanosine (S38): To a solution of 2’-Nb guanosine **S37** (200 mg, 0.362 mmol) in DMF (2.90 mL) was added iodomethane (405 mg, 177 µL, 2.85 mmol). After being stirred at room temperature for 11 hours, the reaction mixture was concentrated. The residue was dissolved in dichloromethane (30.0 mL) and washed with deionized water (30.0 mL). However, the target compound was partitioned to both aqueous and organic layers. Therefore, both organic and aqueous layers were concentrated by using rotary evaporator. The residue was dissolved in acetone (20.0 mL). The solution was added dropwise to ether (200 mL) under stirring, and the resulting precipitate was collected by filtration. The solid was dried under reduced pressure to give compound **S38** (332 mg, >100% yield) as yellow solid. ^{1}H NMR (600 MHz, DMSO-d_6) δ 11.69 (s, 1H), 9.41 (s, 0.5H), 9.20 (s, 0.5H), 7.94 (dt, J = 8.3, 1.4 Hz, 0.5H), 7.86 (dd, J = 8.2, 1.4 Hz, 0.5H), 7.78 – 7.65 (m, 2H), 7.58 – 7.46 (m, 1H),

7.27 – 7.21 (m, 2H), 7.20 – 7.12 (m, 2H), 7.10 – 7.04 (m, 1H), 6.01 (dd, J = 5.3, 1.4 Hz, 0.5H), 5.80 (dd, J = 4.4, 1.4 Hz, 0.5H), 5.46 (dd, J = 6.0, 1.3 Hz, 0.5H), 5.38 (dd, J = 5.3, 1.3 Hz, 0.5H), 5.16 – 5.08 (m, 1.5H), 4.97 (dd, J = 8.2, 4.9 Hz, 0.5H), 4.91 – 4.87 (m, 0.5H), 4.84 – 4.80 (m, 0.5H), 4.69 – 4.63 (m, 1H), 4.56 – 4.50 (m, 0.5H), 4.45 (t, J = 4.7 Hz, 0.5H), 4.31 (q, J = 4.8 Hz, 1H), 4.06 – 4.03 (m, 0.5H), 3.99 (d, J = 4.7 Hz, 2H), 3.89 (s, 1H), 3.76 – 3.55 (m, 2H), 2.88 (d, J = 1.5 Hz, 1H), 2.72 (d, J = 1.5 Hz, 1H), 2.71 – 2.68 (m, 0.1H), 2.63 – 2.54 (m, 1.4H), 2.33 – 2.25 (m, 0.5H), 2.08 (d, J = 1.4 Hz, 0.5H), 2.05 – 1.82 (m, 2H) ppm. ^{13}C NMR (151 MHz, DMSO-d_6) δ 162.31, 155.76, 155.61, 153.34, 153.24, 149.23, 148.96, 148.09, 147.55, 141.30, 141.03, 137.04, 136.35, 135.94, 133.75, 133.42, 128.90, 128.52, 128.34, 128.31, 128.20, 128.13, 127.95, 125.93, 125.89, 125.81, 124.27, 123.92, 107.46, 107.37, 93.24, 92.04, 87.06, 87.03, 86.80, 86.63, 79.42, 78.78, 73.86, 73.45, 68.74, 68.58, 64.92, 60.72, 60.42, 38.90, 38.78, 35.84, 35.80, 35.73, 34.41, 31.63, 31.37, 30.77, 30.71. ESI-TOF-MS $C_{27}H_{31}N_6O_8$ (M – I$^-$)$^+$ calcd. *m/z* 567.2198, found *m/z* 567.2254.

2'-(Phenylethyl)Nb-*N*^{7}-methylguanosine 5'-monophosphate (S39): A solution of the N^7-methyl 2'-Nb-guanosine **S38** (100 mg, 144 μmol) in trimethyl phosphate (720 μL) was cooled to –10 ºC. 2,6-Lutidine (35.5 mg, 38.6 μL, 331 μmol) and phosphoryl chloride (50.8 mg, 30.9 μL, 331 μmol) were successively added to the mixture. After being stirred at –10 ºC for 6.5 hours, the reaction mixture quenched by the addition of 0.2 M TEAB buffer (pH 7.9, 1.00 mL) at –10 ºC. After warming to room temperature, the mixture was diluted with 25% CH_3CN/water (40.0 mL). The mixture was purified by using Wakosil®25C18 (Particle size: 15–30 μm (spherical), Column size: φ = 4.80 cm, h = 10.2 cm, 184 cm^3 (12 mL/min), Solvent A: 50 mM TEAA buffer (pH 6.0) contains 0.5% CH_3CN, Solvent B: CH_3CN, Linear gradient 0–90%B over 60 minutes). The fractions containing the target product were combined and concentrated. The residue was further purified by ion-exchange chromatography on QAE-Sephadex™ A-25 (φ = 3.0 cm, h = 10 cm, 70 cm^3, 6 mL/min) in linear 0–0.8 M gradient of TEAA buffer (pH 6.0) containing 0–8% CH_3CN (270 minutes). The fractions containing the target product was combined and concentrated. The product was further purified by using Wakosil®25C18 (Particle size: 15–30 μm (spherical), Column size: φ = 4.80 cm, h = 10.2 cm, 184 cm^3 (12 mL/min), Solvent A: 50 mM TEAA buffer (pH 6.0) contains 0.5% CH_3CN, Solvent B: CH_3CN, Linear gradient 0–90%B over 60 minutes). The fractions containing the target product were combined and concentrated, to afford compound **S39** (57.5 mg, 49.6% yield, contains 1 eq. of TEAA) as white solid. ^{1}H NMR (400 MHz, CD_3OD) δ 7.86 (ddd, J = 16.3, 8.2, 1.2 Hz, 1H), 7.80 – 7.64 (m, 2H), 7.46 (tdd, J = 9.6, 7.2, 3.0 Hz, 1H), 7.23 – 7.14 (m, 2H), 7.13 – 7.06 (m, 3H), 6.14 (d, J = 3.6 Hz, 0.5H), 5.96 (d, J = 3.0 Hz, 0.5H), 5.31 (dd, J = 8.0, 3.9 Hz, 0.5H), 5.19 (dd, J = 8.1, 4.1 Hz, 0.5H), 5.07 (d, J = 7.0 Hz, 0.5H), 4.97 (s, 0.5H), 4.78

(d, J = 7.1 Hz, 0.5H), 4.75 – 4.68 (m, 1H), 4.62 (dd, J = 5.0, 3.1 Hz, 0.5H), 4.54 (t, J = 5.3 Hz, 0.5H), 4.45 (t, J = 4.9 Hz, 0.5H), 4.29 – 4.20 (m, 1.5H), 4.17 (dd, J = 5.7, 2.4 Hz, 0.5H), 4.06 (s, 2.5H), 4.00 (s, 1.5H), 3.18 (q, J = 7.3 Hz, 6H, C<u>H</u>$_2$ of triethylammonium), 2.86 – 2.63 (m, 1.6H), 2.46 (ddd, J = 13.7, 10.7, 5.9 Hz, 0.4H), 2.13 – 1.96 (m, 2H), 1.94 (d, J = 0.9 Hz, 3H, C<u>H</u>$_3$COO$^-$), 1.30 (t, J = 7.3 Hz, 9H, C<u>H</u>$_3$ of triethylammonium) ppm. ^{13}C NMR (101 MHz, CDCl$_3$) δ 178.63 (CH$_3$<u>C</u>OO$^-$), 158.33, 158.16, 156.07, 151.09, 150.77, 149.96, 149.52, 142.95, 142.69, 138.90, 138.49, 134.70, 134.53, 129.81, 129.76, 129.68, 129.48, 129.44, 129.35, 129.32, 126.98, 126.82, 125.35, 125.24, 109.18, 109.12, 94.56, 89.75, 89.60, 86.65, 86.51, 81.43, 81.29, 75.72, 75.58, 69.99, 69.63, 64.10, 47.50 (<u>C</u>H$_2$ of triethylammonium), 40.70, 40.48, 36.67, 33.30, 33.18, 23.06 (<u>C</u>H$_3$COO$^-$)), 9.14 (<u>C</u>H$_3$ of triethylammonium) ppm. ^{31}P NMR (162 MHz, CD$_3$OD) δ 1.86 ppm. ESI-TOF-MS $C_{27}H_{30}N_6O_{11}P$ (M– H)$^-$ calcd. *m/z* 645.1716, found *m/z* 645.1916.

<u>2'-(Phenylethyl)Nb-Di-PureCap Analog (23):</u> To a solution of 2'-Nb-N^7-methylguanosine 5'-monophosphate **<u>S39</u>** (57.5 mg, 71.2 µmol) in DMSO (1.42 mL), guanosine 5'-diphosphate imidazolide (**S24**) (153 mg, 285 µmol) and zinc chloride (194 mg, 1.42 mmol) were added. After being incubated for 3.5 days at 37 °C, the reaction mixture was quenched by the addition of 500 mM EDTA-NaOH *aq.* (pH 8.0, 1.85 mmol, 3.70 mL) and diluted with 10% acetonitrile/water (70.0 mL). The residue was purified by ion-exchange chromatography on DEAE-Sephadex™ A-25 (φ = 4.5 cm, h = 8.4 cm, 140 cm^3, 12 mL/min) in linear 0.05–1.0 M gradient of TEAA buffer (pH 6.0) containing 0.5–10% CH$_3$CN. The fractions containing the product was collected and concentrated. The product was further purified by using Wakosil®25C18 (Particle size: 15–30 µm (spherical), Column size: φ = 4.80 cm, h = 10.2 cm, 184 cm^3 (12 mL/min), Solvent A: 50 mM TEAA buffer (pH 6.0) contains 0.5% CH$_3$CN, Solvent B: CH$_3$CN, Linear gradient 0–80%B over 60 minutes). The fractions containing the product was collected and concentrated to afford the target compound as triethylammonium salt. The product was redissolved in methanol (5.00 mL). 190 mM NaClO$_4$ in acetone (45.0 mL) was added to the mixture, and the resulting suspension was centrifuged (4,000 rpm, 10 minutes). The supernatant was discarded, and the precipitate was resuspended in acetone. The suspending-centrifugation processes were repeated additional 3 times. The precipitate was dried under reduced pressure to afford di-PureCap analog **23** (40.4 mg, 35.5 µmol, 49.9% yield) as sodium salt. The yield was calculated by using absorbance of the product at 260 nm measured by NanoDrop2000 spectrometer. Extinction coefficient (ε_{260}) = 26,300 M^{-1}•cm^{-1} was used for calculation (A. M. Rydzik, *et al., Nucleic Acids Res.*, **2017**, *45*(15), 8661–8675; S. G. Chaulk, *et al., Nat. Protocol.*, **2007**, *2*(5), 1052–1058). ^{1}H NMR (400 MHz, D$_2$O) δ 7.94

(d, J = 8.3 Hz, 1H), 7.47 (t, J = 9.6 Hz, 1H), 7.31 (d, J = 17.2 Hz, 2H), 7.18 – 7.02 (m, 1H), 6.84 – 6.48 (m, 5H), 5.94 – 5.85 (m, 0.5H), 5.73 (dq, J = 9.4, 5.0 Hz, 1.5H), 4.97 (s, 0.5H), 4.85 (d, J = 15.2 Hz, 1.5H), 4.62 – 4.18 (m, 11H), 4.04 – 3.84 (m, 3H), 2.20 (s, 2H), 1.42 (s, 2H) ppm. ^{13}C NMR (101 MHz, D_2O) δ 158.80, 158.51, 153.81, 151.22, 149.39, 149.03, 147.35, 146.41, 140.84, 140.64, 137.13, 136.94, 136.61, 133.89, 133.56, 129.78, 128.12, 127.93, 127.78, 125.73, 125.47, 124.16, 120.33, 115.80, 115.20, 107.91, 107.74, 93.56, 87.17, 86.89, 84.54, 83.43, 79.33, 74.83, 74.20, 70.24, 68.20, 65.38, 64.73, 38.58, 38.20, 36.01, 31.54, 31.04, 30.27 ppm. ^{31}P NMR (162 MHz, D_2O) δ –10.72 (t, J = 18.3 Hz, 2P), –22.07 (t, J = 19.3 Hz, 1P) ppm. ESI-TOF-MS $C_{37}H_{43}N_{11}O_{21}P_3$ (M– H)$^-$ calcd. m/z 1070.1853, found m/z 1070.1910.

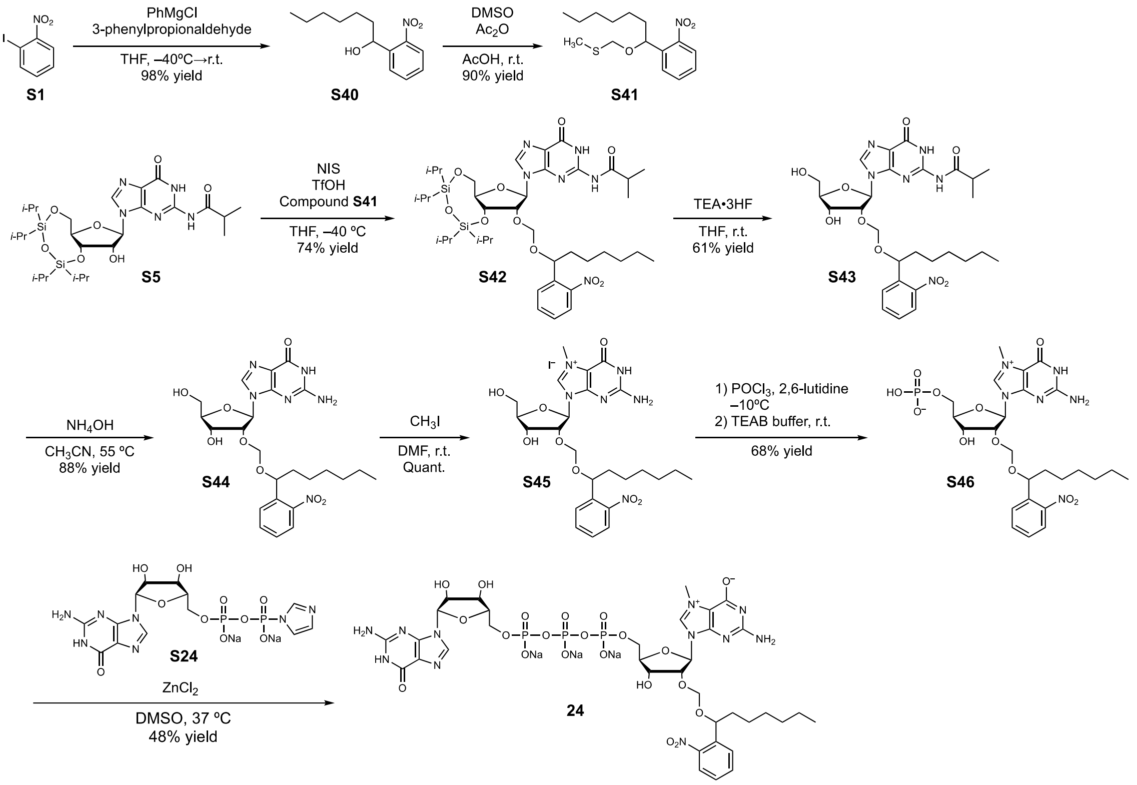

Supplementary Figure 13. Synthesis of 2'-Nb-Di-PureCap Analog with *n*-Hexyl Group (**24**)

(*n*-Hexyl)nitrobenzyl Alcohol (S40): A mixture of 1-iodo-2-nitrobenzene (**S1**) (5.00 g, 20.1 mmol) in THF (41.9 mL) was cooled to –40 ºC. A solution of phenylmagnesium chloride in THF (2 M, 13.1 mL, 26.1 mmol) was added to the mixture and stirred at –40 ºC for 1.5 hour. Heptanal (3.21 g, 3.91 mL, 28.1 mmol) was added to the reaction mixture and stirred at –40 ºC for 1 hour. The reaction mixture was quenched by the addition of aqueous solution of *sat.* NH_4Cl (20.0 mL). The mixture was extracted 3 times with dichloromethane (20.0 mL). The

organic layer was dried over Na_2SO_4 and concentrated. The residue was purified by silica gel column chromatography eluted by 9.1→50% ethyl acetate/hexane, to afford compound **S40** (4.68 g, 98.1% yield) as brown liquid. ^{1}H NMR (600 MHz, $CDCl_3$) *δ* 7.78 (dd, *J* = 8.2, 1.4 Hz, 1H), 7.70 (dd, *J* = 7.9, 1.4 Hz, 1H), 7.52 (td, *J* = 7.6, 1.4 Hz, 1H), 7.34 – 7.29 (m, 1H), 5.15 (dd, *J* = 8.5, 4.0 Hz, 1H), 3.52 (s, 1H), 1.70 (tdd, *J* = 10.3, 7.8, 3.9 Hz, 1H), 1.61 (dddd, *J* = 14.8, 10.0, 8.5, 4.7 Hz, 1H), 1.45 (dh, *J* = 14.9, 5.3 Hz, 1H), 1.34 (dddd, *J* = 13.2, 9.8, 6.2, 3.6 Hz, 1H), 1.28 – 1.18 (m, 7H), 0.83 (q, *J* = 6.5 Hz, 3H) ppm. ^{13}C NMR (151 MHz, $CDCl_3$) *δ* 156.03, 147.55, 140.42, 133.30, 129.39, 127.89, 127.77, 124.08, 120.04, 115.31, 69.13, 38.30, 31.63, 28.90, 25.96, 22.49, 13.95, 13.87 ppm. ESI-TOF-MS $C_{13}H_{19}NNaO_3$ (M + Na)$^+$ calcd. *m/z* 260.1257, found *m/z* 260.1153.

(*n*-Hexyl)nitrobenzyl alcohol methylthioacetal Derivative (S41): To a solution of nitrobenzyl alcohol **S40** (4.68 g, 19.6 mmol) in acetic acid (47.3 g, 45.1 mL, 788 mmol) was added DMSO (30.8 g, 28.0 mL, 394 mmol) and acetic anhydride (40.2 g, 37.2 mL, 394 mmol). After being stirred at room temperature for 4 days, the reaction mixture was added dropwise to 10 M KOH *aq.* (177 mL, 1.77 mol) in an ice-bath. The mixture was stirred at room temperature for 1 hour and then extracted with ethyl acetate (200 mL). The organic layer was washed with brine and dried over Na_2SO_4. The residue was dissolved in hexane and subjected to silica gel column chromatography eluted by 3.8–17% ethyl acetate/hexane, to afford methylthioacetal **S41** (5.30 g, 90.4% yield) as brown oil. ^{1}H NMR (600 MHz, $CDCl_3$) δ 7.88 (dd, *J* = 8.2, 1.4 Hz, 1H), 7.70 (dd, *J* = 7.9, 1.5 Hz, 1H), 7.65 – 7.58 (m, 1H), 7.39 (ddd, *J* = 8.2, 7.3, 1.5 Hz, 1H), 5.26 (dd, *J* = 8.9, 3.5 Hz, 1H), 4.60 (d, *J* = 11.6 Hz, 1H), 4.23 (d, *J* = 11.6 Hz, 1H), 2.10 (s, 3H), 1.82 – 1.67 (m, 2H), 1.58 – 1.41 (m, 2H), 1.39 – 1.33 (m, 1H), 1.31 – 1.24 (m, 5H), 0.89 – 0.82 (m, 3H) ppm. ^{13}C NMR (151 MHz, $CDCl_3$) *δ* 148.82, 138.49, 133.40, 128.37, 128.14, 124.37, 73.95, 73.62, 37.94, 31.84, 28.96, 26.15, 22.66, 14.26, 14.15 ppm. ESI-TOF-MS $C_{15}H_{23}NNaO_3S$ (M + Na)$^+$ calcd. *m/z* 320.1291, found *m/z* 320.1171

***N*^{2}-Isobutyryl-3',5'-*O*-TIPDS-2'-(*n*-Hexyl)Nb-guanosine (S42):** To a suspension of *N*^{2}-Isobutyryl-3',5'-*O*-(1,1,3,3-tetraisopropyldisiloxane-1,3-diyl) guanosine (**S5**) (2.00 g, 3.35 mmol), nitrobenzyl alcohol methylthioacetal **S41** (1.30 g, 4.36 mmol), and powdery molecular sieves 3Å (1.50 g), in tetrahydrofuran (33.5 mL), was added *N*-iodosuccinimide (981 mg, 4.36 mmol) and then cooled to –40 ºC. To the cooled suspension was added trifluoromethanesulfonic acid (755 mg, 447 μL, 5.03 mmol) and stirred at –40ºC for 8.5 hours. The reaction mixture was quenched by the addition of triethylamine (16.8 mL) and then diluted with ethyl acetate (100 mL). The mixture was washed with saturated *aq.* $NaHCO_3$

(100 mL x2), saturated *aq.* sodium thiosulfate (100 mL x2), and brine (100 mL x1), successively. The organic layer was dried over Na_2SO_4 and concentrated. The residue was purified by silica gel column chromatography eluted by 0–4.8% methanol/dichloromethane, to afford compound **S42** (2.08 g, 73.5% yield) as brown foam. ^{1}H NMR (600 MHz, $CDCl_3$, not pure) δ 12.32 – 12.06 (m, 1H), 10.50 – 10.37 (m, 0.5H), 9.40 – 9.26 (m, 0.5H), 8.01 (s, 0.2H), 7.92 (d, *J* = 8.7 Hz, 0.8H), 7.82 (s, 0.3H), 7.72 (dd, *J* = 8.2, 1.4 Hz, 0.3H), 7.68 – 7.63 (m, 0.5H), 7.58 (dd, *J* = 7.9, 1.6 Hz, 0.3H), 7.47 – 7.40 (m, 0.5H), 7.25 – 7.21 (m, 0.2H), 5.83 – 5.75 (m, 0.7H), 5.60 (s, 0.3H), 5.31 (dd, *J* = 8.8, 3.2 Hz, 0.3H), 5.28 – 5.19 (m, 0.3H), 5.15 (d, *J* = 6.5 Hz, 0.3H), 5.06 (d, *J* = 6.5 Hz, 0.3H), 4.66 (d, *J* = 6.5 Hz, 0.2H), 4.58 (d, *J* = 6.5 Hz, 0.3H), 4.50 (dd, *J* = 9.5, 4.1 Hz, 0.3H), 4.42 (ddd, *J* = 15.0, 8.9, 4.6 Hz, 0.8H), 4.27 (d, *J* = 5.1 Hz, 0.5H), 4.21 – 4.17 (m, 0.6H), 4.17 – 4.10 (m, 1H), 4.08 (dt, *J* = 8.6, 2.7 Hz, 0.5H), 4.03 – 3.90 (m, 1.7H), 3.84 (s, 0.4H), 3.39 – 3.35 (m, 0.5H), 3.30 (s, 0.2H), 2.89 (p, *J* = 6.9 Hz, 0.4H), 2.84 – 2.65 (m, 0.7H), 1.81 – 1.61 (m, 1.5H), 1.58 (p, *J* = 2.9 Hz, 0.7H), 1.28 – 1.16 (m, 11H), 1.10 – 0.90 (m, 36H), 0.83 – 0.72 (m, 3H) ppm. ^{13}C NMR (151 MHz, $CDCl_3$, not pure) δ 179.77, 179.30, 178.99, 155.93, 155.70, 155.67, 148.52, 148.20, 147.90, 147.84, 147.42, 147.34, 138.44, 137.96, 136.53, 136.41, 136.30, 133.93, 133.31, 128.52, 128.49, 128.42, 128.20, 128.06, 124.52, 124.03, 121.48, 121.38, 93.80, 91.06, 88.98, 88.10, 87.36, 81.69, 81.21, 81.01, 79.05, 76.36, 75.31, 74.86, 72.10, 70.62, 70.07, 69.57, 68.50, 60.62, 59.54, 59.45, 37.97, 37.89, 36.48, 36.32, 36.12, 31.68, 31.64, 31.53, 29.04, 26.52, 25.77, 25.60, 22.61, 22.46, 22.42, 19.21, 19.10, 19.03, 18.95, 18.86, 17.50, 17.47, 17.44, 17.39, 17.32, 17.30, 17.26, 17.15, 17.07, 17.04, 17.02, 16.98, 16.94, 16.87, 16.84, 14.06, 13.98, 13.41, 13.36, 13.07, 12.98, 12.91, 12.63, 12.53 ppm. ESI-TOF-MS $C_{40}H_{64}N_6O_{10}Si_2$ $(M + Na)^+$ calcd. *m/z* 867.4115, found *m/z* 867.4035

N^2-Isobutyryl-2'-(*n*-Hexyl)Nb-guanosine (S43): To a solution of the protected guanosine **S42** (2.08 g, 2.46 mmol) in THF (10.0 mL) was added triethylamine trihydrofluoride (1.98 g, 12.3 mmol). After being stirred at room temperature for 13 hours, the reaction mixture was concentrated by using rotary evaporator. The residue was diluted with dichloromethane (100 mL) and washed with saturated *aq.* $NaHCO_3$ (100 mL). The aqueous layer was extracted with dichloromethane (100 mL x2). The organic layer was dried over Na_2SO_4 and concentrated. The residue was purified by silica gel column chromatography eluted by 2.4–9.1% methanol/dichloromethane, to afford compound **S43** (900 mg, 60.8% yield) as white foam. ^{1}H NMR (600 MHz, $CDCl_3$) δ 12.46 – 12.29 (m, 1H), 10.49 – 10.28 (m, 1H), 8.27 (s, 0.5H), 8.03 (s, 0.5H), 7.81 – 7.64 (m, 1H), 7.56 – 7.44 (m, 1.5H), 7.42 – 7.28 (m, 1H), 7.23 (d, *J* = 7.8 Hz, 0.5H), 5.98 (s, 0.5H), 5.80 (s, 0.5H), 5.11 – 5.04 (m, 1H), 5.01 – 4.93 (m, 1H), 4.78 – 4.48 (m, 5H), 4.12 (d, *J* = 7.6 Hz, 1H), 3.91 – 3.83 (m, 0.8H), 3.77 (s, 0.5H), 3.65 (d,

J = 13.3 Hz, 0.5H), 3.37 (d, J = 5.9 Hz, 0.2H), 2.94 – 2.83 (m, 1H), 1.65 – 1.43 (m, 2.5H), 1.22 (s, 6.5H), 1.17 – 1.08 (m, 5.2H), 1.02 (d, J = 6.3 Hz, 1.8H), 0.73 (dt, J = 10.6, 6.9 Hz, 3H) ppm. ^{13}C NMR (151 MHz, $CDCl_3$) δ 180.29, 180.16, 155.70, 148.51, 148.35, 148.18, 148.02, 147.47, 139.01, 137.91, 137.48, 133.42, 133.15, 128.45, 128.30, 128.19, 124.11, 123.98, 120.97, 120.88, 94.30, 93.08, 87.79, 86.10, 79.72, 79.46, 75.07, 73.96, 70.59, 70.47, 70.16, 61.90, 37.52, 37.45, 36.14, 36.06, 31.56, 31.41, 29.02, 28.97, 26.48, 25.34, 22.51, 22.40, 19.06, 19.03, 18.99, 18.96, 13.99 ppm. ESI-TOF-MS $C_{28}H_{38}N_6NaO_9$ (M + Na)$^+$ calcd. *m/z* 625.2593, found *m/z* 625.2603.

2'-(*n*-Hexyl)Nb-guanosine (S44): To a solution of the protected guanosine **S43** (900 mg, 1.49 mmol) in acetonitrile (9.77 mL) was added 28% ammonium hydroxide solution (19.5 mL). The mixture was heated to 55 ºC. After being stirred at 55 ºC for 11 hours, the reaction mixture was concentrated and azeotroped with benzene (x1). The residue was dissolved in dichloromethane (30.0mL) and suspended by the addition of diethyl ether (200 mL). The resulting precipitate was collected by filtration and rinsed with diethyl ether. The obtained solid was dried under vacuum to afford compound **S44** (697 mg, 87.8% yield) as white powder. ^{1}H NMR (600 MHz, DMSO-d_6) δ 9.79 (s, 1H), 7.94 (s, 0.5H), 7.88 (d, J = 7.2 Hz, 0.5H), 7.84 (d, J = 8.1 Hz, 0.5H), 7.80 (s, 0.5H), 7.70 (t, J = 7.6 Hz, 0.5H), 7.58 – 7.49 (m, 2H), 7.43 – 7.38 (m, 0.5H), 6.49 (s, 2H), 5.84 (d, J = 6.2 Hz, 0.5H), 5.70 (d, J = 5.9 Hz, 0.5H), 5.04 (dd, J = 7.9, 4.3 Hz, 1H), 4.79 (dd, J = 7.7, 4.9 Hz, 0.5H), 4.69 – 4.63 (m, 1.5H), 4.57 (d, J = 7.0 Hz, 0.5H), 4.51 (t, J = 5.4 Hz, 1H), 4.34 (d, J = 7.3 Hz, 0.5H), 4.27 – 4.22 (m, 1H), 3.92 – 3.85 (m, 1H), 3.62 (td, J = 12.4, 3.7 Hz, 1H), 3.56 – 3.49 (m, 1H), 3.36 (q, J = 7.0 Hz, 1H), 1.66 – 1.56 (m, 1H), 1.48 – 1.36 (m, 1H), 1.23 – 1.13 (m, 5H), 1.08 (t, J = 7.0 Hz, 1.5H), 1.02 (dt, J = 6.9, 3.4 Hz, 1.5H), 0.80 (dd, J = 17.4, 7.1 Hz, 3H) ppm. ^{13}C NMR (151 MHz, DMSO-d_6) δ 156.95, 153.86, 153.81, 151.18, 151.07, 148.54, 147.71, 137.25, 136.48, 135.65, 135.22, 133.46, 133.28, 128.76, 128.39, 128.35, 128.12, 124.00, 123.94, 116.92, 116.70, 93.21, 91.53, 85.98, 85.66, 85.15, 84.84, 78.85, 77.98, 74.09, 73.03, 69.64, 69.16, 64.95, 61.54, 61.31, 37.16, 36.84, 31.12, 30.89, 28.40, 28.36, 25.01, 24.97, 22.02, 15.18, 13.94 ppm. ESI-TOF-MS $C_{24}H_{32}N_6NaO_8$ (M– H)$^-$ calcd. *m/z* 555.2174, found *m/z* 555.2163.

2'-(*n*-Hexyl)Nb-*N*^{7}-methylguanosine (S45): To a solution of 2'-modified guanosine **S44** (200 mg, 0.376 mmol) in DMF (3.01 mL) was added iodomethane (420 mg, 184 µL, 2.96 mmol). After being stirred at room temperature for 16 hours, the reaction mixture was concentrated. The residue was dissolved in acetone (10.0 mL). The solution was added dropwise to ether (150 mL) under stirring, and the resulting precipitate was collected by filtration. The solid was dried under reduced pressure to give compound **S45** (311 mg, >100%

yield) as pale-yellow solid. ^{1}H NMR (600 MHz, DMSO-d_6) *δ* 11.78 (d, *J* = 3.0 Hz, 0.5H), 11.69 (d, *J* = 3.0 Hz, 0.5H), 9.43 (d, *J* = 3.3 Hz, 0.5H), 9.21 (d, *J* = 3.3 Hz, 0.5H), 7.93 – 7.89 (m, 0.5H), 7.84 (dd, *J* = 8.2, 3.1 Hz, 0.5H), 7.75 – 7.71 (m, 0.5H), 7.64 (ddd, *J* = 16.8, 8.7, 2.7 Hz, 1.5H), 7.53 (td, *J* = 7.7, 2.5 Hz, 0.5H), 7.49 – 7.45 (m, 0.5H), 5.97 (dd, *J* = 5.5, 3.3 Hz, 0.5H), 5.80 (t, *J* = 4.1 Hz, 0.5H), 5.44 – 5.31 (m, 1H), 5.15 – 5.02 (m, 1.5H), 4.84 (ddd, *J* = 10.7, 8.0, 3.8 Hz, 1H), 4.75 (dd, *J* = 7.2, 3.6 Hz, 0.5H), 4.64 – 4.57 (m, 1H), 4.48 – 4.39 (m, 1H), 4.26 (dd, *J* = 8.5, 4.3 Hz, 1H), 4.06 (d, *J* = 3.4 Hz, 1.5H), 4.02 (t, *J* = 3.8 Hz, 0.5H), 3.99 (d, *J* = 3.5 Hz, 1.5H), 3.97 (t, *J* = 3.9 Hz, 0.5H), 3.72 – 3.63 (m, 1H), 3.58 (dq, *J* = 12.6, 3.6 Hz, 1H), 1.67 – 1.51 (m, 2H), 1.22 – 1.17 (m, 4H), 1.09 (dtd, *J* = 14.1, 7.0, 3.7 Hz, 4H), 0.83 – 0.79 (m, 3H) ppm. ^{13}C NMR (151 MHz, DMSO-d_6) δ 155.77, 155.66, 155.62, 153.40, 153.35, 149.30, 149.12, 149.03, 148.37, 147.61, 137.52, 137.24, 137.02, 136.63, 136.29, 136.20, 135.81, 133.72, 133.52, 133.35, 129.08, 128.63, 128.23, 128.13, 127.50, 124.25, 123.99, 123.68, 107.49, 107.34, 94.16, 93.32, 93.16, 92.63, 91.58, 87.66, 87.48, 87.32, 87.12, 87.03, 86.90, 86.49, 86.19, 86.00, 80.22, 79.70, 79.25, 79.00, 78.22, 74.30, 73.52, 73.43, 73.18, 72.53, 69.56, 68.77, 68.41, 68.19, 67.84, 65.88, 65.25, 64.94, 64.31, 63.99, 61.34, 61.18, 60.69, 60.50, 60.00, 59.81, 37.59, 37.31, 37.03, 36.80, 36.63, 36.17, 36.07, 35.64, 35.11, 34.63, 31.73, 31.10, 31.00, 30.33, 29.21, 28.50, 25.24, 25.05, 24.91, 22.86, 22.26, 22.05, 22.00, 15.87, 15.22, 15.14, 15.06, 14.86, 14.49, 14.40, 14.25, 14.05, 13.85, 13.03 ppm. ESI-TOF-MS $C_{25}H_{35}N_6O_8$ (M – I$^-$)$^+$ calcd. *m/z* 547.2511, found *m/z* 547.2549.

2'-(*n*-Hexyl)Nb-*N*^{7}-methylguanosine 5'-monophosphate (S46): A solution of the 2'-Nb-*N*^{7}-methylguanosine **S45** (100 mg, 148 μmol) in trimethyl phosphate (740 μL) was cooled to –10 ºC. 2,6-Lutidine (36.4 mg, 39.6 μL, 340 μmol) and phosphoryl chloride (52.1 mg, 31.8 μL, 340 μmol) were successively added to the mixture. After being stirred at –10 ºC for 10 hours, the reaction mixture quenched by the addition of 0.2 M TEAB buffer (pH 7.9, 1.00 mL) at –10 ºC. After warming to room temperature, the mixture was diluted with 20% CH_3CN/water (40.0 mL). The mixture was purified by using CHRMATREX C8 MB-100-40/75 column (Column size: φ = 3.0 cm, *h* = 10 cm, 70 cm^3 (7 mL/min), Solvent A: 50 mM TEAA buffer (pH 6.0) contains 0.5% CH_3CN, Solvent B: CH_3CN, Linear gradient 0–90%B over 60 minutes). The fractions containing the target product were combined, concentrated, and lyophilized, to afford compound **S46** (88.1 mg, 68.3% yield, contains 1.8 eq. of TEAA) as white solid. ^{1}H NMR (400 MHz, CD_3OD) *δ* 7.89 – 7.79 (m, 1H), 7.78 – 7.63 (m, 2H), 7.53 – 7.38 (m, 1H), 6.13 (d, *J* = 3.3 Hz, 0.5H), 5.96 (d, *J* = 3.3 Hz, 0.5H), 5.24 (dd, *J* = 7.5, 4.6 Hz, 0.5H), 5.15 (dd, *J* = 7.4, 4.8 Hz, 1H), 5.04 (d, *J* = 7.1 Hz, 1H), 4.96 (d, *J* = 7.0 Hz, 0.5H), 4.79 (d, *J* = 7.1 Hz, 0.5H), 4.73 (d, *J* = 6.8 Hz, 0.5H), 4.68 – 4.60 (m, 1H), 4.52 (t, *J* = 5.2 Hz, 0.5H), 4.43 (t,

J = 5.2 Hz, 0.5H), 4.30 – 4.19 (m, 2H), 4.15 (s, 2H), 4.14 – 4.05 (m, 3H), 3.18 (q, *J* = 7.3 Hz, 10H, C<u>H</u>$_2$ of triethylammonium), 1.93 (s, 5.4H, C<u>H</u>$_3$COO$^–$), 1.80 – 1.60 (m, 2.5H), 1.31 (t, *J* = 7.4 Hz, 16H), 1.21 (d, *J* = 8.9 Hz, 7.5H, C<u>H</u>$_3$ of triethylammonium), 0.83 (t, *J* = 7.0 Hz, 3H) ppm. ^{13}C NMR (101 MHz, CD$_3$OD) *δ* 178.93 (CH$_3$<u>C</u>OO$^–$), 158.56, 158.34, 156.44, 156.24, 151.00, 150.81, 150.10, 149.51, 139.05, 138.58, 134.53, 134.39, 129.78, 129.73, 129.64, 129.42, 125.14, 125.05, 109.17, 109.01, 94.64, 89.66, 86.56, 86.48, 86.28, 81.37, 81.29, 75.83, 75.39, 69.83, 69.76, 64.14, 63.99, 47.30 (<u>C</u>H$_2$ of triethylammonium), 38.65, 38.60, 36.76, 36.71, 32.79, 30.24, 30.17, 26.64, 26.53, 23.64, 23.60, 23.33 (<u>C</u>H$_3$COO$^–$), 14.47, 14.44, 9.08 (<u>C</u>H$_3$ of triethylammonium) ppm. ^{31}P NMR (162 MHz, CD$_3$OD) *δ* 1.97 ppm. ESI-TOF-MS $C_{25}H_{34}N_6O_{11}P$ (M– H)$^–$ calcd. *m/z* 625.2029, found *m/z* 625.2056

<u>**2'-(*n*-Hexyl)Nb-Di-PureCap Analog (24):**</u> To a solution of 2'-Nb-*N*^{7}-methylguanosine 5'-monophosphate **S46** (88.1 mg, 101 μmol) in DMSO (2.02 mL), guanosine 5'-diphosphate imidazolide (**S24**) (217 mg, 404 μmol) and zinc chloride (275 mg, 2.02 mmol) were added. After being incubated for 3.5 days at 37 ºC, the reaction mixture was quenched by the addition of 500 mM EDTA-NaOH *aq.* (pH 8.0, 2.63 mmol, 5.26 mL) and diluted with 10% acetonitrile/water (80.0 mL). The residue was purified by ion-exchange chromatography on DEAE-Sephadex™ A-25 (φ = 4.5 cm, *h* = 8.4 cm, 140 cm^3, 12 mL/min) in linear 0.05–1.0 M gradient of TEAA buffer (pH 6.0) containing 0.5–10% CH$_3$CN. The fractions containing the product was collected and concentrated. The product was further purified by using Wakosil®25C18 (Particle size: 15–30 μm (spherical), Column size: φ = 4.80 cm, *h* = 10.2 cm, 184 cm^3 (12 mL/min), Solvent A: 50 mM TEAA buffer (pH 6.0) contains 0.5% CH$_3$CN, Solvent B: CH$_3$CN, Linear gradient 0–80%B over 60 minutes). The fractions containing the product was collected and concentrated to afford the target compound as triethylammonium salt. The product was redissolved in methanol (5.00 mL). 190 mM NaClO$_4$ in acetone (45.0 mL) was added to the mixture, and the resulting suspension was centrifuged (4,000 rpm, 10 minutes). The supernatant was discarded, and the precipitate was resuspended in acetone. The suspending-centrifugation processes were repeated additional 3 times. The precipitate was dried under reduced pressure to afford di-PureCap analog **24** (54.4 mg, 48.7 μmol, 48.2% yield) as sodium salt. The yields were calculated by using absorbance of the product at 260 nm measured by NanoDrop. Extinction coefficient (ε_{260}) = 26,300 M$^{–1}$•cm$^{–1}$ was used for calculation (A. M. Rydzik, *et al., Nucleic Acids Res.*, **2017**, *45*(15), 8661–8675; S. G. Chaulk, *et al., Nat. Protocol.*, **2007**, *2*(5), 1052–1058). ^{1}H NMR (400 MHz, D$_2$O) *δ* 7.96 (d, *J* = 9.3 Hz, 1H), 7.70 – 7.54 (m, 1H), 7.54 – 7.32 (m, 2H), 7.23 (s, 1H), 5.98 (s, 0.5H), 5.78 (d, *J* = 11.3 Hz, 1.5H), 5.01 (s, 2.5H), 4.75 (s, 0.5H), 4.69 – 4.18 (m, 10H), 4.16 – 3.97 (m, 3H), 1.42 – 1.09 (m, 2H), 0.97 – 0.39 (m, 8H), 0.26 – 0.00 (m, 3H) ppm. ^{13}C NMR (101 MHz, D$_2$O) *δ*

159.40, 158.59, 153.89, 151.21, 149.65, 149.29, 147.84, 146.62, 137.34, 136.80, 133.69, 129.65, 128.16, 124.02, 120.08, 115.79, 115.20, 107.94, 107.67, 93.67, 87.08, 83.44, 79.30, 74.77, 74.44, 70.25, 68.80, 65.37, 37.02, 36.16, 30.79, 30.28, 28.56, 24.88, 24.47, 23.30, 21.91, 21.81, 13.27, 13.15 ppm. ^{31}P NMR (162 MHz, D_2O) δ –10.62 (t, J = 15.0 Hz, 2P), –21.90 (t, J = 19.3 Hz, 1P) ppm. ESI-TOF-MS $C_{35}H_{46}N_{11}O_{21}P_3$ $(M-2H)^{2-}$ calcd. m/z 524.6047, found m/z 524.6069.

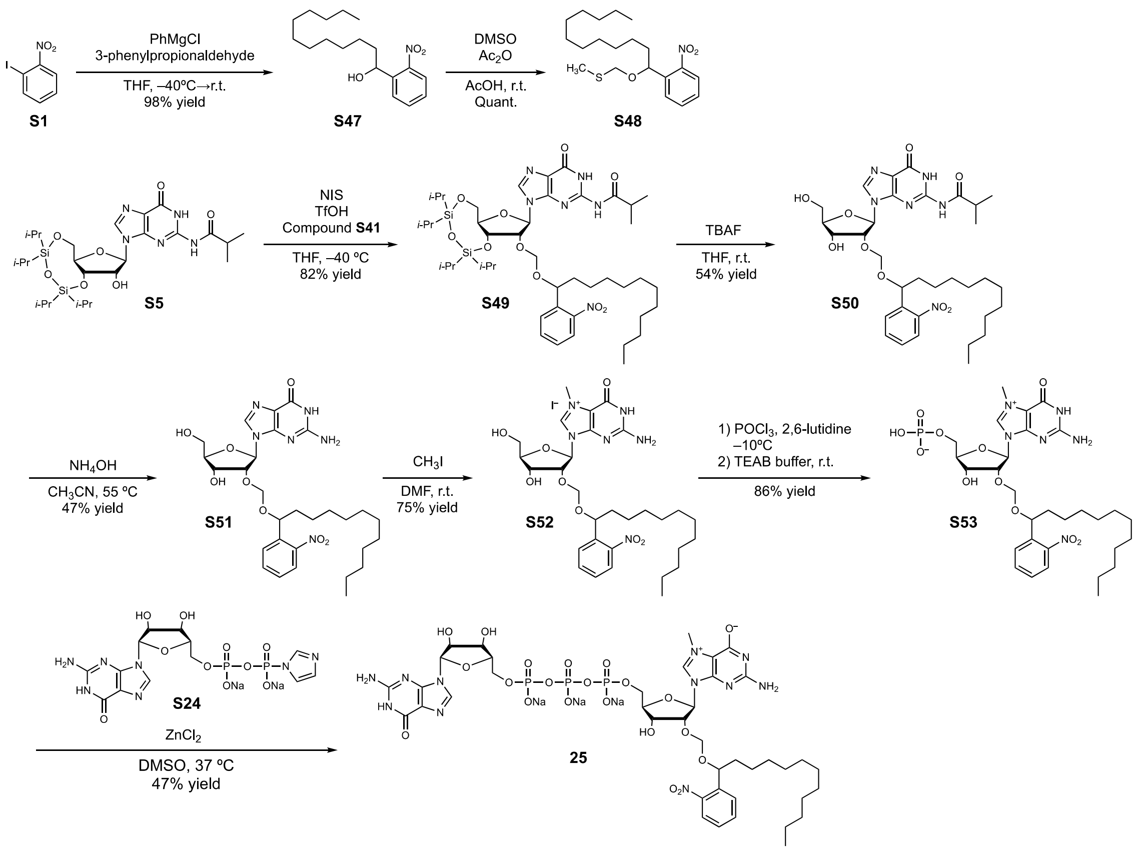

Supplementary Figure 14. Synthesis of 2'-Nb-Di-PureCap Analog with *n*-Undecyl Group (**25**)

<u>**(*n*-Undecyl)nitrobenzyl Alcohol (S47):**</u> A mixture of 1-iodo-2-nitrobenzene (**S1**) (5.00 g, 20.1 mmol) in THF (41.9 mL) was cooled to –40 °C. A solution of phenylmagnesium chloride in THF (2 M, 13.1 mL, 26.1 mmol) was added to the mixture and stirred at –40 °C for 1 hour. Dodecanal (5.18 g, 6.24 mL, 28.1 mmol) was added to the reaction mixture and stirred at –40 °C for 1 hour. The reaction mixture was quenched by the addition of aqueous solution of *sat.* NH_4Cl (20.0 mL). The mixture was extracted 3 times with dichloromethane (20.0 mL). The organic layer was dried over Na_2SO_4 and concentrated. The residue was purified by silica gel column chromatography eluted by 17→50% ethyl acetate/hexane, to afford

compound **S47** (6.08 g, 98.4% yield) as brown oil. ^{1}H NMR (60 MHz, $CDCl_3$) *δ* 8.09–6.79 (m, 5H, overlapping with the signal of $CHCl_3$), 5.59–4.86 (m, 1H), 1.34 (s, 20H), 0.99 (d, *J* = 4.8 Hz, 3H) ppm. ^{13}C NMR (15 MHz, $CDCl_3$) *δ* 147.46, 140.76, 137.30, 133.11, 130.05, 129.25, 127.92, 127.57, 123.94, 119.79, 115.41, 68.96, 60.50, 38.59, 31.88, 29.63, 29.33, 26.10, 22.62, 13.97, 13.88 ppm. ESI-TOF-MS $C_{18}H_{29}NNaO_3$ (M + Na)$^+$ calcd. *m/z* 330.2040, found *m/z* 330.1920.

(*n*-Undecyl)nitrobenzyl alcohol methylthioacetal Derivative (S48): To a solution of the nitrobenzyl alcohol derivative **S47** (6.08 g, 19.8 mmol) in acetic acid (47.6 g, 45.3 mL, 792 mmol), DMSO (30.9 g, 28.1 mL, 396 mmol) and acetic anhydride (40.4 g, 37.4 mL, 396 mmol) were added. After being stirred at room temperature for 4 days, the reaction mixture was added dropwise to 10 M KOH *aq.* (178 mL, 1.78 mol) in an ice-bath. The mixture was stirred at room temperature for 4 hours and then extracted with ethyl acetate. The organic layer was washed with brine and dried over Na_2SO_4. The residue was dissolved in hexane and subjected to silica gel column chromatography eluted by 4.8–17% ethyl acetate/hexane, to afford methylthioacetal **S48** (10.6 g, >100% yield) as yellow oil. ^{1}H NMR (600 MHz, $CDCl_3$) *δ* 7.88 (dd, *J* = 8.2, 1.3 Hz, 1H), 7.70 (dd, *J* = 7.9, 1.5 Hz, 1H), 7.64–7.58 (m, 1H), 7.39 (ddd, *J* = 8.2, 7.3, 1.5 Hz, 1H), 5.26 (dd, *J* = 8.9, 3.4 Hz, 1H), 4.60 (d, *J* = 11.6 Hz, 1H), 4.23 (d, *J* = 11.6 Hz, 1H), 2.10 (s, 3H), 1.81–1.67 (m, 2H), 1.58–1.41 (m, 2H), 1.39–1.33 (m, 1H), 1.29–1.21 (m, 15H), 0.85 (t, *J* = 7.1 Hz, 3H) ppm. ^{13}C NMR (151 MHz, $CDCl_3$) *δ* 148.81, 138.49, 133.35, 128.35, 128.10, 124.33, 73.92, 73.60, 37.92, 31.96, 29.70, 29.66, 29.64, 29.61, 29.38, 29.30, 26.18, 22.73, 14.23, 14.15 ppm. ESI-TOF-MS $C_{20}H_{33}NNaO_3S$ (M + Na)$^+$ calcd. *m/z* 390.2073, found *m/z* 390.2031.

***N*^{2}-Isobutyryl-3',5'-*O*-TIPDS-2'-(*n*-Undecyl)Nb-guanosine (S49):** To a suspension of *N*^{2}-isobutyryl-3',5'-*O*-(1,1,3,3-tetraisopropyldisiloxane-1,3-diyl) guanosine (**S5**) (2.00 g, 3.36 mmol), methylthioacetal derivative **S48** (1.61 g, 4.37 mmol), and powdery molecular sieves 3Å (1.50 g), in tetrahydrofuran (33.6 mL), *N*-iodosuccinimide (983 mg, 4.37 mmol) was added and then cooled to –40 ºC. After 20 minutes at –40ºC, to the suspension was added trifluoromethanesulfonic acid (756 mg, 448 µL, 5.04 mmol) and stirred at –40ºC for 16.5 h. The reaction mixture was quenched by the addition of triethylamine (16.8 mL) and then diluted with ethyl acetate (100 mL). The mixture was washed with saturated *aq.* $NaHCO_3$ (100 mL x2), saturated *aq.* sodium thiosulfate (100 mL x2), and brine (100 mL x1), successively. The organic layer was dried over Na_2SO_4 and concentrated. The residue was purified by silica gel column chromatography eluted by 0–4.8% methanol/dichloromethane, to afford compound **S49** (2.53 g, 82.1% yield) as brown foam. ^{1}H NMR (600 MHz, $CDCl_3$,

mixture of the target compound and the starting material) δ 12.32–12.08 (m, 1.5H), 10.79 (s, 0.7H), 9.70 (s, 0.5H), 9.39 (s, 0.3H), 7.99 (s, 0.3H), 7.93–7.88 (m, 1H), 7.82 (s, 0.3H), 7.70 (dd, *J* = 8.2, 1.4 Hz, 0.4H), 7.63 (dd, *J* = 5.4, 2.0 Hz, 0.6H), 7.54 (dd, *J* = 7.9, 1.5 Hz, 0.3H), 7.41 (dddd, *J* = 10.6, 9.0, 6.9, 2.1 Hz, 0.6H), 7.23 (td, *J* = 7.9, 1.5 Hz, 0.3H), 5.79 (s, 0.4H), 5.73 (s, 0.9H), 5.58 (s, 0.4H), 5.29 (dd, *J* = 8.6, 3.3 Hz, 0.4H), 5.24 (s, 1.5H), 5.21 (dd, *J* = 7.6, 4.6 Hz, 0.5H), 5.13 (d, *J* = 6.5 Hz, 0.3H), 5.03 (d, *J* = 6.6 Hz, 0.4H), 4.63 (d, *J* = 6.6 Hz, 0.4H), 4.58 (d, *J* = 6.5 Hz, 0.3H), 4.48 (dd, *J* = 9.4, 4.2 Hz, 0.3H), 4.38 (dt, *J* = 10.5, 5.4 Hz, 1.2H), 4.25 (d, *J* = 5.2 Hz, 0.8H), 4.20–4.05 (m, 3.3H), 4.03–3.87 (m, 3.3H), 3.44 (s, 0.4H), 3.36 (d, *J* = 5.9 Hz, 0.4H), 2.92 (p, *J* = 6.8 Hz, 0.8H), 2.81 (p, *J* = 6.9 Hz, 0.2H), 2.69 (p, *J* = 6.8 Hz, 0.4H), 1.78–1.59 (m, 2H), 1.25–1.14 (m, 24H), 1.13–1.09 (m, 3H), 1.08–0.84 (m, 54H), 0.79 (t, *J* = 7.1 Hz, 4H) ppm. ^{13}C NMR (151 MHz, $CDCl_3$, mixture of the target compound and the starting material) δ 180.13, 179.64, 179.13, 156.03, 155.80, 155.72, 148.50, 148.38, 148.02, 147.97, 147.58, 147.45, 138.31, 137.95, 136.56, 136.43, 136.37, 133.94, 133.27, 128.57, 128.51, 128.45, 128.07, 124.52, 124.00, 121.45, 121.35, 121.23, 93.62, 91.30, 89.08, 88.14, 87.38, 81.66, 81.25, 81.07, 79.04, 76.60, 75.30, 74.74, 72.28, 70.63, 70.03, 69.50, 69.37, 60.55, 59.57, 59.50, 53.55, 50.39, 37.93, 37.87, 36.45, 36.24, 36.05, 31.92, 29.68, 29.65, 29.62, 29.59, 29.55, 29.51, 29.49, 29.46, 29.43, 29.37, 29.33, 26.54, 25.81, 25.64, 22.69, 19.21, 19.08, 18.97, 18.95, 18.90, 17.49, 17.46, 17.40, 17.33, 17.26, 17.20, 17.17, 17.11, 17.08, 17.05, 17.00, 16.98, 16.96, 16.88, 16.84, 14.12, 13.44, 13.39, 13.08, 13.00, 12.93, 12.67, 12.54. ppm. ESI-TOF-MS $C_{45}H_{74}N_6NaO_{10}Si_2$ $(M + Na)^+$ calcd. *m/z* 937.4897, found *m/z* 937.4775.

***N*²-Isobutyryl-2'-(*n*-Undecyl)Nb-guanosine (S50):** A solution of the protected guanosine **S49** (2.53 g, 2.76 mmol) in tetrahydrofuran (13.8 mL) was cooled in an ice-bath and 1 M TBAF/THF (27.6 mL, 27.6 mmol) was added to the solution. After being stirred for 4 hours at 0 °C, the reaction mixture was diluted with dichloromethane (120 mL) and washed with water (120 mL). The aqueous layer was extracted with dichloromethane (120 mL). The combined organic layer was dried over Na_2SO_4 and concentrated. The residue was purified by silica gel column chromatography eluted by 0–9.1% methanol/dichloromethane, to afford compound **S50** (1.00 g, 53.7% yield) as white foam. ^1H NMR (600 MHz, $CDCl_3$) δ 12.15 (d, *J* = 17.5 Hz, 1H), 10.71–10.47 (m, 1H), 7.81 (s, 0.5H), 7.69 (d, *J* = 8.0 Hz, 0.5H), 7.62 (dd, *J* = 8.2, 1.4 Hz, 0.5H), 7.49–7.42 (m, 2H), 7.28–7.19 (m, 1H), 7.16–7.10 (m, 0.5H), 5.71 (d, *J* = 3.2 Hz, 0.5H), 5.30 (d, *J* = 2.9 Hz, 0.5H), 5.10 (t, *J* = 6.4 Hz, 0.5H), 5.02–4.92 (m, 1.5H), 4.82 (d, *J* = 6.8 Hz, 0.5H), 4.76 (d, *J* = 6.9 Hz, 0.5H), 4.72 (d, *J* = 7.1 Hz, 0.5H), 4.60 (t, *J* = 4.2 Hz, 0.5H), 4.50 (dd, *J* = 4.9, 3.0 Hz, 0.5H), 4.44 (d, *J* = 7.2 Hz, 0.5H), 4.23 (s, 1H), 3.92 (dt, *J* = 6.5, 3.3 Hz, 0.5H), 3.87 (dt, *J* = 7.0, 3.5 Hz, 0.5H), 3.78–3.59 (m, 2H), 3.27 (d, *J* =

5.5 Hz, 0.5H), 3.24–3.17 (m, 6.5H), 3.05–2.93 (m, 1H), 1.56 (dq, *J* = 16.3, 7.9 Hz, 8H), 1.50–1.39 (m, 1.5H), 1.30 (h, *J* = 6.9 Hz, 7H), 1.17–1.01 (m, 23H), 1.01–0.92 (m, 3H), 0.92–0.88 (m, 1H), 0.85 (td, *J* = 7.4, 1.3 Hz, 10H), 0.71 (td, *J* = 7.1, 1.4 Hz, 3H) ppm. ^{13}C NMR (151 MHz, $CDCl_3$) *δ* 180.47, 180.28, 155.49, 155.47, 148.15, 147.87, 147.77, 147.73, 147.55, 147.51, 147.10, 138.97, 138.76, 138.44, 137.71, 133.25, 132.77, 128.26, 128.18, 127.97, 127.74, 123.89, 123.56, 121.42, 121.21, 94.72, 93.07, 88.61, 88.47, 84.22, 83.86, 78.84, 78.60, 74.87, 73.51, 70.30, 69.29, 69.11, 61.01, 60.87, 58.63, 37.49, 37.37, 35.53, 35.42, 31.59, 29.31, 29.29, 29.17, 29.08, 29.01, 26.22, 25.41, 25.26, 23.76, 22.37, 19.46, 18.97, 18.94, 18.83, 18.79, 13.84, 13.40 ppm. ESI-TOF-MS $C_{33}H_{48}N_6NaO_9$ (M + Na)$^+$ calcd. *m/z* 695.3375, found *m/z* 695.3354.

2'-(*n*-Undecyl)Nb-guanosine (S51): To a solution of the protected guanosine **S50** (970 mg, 1.44 mmol) in acetonitrile (9.40 mL) was added 28% ammonium hydroxide solution (18.8 mL). The mixture was heated to 55 ºC. After being stirred at 55 ºC for 19.5 hours, the reaction mixture was concentrated and azeotroped with benzene (x1). The residue was dissolved in methanol (6.00 mL) and suspended by the addition of diethyl ether (200 mL). The resulting precipitate was collected by filtration and rinsed with diethyl ether. The obtained solid was dried under vacuum to afford compound **S51** (408 mg, 47.0% yield) as white solid. ^{1}H NMR (600 MHz, DMSO-d_6) *δ* 10.23 (s, 0.5H), 7.93 (s, 0.5H), 7.88 (dd, *J* = 8.2, 1.3 Hz, 0.5H), 7.84 (dt, *J* = 8.1, 0.9 Hz, 0.5H), 7.78 (s, 0.5H), 7.70 (td, *J* = 7.6, 1.4 Hz, 0.5H), 7.57 (dd, *J* = 7.9, 1.5 Hz, 0.5H), 7.55–7.50 (m, 1.5H), 7.40 (ddd, *J* = 8.1, 5.2, 3.6 Hz, 0.5H), 6.43 (s, 1.5H), 5.82 (d, *J* = 6.3 Hz, 0.5H), 5.68 (d, *J* = 5.9 Hz, 0.5H), 5.23 (d, *J* = 5.3 Hz, 0.5H), 5.16 (d, *J* = 5.0 Hz, 0.5H), 5.11 (t, *J* = 5.5 Hz, 0.5H), 5.08 (t, *J* = 5.4 Hz, 0.5H), 5.04 (dd, *J* = 7.8, 4.4 Hz, 0.5H), 4.78 (dd, *J* = 7.6, 4.9 Hz, 0.5H), 4.69–4.62 (m, 1.5H), 4.57 (d, *J* = 7.0 Hz, 0.5H), 4.52–4.47 (m, 0.5H), 4.34 (d, *J* = 7.3 Hz, 0.5H), 4.22 (dtd, *J* = 16.7, 5.0, 3.4 Hz, 1H), 3.89 (q, *J* = 3.7 Hz, 0.5H), 3.85 (q, *J* = 3.7 Hz, 0.5H), 3.61 (tdd, *J* = 12.5, 5.1, 3.6 Hz, 1H), 3.51 (dtd, *J* = 12.0, 6.3, 3.9 Hz, 1H), 3.20–3.14 (m, 2H), 1.60–1.53 (m, 2H), 1.31 (p, *J* = 7.4 Hz, 2H), 1.28–1.15 (m, 13H), 0.93 (t, *J* = 7.4 Hz, 3H), 0.85 (td, *J* = 7.1, 0.9 Hz, 3H) ppm. ^{13}C NMR (151 MHz, DMSO-d_6) *δ* 156.71, 153.69, 153.64, 151.13, 151.02, 148.51, 147.69, 137.19, 136.43, 135.59, 135.17, 133.42, 133.24, 128.74, 128.34, 128.10, 123.97, 123.91, 116.89, 116.67, 93.20, 91.53, 85.92, 85.60, 85.05, 84.77, 78.82, 77.97, 74.07, 73.01, 69.60, 69.09, 61.49, 61.27, 57.54, 37.09, 36.76, 31.31, 31.28, 29.01, 28.96, 28.93, 28.83, 28.72, 28.69, 28.63, 24.98, 24.92, 23.06, 22.10, 19.21, 13.96, 13.49 ppm. ESI-TOF-MS $C_{29}H_{42}N_6NaO_8$ (M + Na)$^+$ calcd. *m/z* 625.2956, found *m/z* 625.2948.

2'-(*n*-Undecyl)Nb-*N*^{7}-methylguanosine (S52): To a solution of 2'-modified guanosine **S51**

(100 mg, 0.166 mmol) in DMF (1.33 mL) was added iodomethane (186 mg, 81.6 μL, 1.31 mmol). After being stirred at room temperature for 12.5 hours, to the reaction mixture was added water (15.0 mL) and extracted 4 times with dichloromethane (15.0 mL). The organic layer was concentrated, and the residue was dissolved in acetone (2.00 mL). The solution was added dropwise to ether (150 mL), and the resulting precipitate was collected by filtration. The solid was dried under reduced pressure to give compound **S52** (107 mg, 75.4% yield) as yellow solid. ^{1}H NMR (600 MHz,$CDCl_3$) *δ* 9.56 (d, *J* = 7.3 Hz, 0.5H), 9.45 (d, *J* = 7.6 Hz, 0.5H), 7.98 (d, *J* = 7.1 Hz, 0.2H), 7.82 (q, *J* = 8.7 Hz, 0.5H), 7.75 (t, *J* = 7.8 Hz, 0.5H), 7.64 (dq, *J* = 10.5, 5.5 Hz, 2H), 7.40 (dtd, *J* = 14.8, 8.7, 5.4 Hz, 0.8H), 7.31–7.23 (m, 0.5H), 6.72 (s, 1H), 6.04 (q, *J* = 3.6 Hz, 0.5H), 5.89 (q, *J* = 3.4 Hz, 0.5H), 5.18–5.12 (m, 0.5H), 5.06 (q, *J* = 6.6 Hz, 0.5H), 4.96 (t, *J* = 7.0 Hz, 0.5H), 4.89 (t, *J* = 7.1 Hz, 0.5H), 4.78 (t, *J* = 7.1 Hz, 0.5H), 4.73 (t, *J* = 7.0 Hz, 0.5H), 4.56 (qt, *J* = 8.5, 4.8 Hz, 1H), 4.45 (dt, *J* = 10.2, 5.0 Hz, 0.5H), 4.23–4.11 (m, 3H), 4.00–3.91 (m, 1H), 3.79 (dt, *J* = 12.7, 5.9 Hz, 1H), 3.28 (dd, *J* = 10.8, 6.2 Hz, 1.5H), 3.02–2.81 (m, 1H), 1.76–1.62 (m, 4H), 1.44 (hept, *J* = 7.4 Hz, 2H), 1.27–1.13 (m, 14H), 1.00 (q, *J* = 7.4 Hz, 2.5H), 0.82 (q, *J* = 7.1 Hz, 2.5H) ppm. ^{13}C NMR (151 MHz, $CDCl_3$) *δ* 162.91, 156.24, 155.98, 154.16, 153.87, 148.90, 148.65, 148.53, 147.91, 137.62, 137.37, 136.28, 136.15, 133.73, 133.56, 128.68, 128.60, 128.45, 124.30, 124.02, 108.76, 108.55, 94.32, 94.12, 88.95, 86.85, 86.38, 80.38, 80.27, 75.20, 74.77, 69.30, 68.87, 60.50, 60.23, 59.26, 50.06, 49.91, 49.77, 49.63, 49.48, 37.63, 37.55, 37.04, 36.97, 36.74, 31.98, 29.73, 29.58, 29.54, 29.41, 25.70, 25.43, 24.29, 22.75, 19.88, 14.19, 13.80 ppm. ESI-TOF-MS $C_{30}H_{43}N_6O_8$ (M – I^-– 2H)$^-$ calcd. *m/z* 615.3148, found *m/z* 615.3225.

<u>2’-(*n*-Undecyl)Nb-*N*^{7}-methylguanosine 5’-monophosphate (S53):</u> A suspension of 2’-C11Nb-*N*^{7}-methylguanosine **S52** (53.2 mg, 62.3 μmol) in trimethyl phosphate (312 μL) was cooled to –10 ºC. 2,6-Lutidine (15.3 mg, 16.7 μL, 143 μmol) and phosphoryl chloride (21.9 mg, 13.4 μL, 143 μmol) were successively added to the mixture. After being stirred at –10 ºC for 9 hours, the reaction mixture quenched by the addition of 0.2 M TEAB buffer (pH 7.9, 500 μL) at –10 ºC. After warming to room temperature, the mixture was diluted with methanol (9.00 mL). The mixture was further diluted with 50% methanol/water (~50mL) and purified by using CHRMATREX C8 MB-100-40/75 column (Column size: φ = 3.0 cm, *h* = 10 cm, 70 cm^3 (7 mL/min), Solvent A: 50 mM TEAA buffer (pH 6.0) contains 0.5% CH_3CN, Solvent B: CH_3CN, Linear gradient 0–90%B over 60 minutes). The fractions containing the target product were combined, concentrated, and lyophilized, to afford compound **S53** (37.3 mg, 85.9% yield) as white solid. ^{1}H NMR (600MHz, CD_3OD) *δ* 7.88–7.79 (m, 1H), 7.74–7.62 (m, 2H), 7.49–7.41 (m, 1H), 6.12 (d, *J* = 3.5 Hz, 0.5H), 5.96 (d, *J* = 3.5 Hz, 0.5H), 5.22 (dd, *J* = 7.6, 4.5 Hz, 0.5H), 5.12 (dd, *J* = 7.6, 4.8 Hz, 0.5H), 5.01 (d, *J* = 7.1 Hz, 0.5H), 4.77 (d, *J* =

7.1 Hz, 0.5H), 4.70 (d, *J* = 7.0 Hz, 0.5H), 4.61 (ddd, *J* = 16.9, 4.8, 3.4 Hz, 1H), 4.47 (t, *J* = 5.1 Hz, 0.5H), 4.41–4.34 (m, 0.5H), 4.24–4.16 (m, 1.5H), 4.13 (d, *J* = 4.6 Hz, 2H), 4.06 (d, *J* = 1.4 Hz, 1H), 4.04 (dd, *J* = 4.7, 1.7 Hz, 0.5H), 4.02 (dt, *J* = 4.7, 1.4 Hz, 0.5H), 3.24–3.19 (m, 3H), 3.16 (q, *J* = 7.4 Hz, 16H, triethylammonium), 1.91 (s, 10H, acetate), 1.67–1.61 (m, 4H), 1.39 (h, *J* = 7.4 Hz, 4H), 1.28 (t, *J* = 7.4 Hz, 25H, triethylammonium), 1.22 (d, *J* = 13.0 Hz, 14H), 1.00 (t, *J* = 7.4 Hz, 5H), 0.86 (dd, *J* = 7.6, 6.5 Hz, 3H) ppm. ^{13}C NMR (151 MHz, CD_3OD) *δ* 178.42 (acetate), 158.23, 158.05, 156.05, 155.91, 151.05, 150.87, 150.18, 149.47, 139.09, 138.56, 138.40, 134.49, 134.37, 129.79, 129.73, 129.67, 129.62, 129.37, 125.14, 125.06, 109.30, 109.10, 94.72, 94.62, 89.68, 89.63, 86.64, 86.40, 86.35, 81.47, 81.33, 80.84, 79.51, 79.29, 79.07, 75.88, 75.39, 69.89, 69.76, 64.26, 64.08, 59.49, 47.48, 38.63, 38.54, 36.70, 36.64, 33.04, 30.73, 30.70, 30.68, 30.64, 30.54, 30.49, 30.44, 26.64, 26.51, 24.76, 23.71, 22.96, 20.70, 14.45, 13.93, 9.10 ppm. ^{31}P NMR (243 MHz, CD_3OD) *δ* 1.65 ppm. ESI-TOF-MS $C_{30}H_{44}N_6O_{11}P$ $(M-H)^-$ calcd. *m/z* 695.2811, found *m/z* 695.2828.

2'-(*n*-Undecyl)Nb-Di-PureCap Analog (25): To a solution of 2'-Nb-*N*^{7}-methylguanosine 5'-monophosphate **S53** (37.3 mg, 53.5 μmol) in DMSO (1.07 mL) was added guanosine 5'-diphosphate imidazolide (**S24**) (115 mg, 214 μmol) and zinc chloride (146 mg, 1.07 mmol). The mixture was incubated at 37 ºC for 2 days. The reaction mixture was quenched by the addition of 500 mM *aq.* EDTA (pH 8.0, 2.78 mL, EDTA: 1.39 mmol) and diluted with water (10.0 mL). The crude was purified by using CHRMATREX C8 MB-100-40/75 column (Column size: φ = 3.0 cm, *h* = 10 cm, 70 cm^3 (7 mL/min), Solvent A: 50 mM TEAA buffer (pH 6.0) contains 0.5% CH_3CN, Solvent B: CH_3CN, Linear gradient 0–90%B over 60 minutes). The fractions containing the target product were combined, concentrated, and lyophilized, to afford the target compound as triethylammonium form. The cap analog triethylammonium salt was dissolved in methanol (1.00 mL). A solution of 0.19 M $NaClO_4$ in acetone (12.0 mL) was added to the methanol solution of cap analog to give white precipitate. The suspension was centrifuged for 15 minutes at 4,500 rpm. The supernatant was discarded, and the precipitate was resuspended in acetone. The suspending-centrifugation processes were repeated additional 3 times. The precipitate was dried under reduced pressure to afford di-PureCap analog **25** (30.0 mg, 25.3 μmol, 47.2% yield) as sodium salt. The yield was calculated by using absorbance of the product at 260 nm measured by NanoDrop. Extinction coefficient (ε_{260}) = 26,300 M^{-1}•cm^{-1} was used for calculation (A. M. Rydzik, *et al., Nucleic Acids Res.*, **2017**, *45*(15), 8661–8675; S. G. Chaulk, *et al., Nat. Protocol.*, **2007**, *2*(5), 1052–1058). ^1H NMR (600 MHz, D_2O) *δ* 7.97 (d, *J* = 10.7 Hz, 1H), 7.70 (s, 1H), 7.64 – 7.40 (m, 2H), 7.29 (s, 1H), 6.06 (s, 0.5H), 5.94 – 5.75 (m, 1.5H), 5.16 (s, 0.5H), 5.07 (s, 1H), 4.98 (s, 1H), 4.68 (d, *J* = 10.4 Hz, 1.5H), 4.58 (s, 1.5H), 4.49 (s, 2H), 4.38 (s, 1.5H), 4.33 – 4.22 (m,

4H), 4.11 (s, 3H), 1.43 (s, 2H), 1.11 – 0.88 (m, 2H), 0.57 (s, 16H), 0.21 (s, 3H) ppm. ^{13}C NMR (151 MHz, D_2O) δ 158.68, 154.03, 151.26, 149.77, 147.75, 136.73, 128.26, 124.11, 115.80, 107.92, 100.39, 87.18, 83.38, 79.08, 74.54, 70.32, 65.31, 37.44, 36.25, 31.40, 30.28, 29.22, 28.86, 25.31, 23.31, 22.11, 13.46 ppm. ^{31}P NMR (243 MHz, D_2O) δ –10.50 (2P), –21.71 (1P) ppm. ESI-TOF-MS $C_{40}H_{56}N_{11}O_{21}P_3$ $(M-2H)^{2-}$ calcd. *m/z* 559.6438, found *m/z* 559.6413.

3. NMR Spectra of Synthesized Compounds

^{1}H and $^{13}C\{^{1}H\}$ NMR Spectra of Compound **12**

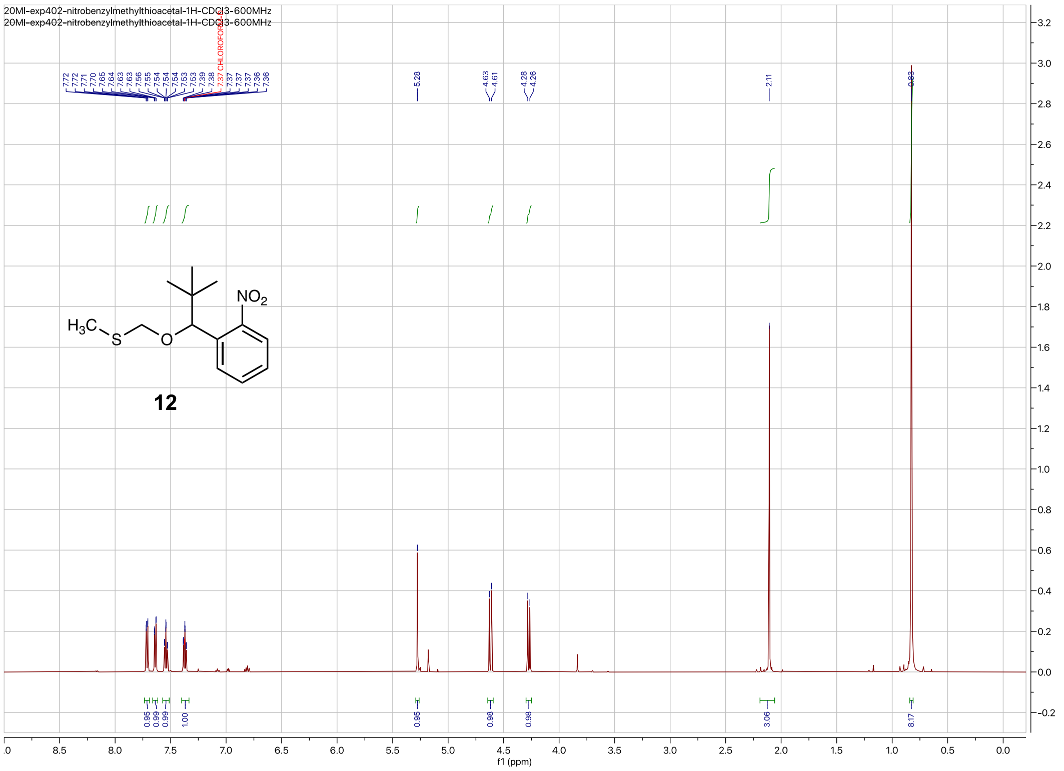

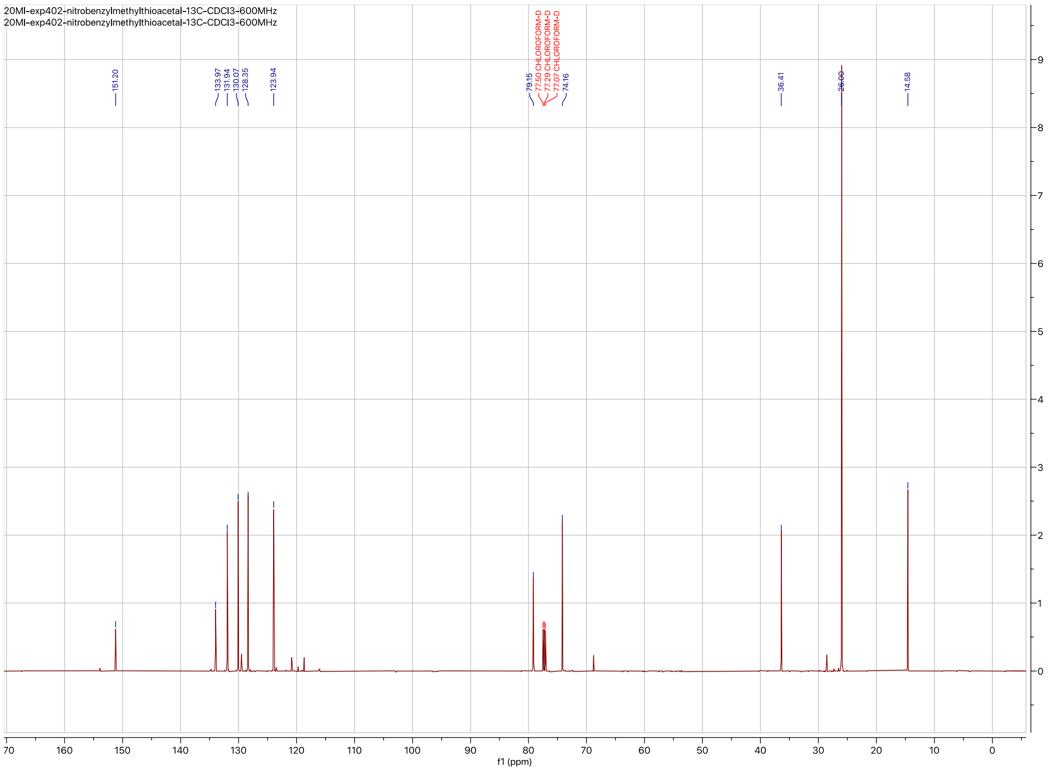

^{1}H and ^{13}C{^{1}H} NMR Spectra of Compound **S3**

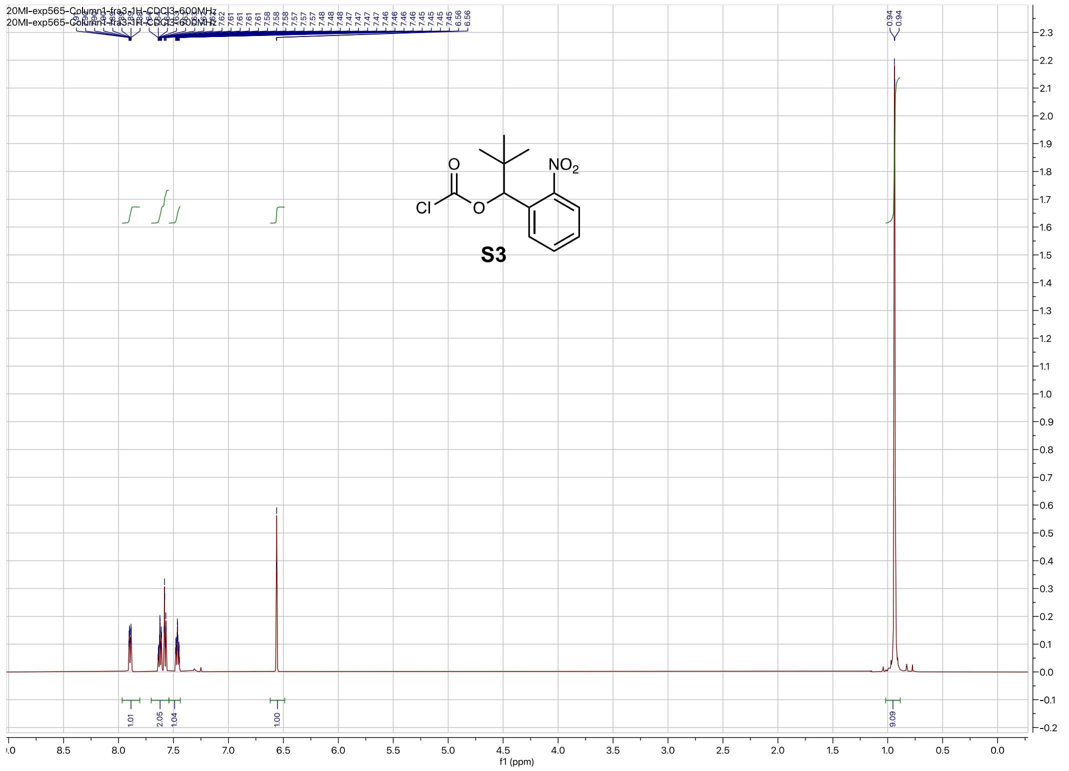

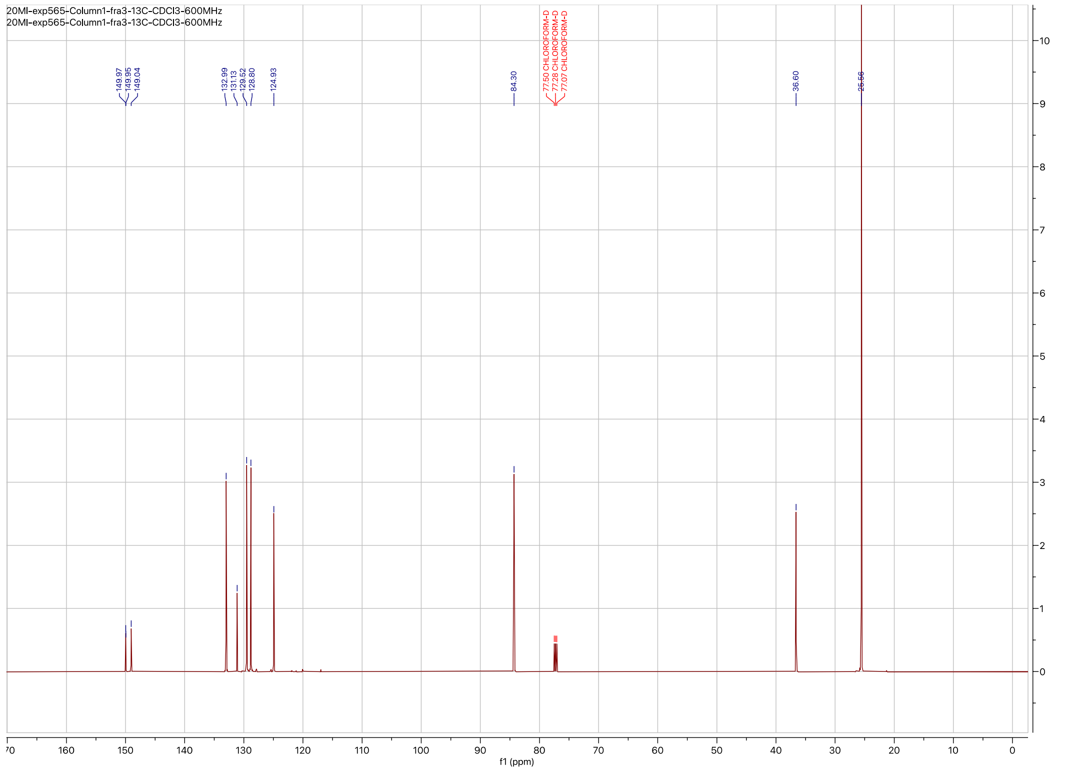

^{1}H and $^{13}C\{^{1}H\}$ NMR Spectra of Compound **S4**

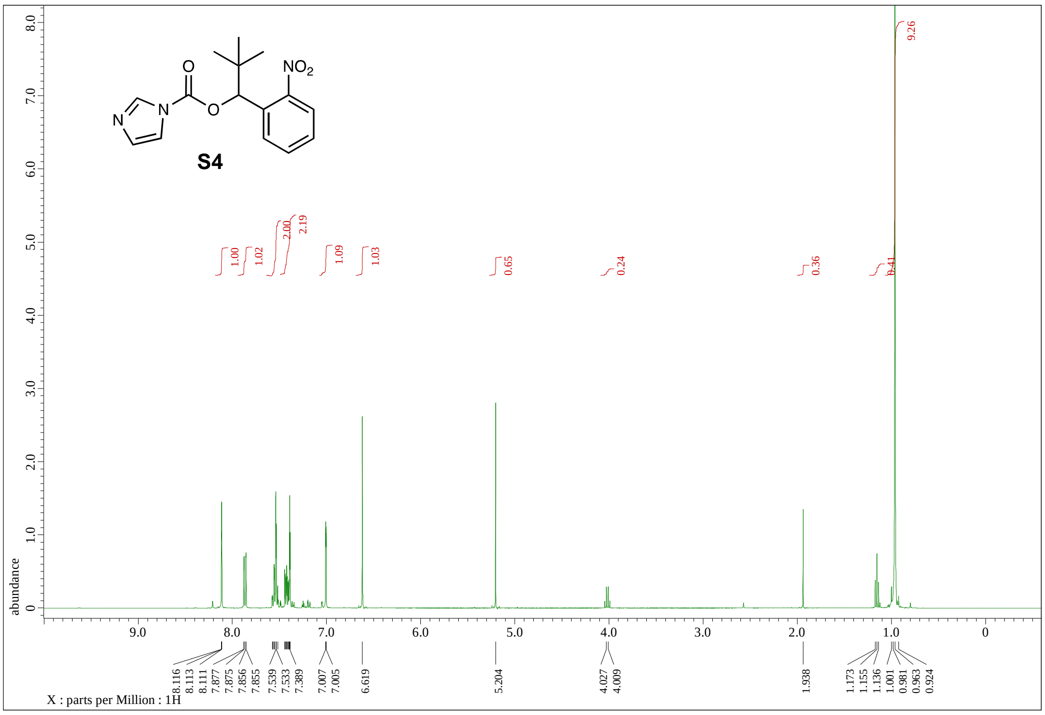

You created this PDF from an application that is not licensed to print to novaPDF printer (http://www.novapdf.com)

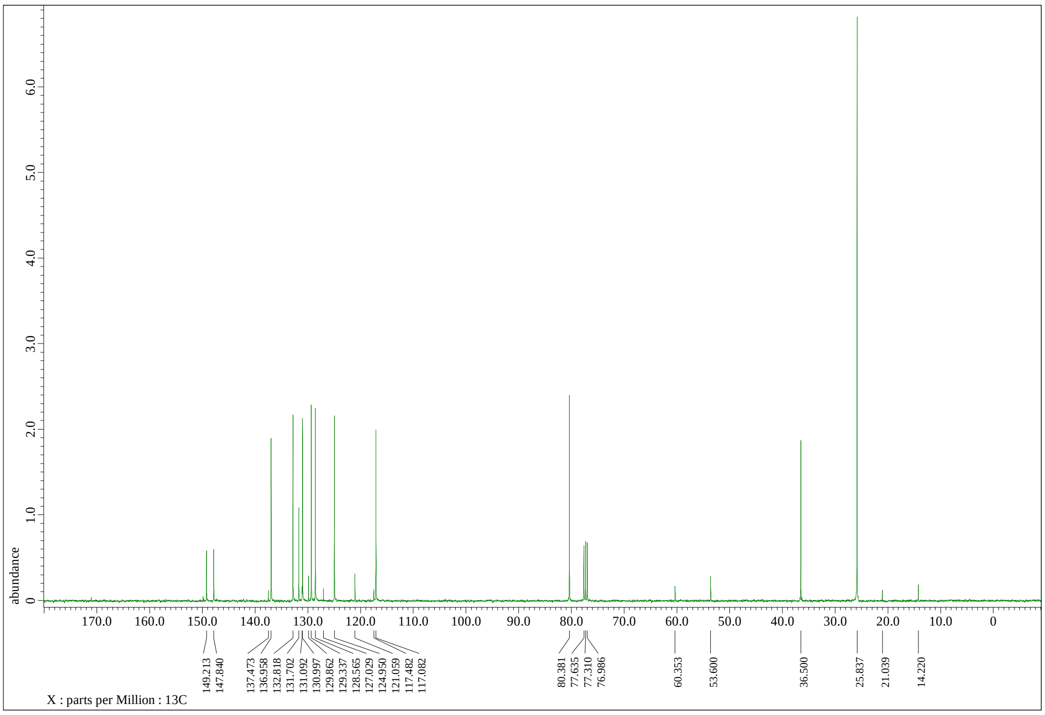

You created this PDF from an application that is not licensed to print to novaPDF printer (http://www.novapdf.com)

^{1}H and ^{13}C{^{1}H} NMR Spectra of Compound **13**

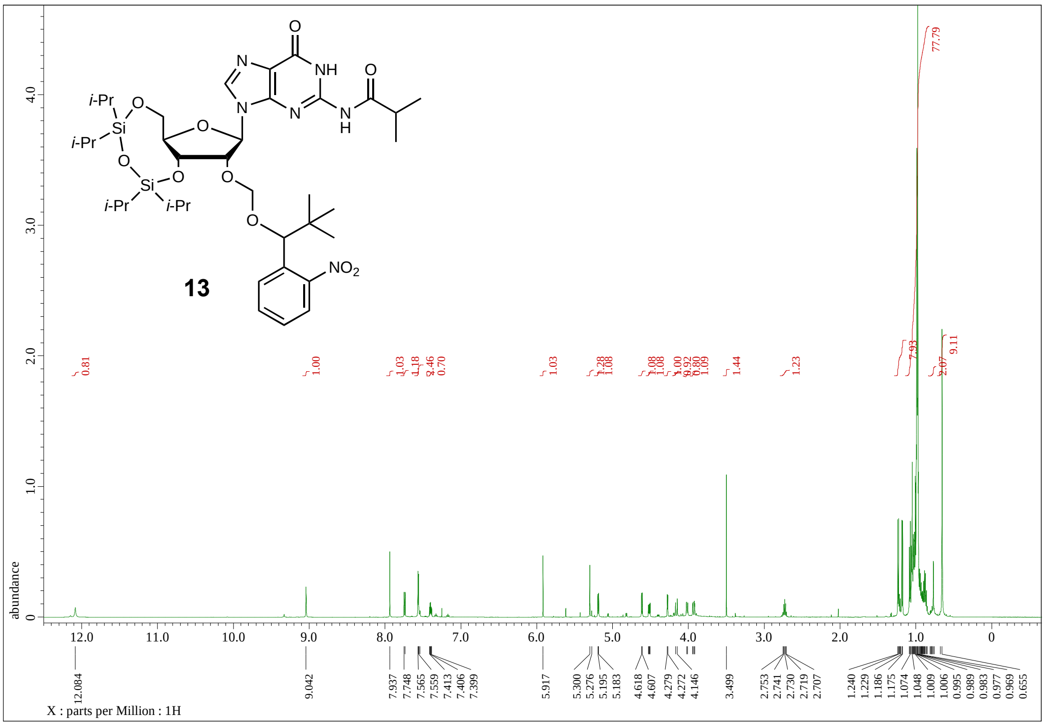

You created this PDF from an application that is not licensed to print to novaPDF printer (http://www.novapdf.com)

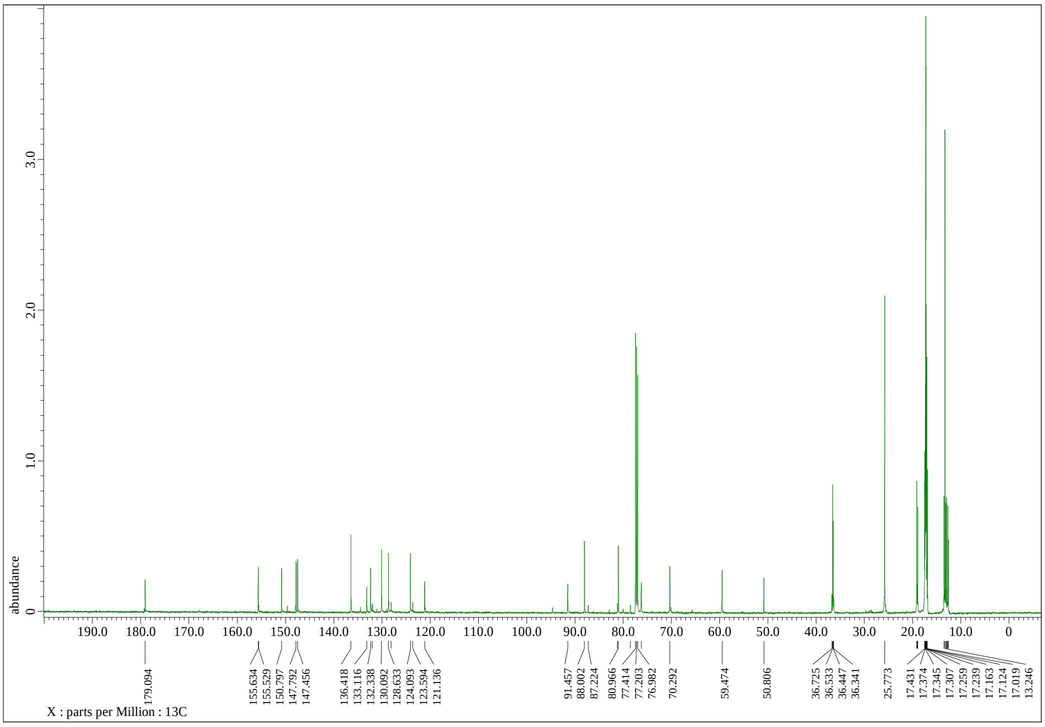

You created this PDF from an application that is not licensed to print to novaPDF printer (http://www.novapdf.com)

^1H and $^{13}C\{^1H\}$ NMR Spectra of Compound **S6**

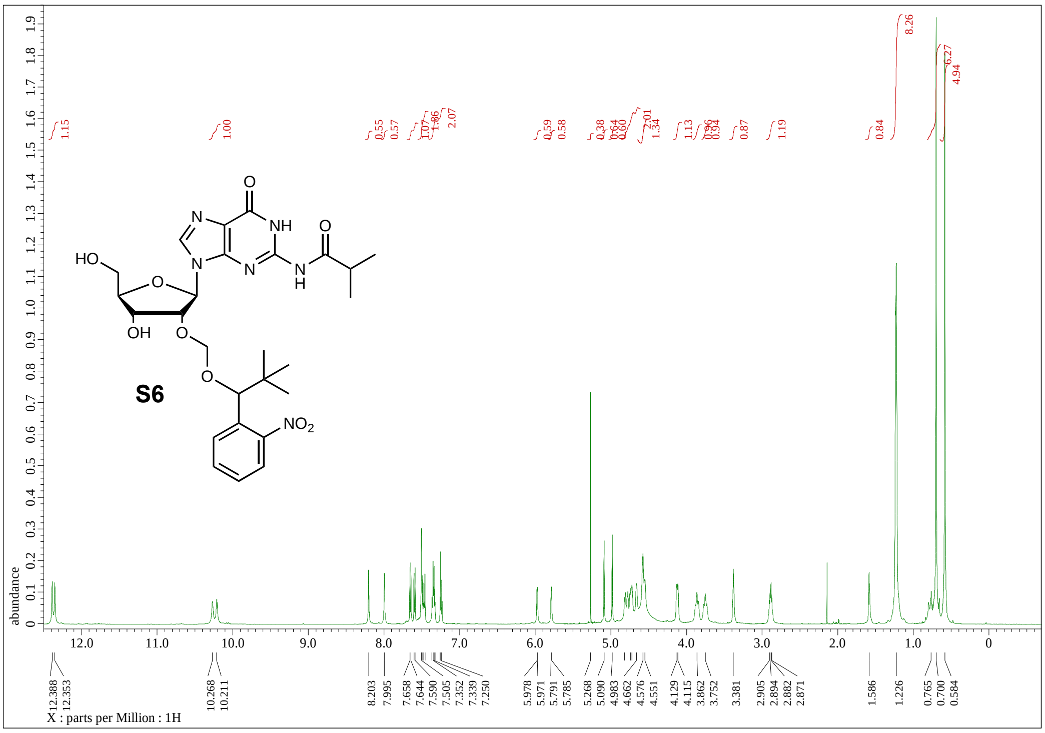

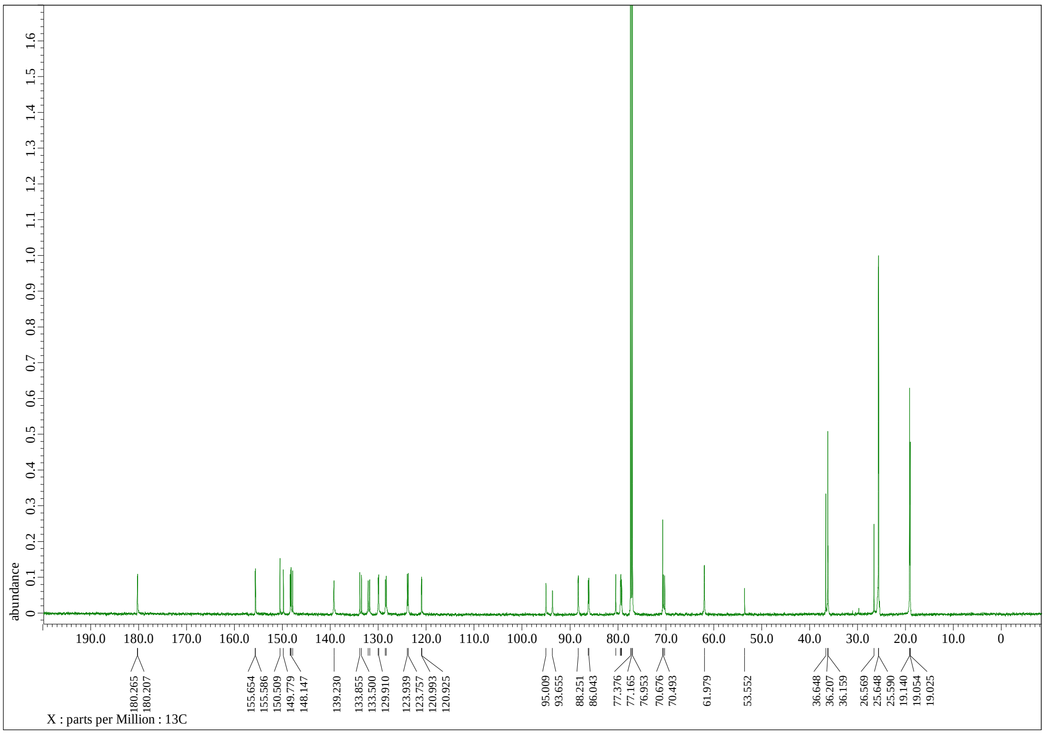

^{1}H and $^{13}C\{^{1}H\}$ NMR Spectra of Compound **14**

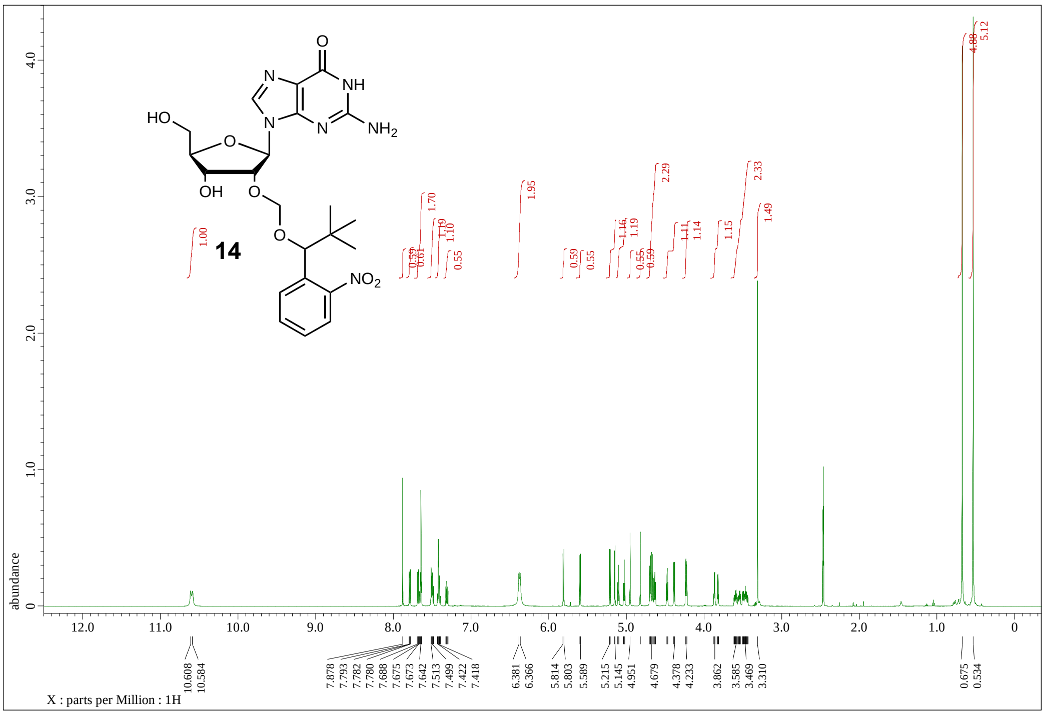

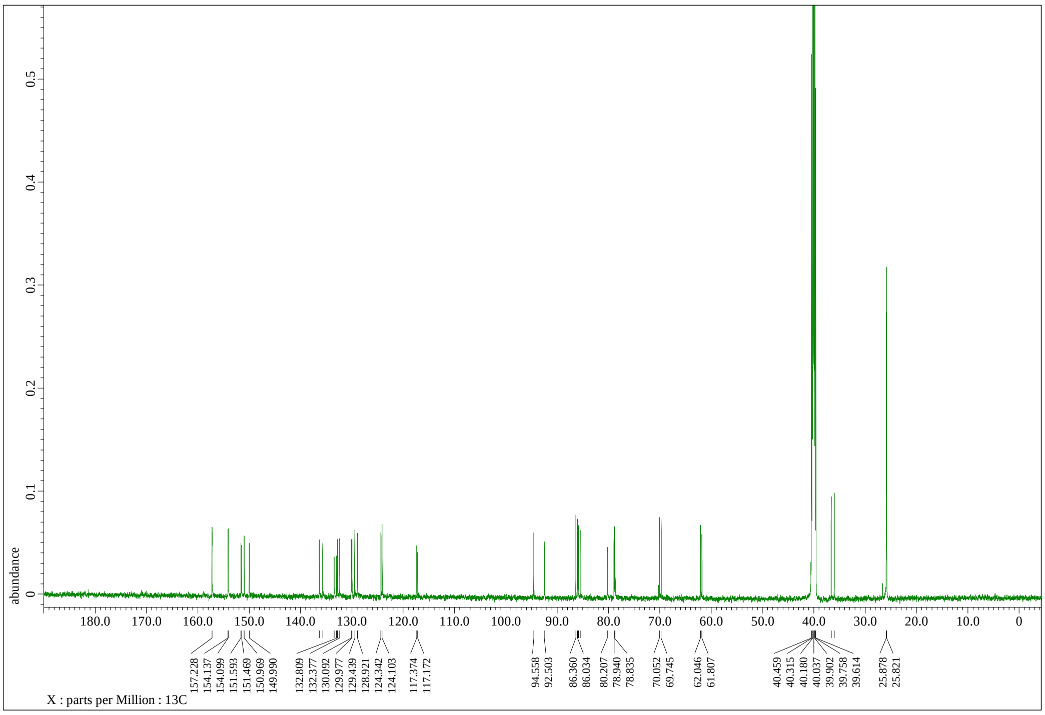

^{1}H and ^{13}C{^{1}H} NMR Spectra of Compound **15**

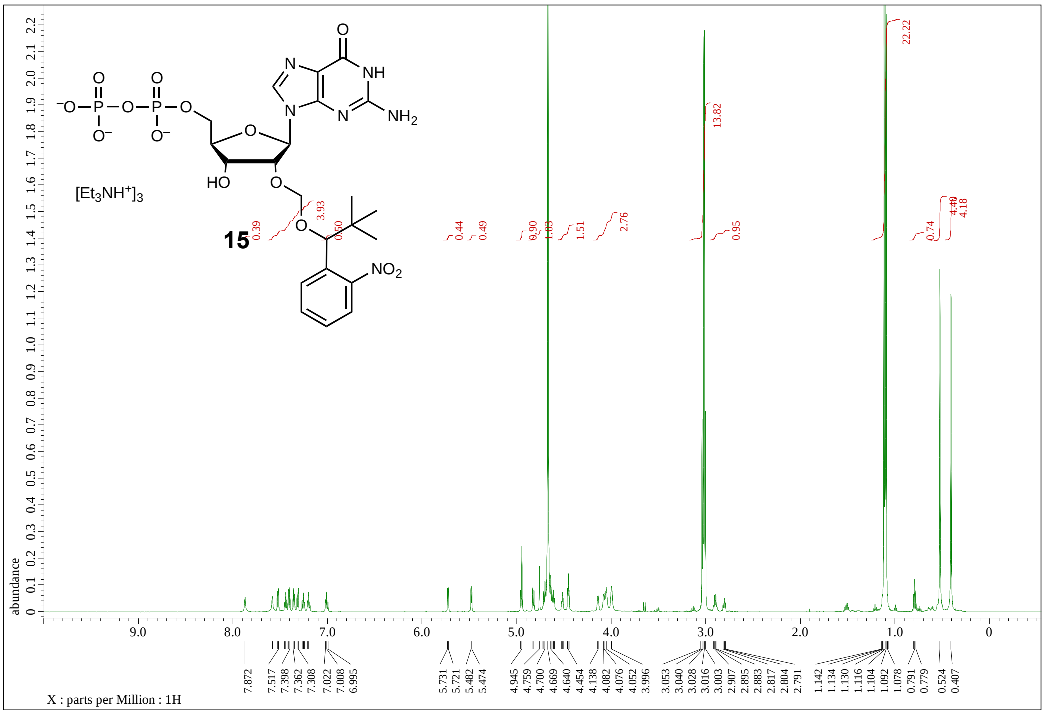

You created this PDF from an application that is not licensed to print to novaPDF printer (http://www.novapdf.com)

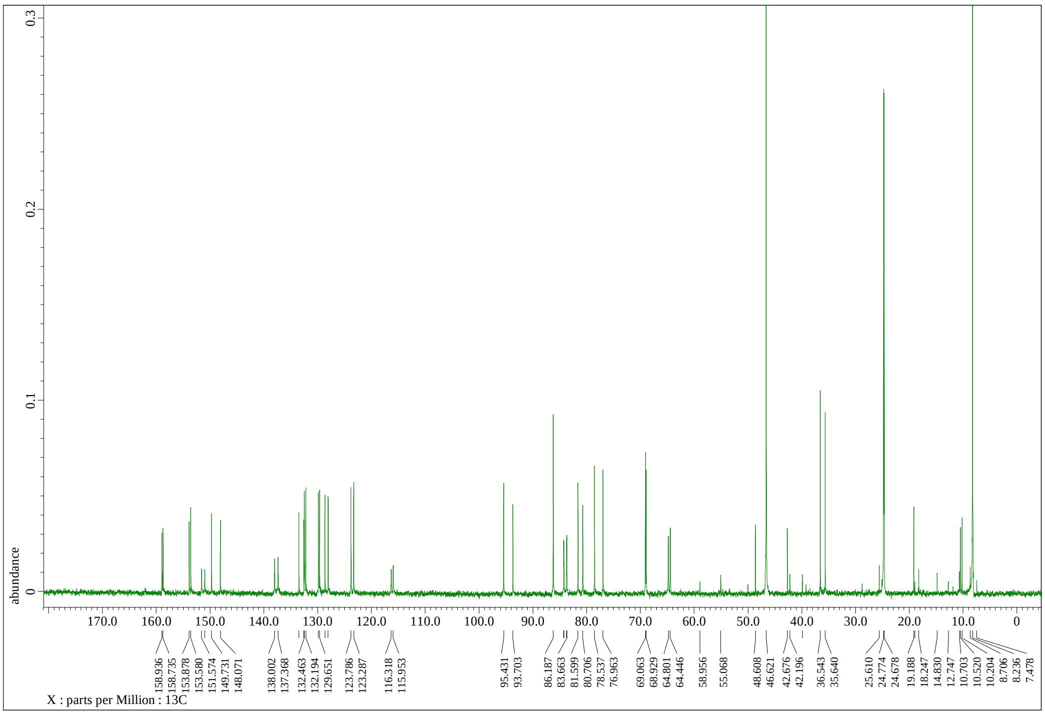

You created this PDF from an application that is not licensed to print to novaPDF printer (http://www.novapdf.com)

$^{31}P\{^1H\}$ NMR Spectrum of Compound **15**

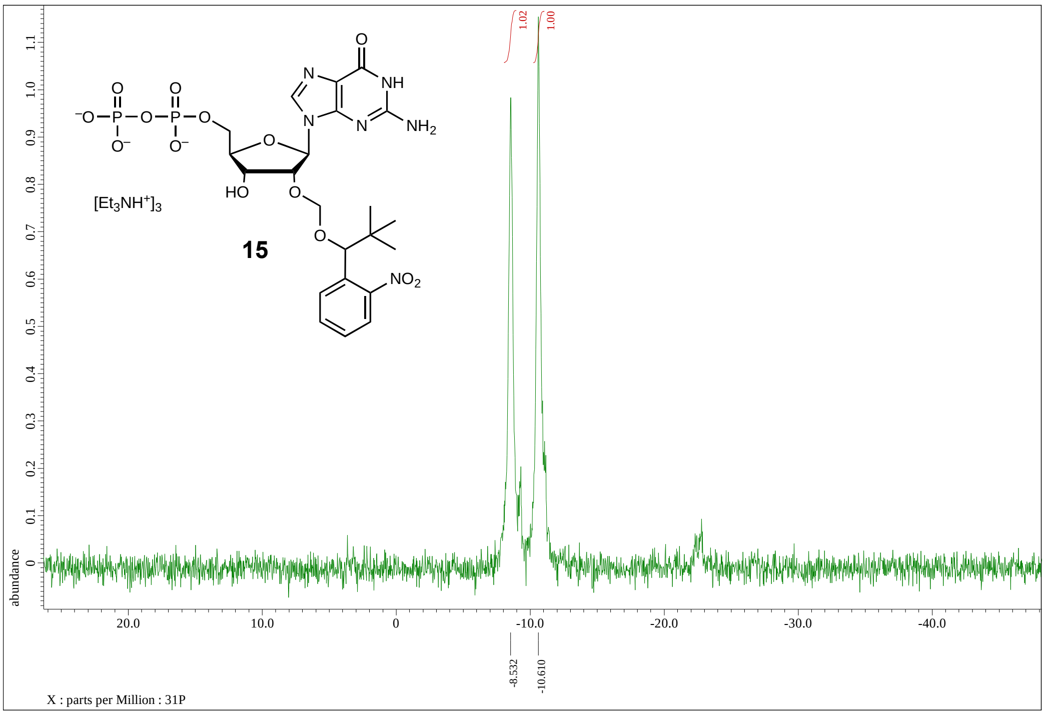

You created this PDF from an application that is not licensed to print to novaPDF printer (http://www.novapdf.com)

^{1}H and $^{13}C\{^{1}H\}$ NMR Spectra of Compound **17**

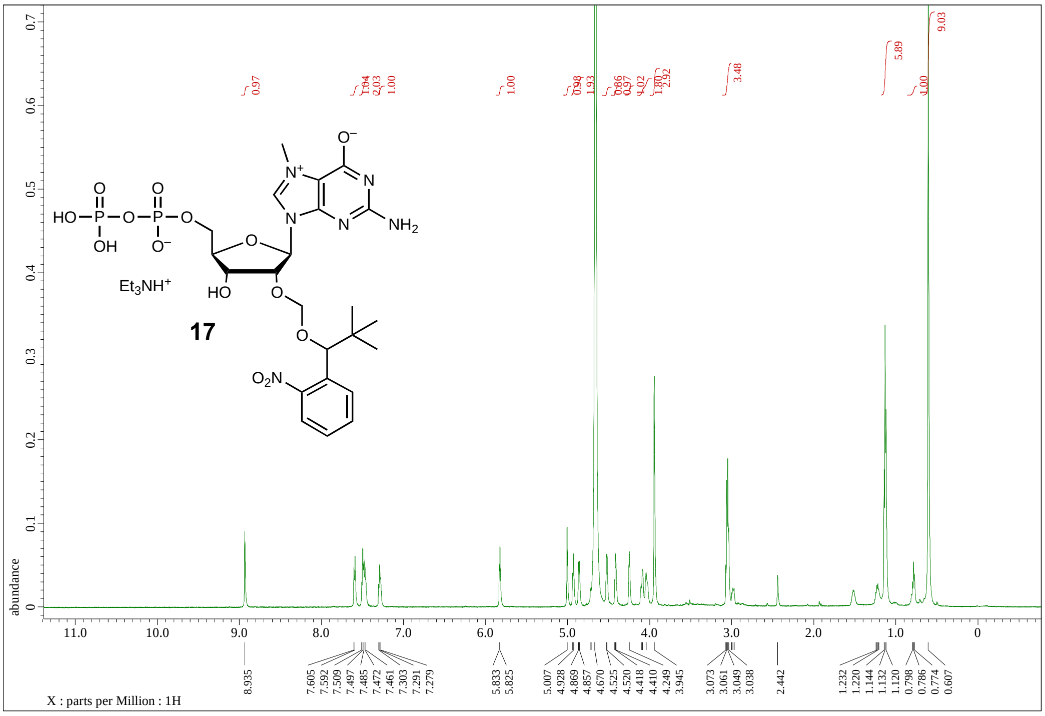

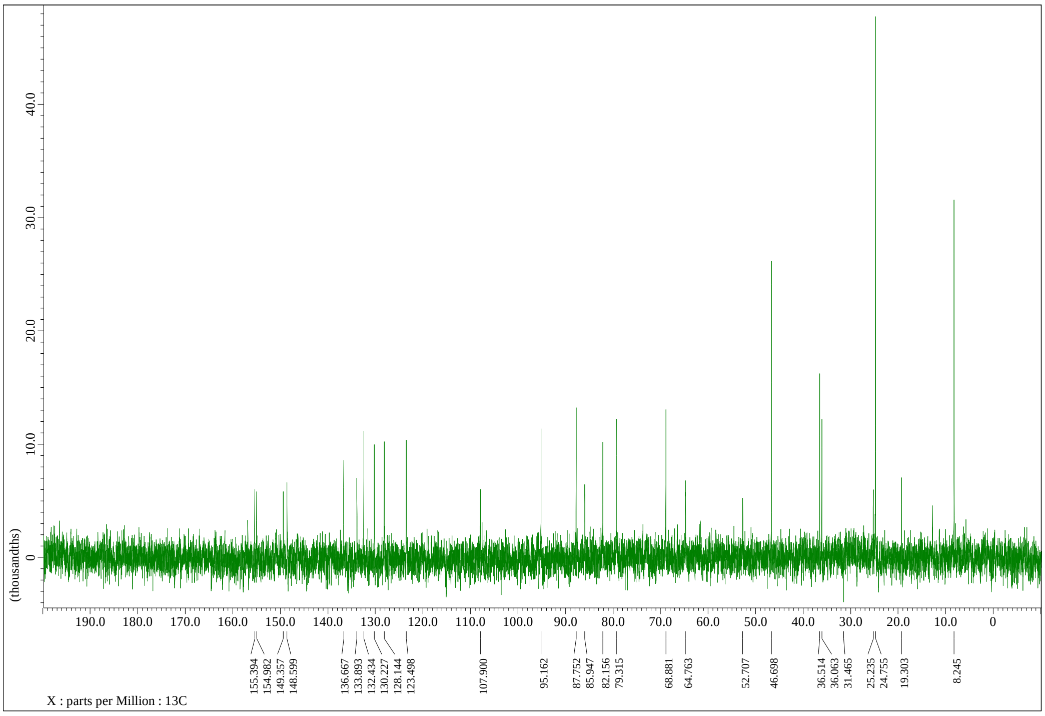

$^{31}P\{^{1}H\}$ NMR Spectrum of Compound **17**

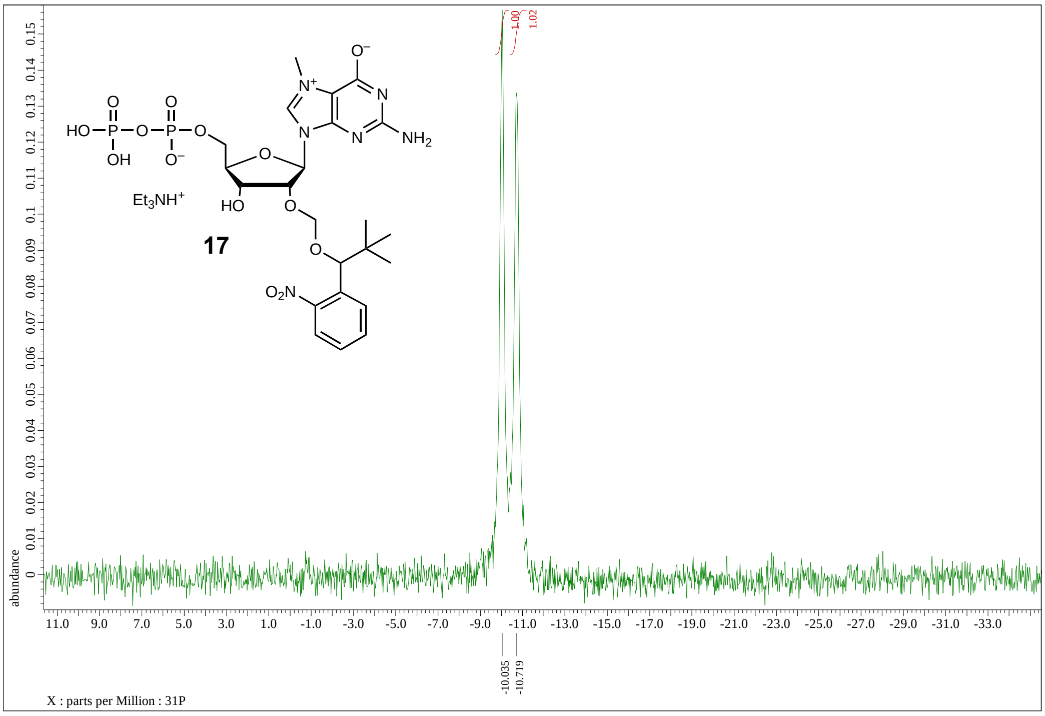

^{1}H and ^{13}C{^{1}H} NMR Spectra of Compound **1**

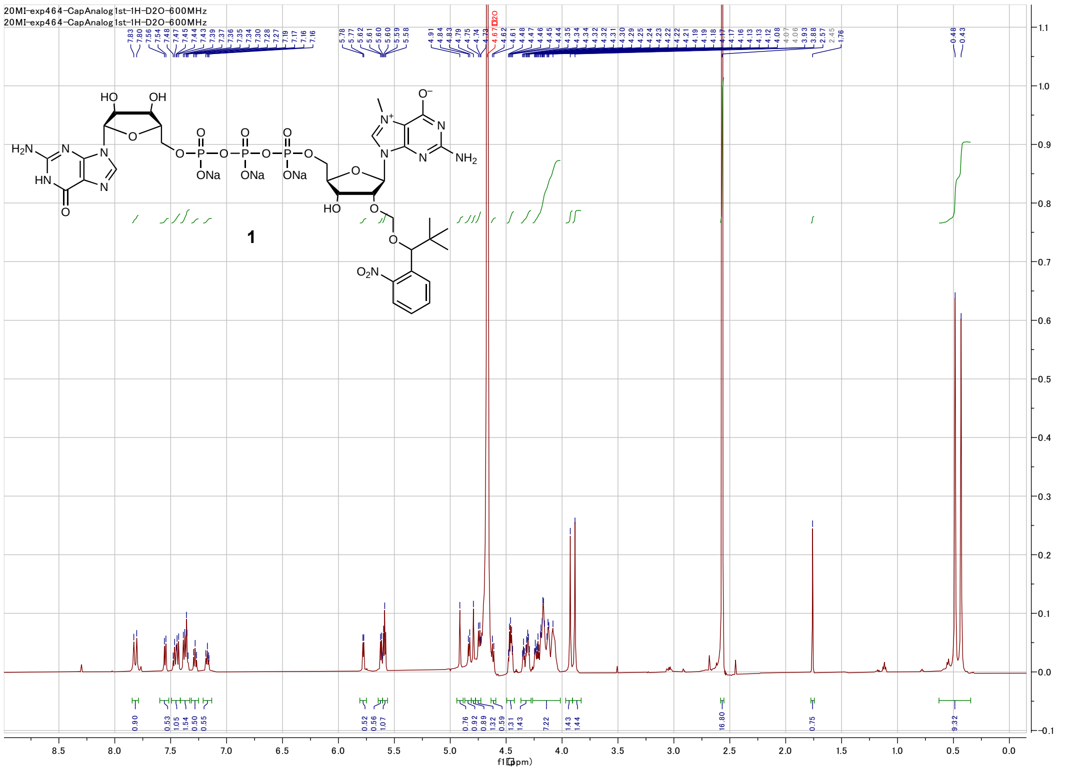

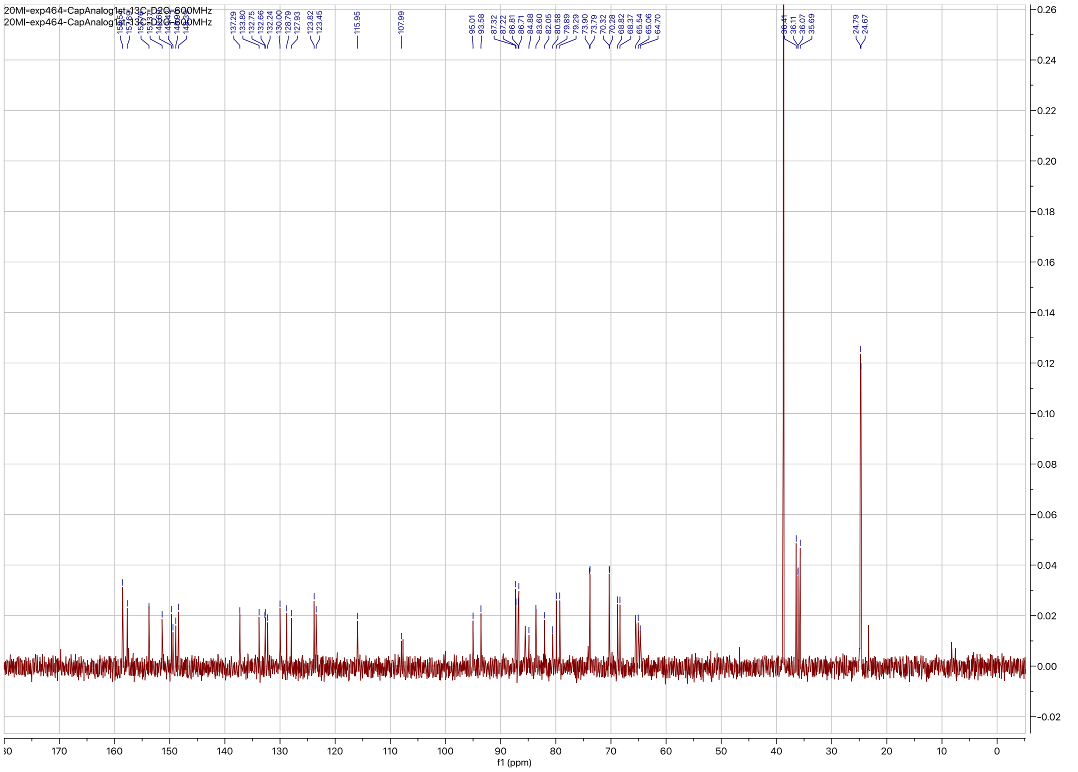

$^{31}P\{^1H\}$ NMR Spectrum of Compound **1**

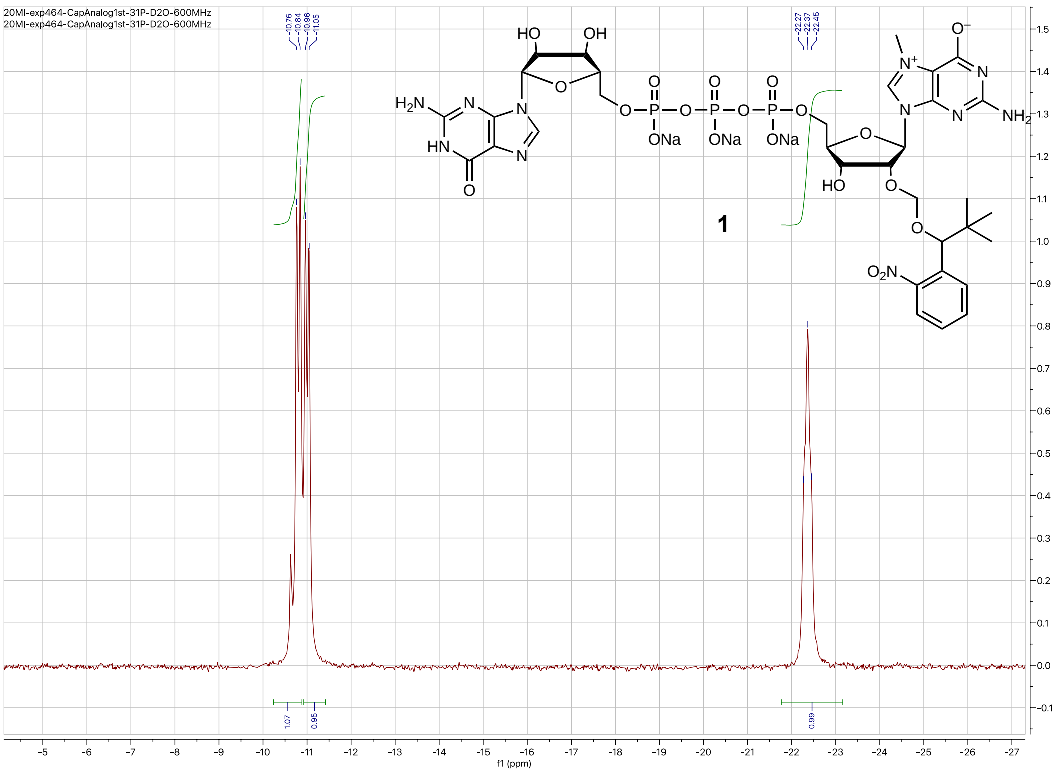

^{1}H and $^{13}C\{^{1}H\}$ NMR Spectra of Compound **S8**

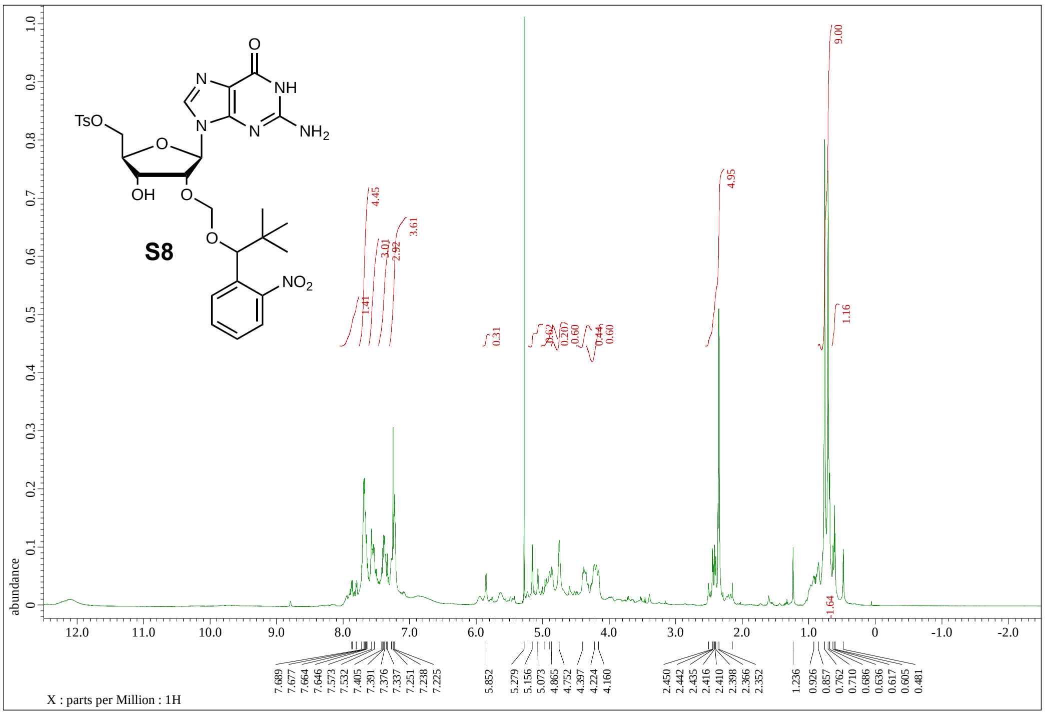

You created this PDF from an application that is not licensed to print to novaPDF printer (http://www.novapdf.com)

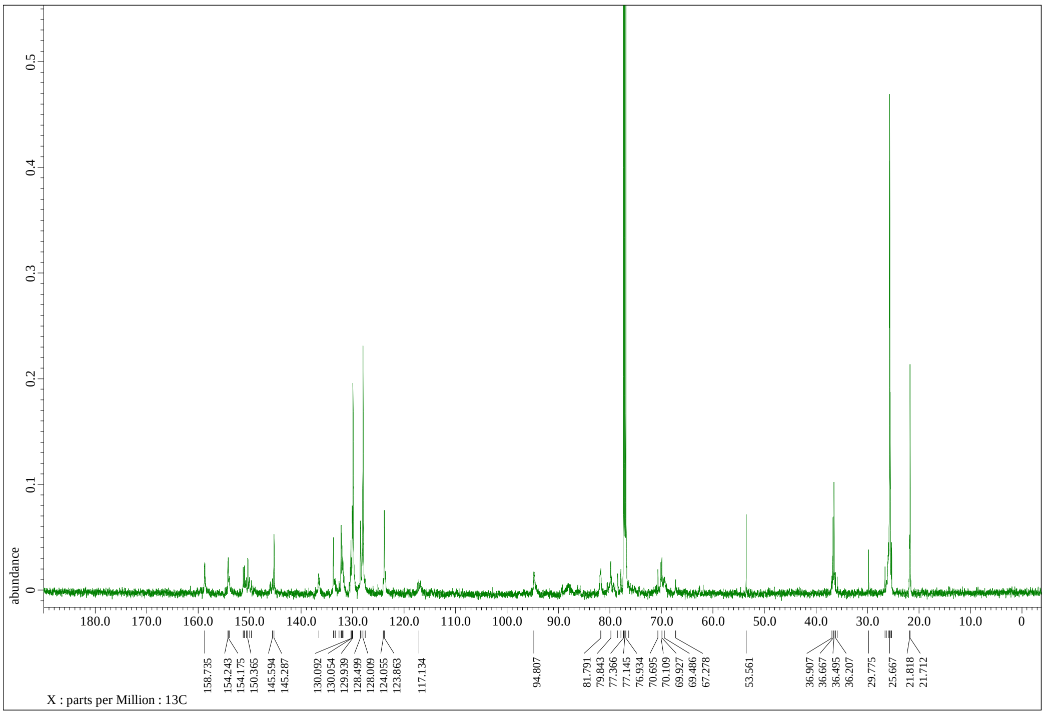

You created this PDF from an application that is not licensed to print to novaPDF printer (http://www.novapdf.com)

^{1}H and $^{13}C\{^{1}H\}$ NMR Spectra of Compound **16**

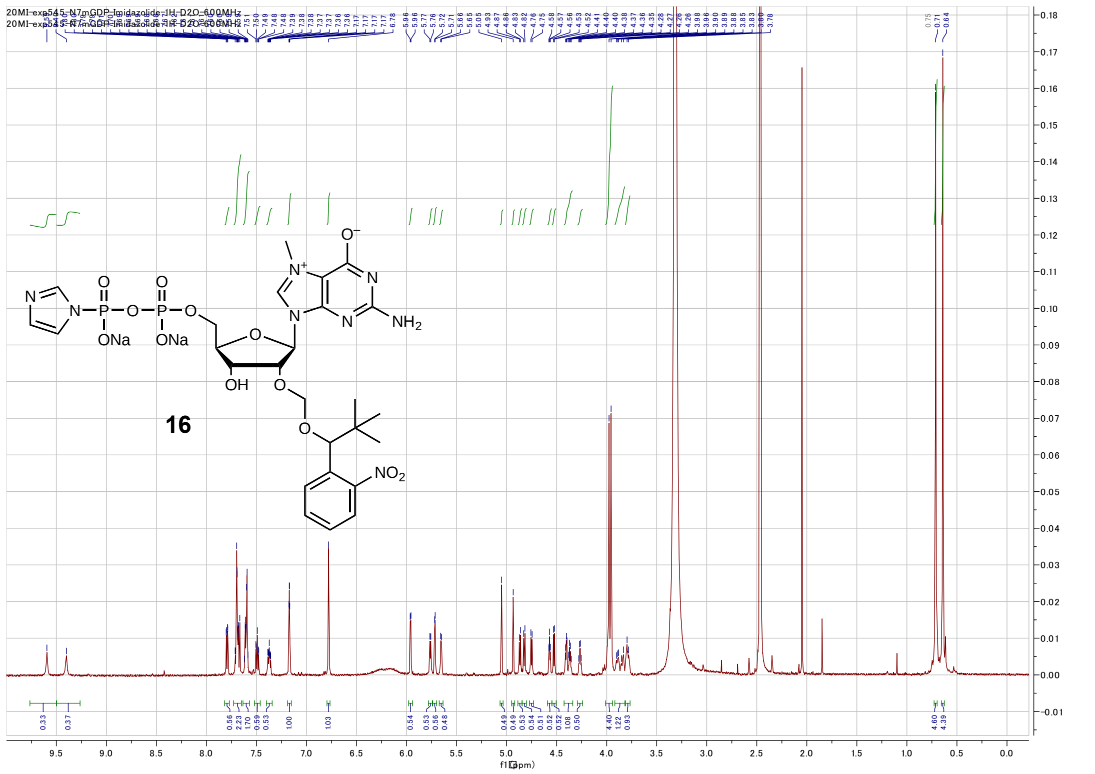

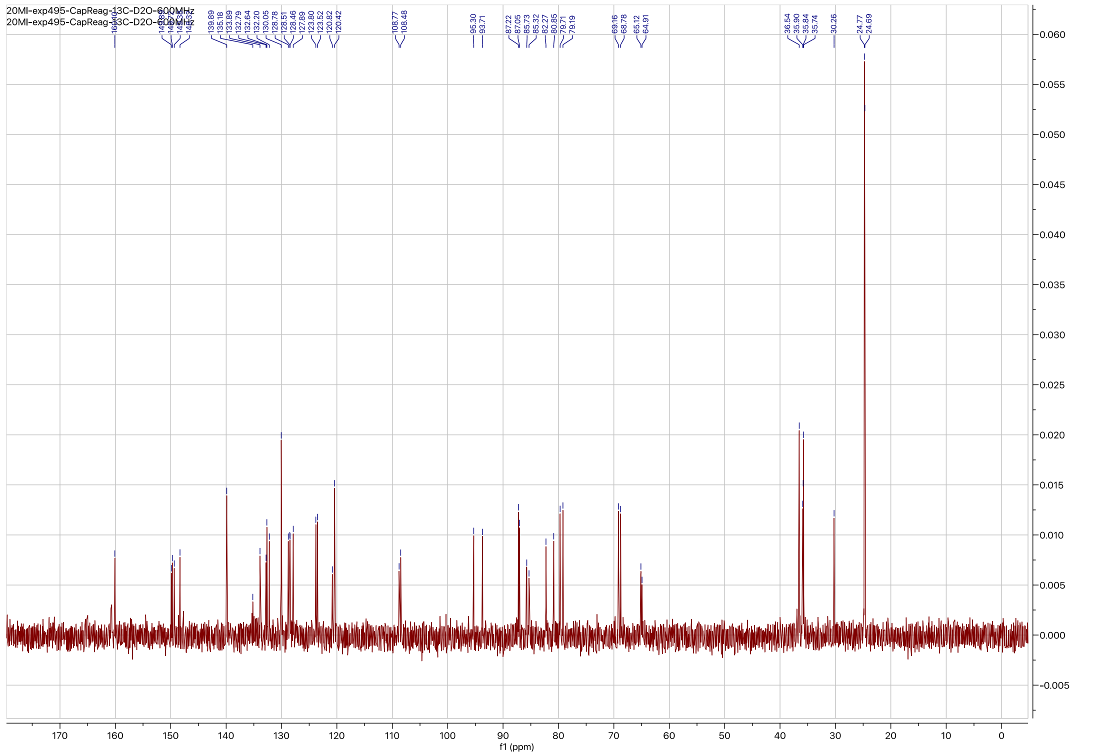

$^{31}P\{^1H\}$ NMR Spectrum of Compound **16**

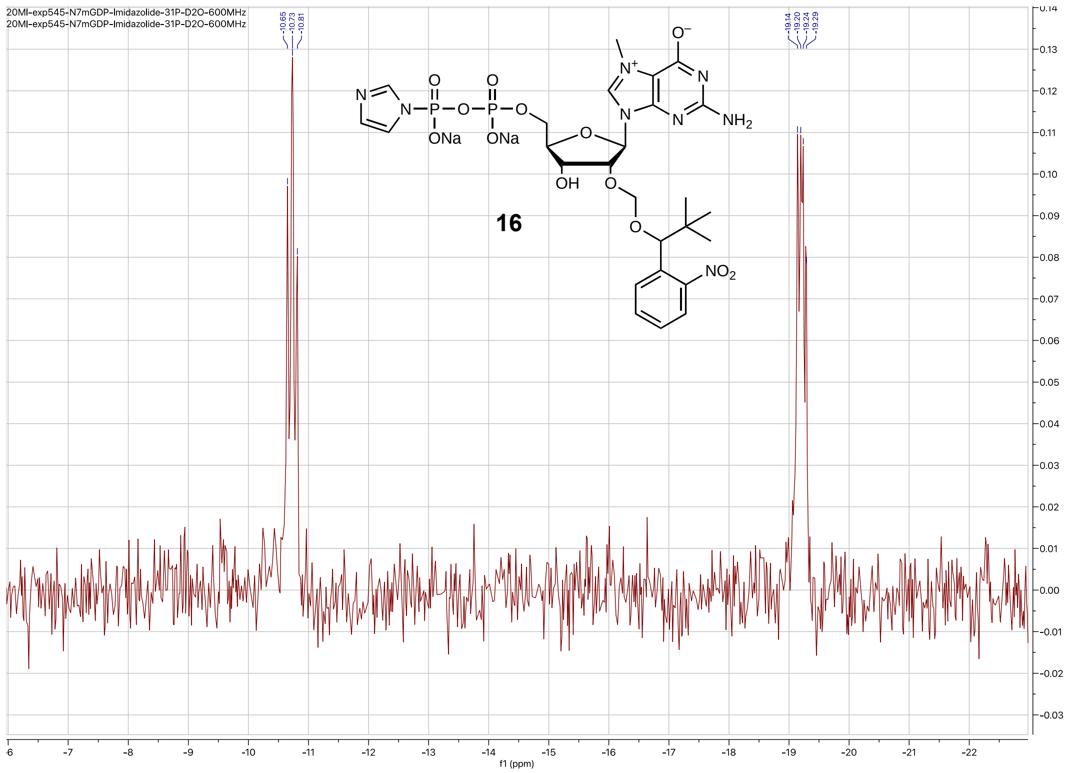

^{1}H and ^{13}C{^{1}H} NMR Spectra of Compound **S11**

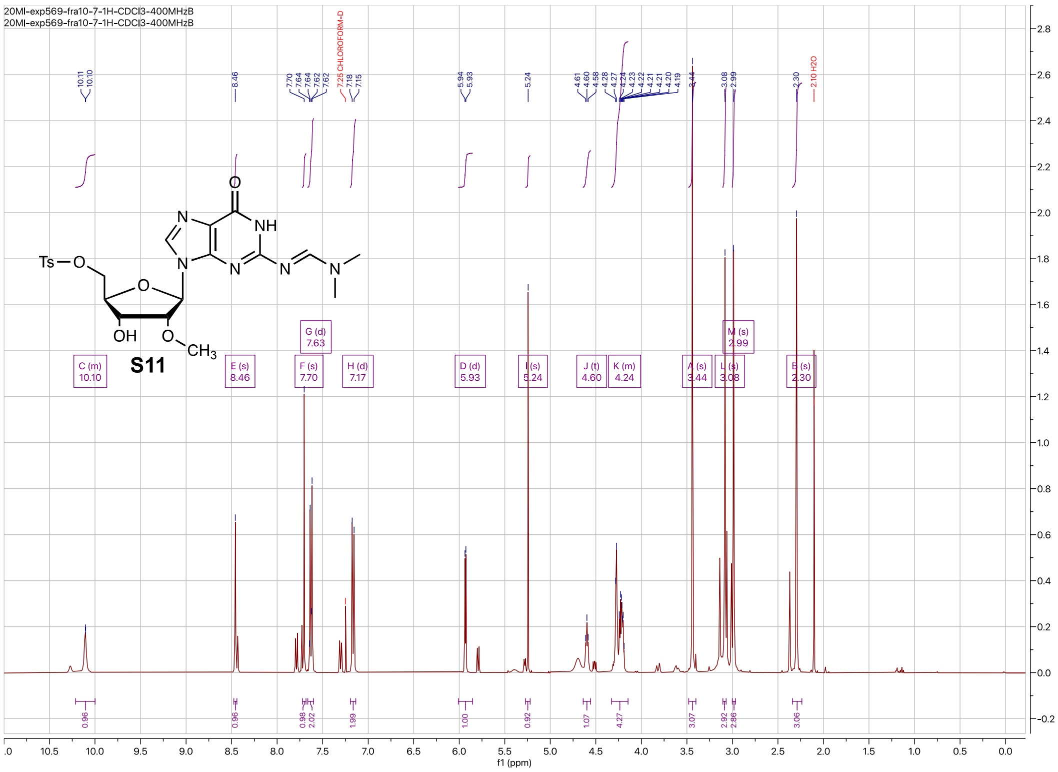

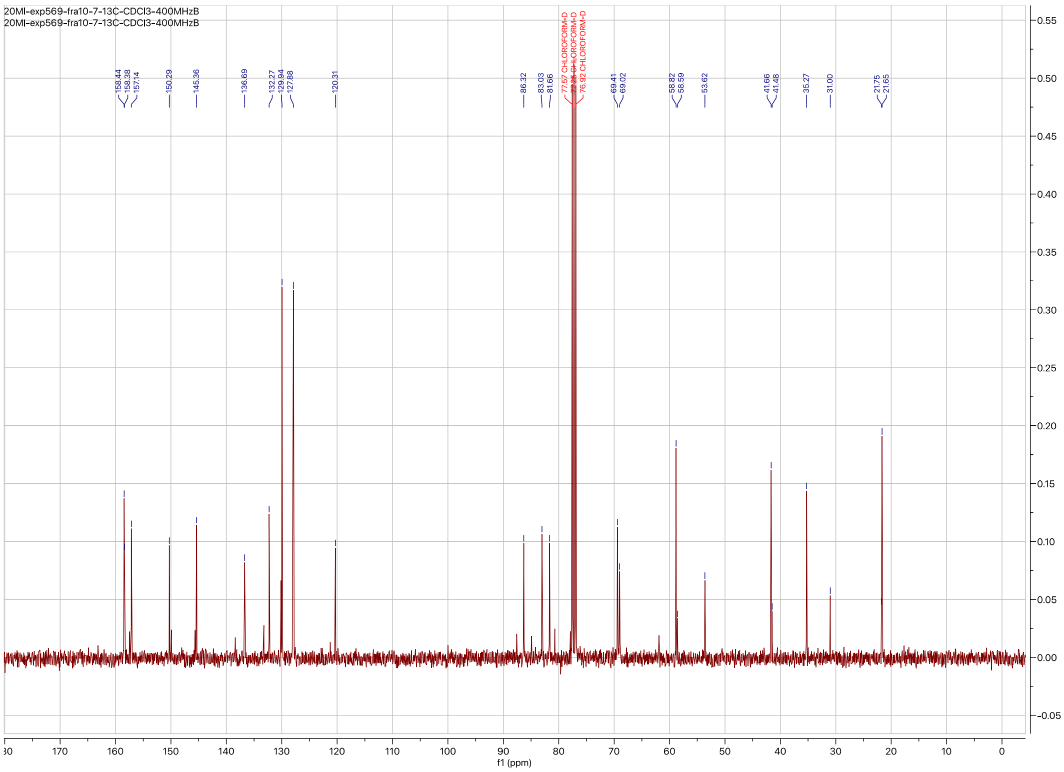

^{1}H and ^{13}C{^{1}H} NMR Spectra of Compound **S12**

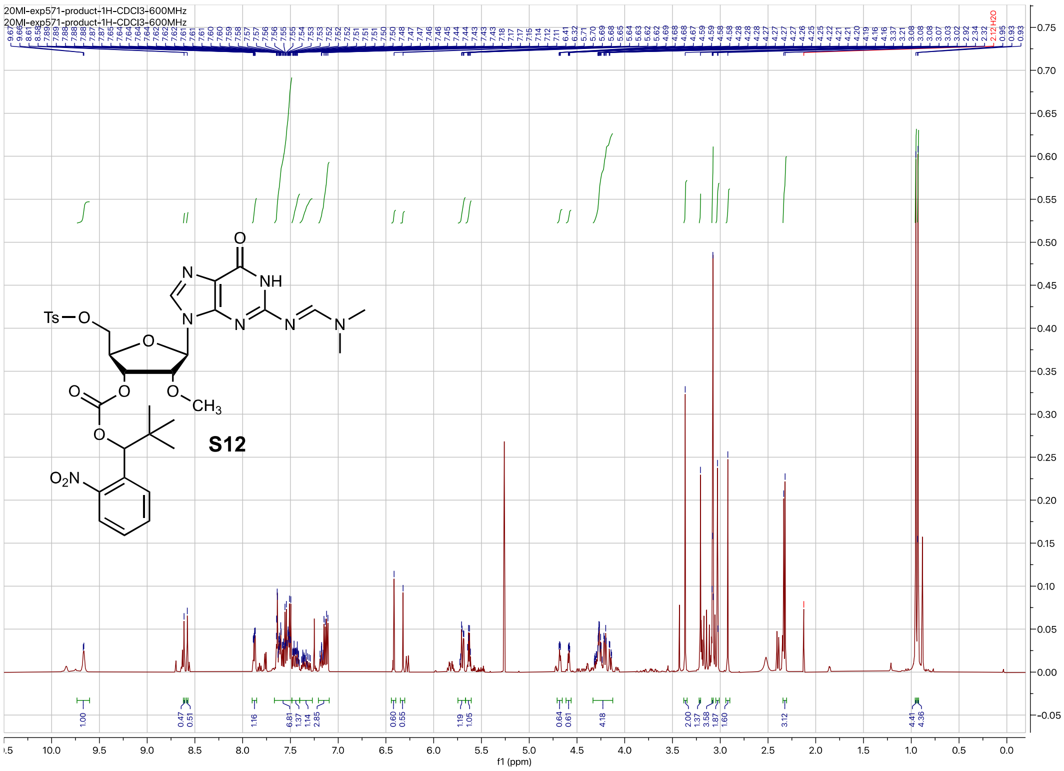

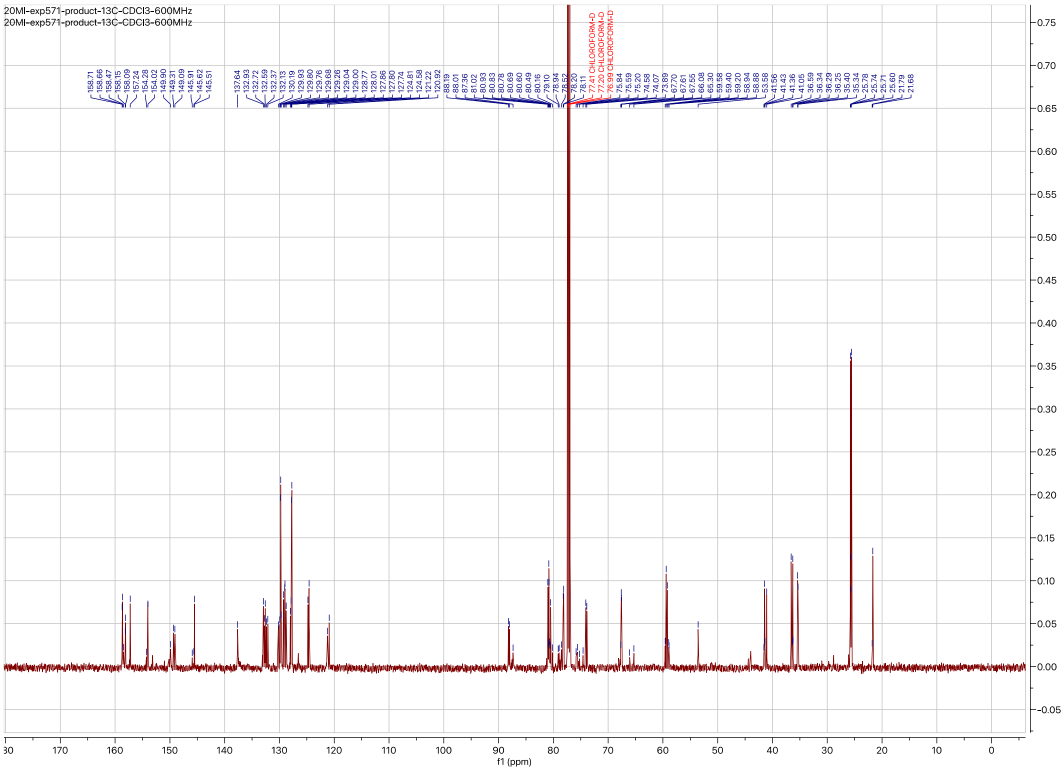

^{1}H and ^{13}C{^{1}H} NMR Spectra of Compound **S13**

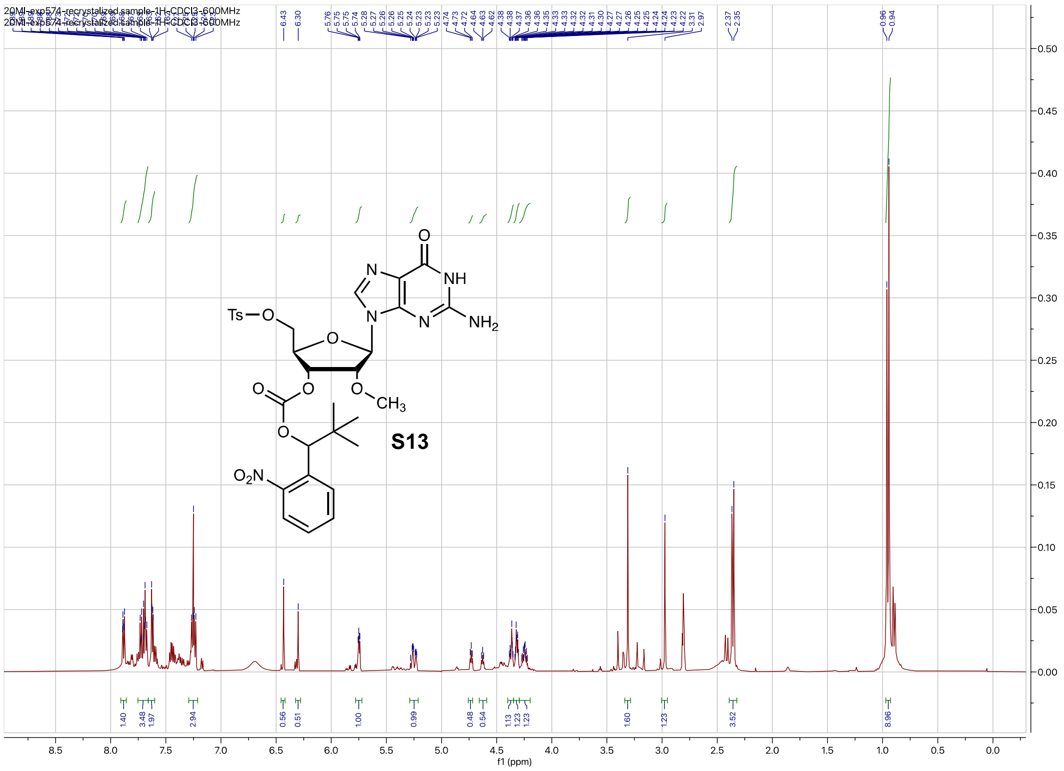

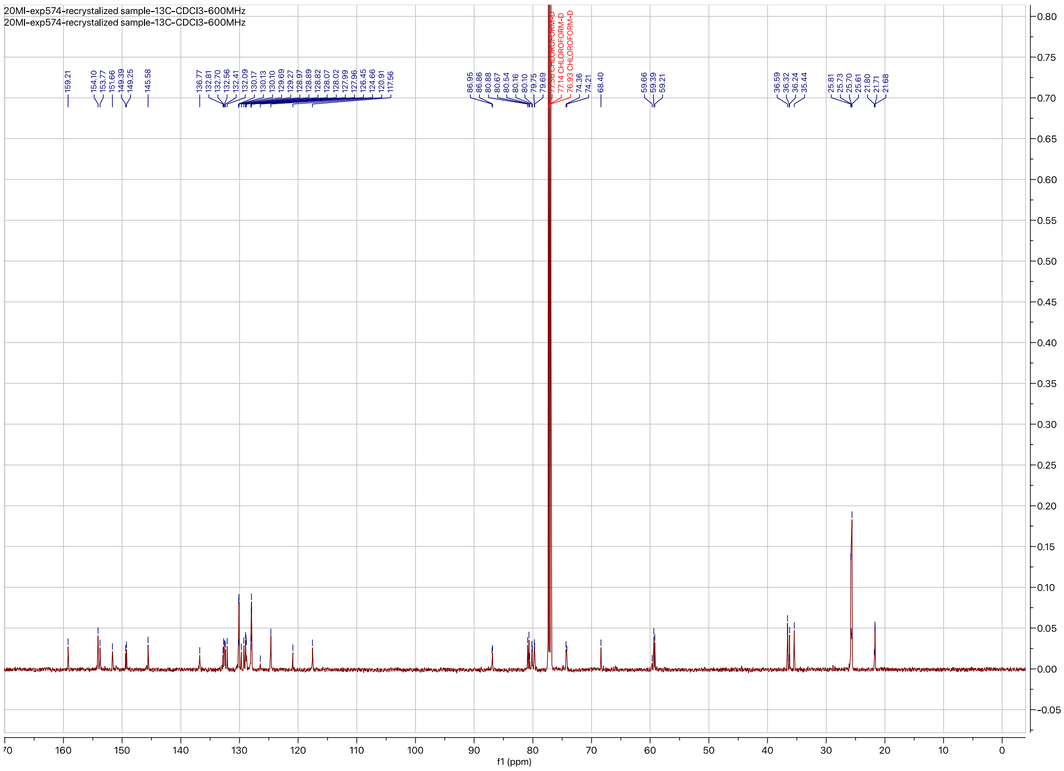

^1H and $^{13}C\{^1H\}$ NMR Spectra of Compound **S14**

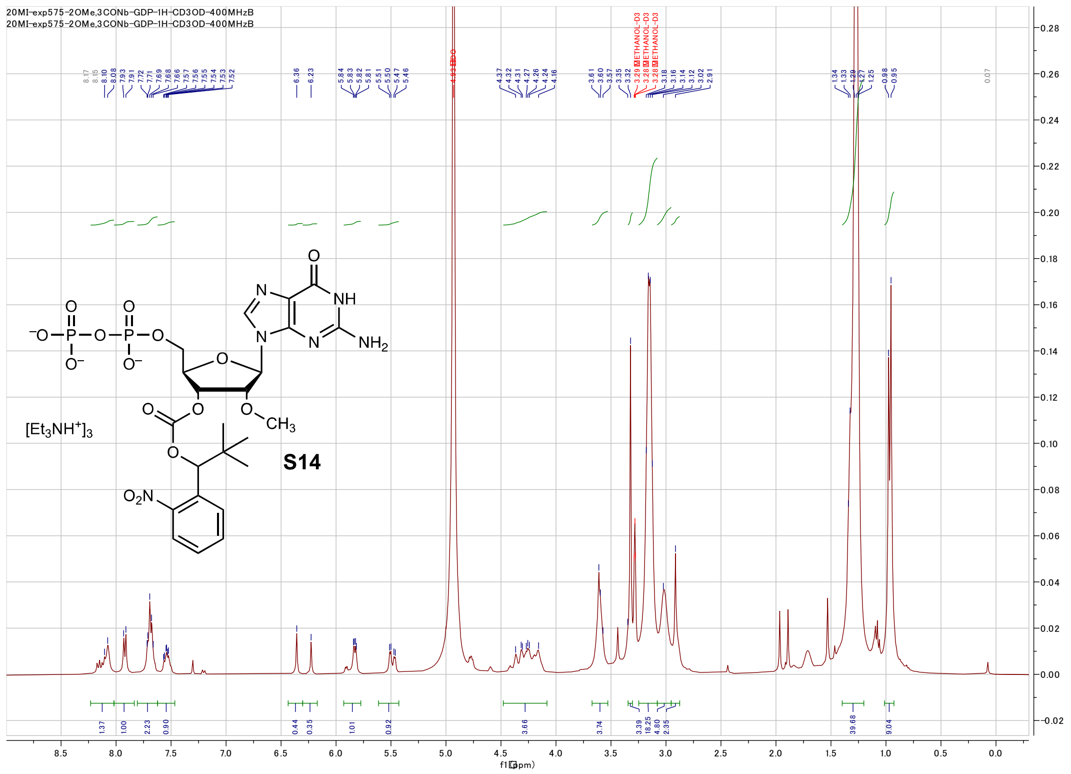

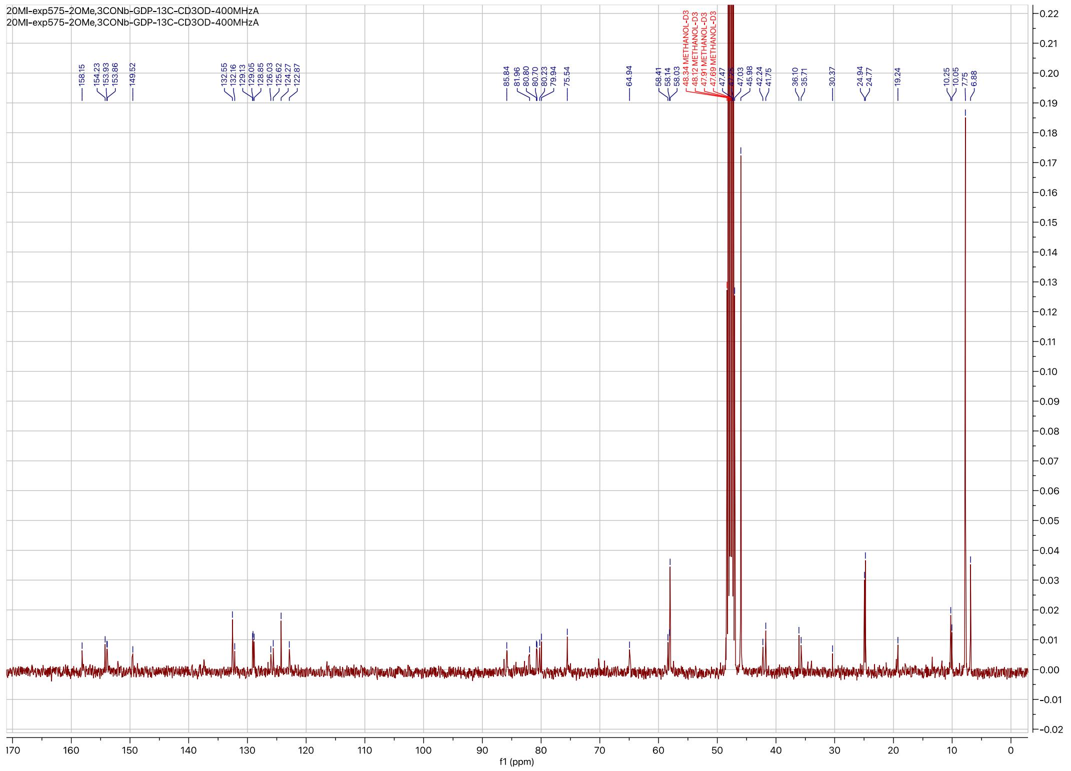

$^{31}P\{^{1}H\}$ NMR Spectrum of Compound **S14**

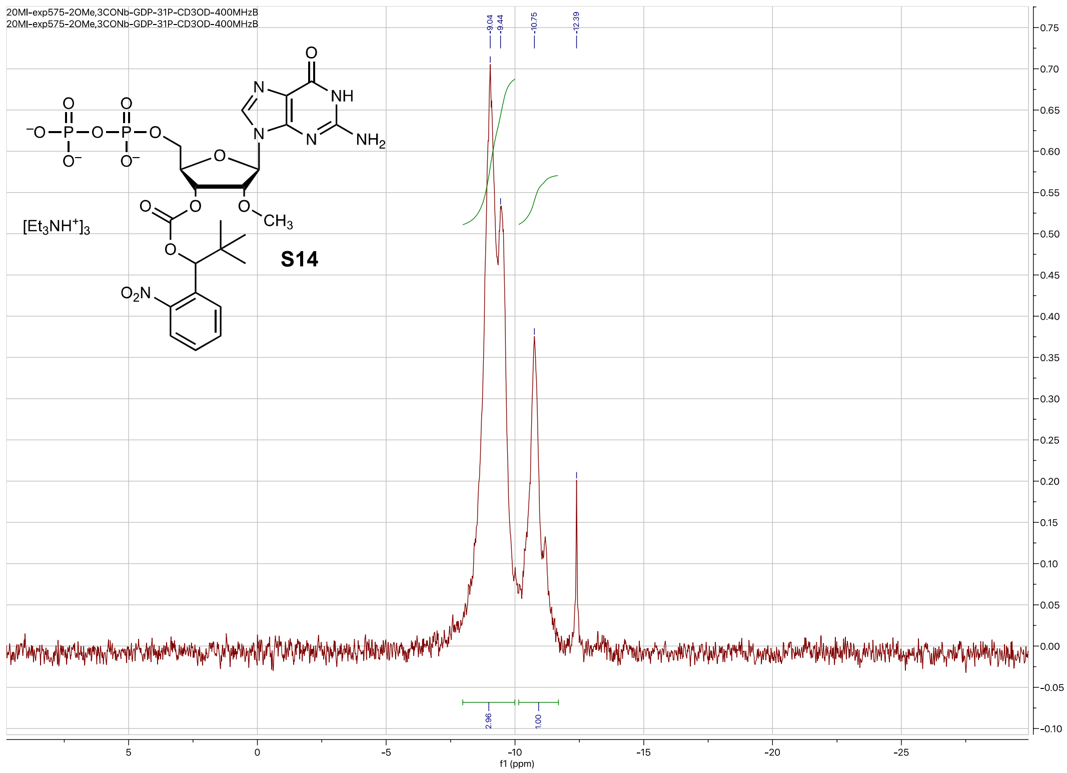

^{1}H and $^{13}C\{^{1}H\}$ NMR Spectra of Compound **S15**

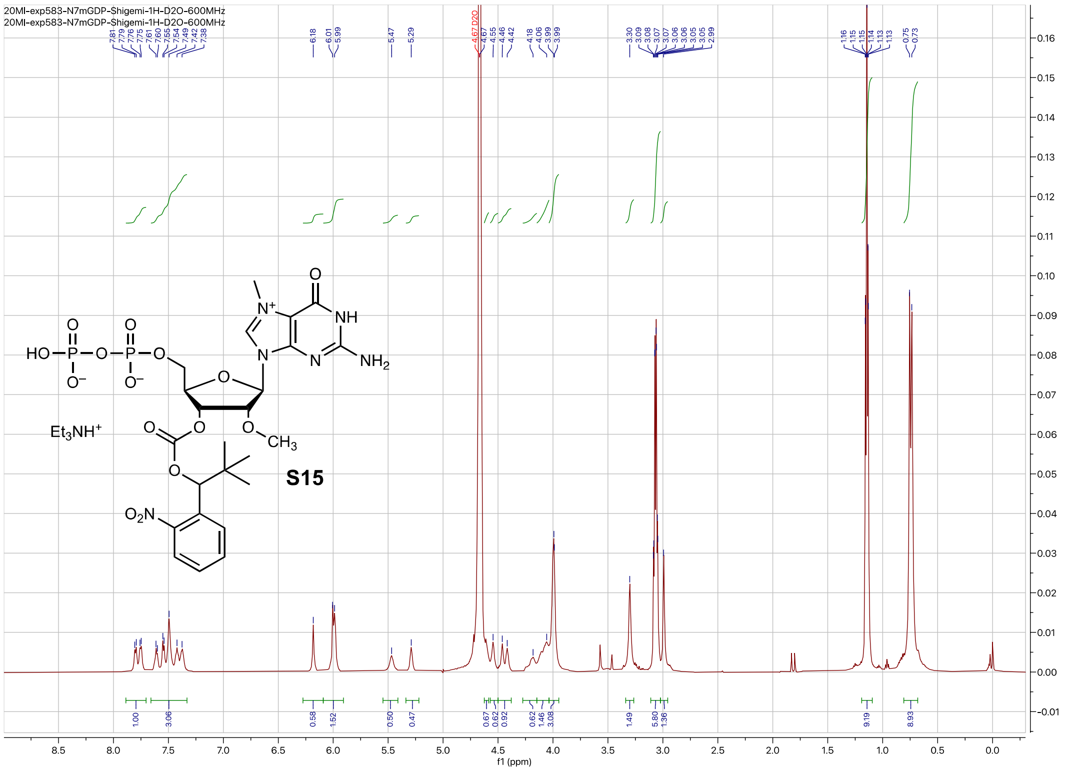

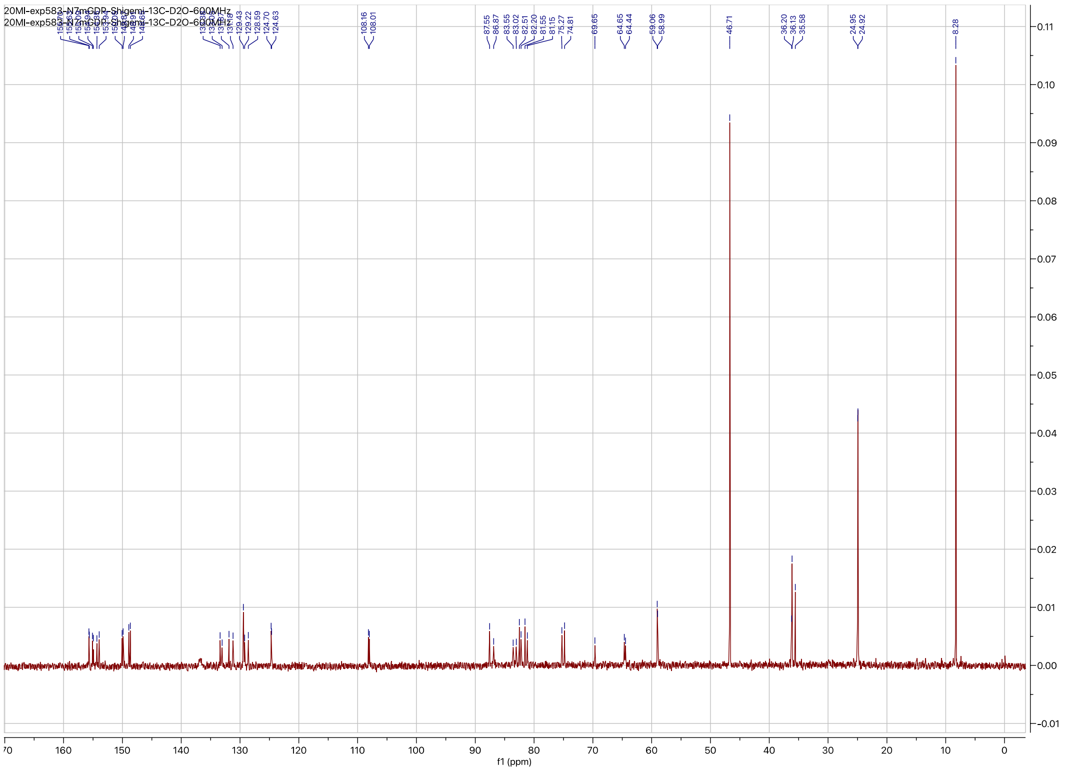

$^{31}P\{^{1}H\}$ NMR Spectrum of Compound **S15**

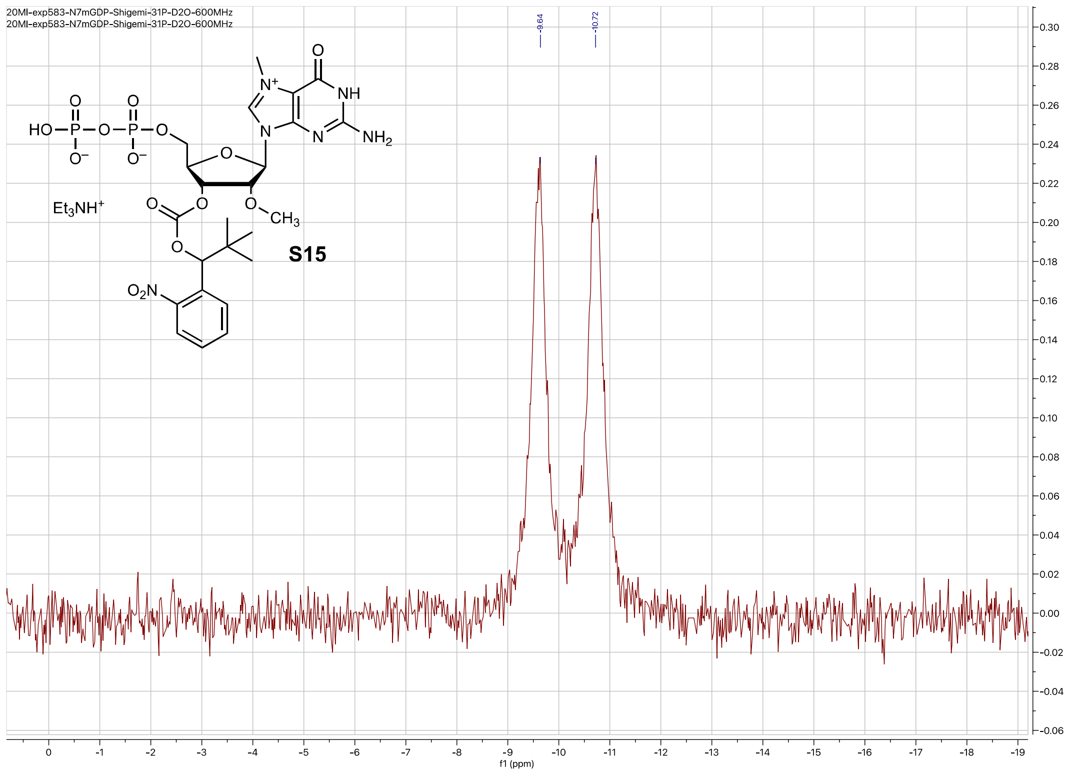

^{1}H and $^{13}C\{^{1}H\}$ NMR Spectra of Compound **2** (Triethylammonium salt)

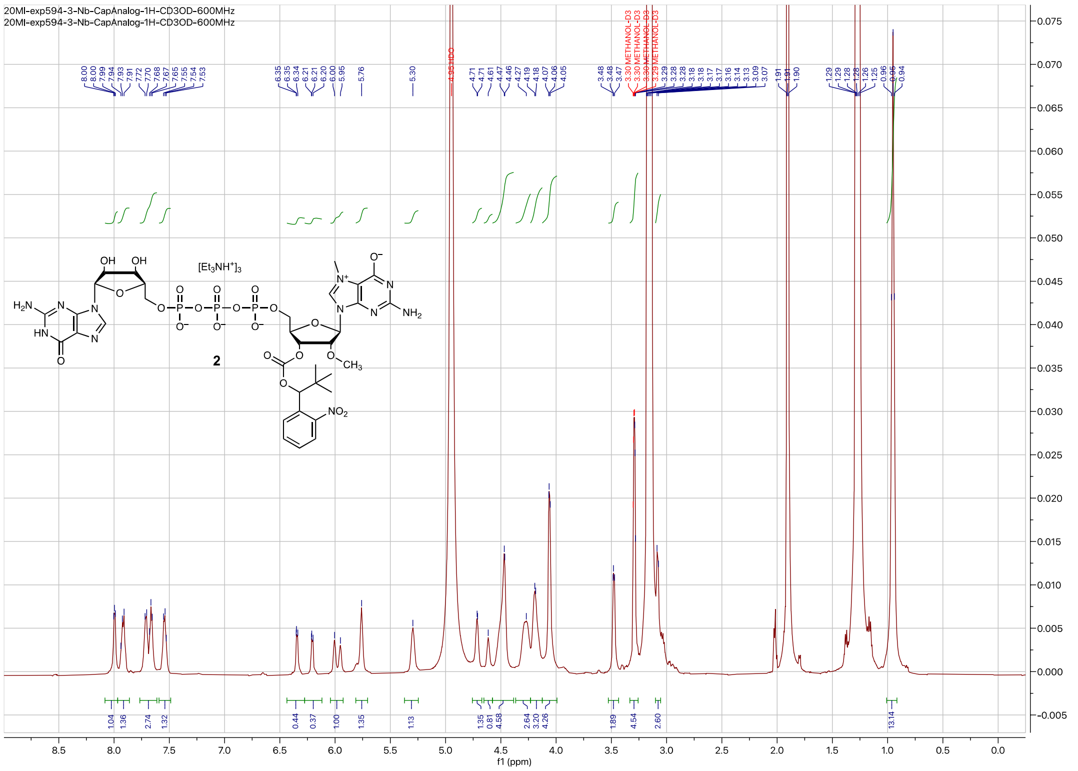

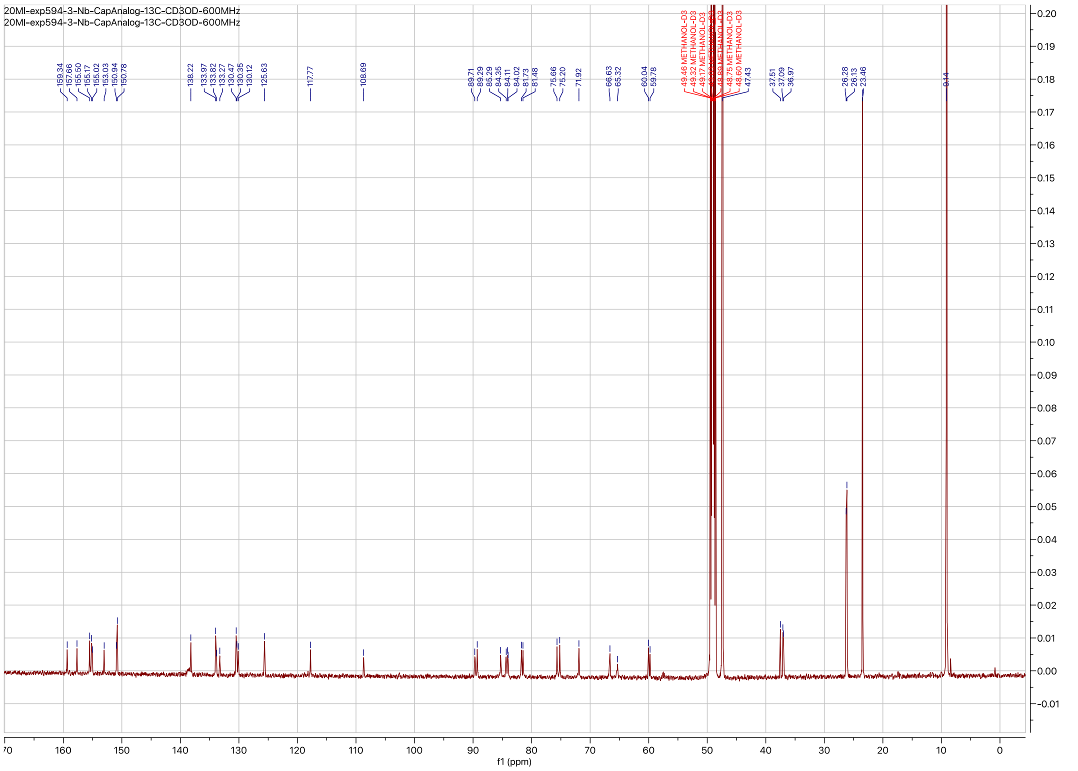

$^{31}P\{^1H\}$ NMR Spectrum of Compound **2** (Triethylammonium salt)

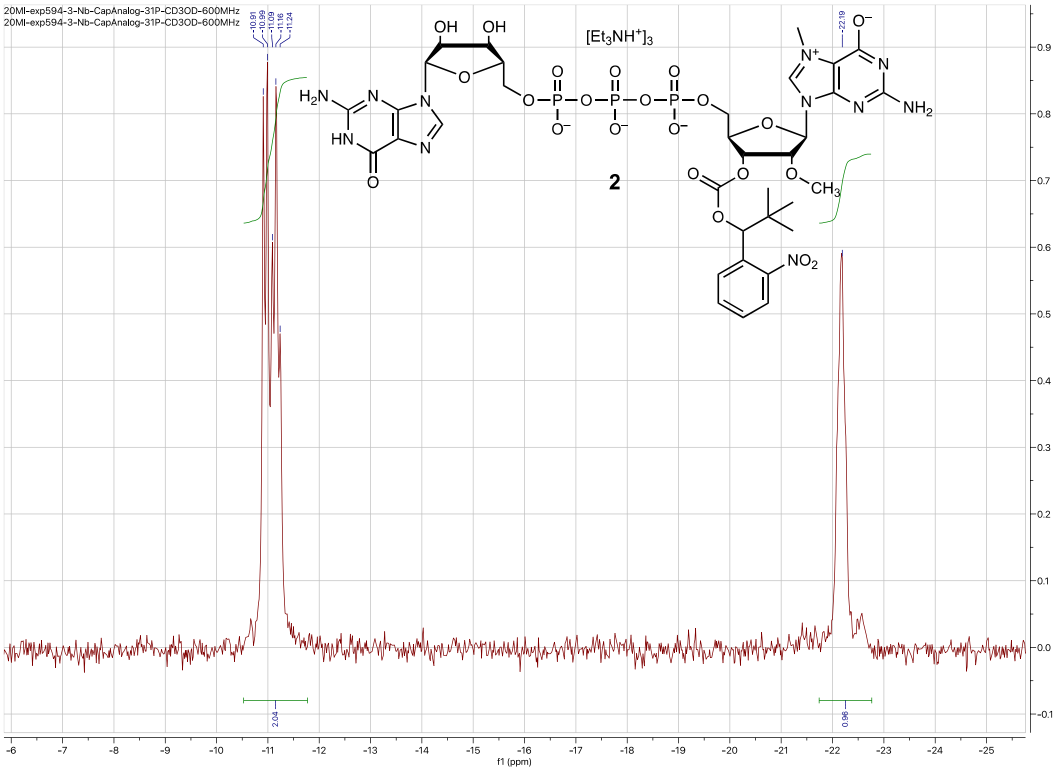

^{1}H and ^{31}P{^{1}H} NMR Spectra of Compound **2** (Sodium salt)

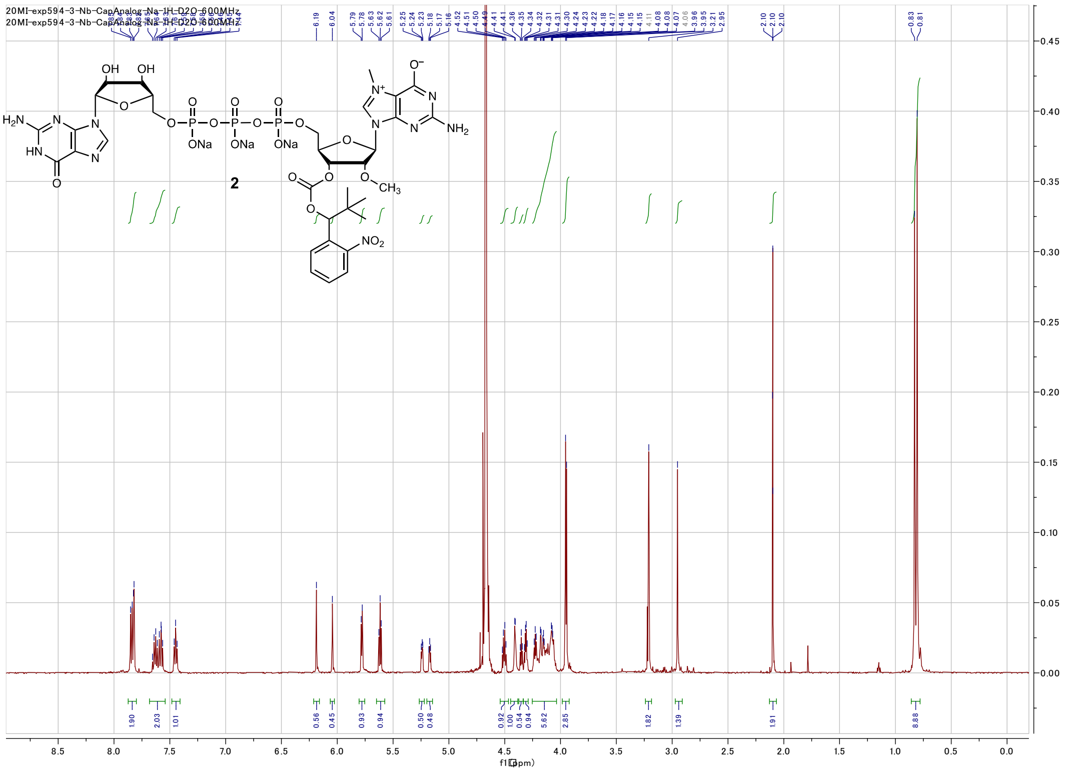

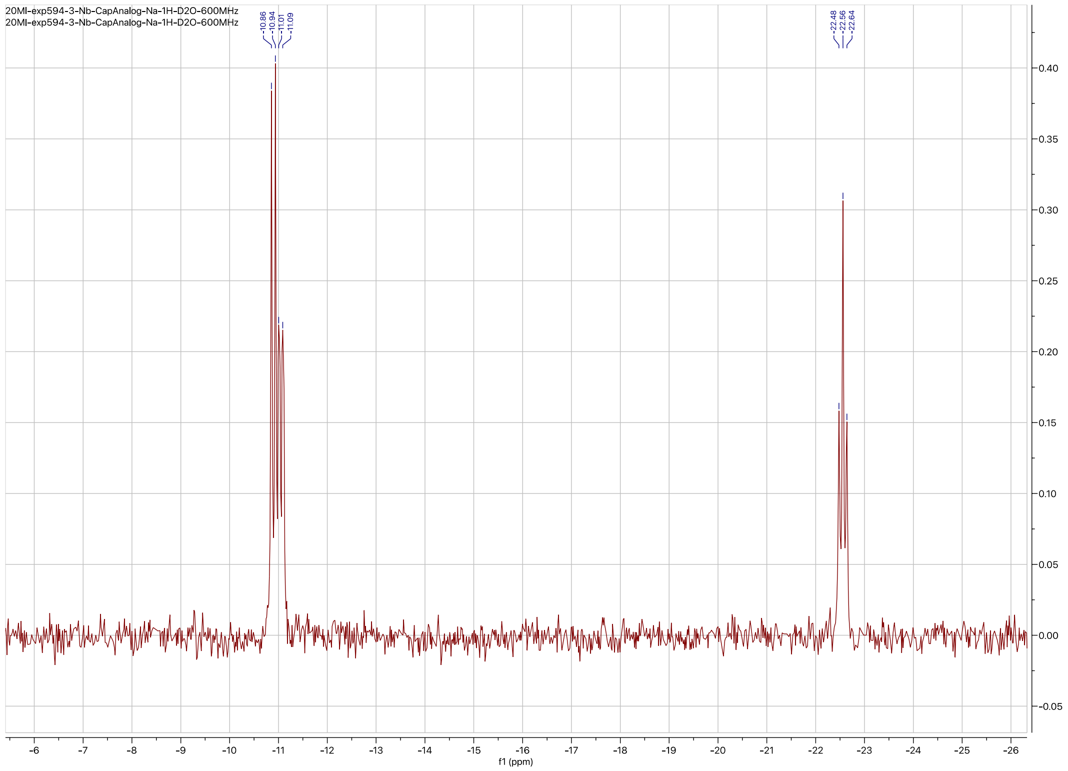

^{1}H and ^{13}C{^{1}H} NMR Spectra of Compound **S17**

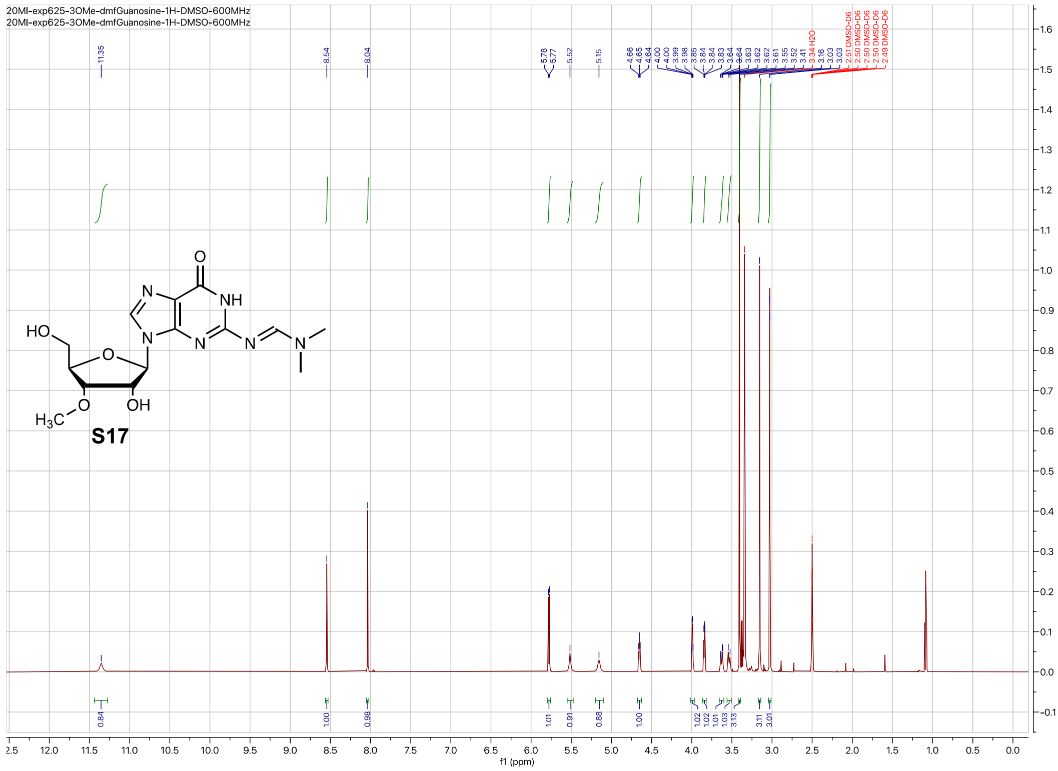

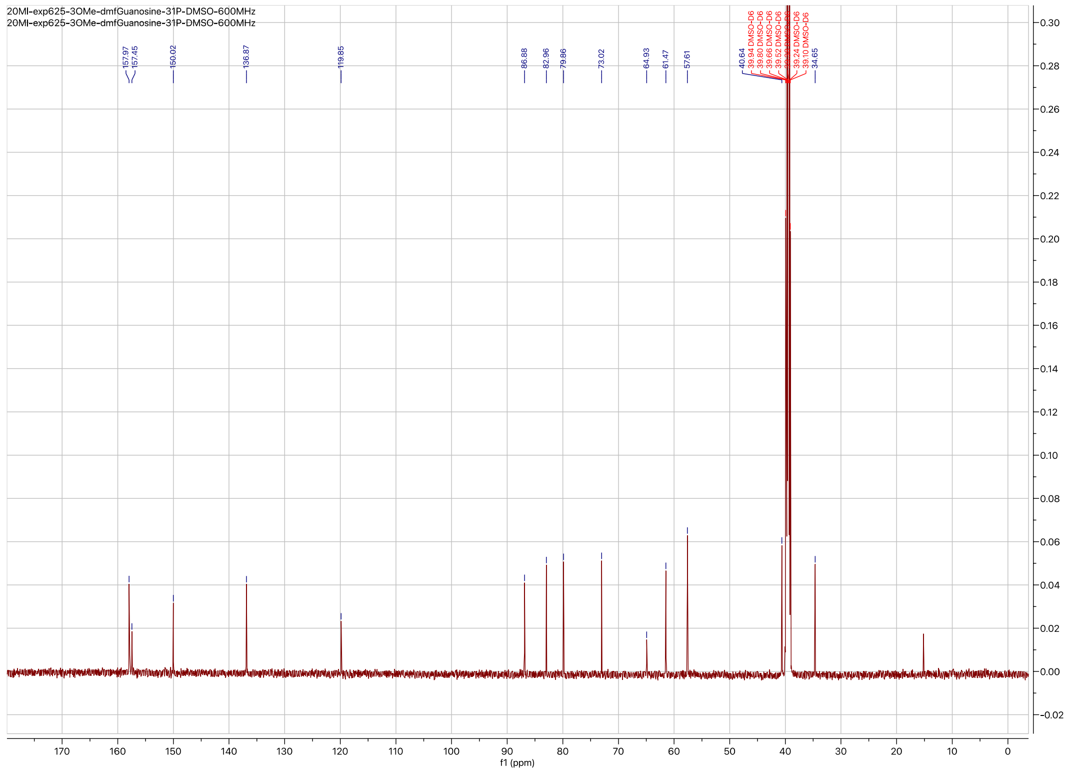

^{1}H and ^{13}C{^{1}H} NMR Spectra of Compound **S18**

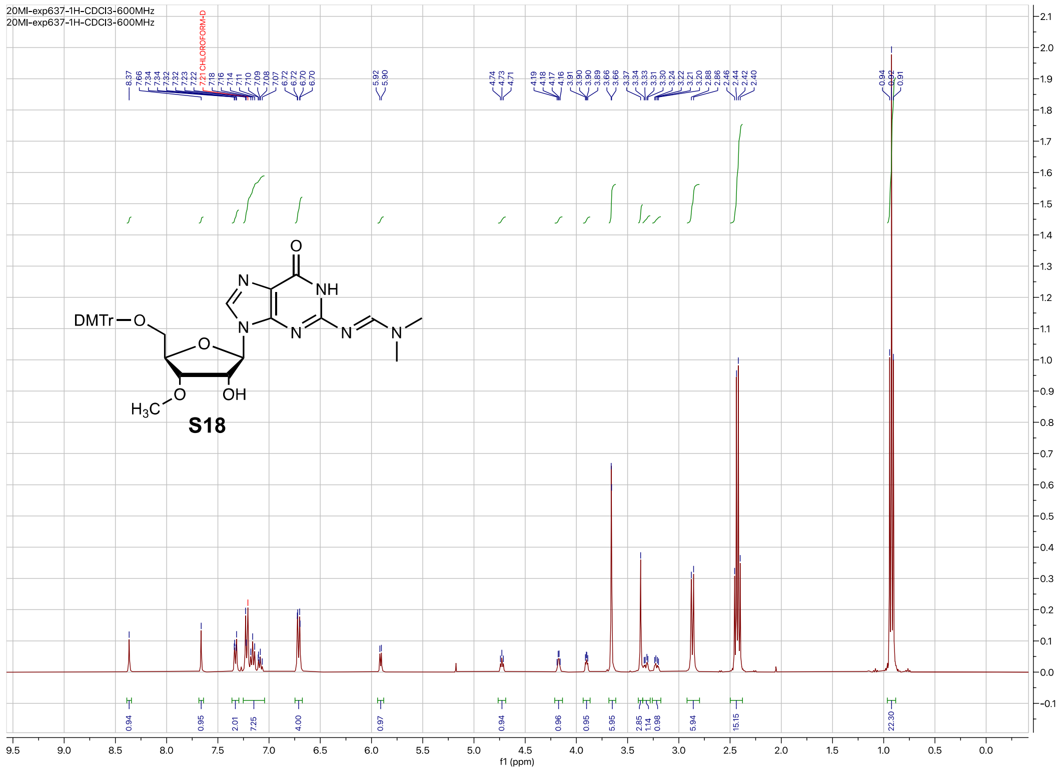

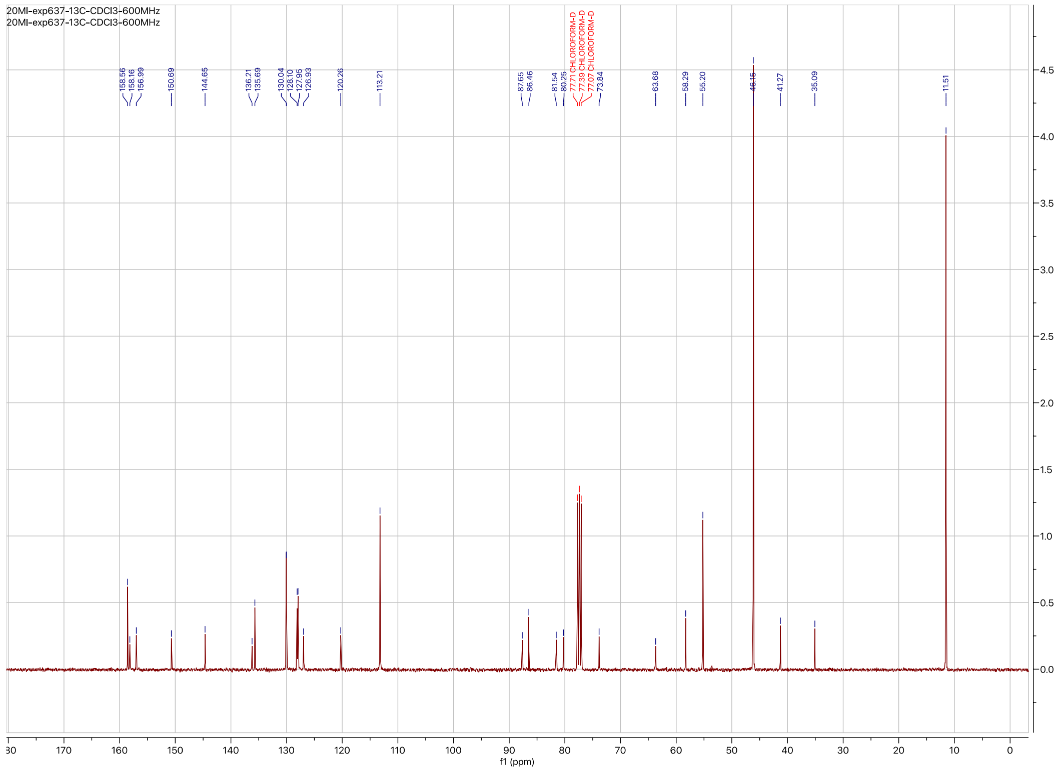

^{1}H and $^{13}C\{^{1}H\}$ NMR Spectra of Compound **S19**

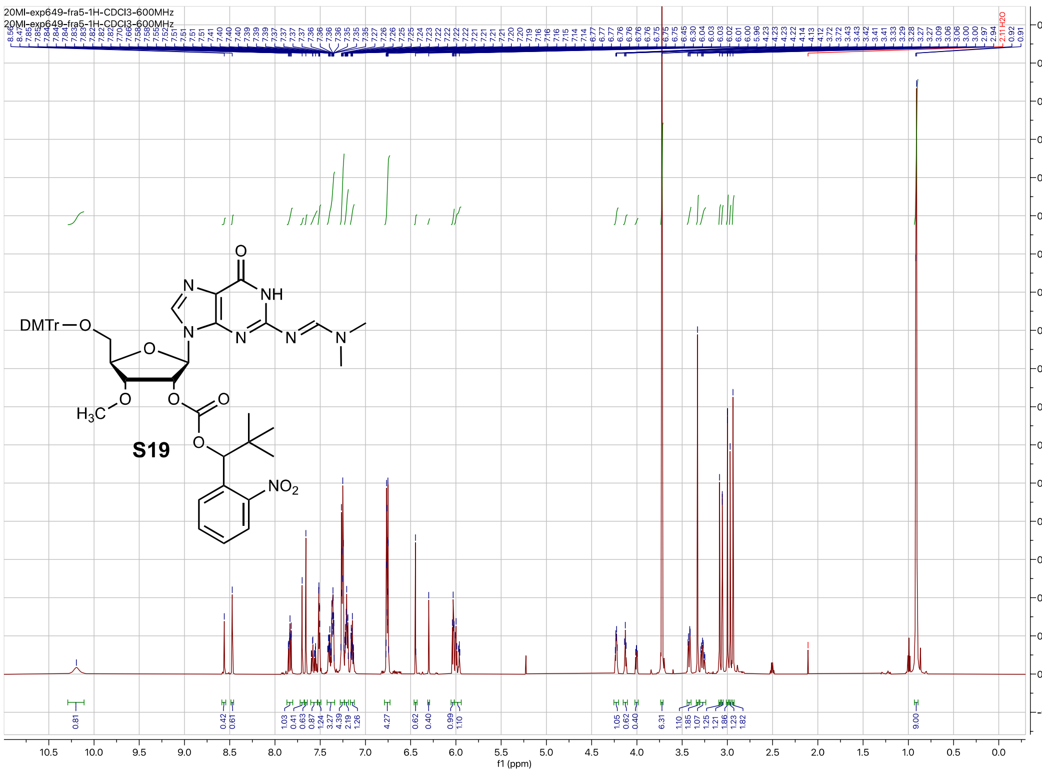

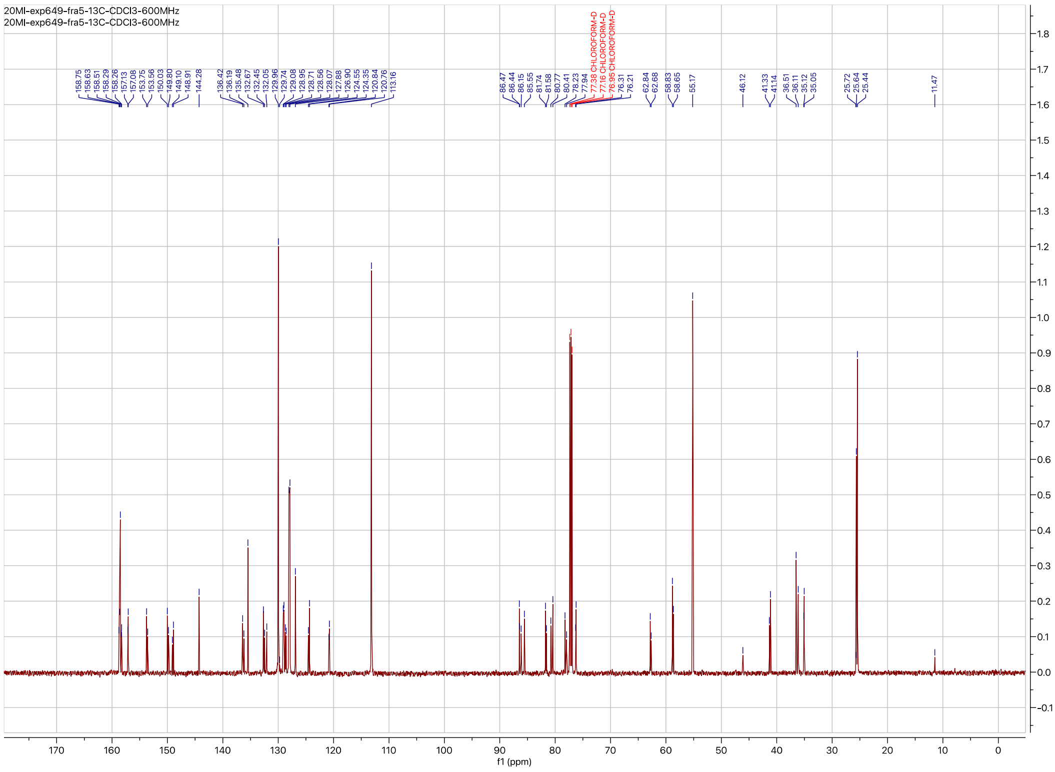

^{1}H and ^{13}C{^{1}H} NMR Spectra of Compound **S20**

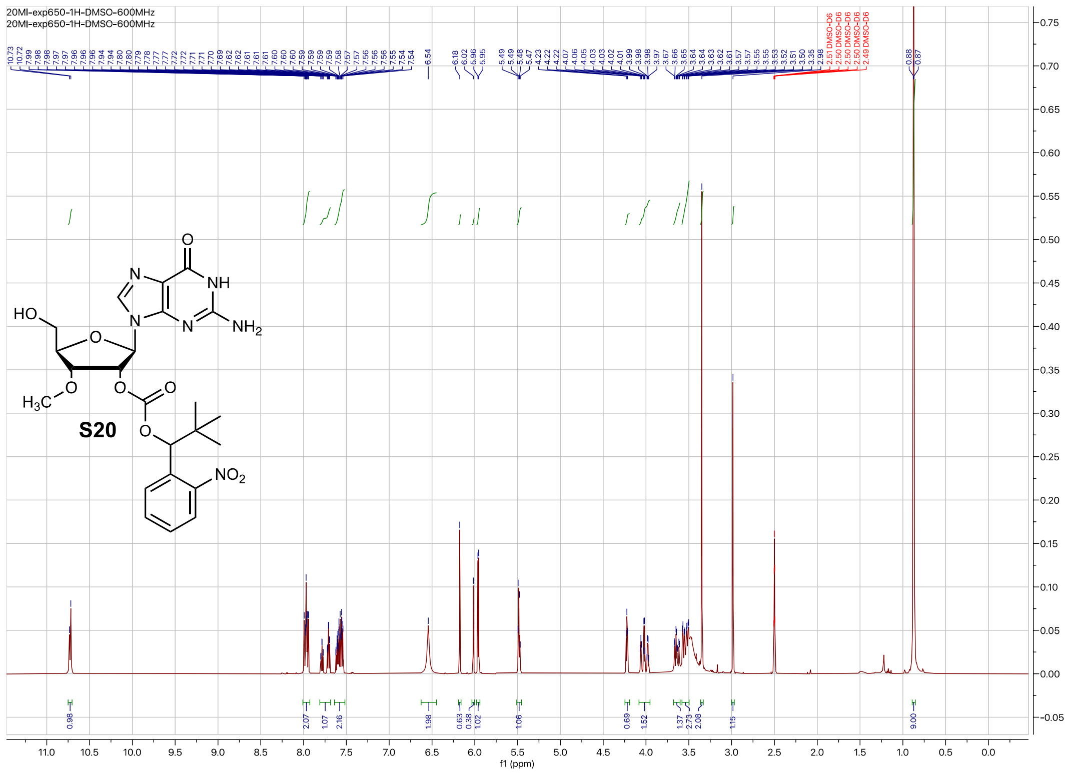

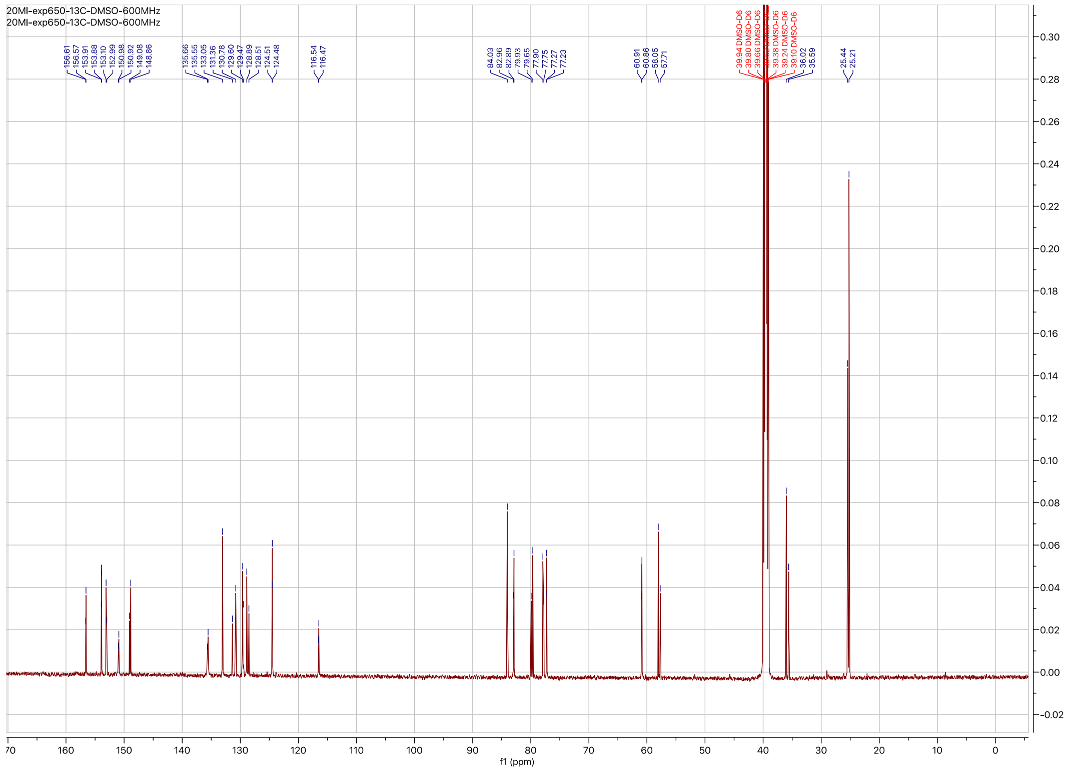

^{1}H and $^{13}C\{^{1}H\}$ NMR Spectra of Compound **S21**

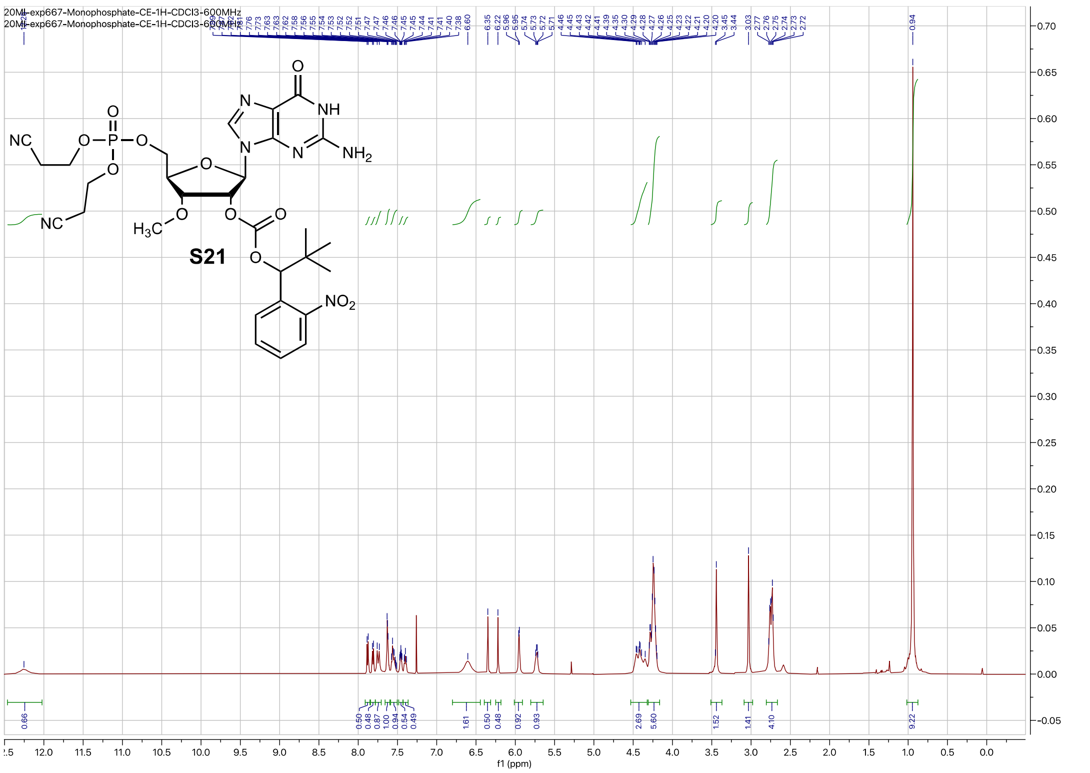

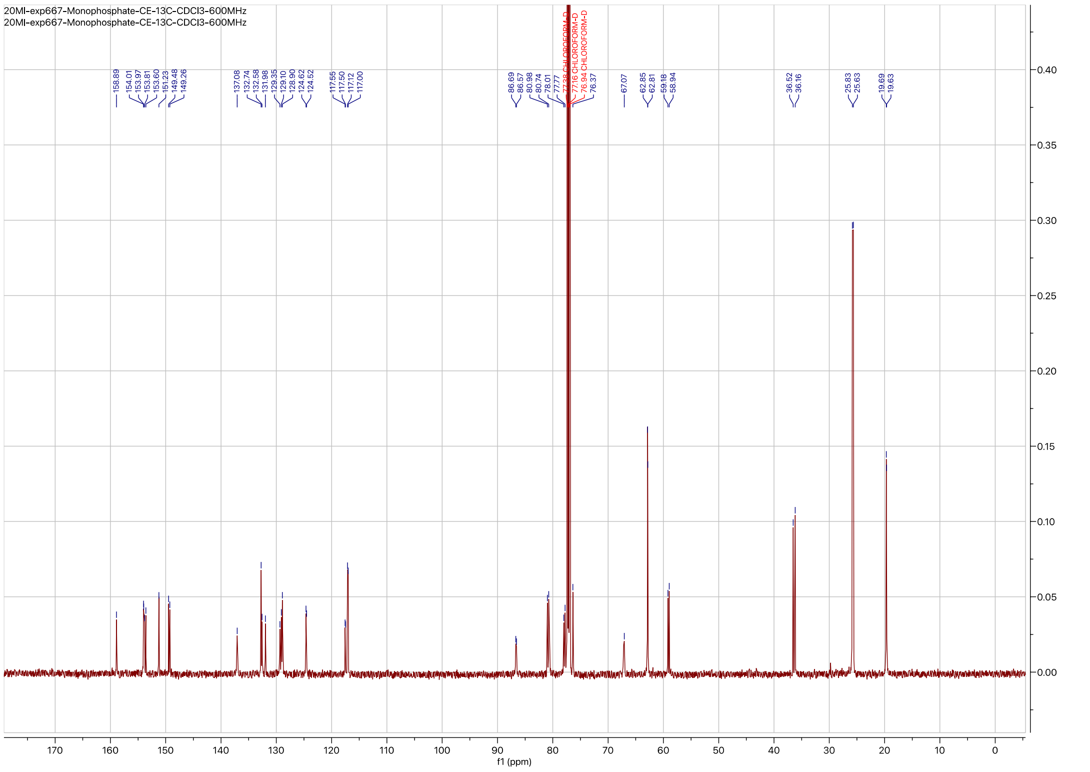

$^{31}P\{^1H\}$ NMR Spectrum of Compound **S21**

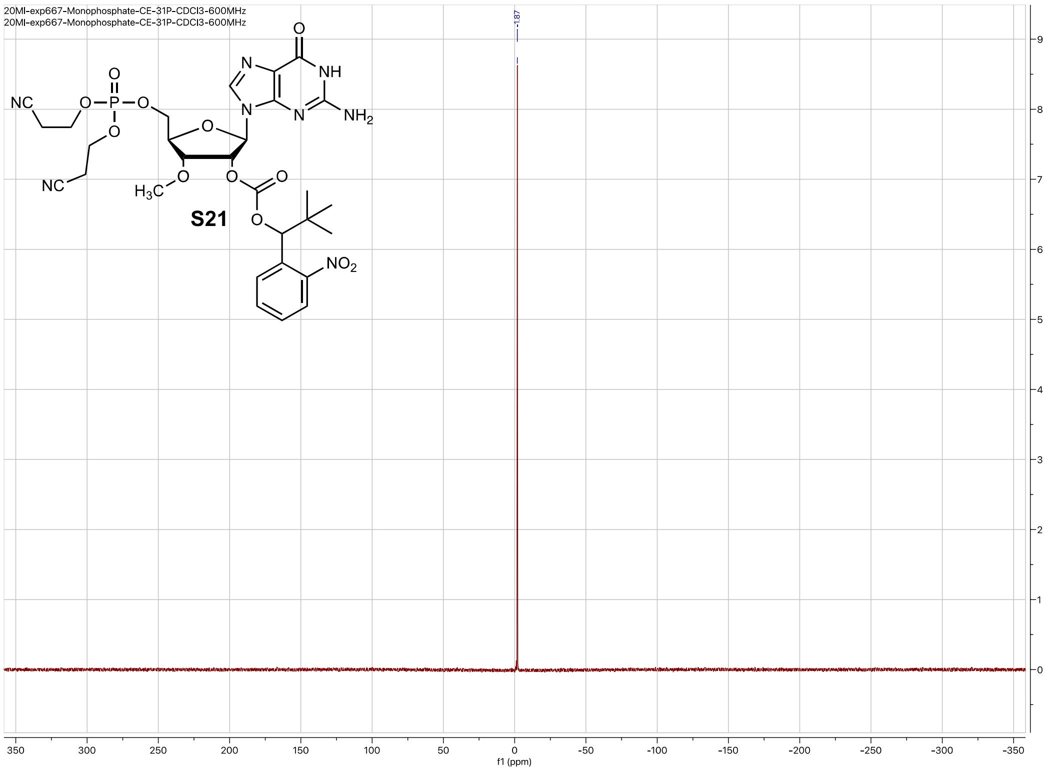

^{1}H and $^{13}C\{^{1}H\}$ NMR Spectra of Compound **S22**

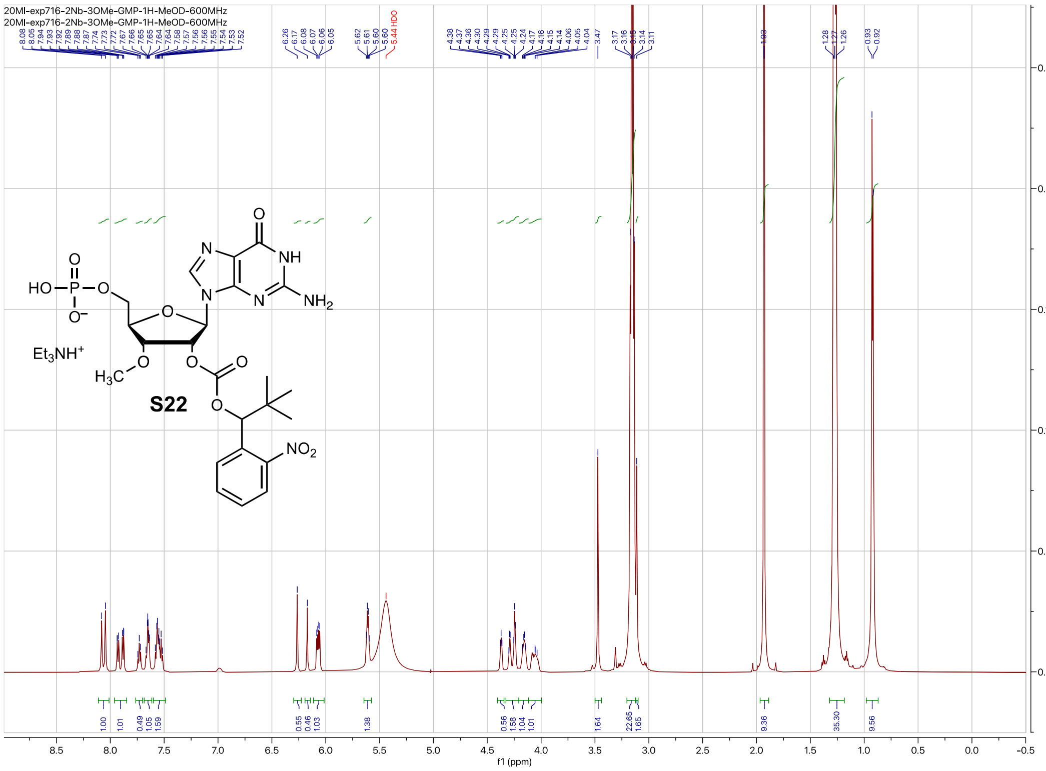

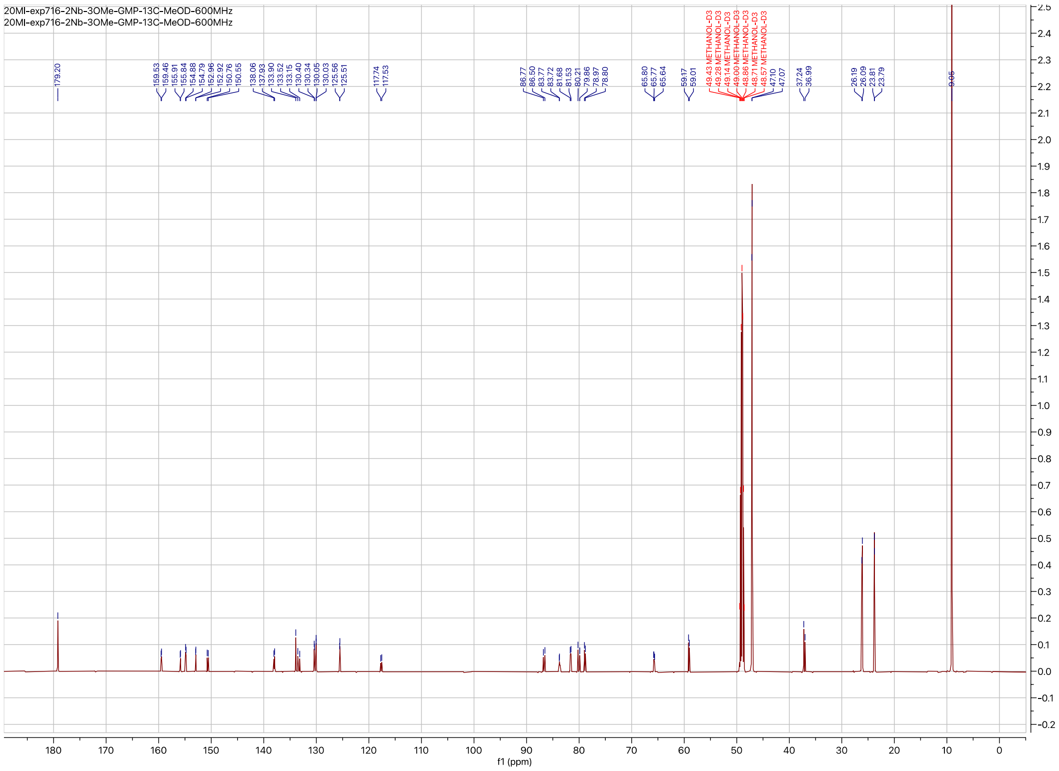

$^{31}P\{^1H\}$ NMR Spectrum of Compound **S22**

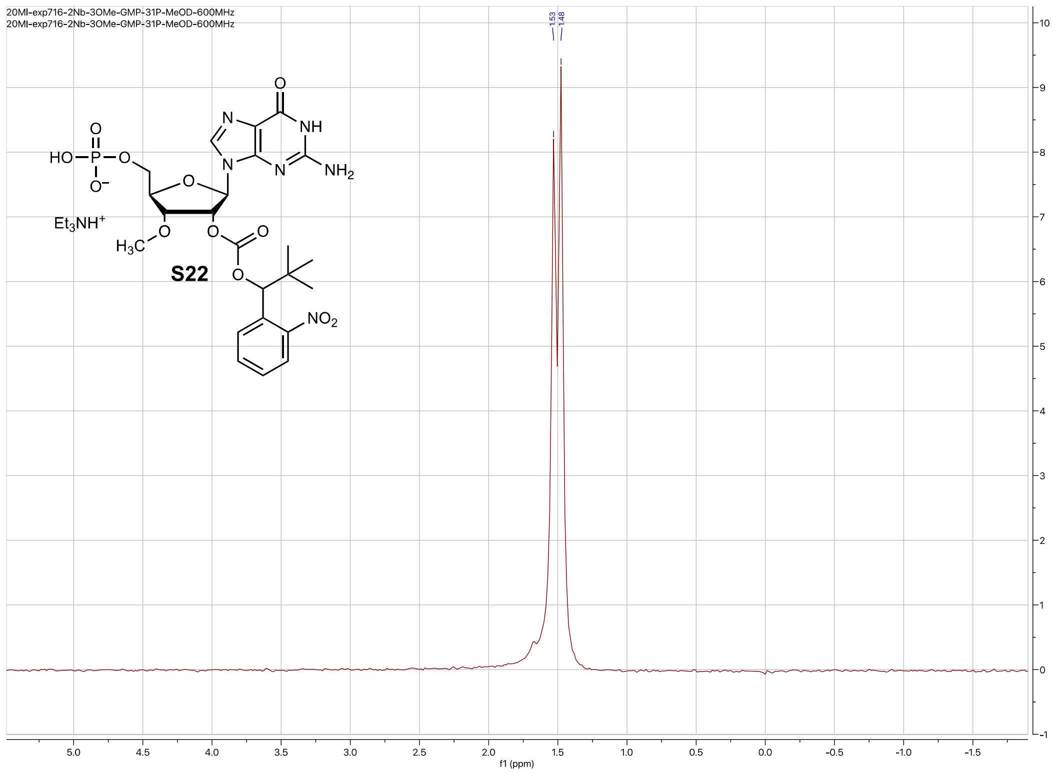

^{1}H and $^{13}C\{^{1}H\}$ NMR Spectra of Compound **S23**

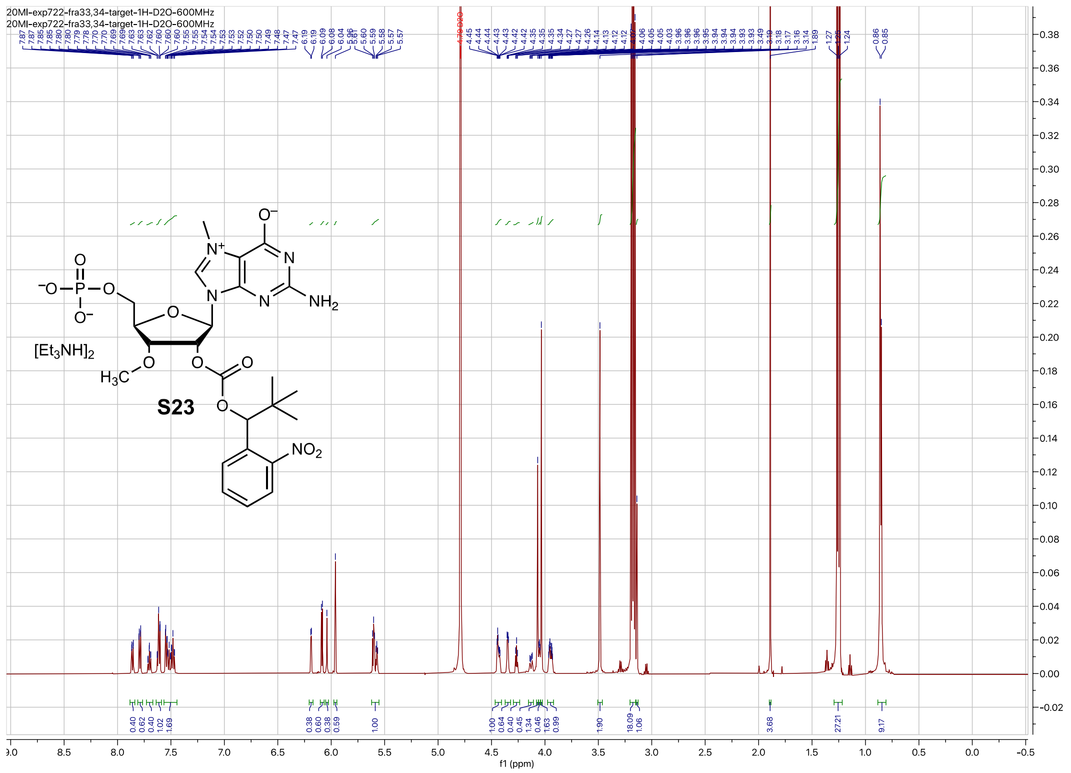

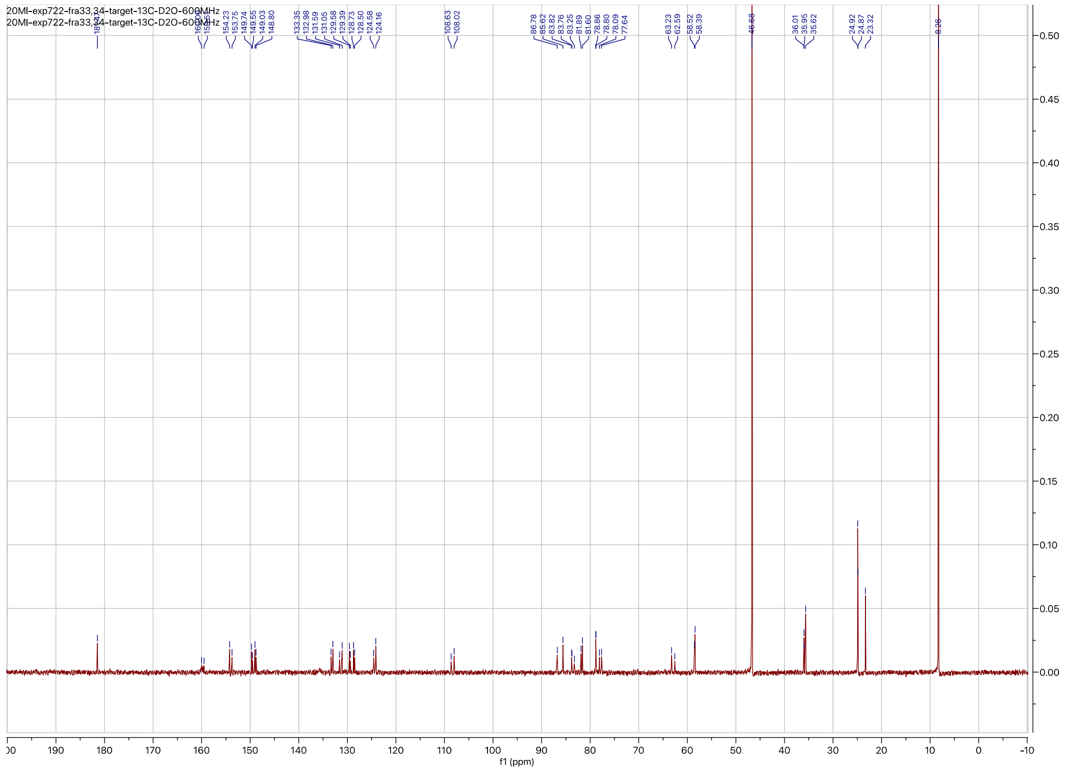

$^{31}P\{^1H\}$ NMR Spectrum of Compound **S23**

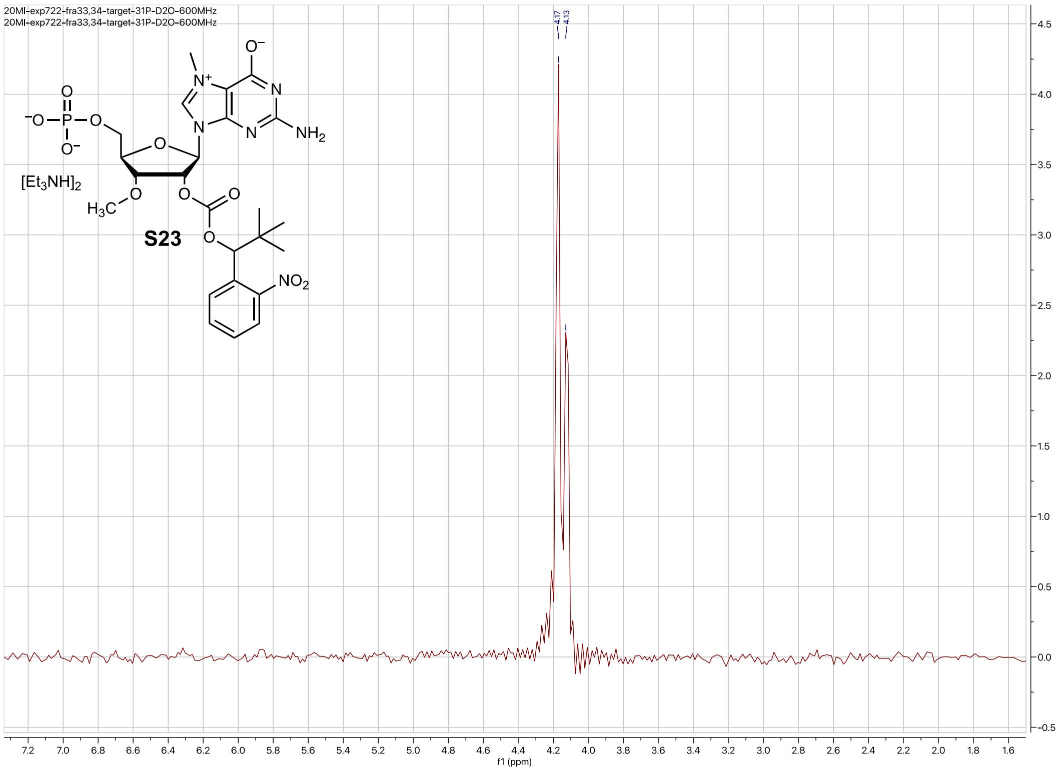

^{1}H and $^{13}C\{^{1}H\}$ NMR Spectra of Compound **3**

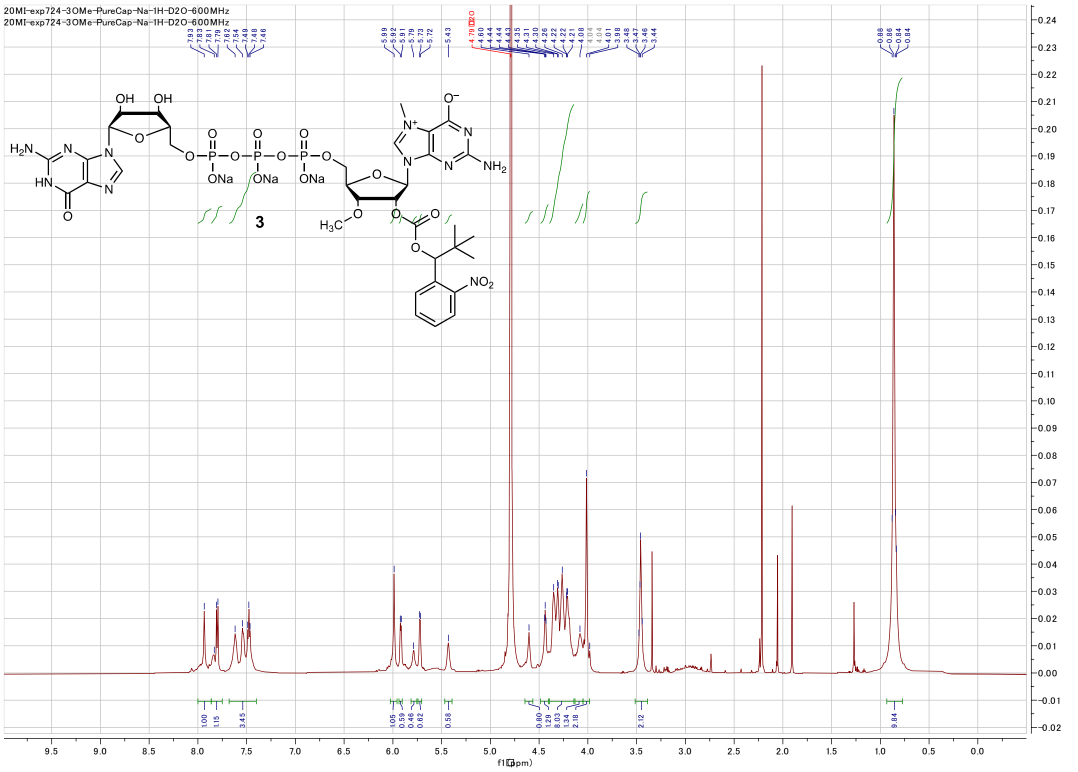

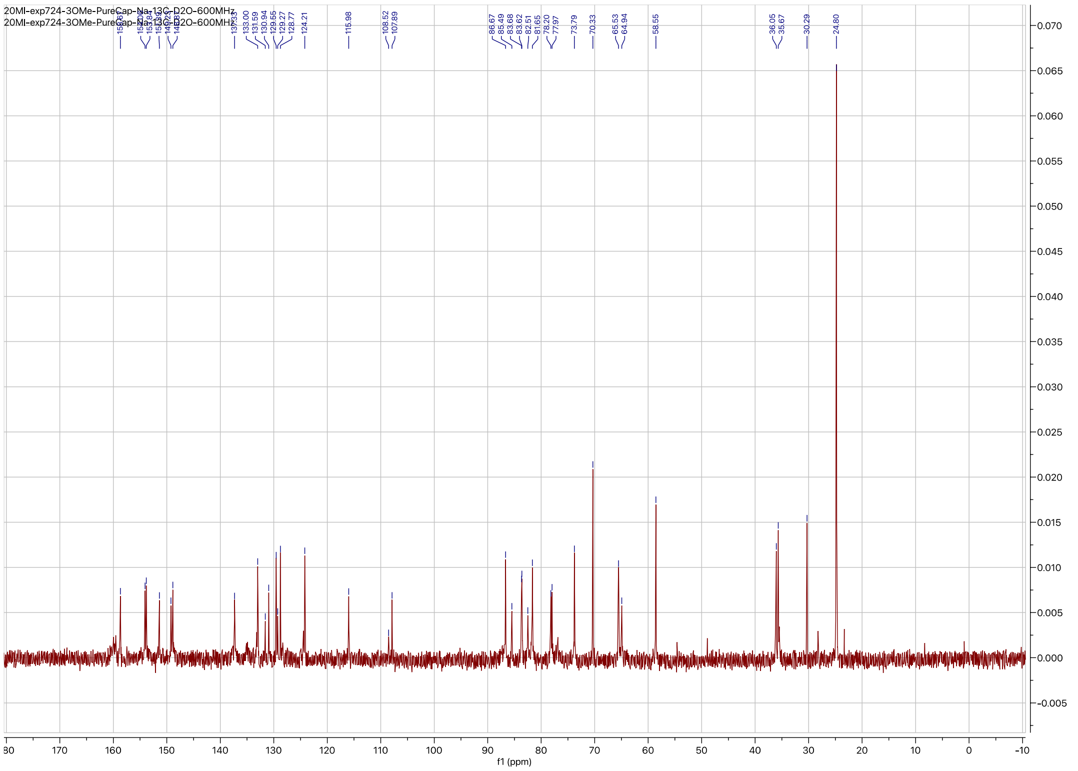

$^{31}P\{^{1}H\}$ NMR Spectrum of Compound **3**

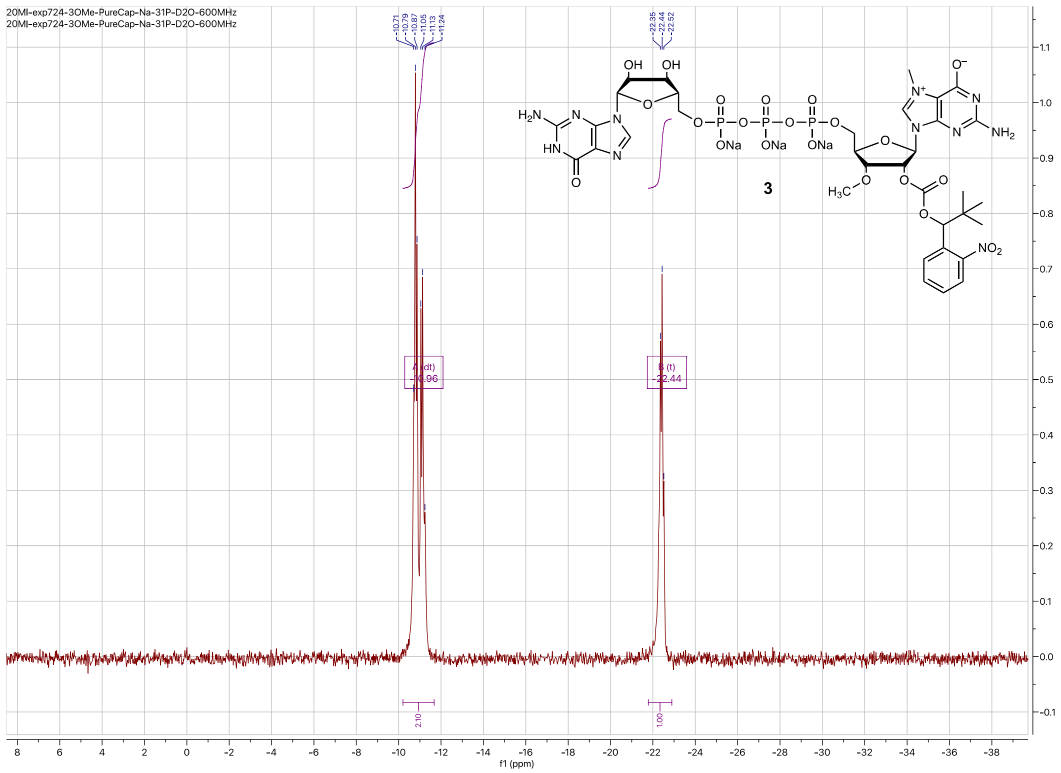

^1H and $^{13}C\{^1H\}$ NMR Spectra of Compound **S27**

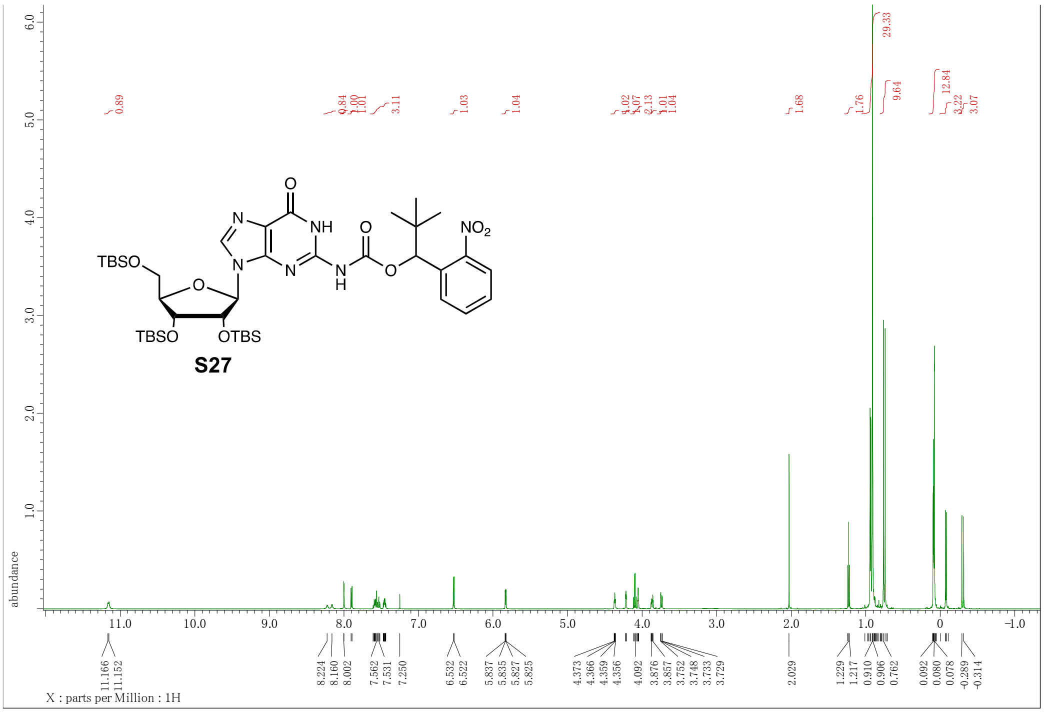

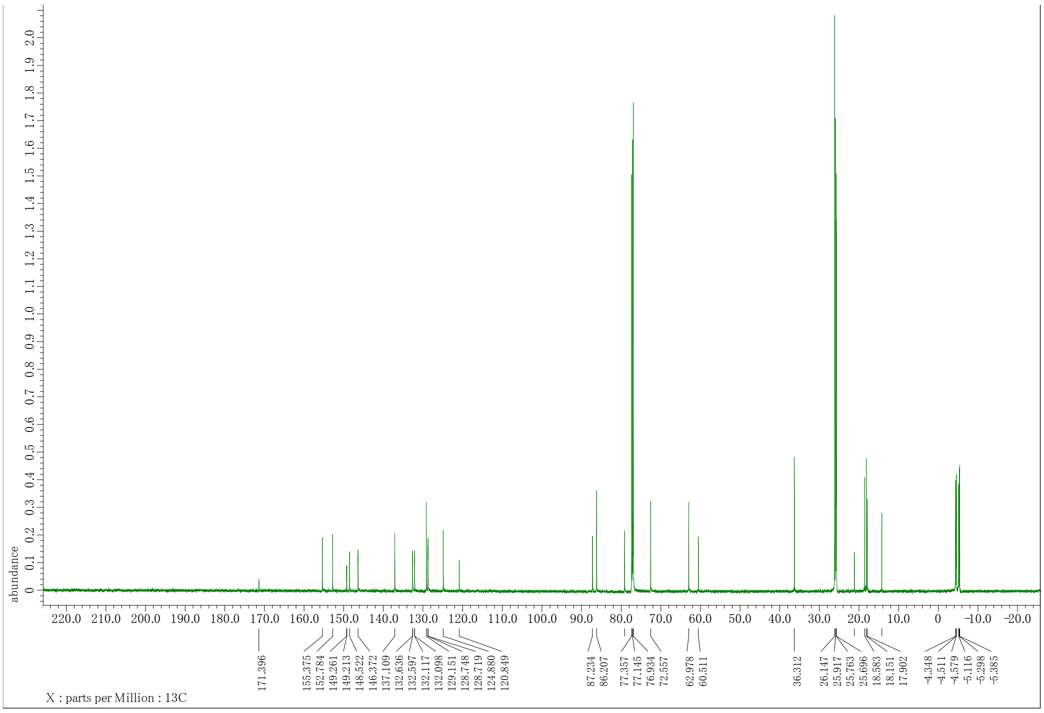

^{1}H and $^{13}C\{^{1}H\}$ NMR Spectra of Compound **S28**

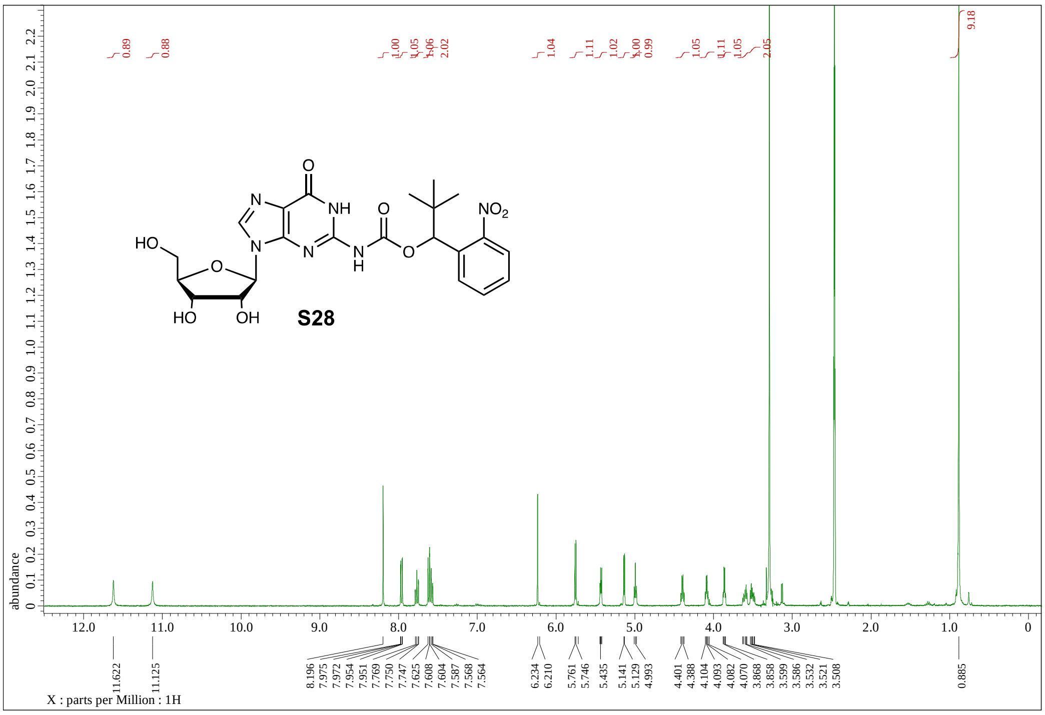

You created this PDF from an application that is not licensed to print to novaPDF printer (http://www.novapdf.com)

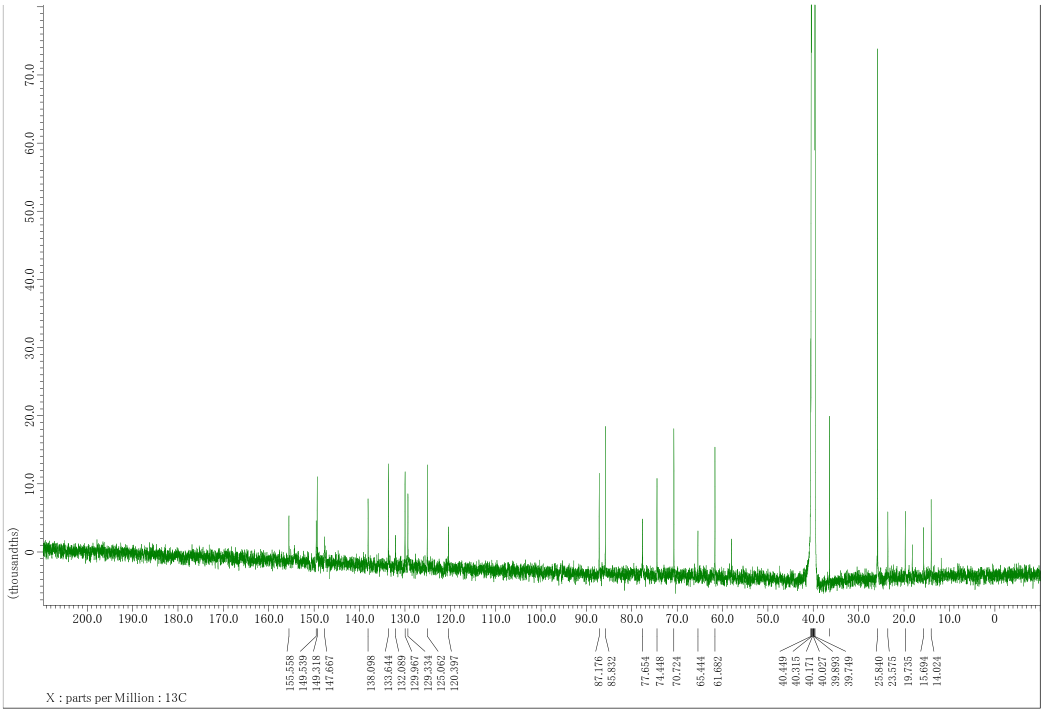

^{1}H and ^{13}C{^{1}H} NMR Spectra of Compound **S30**

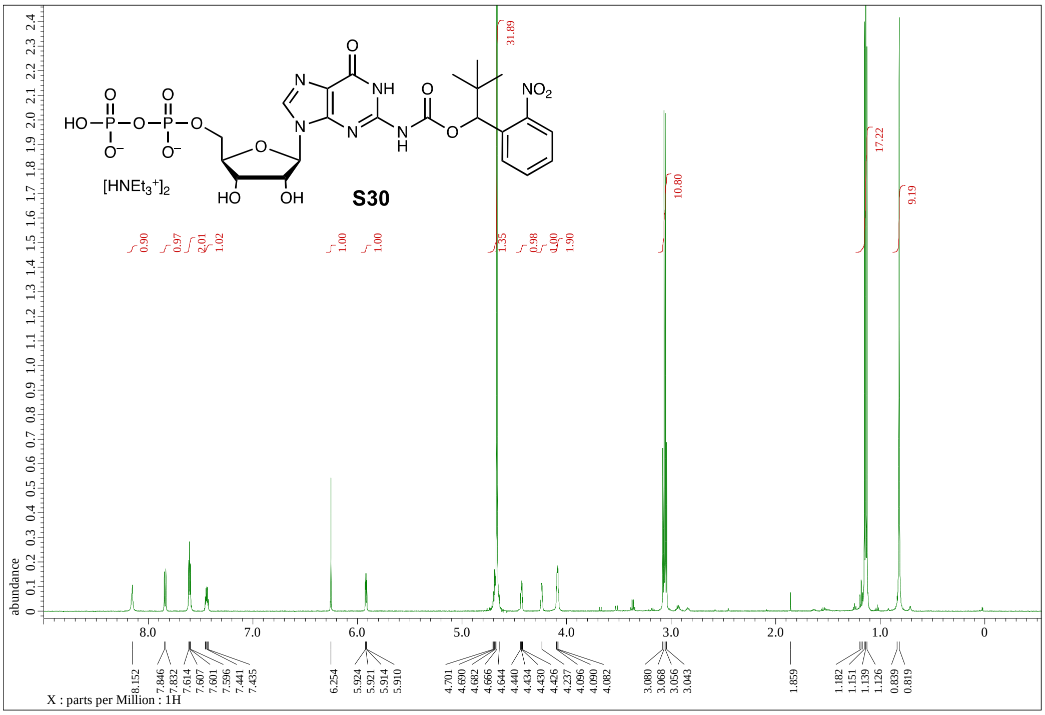

You created this PDF from an application that is not licensed to print to novaPDF printer (http://www.novapdf.com)

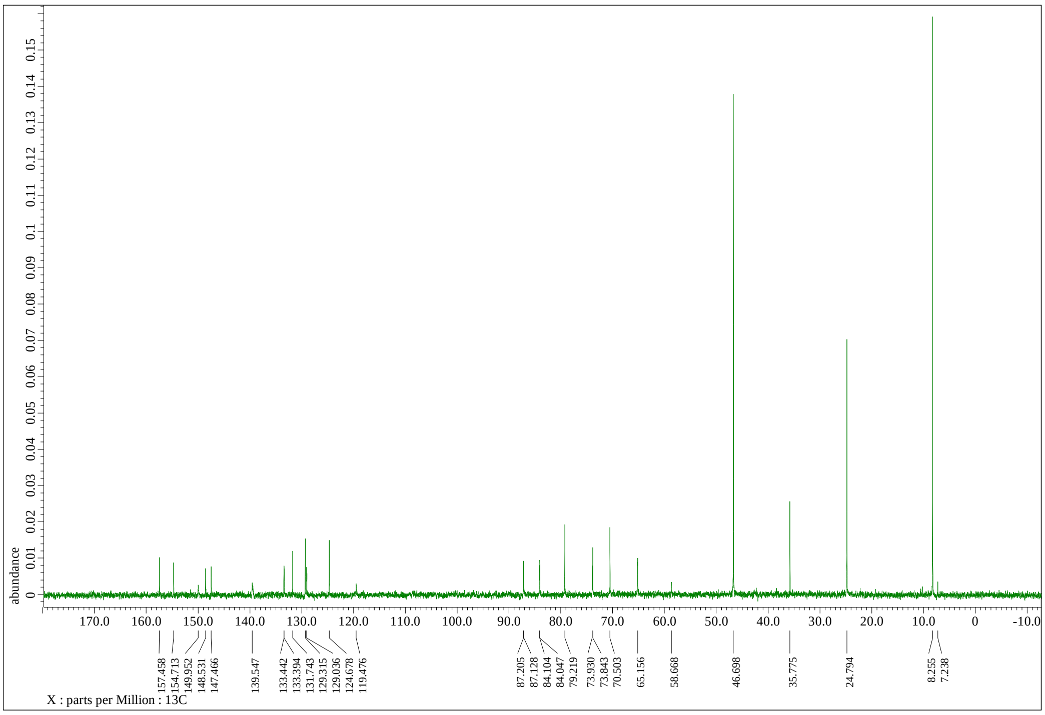

You created this PDF from an application that is not licensed to print to novaPDF printer (http://www.novapdf.com)

$^{31}P\{^{1}H\}$ NMR Spectrum of Compound **S30**

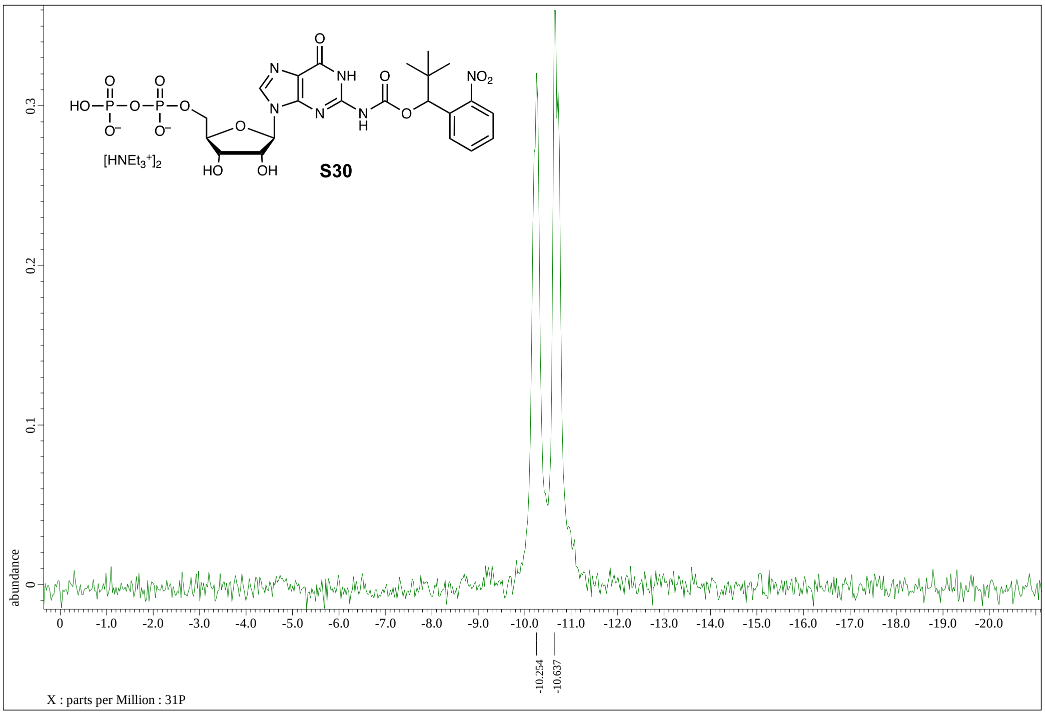

You created this PDF from an application that is not licensed to print to novaPDF printer (http://www.novapdf.com)

^{1}H and ^{13}C{^{1}H} NMR Spectra of Compound **S31**

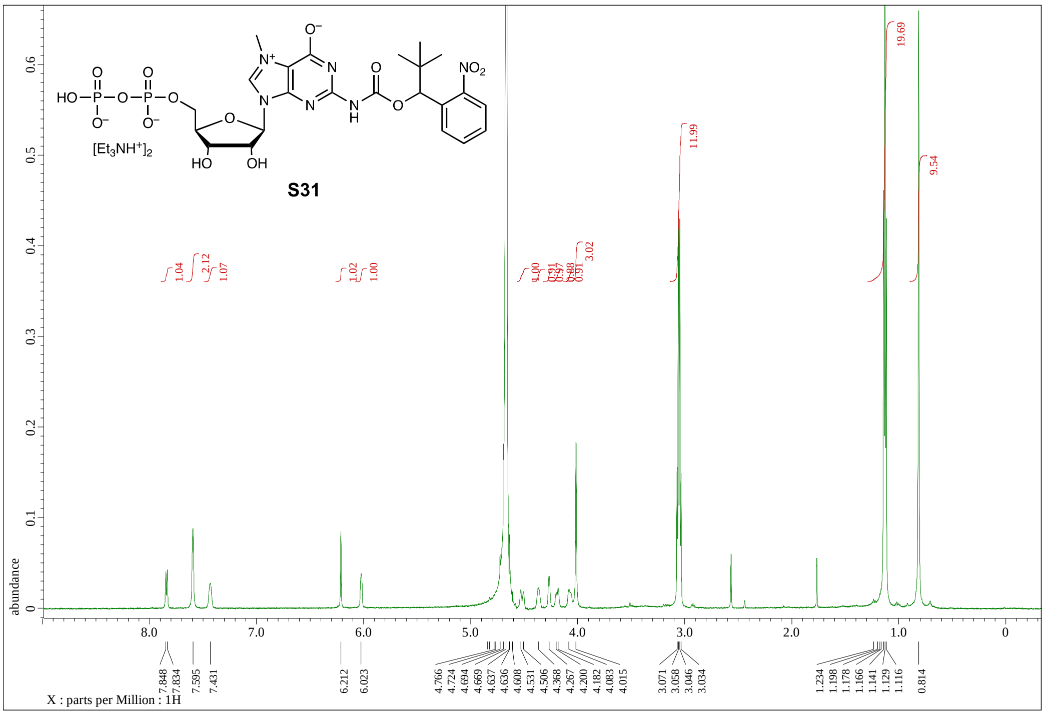

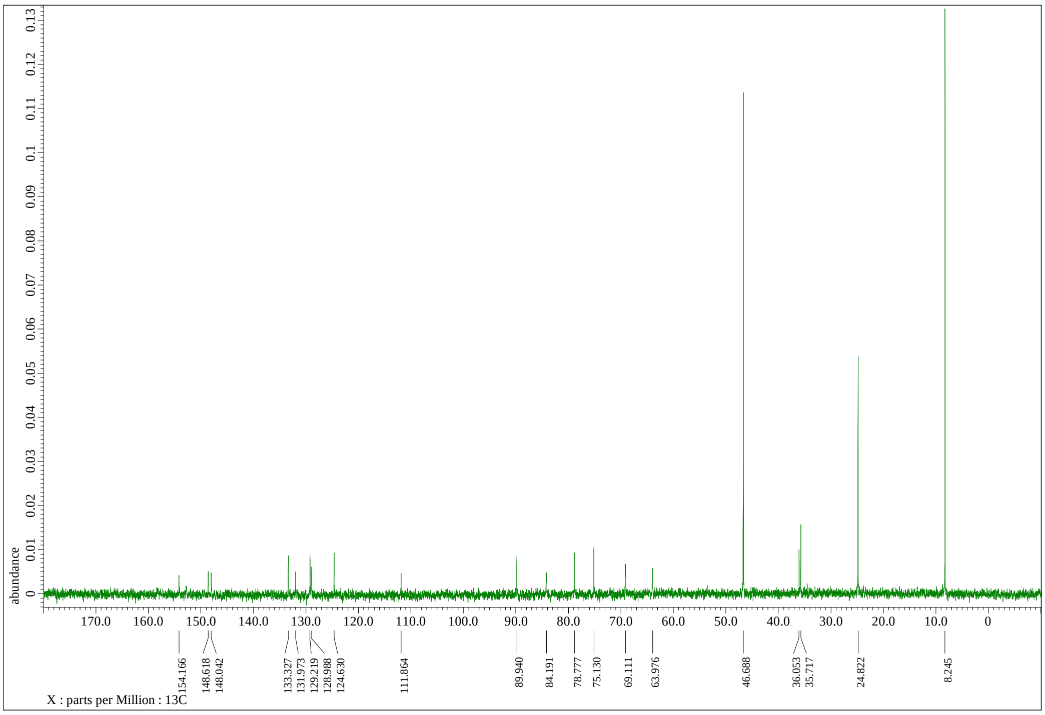

$^{31}P\{^{1}H\}$ NMR Spectrum of Compound **S31**

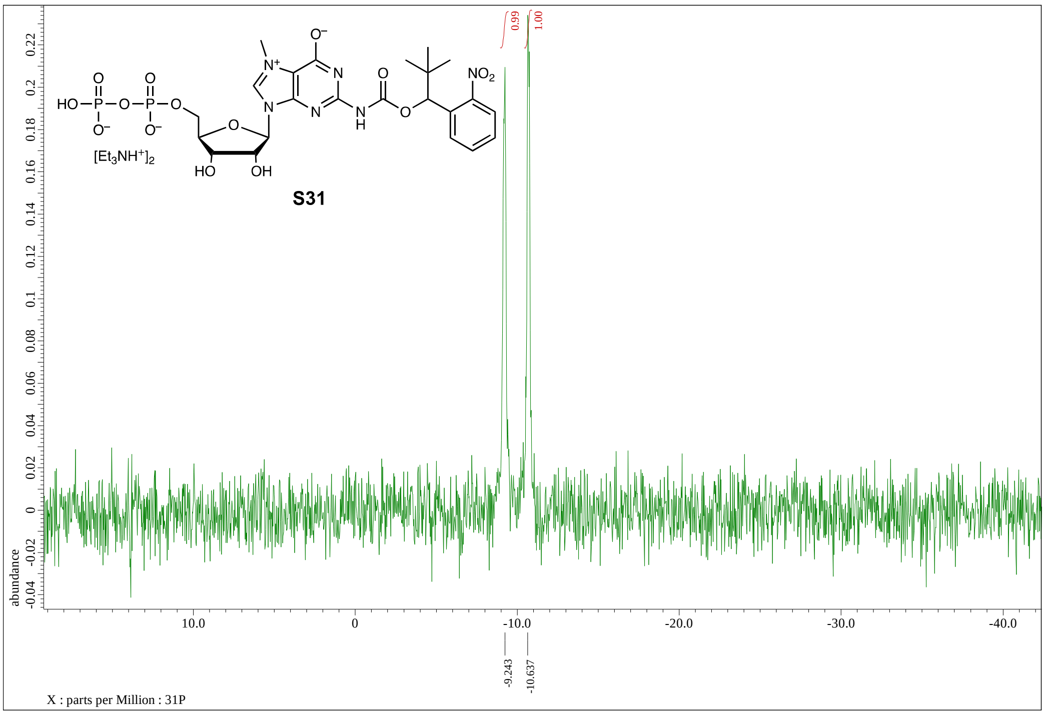

^{1}H and $^{13}C\{^{1}H\}$ NMR Spectra of Compound **4**

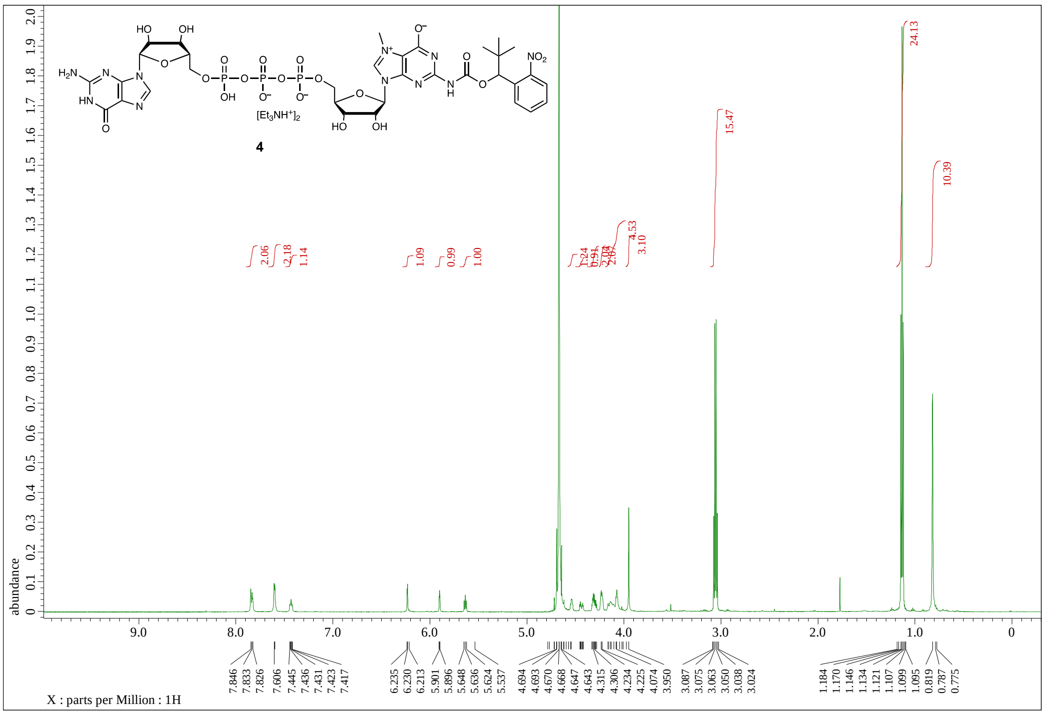

You created this PDF from an application that is not licensed to print to novaPDF printer (http://www.novapdf.com)

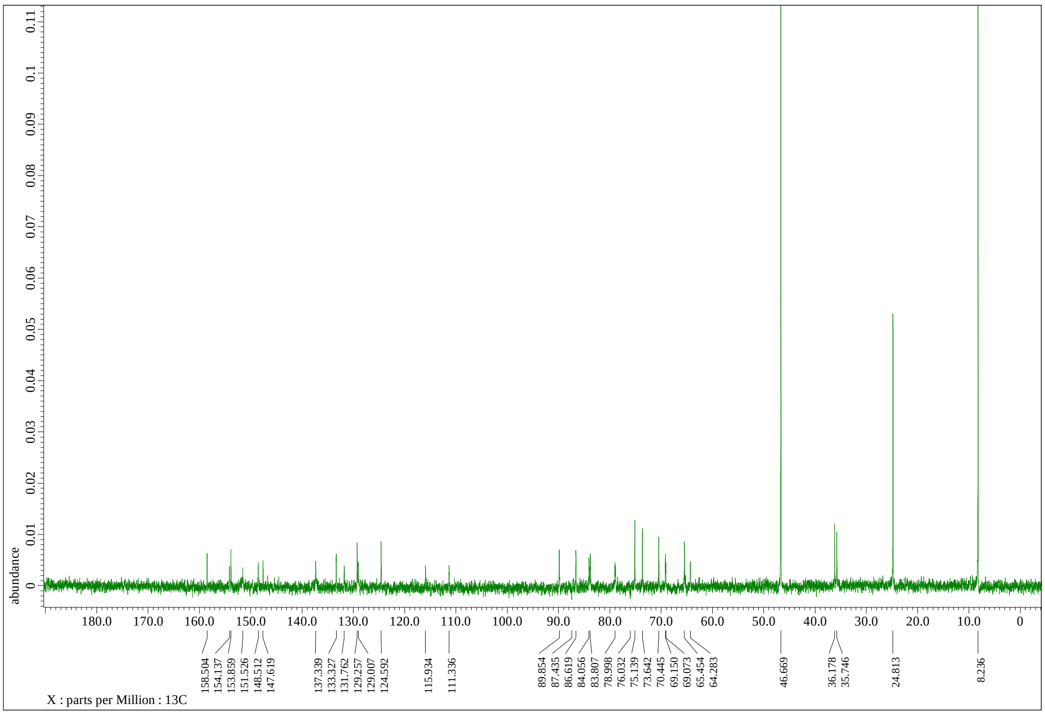

You created this PDF from an application that is not licensed to print to novaPDF printer (http://www.novapdf.com)

$^{31}P\{^{1}H\}$ NMR Spectrum of Compound **4**

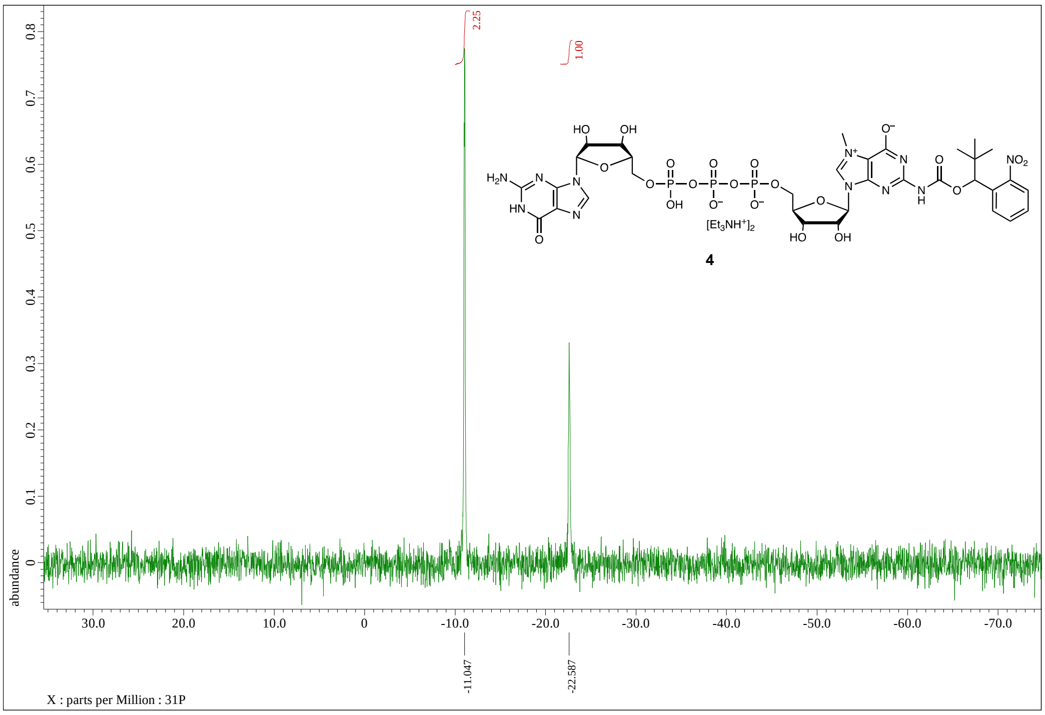

You created this PDF from an application that is not licensed to print to novaPDF printer (http://www.novapdf.com)

^{1}H and $^{31}P\{^{1}H\}$ NMR Spectra of Compound **19**

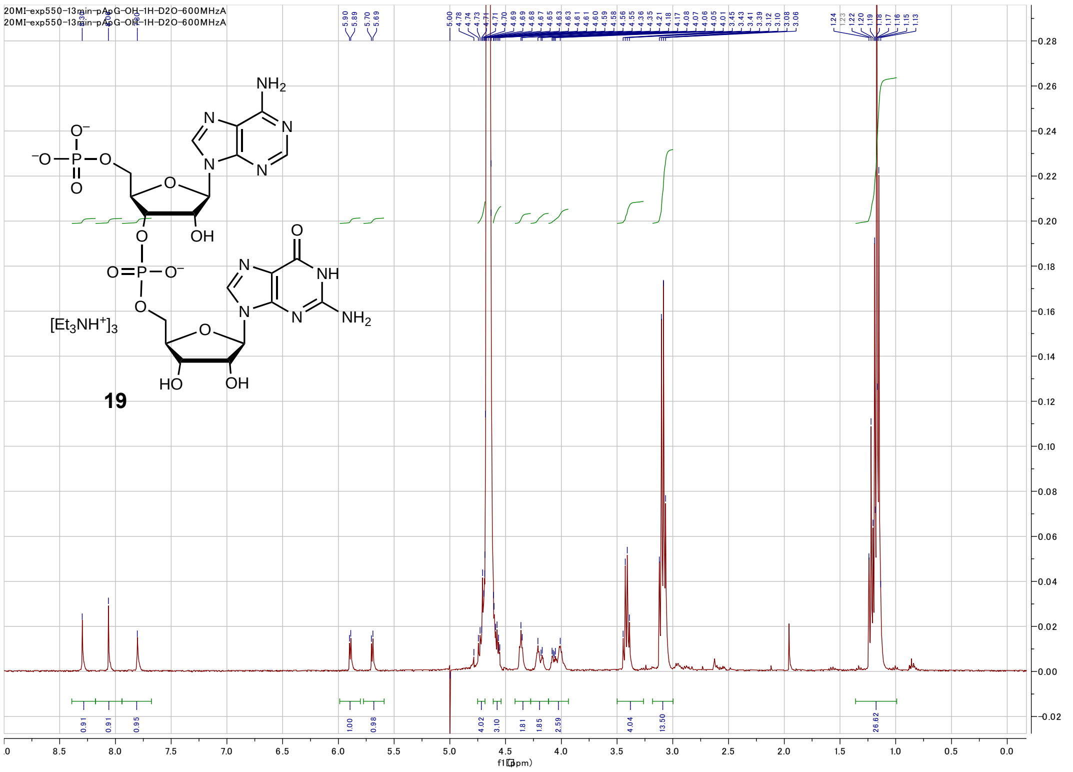

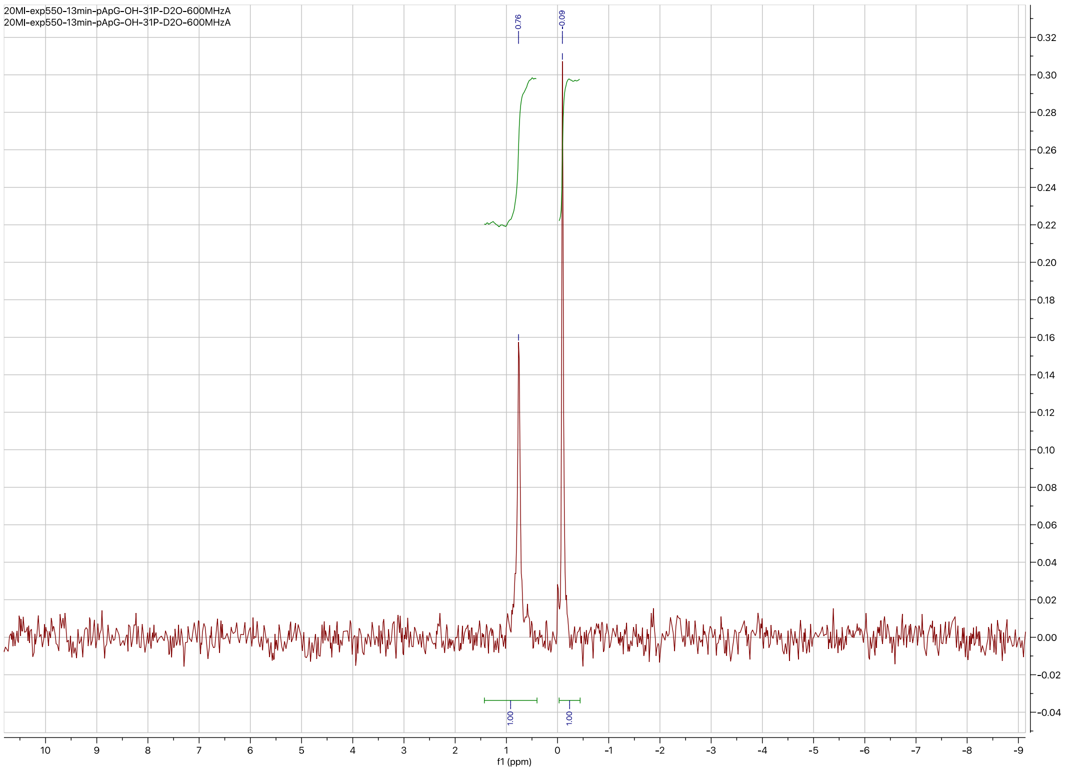

^{1}H and ^{13}C{^{1}H} NMR Spectra of Compound **20**

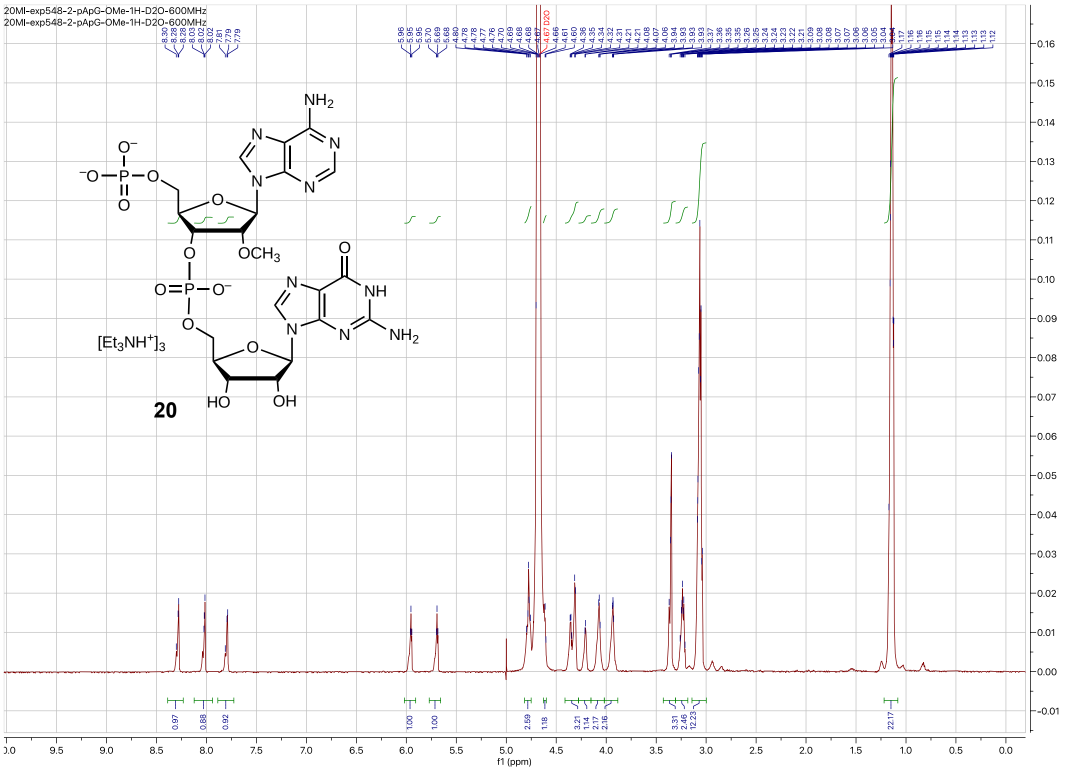

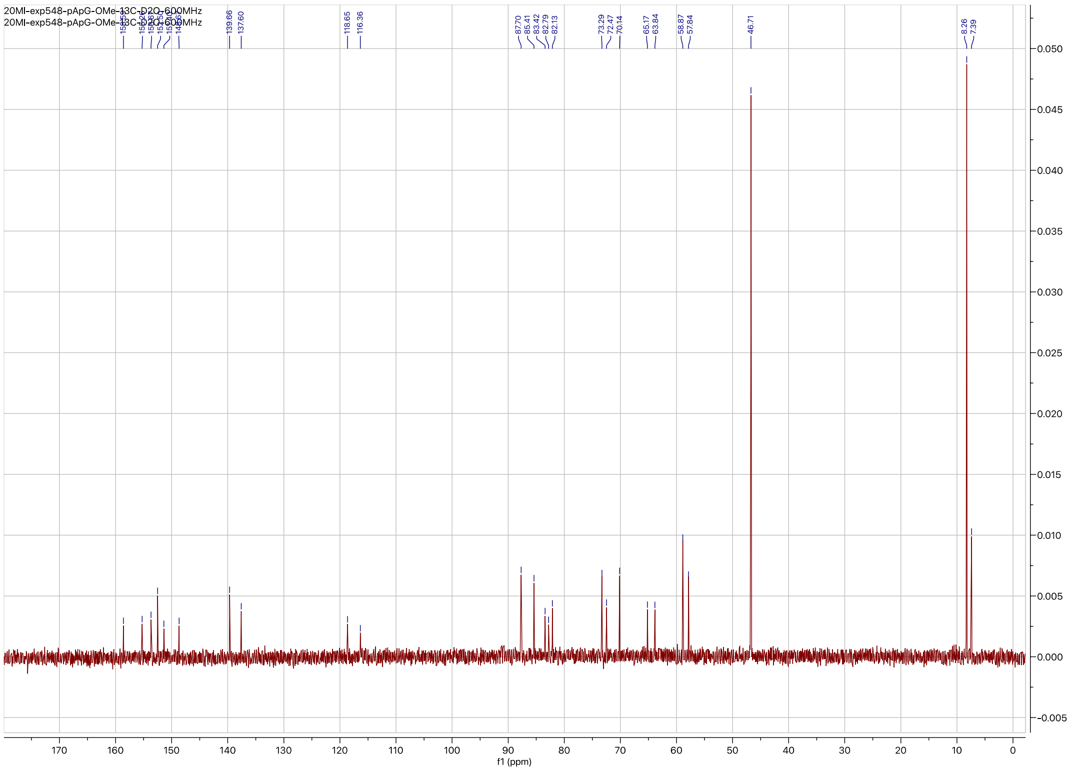

$^{31}P\{^{1}H\}$ NMR Spectrum of Compound **20**

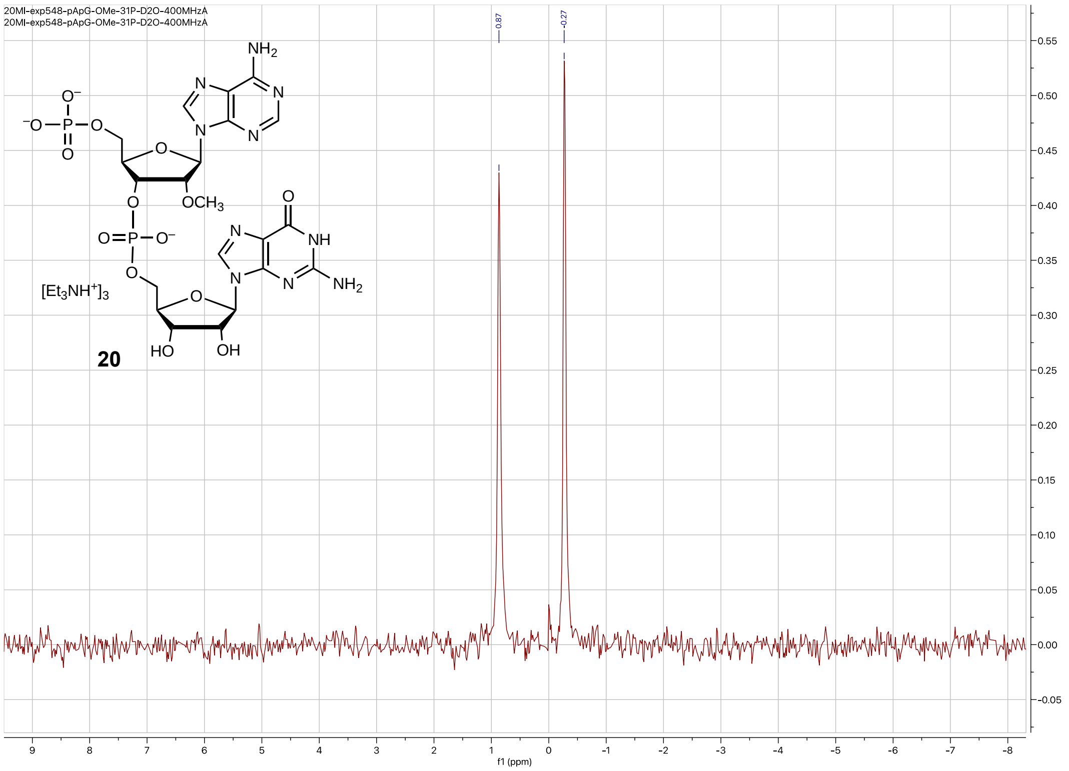

^{1}H and ^{31}P{^{1}H} NMR Spectra of Compound **21**

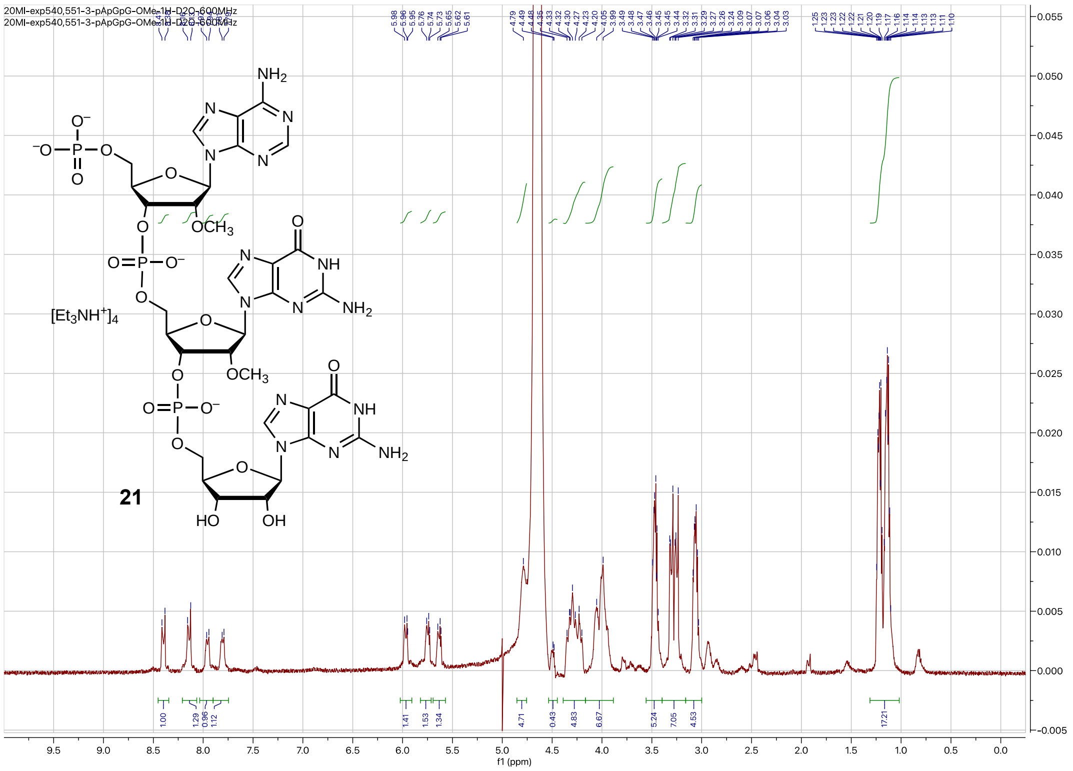

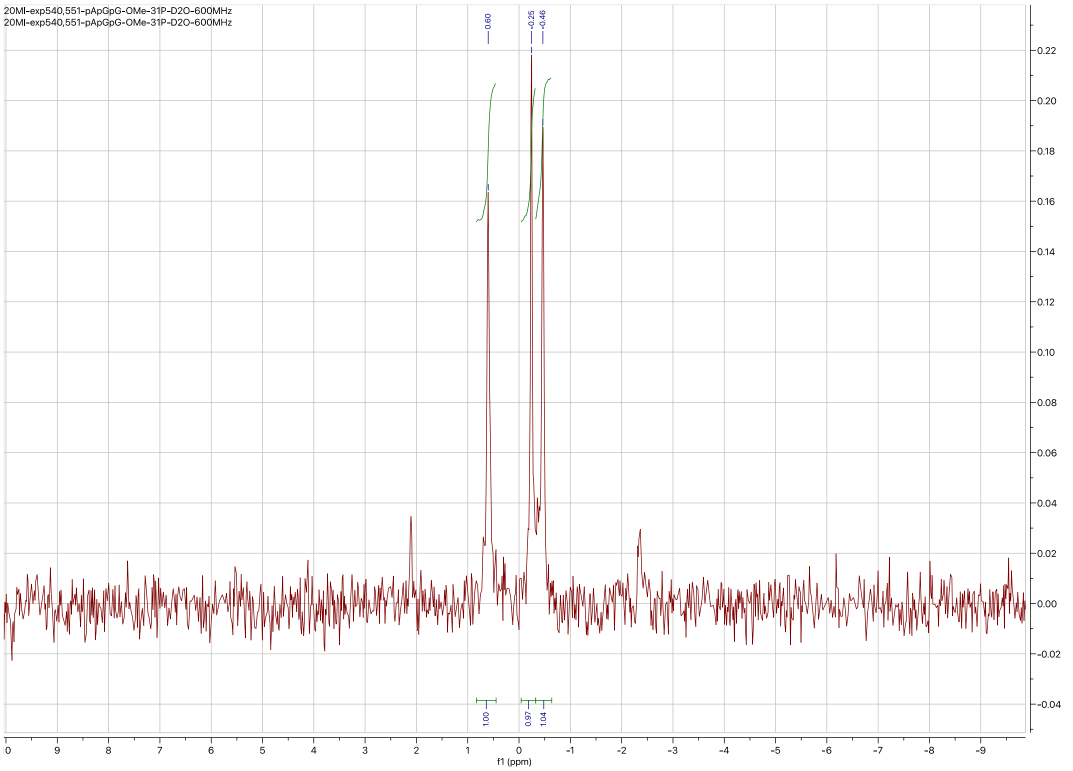

^{1}H and $^{13}C\{^1H\}$ NMR Spectra of Compound **22**

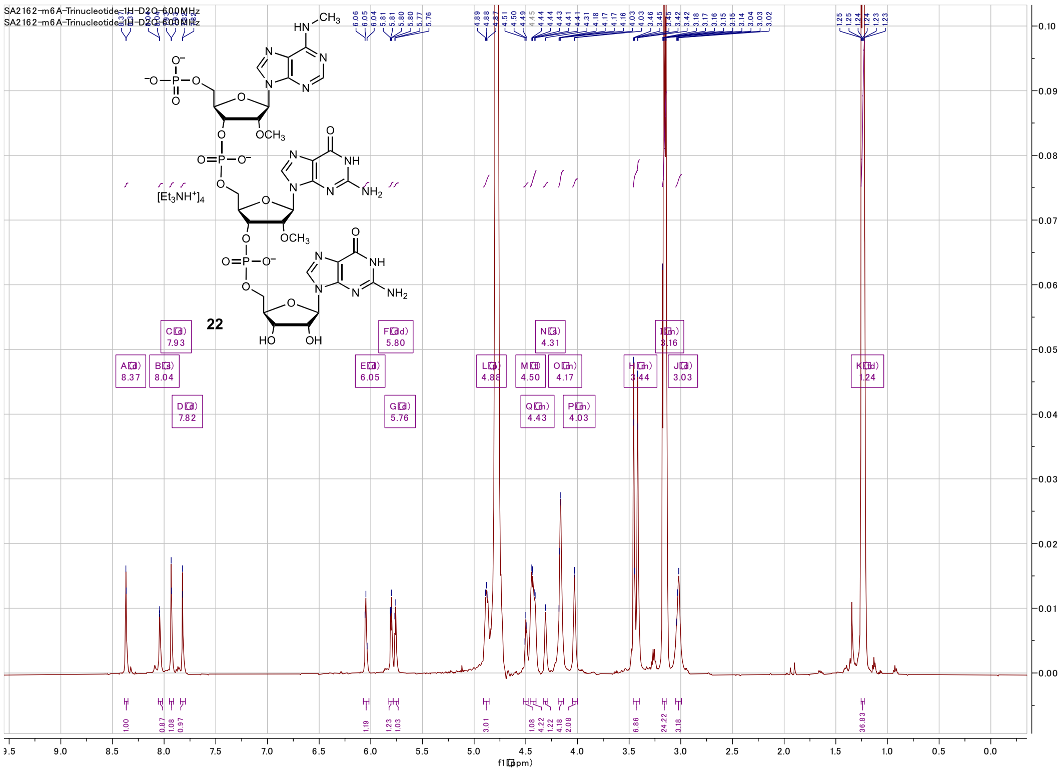

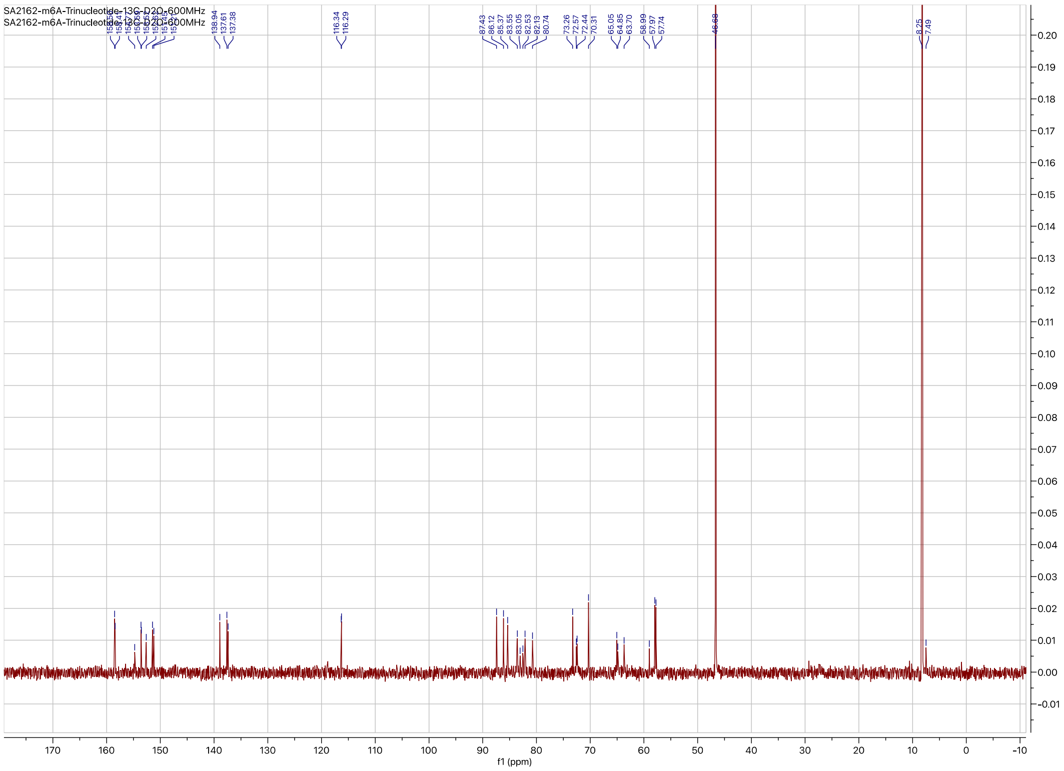

$^{31}P\{^{1}H\}$ NMR Spectrum of Compound **22**

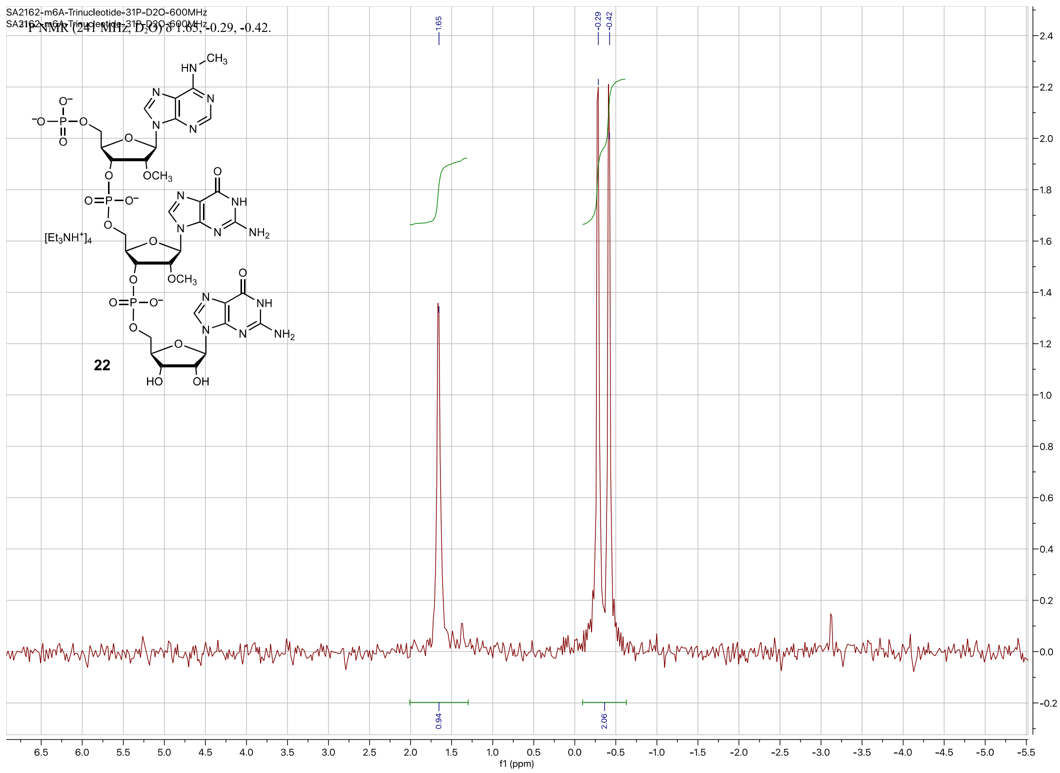

^{1}H and $^{13}C\{^{1}H\}$ NMR Spectra of Compound **5**

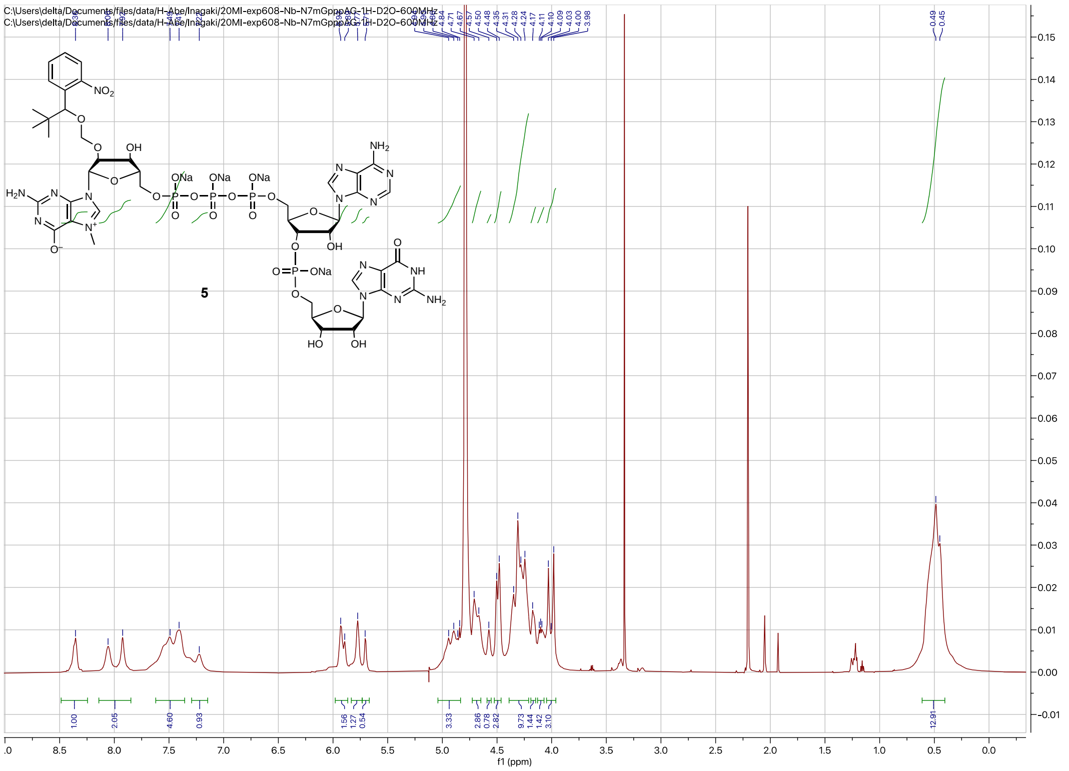

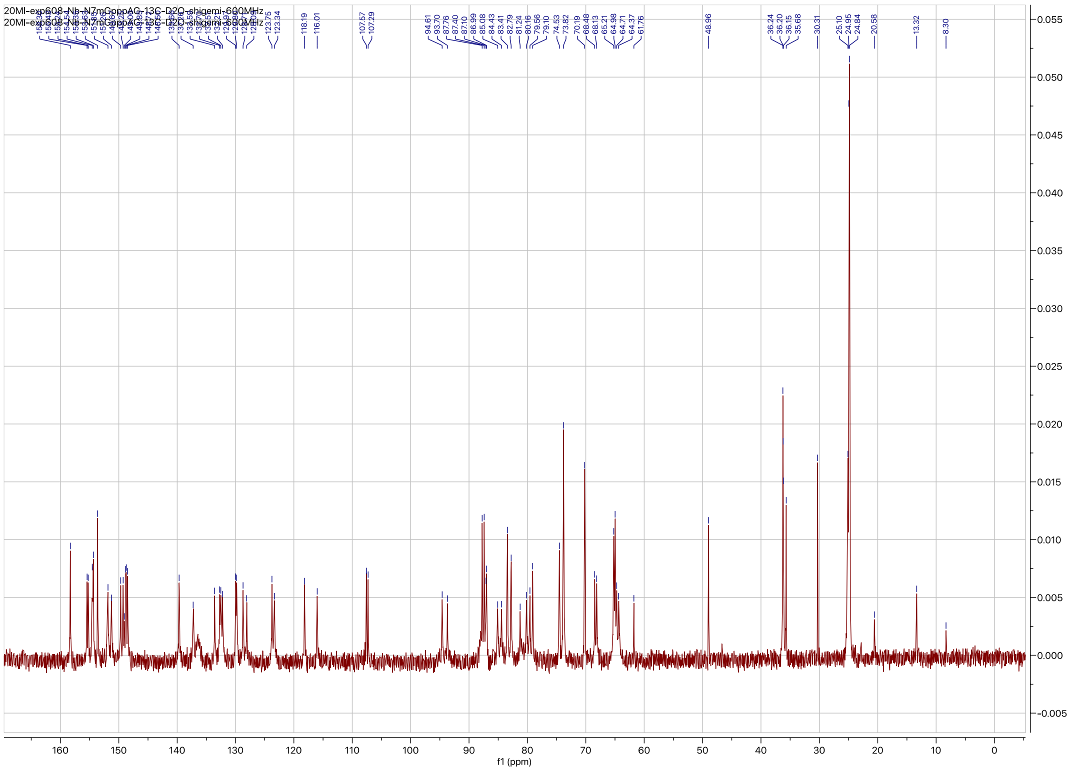

$^{31}P\{^1H\}$ NMR Spectrum of Compound **5**

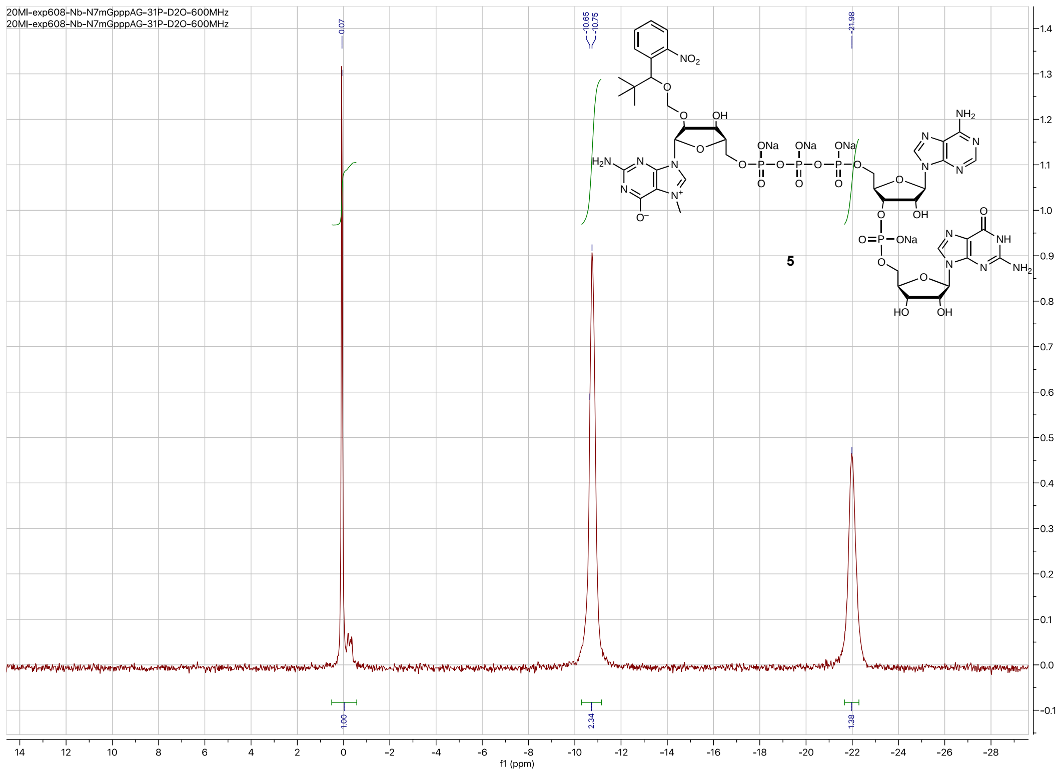

^{1}H and ^{13}C{^{1}H} NMR Spectra of Compound **6**

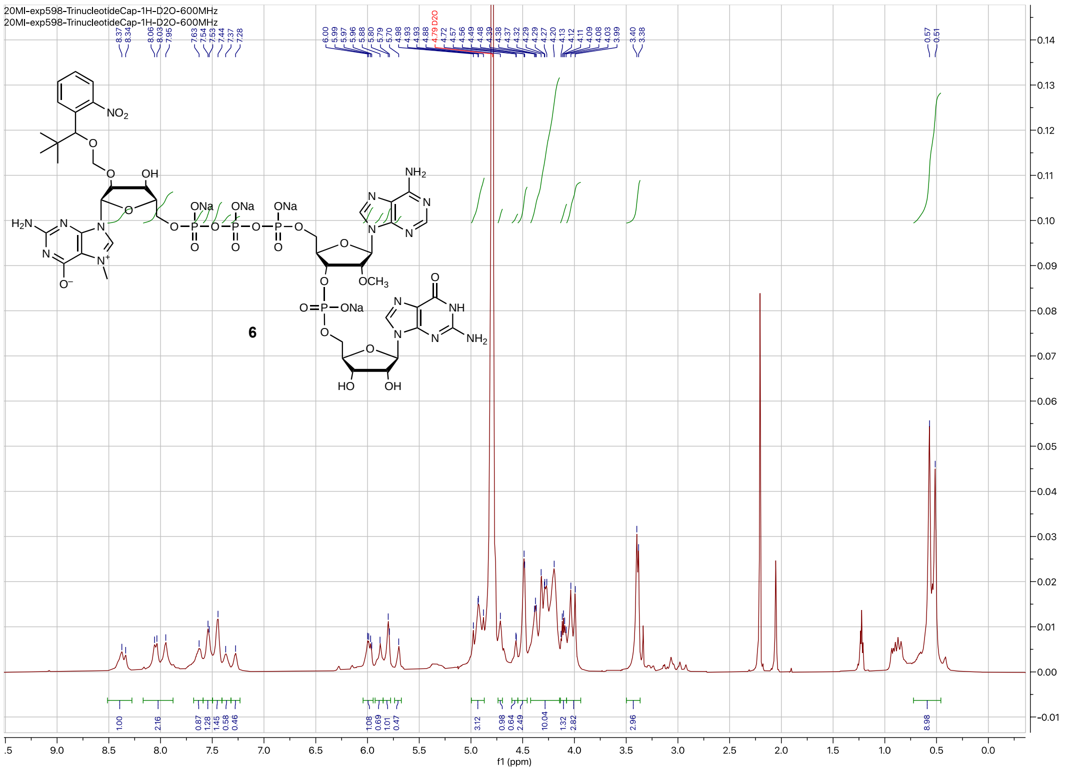

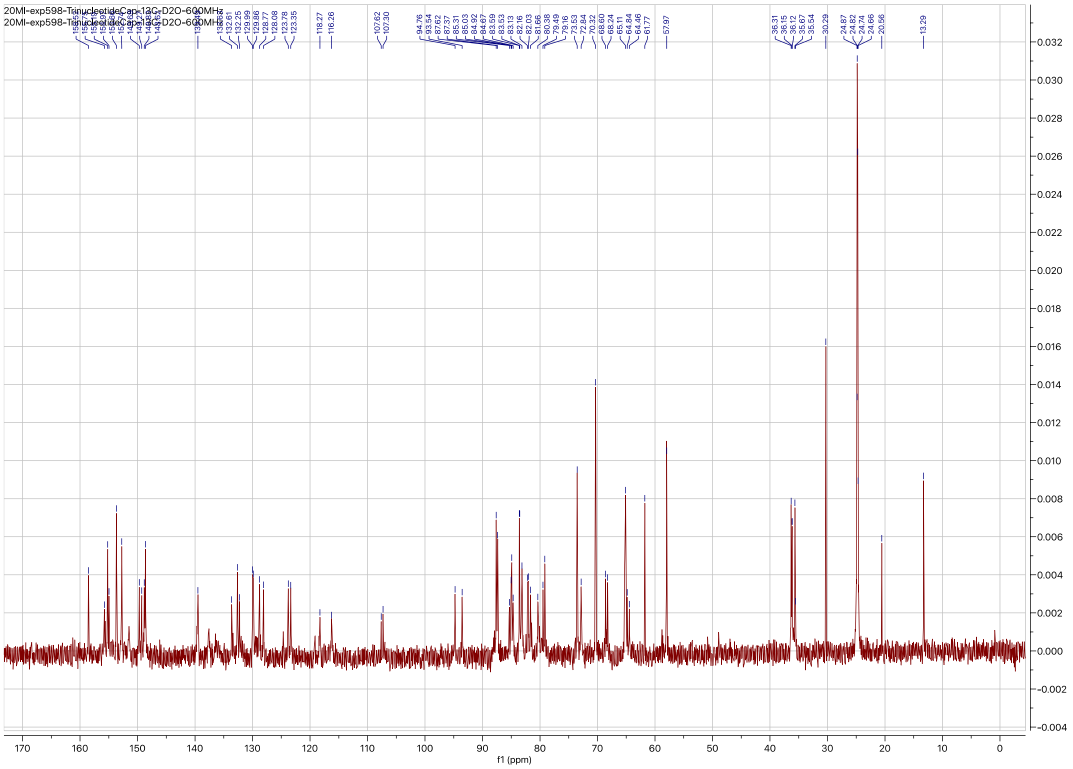

$^{31}P\{^{1}H\}$ NMR Spectrum of Compound **6**

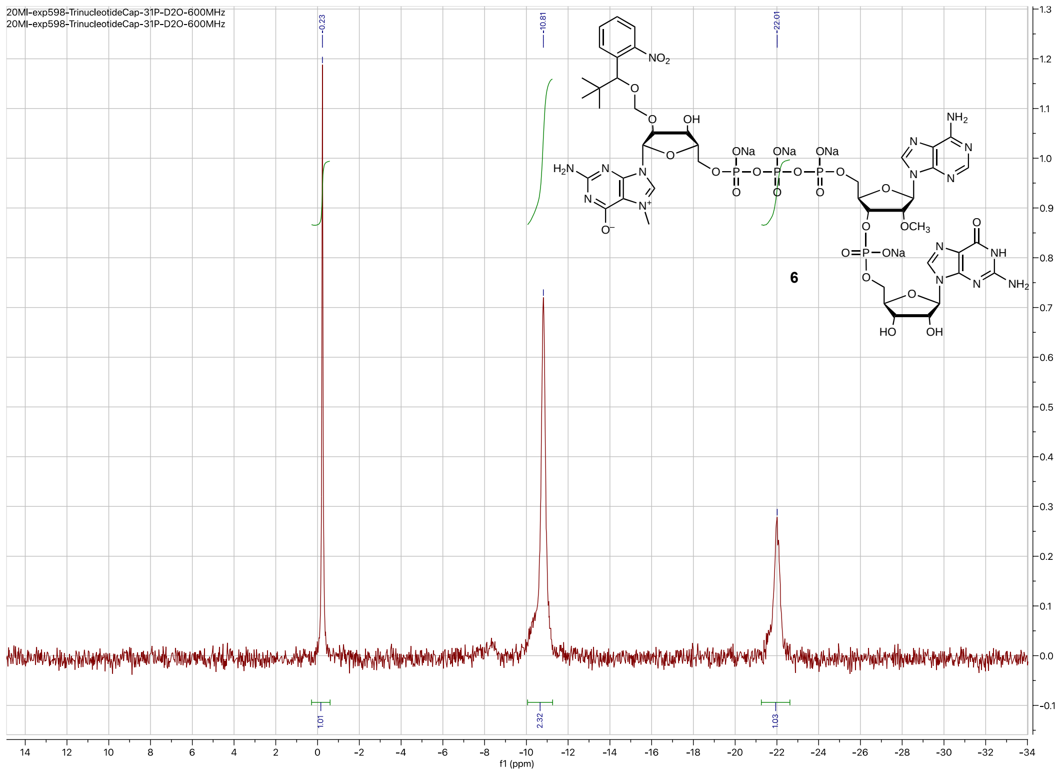

^{1}H and $^{13}C\{^{1}H\}$ NMR Spectra of Compound **7**

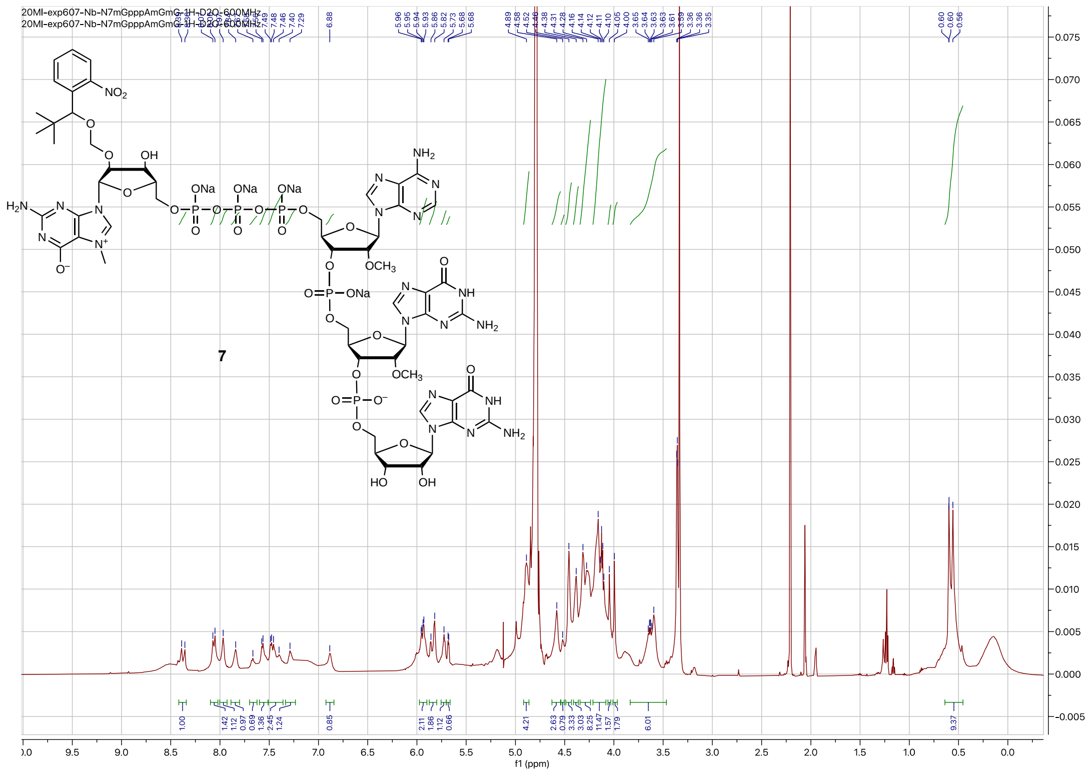

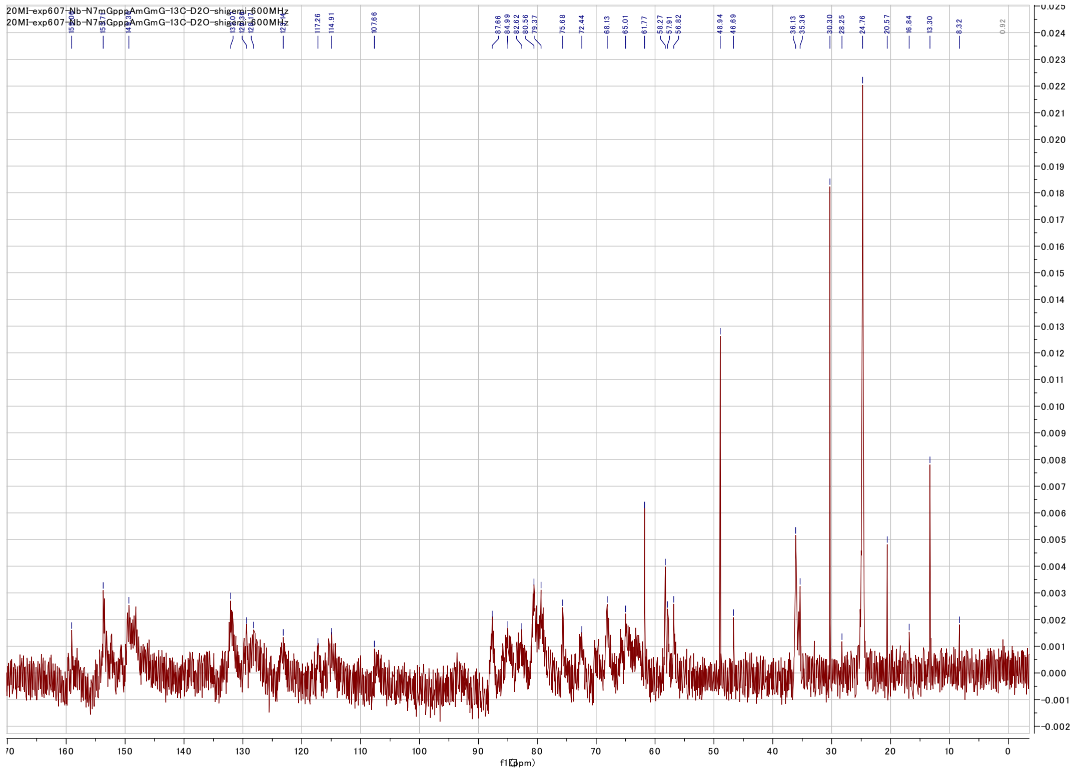

$^{31}P\{^1H\}$ NMR Spectrum of Compound **7**

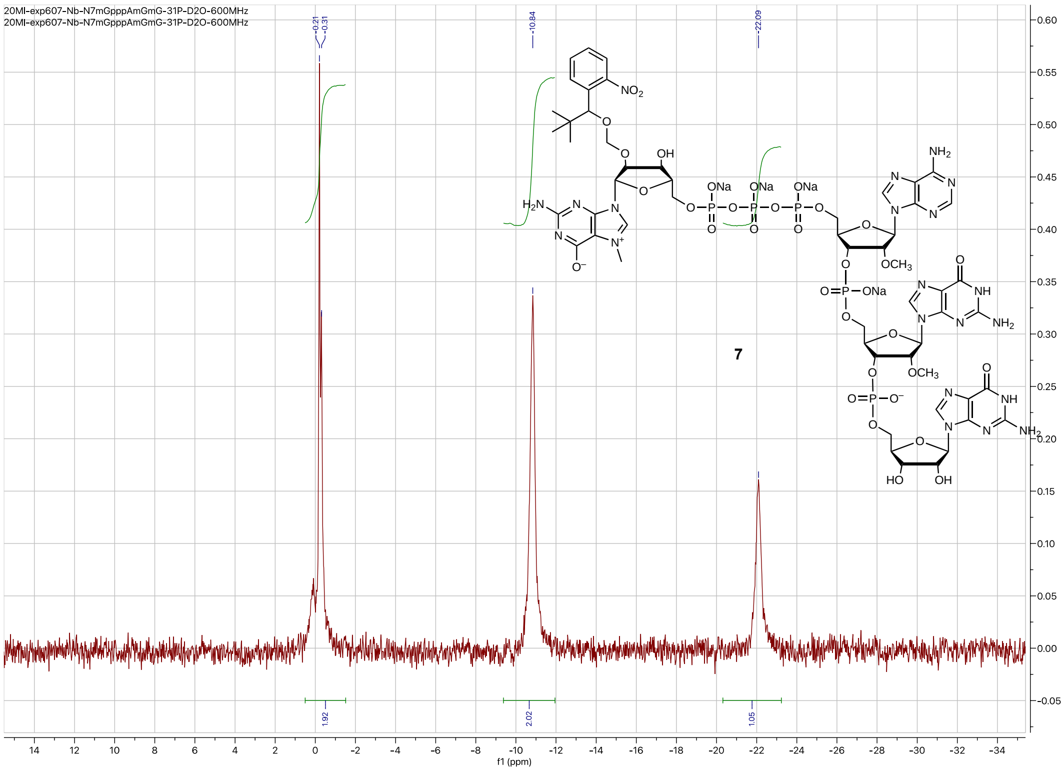

^{1}H and $^{13}C\{^{1}H\}$ NMR Spectra of Compound **8**

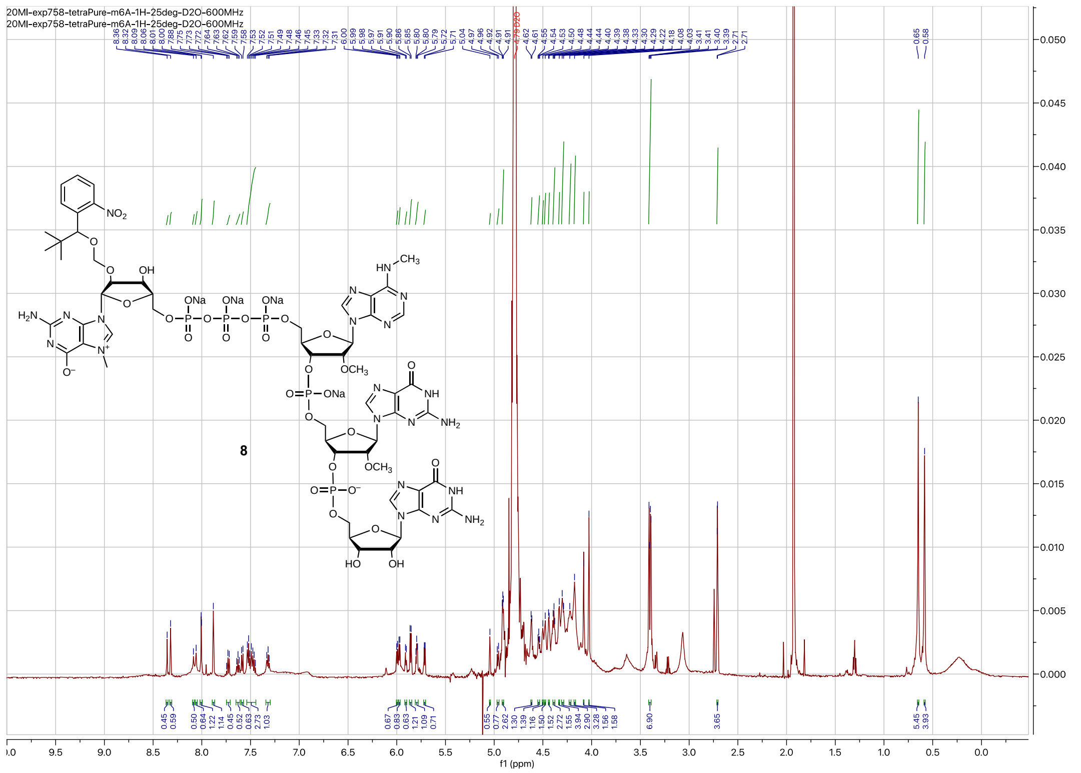

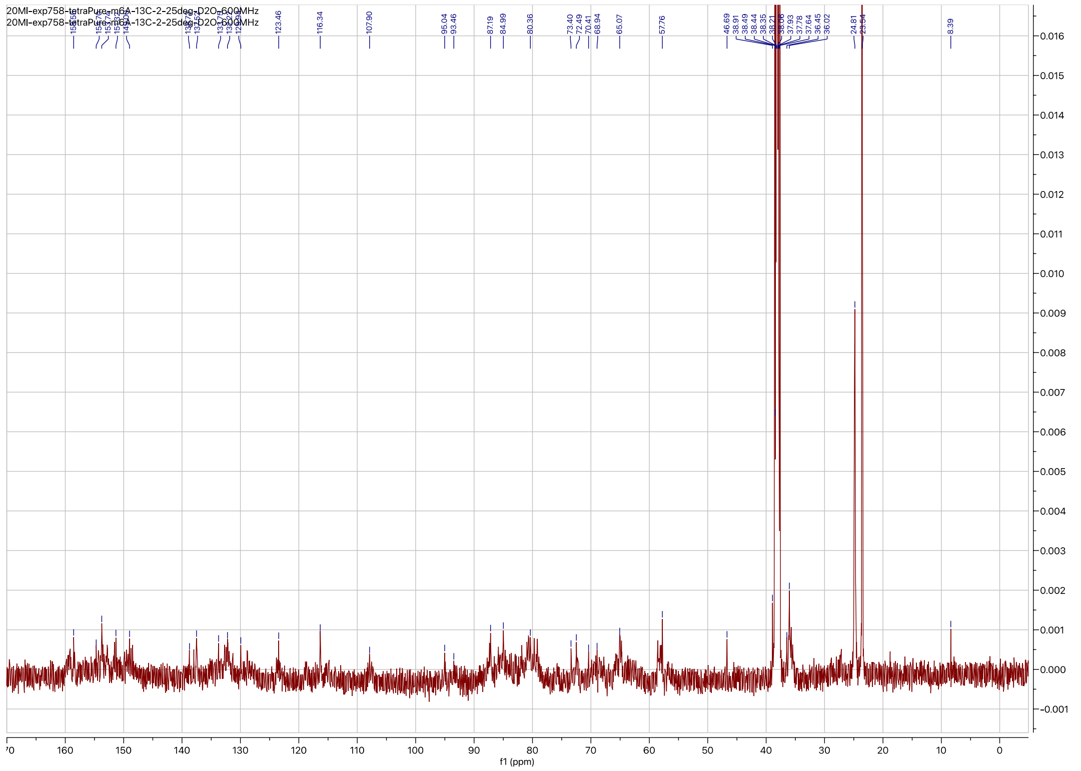

$^{31}P\{^1H\}$ NMR Spectrum of Compound **8**

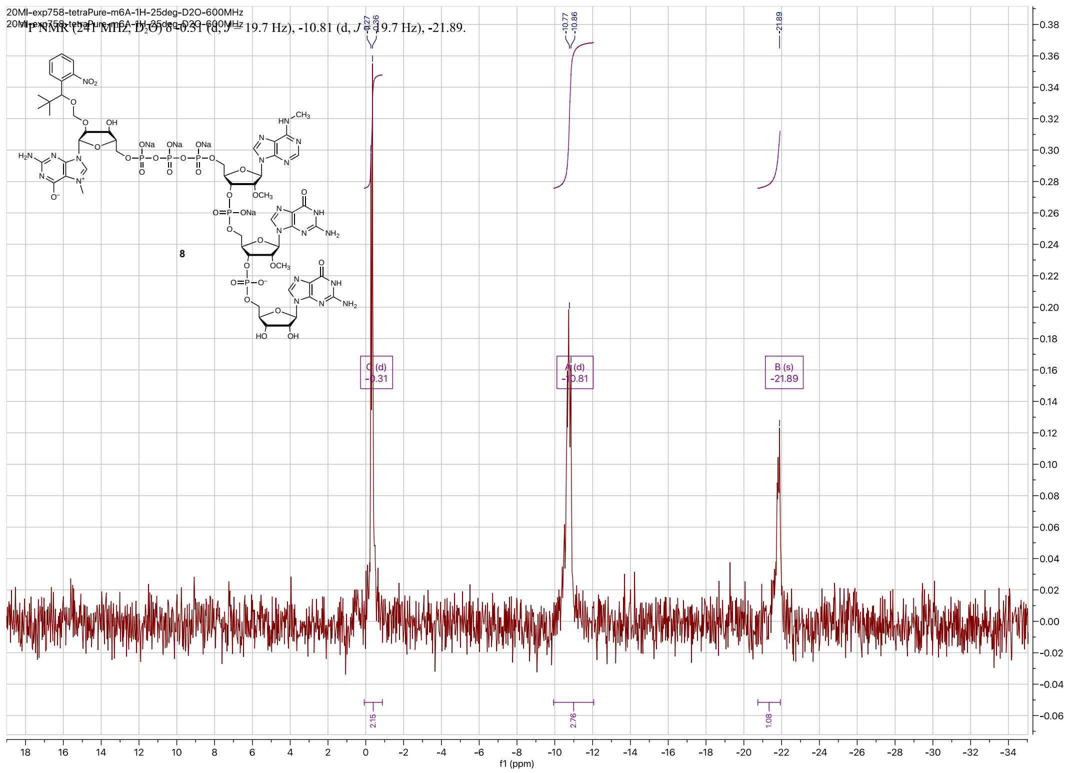

^{1}H and $^{13}C\{^{1}H\}$ NMR Spectra of Compound **9**

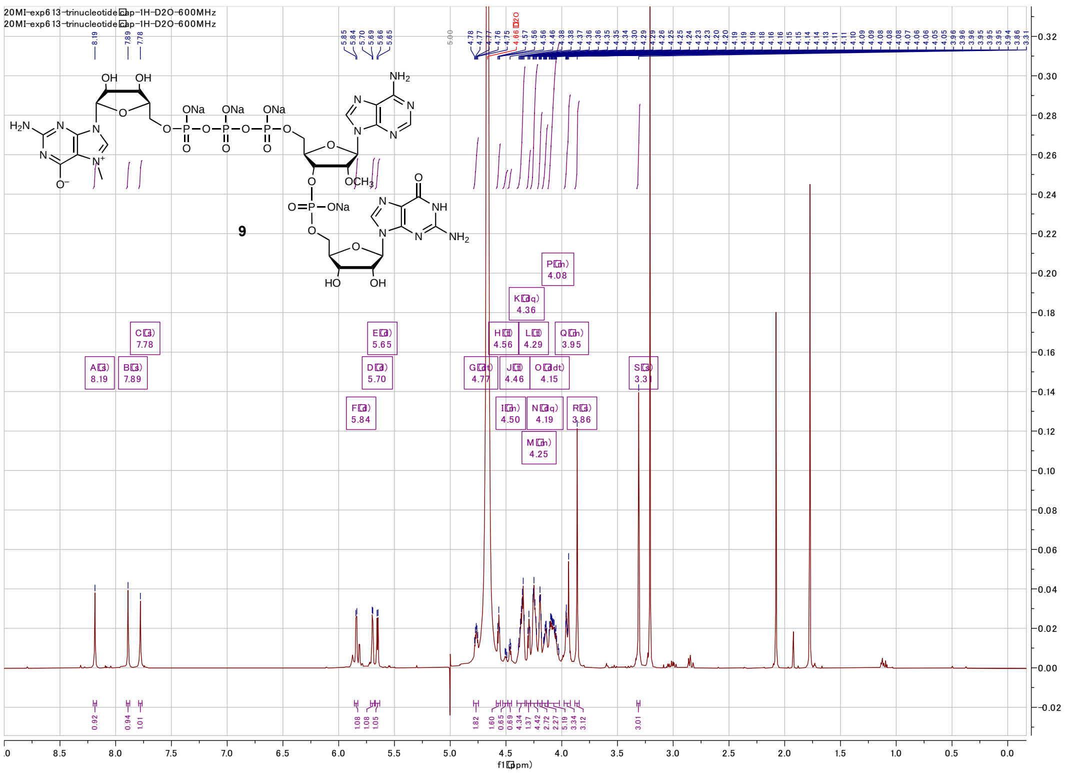

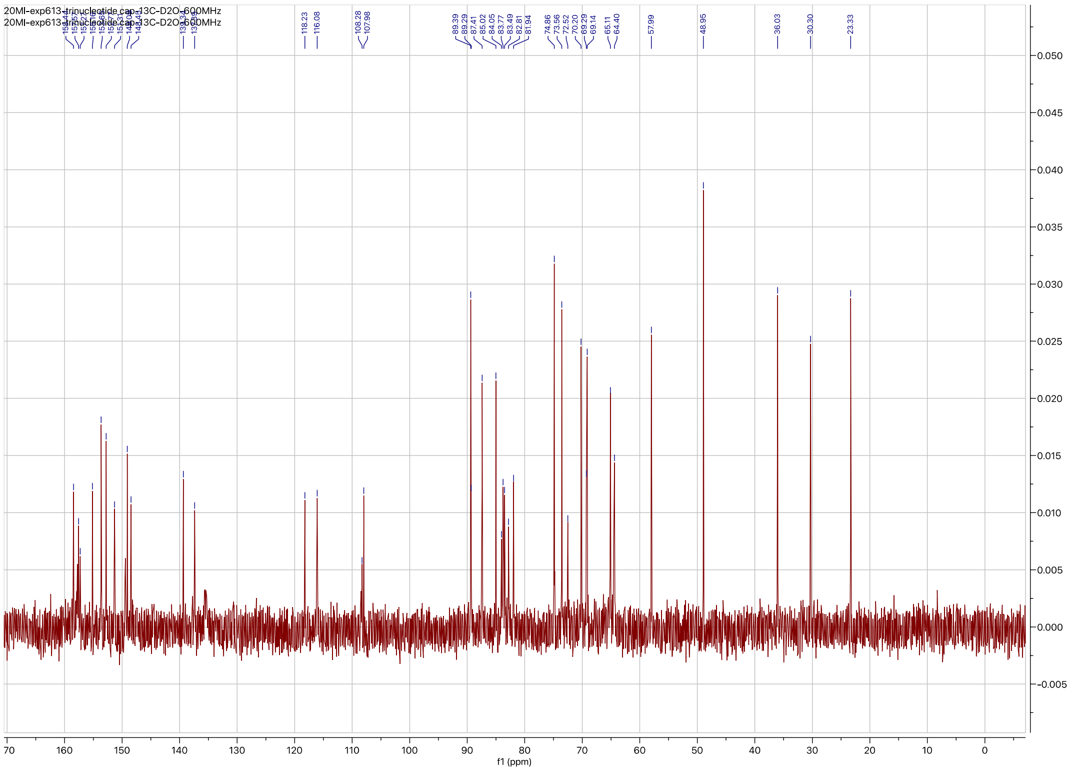

$^{31}P\{^1H\}$ NMR Spectrum of Compound **9**

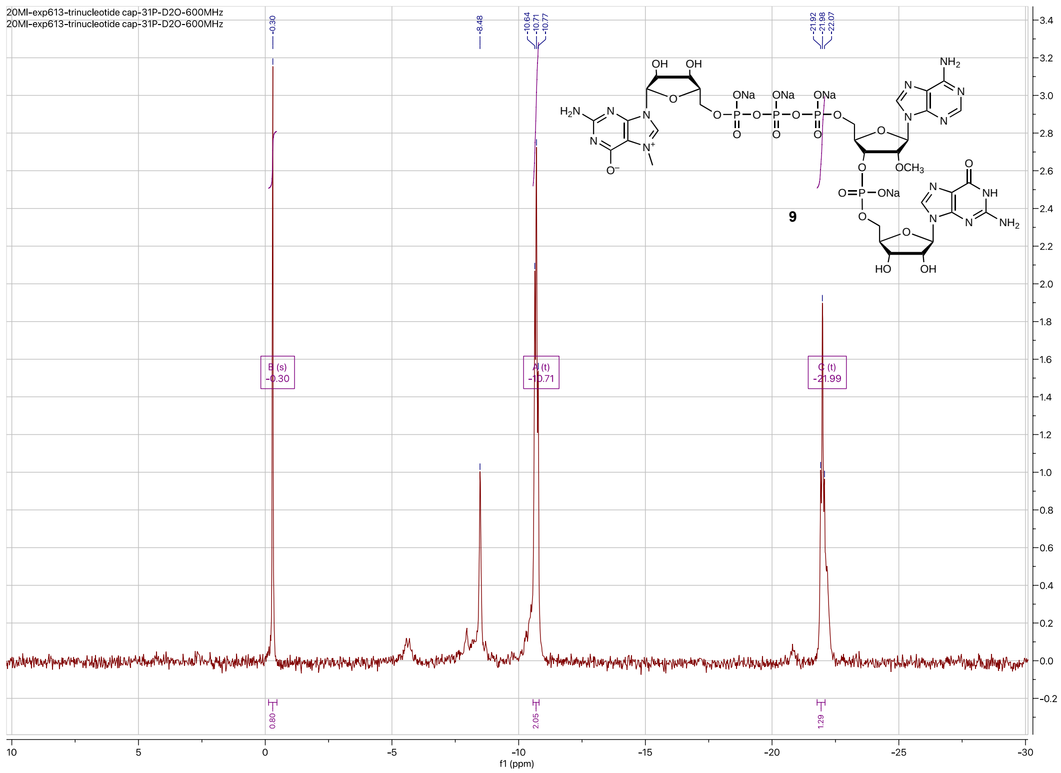

^{1}H and ^{13}C{^{1}H} NMR Spectra of Compound **10**

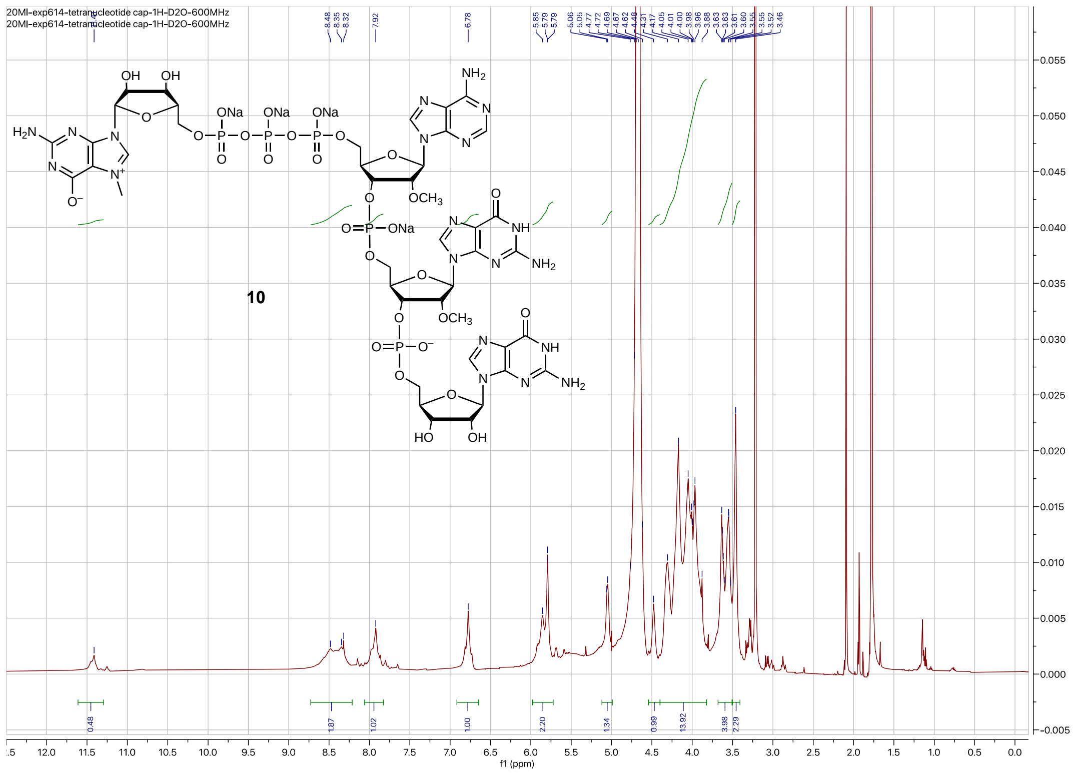

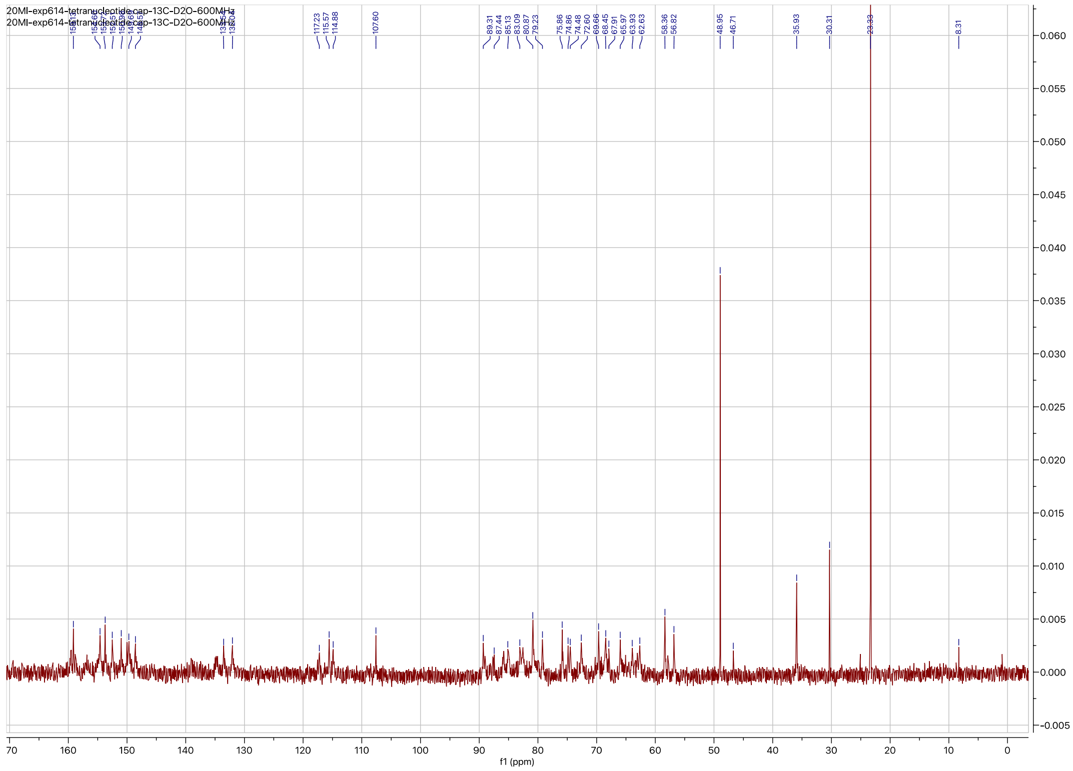

$^{31}P\{^1H\}$ NMR Spectrum of Compound **10**

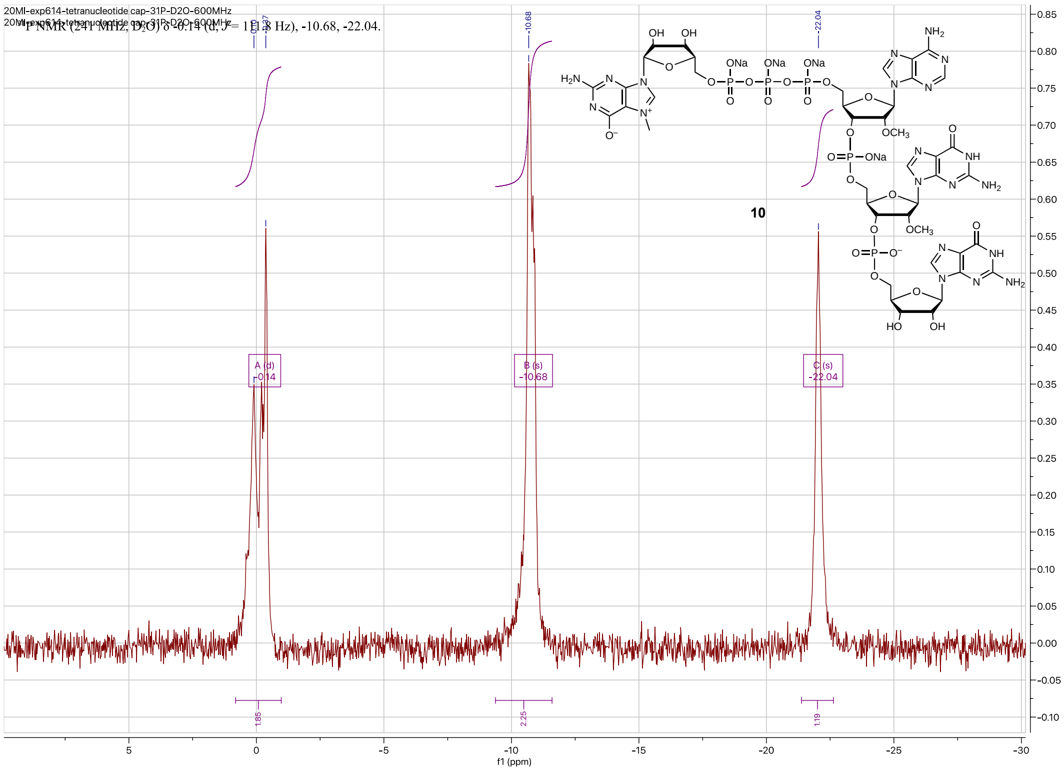

^{1}H and $^{13}C\{^{1}H\}$ NMR Spectra of Compound **S33**

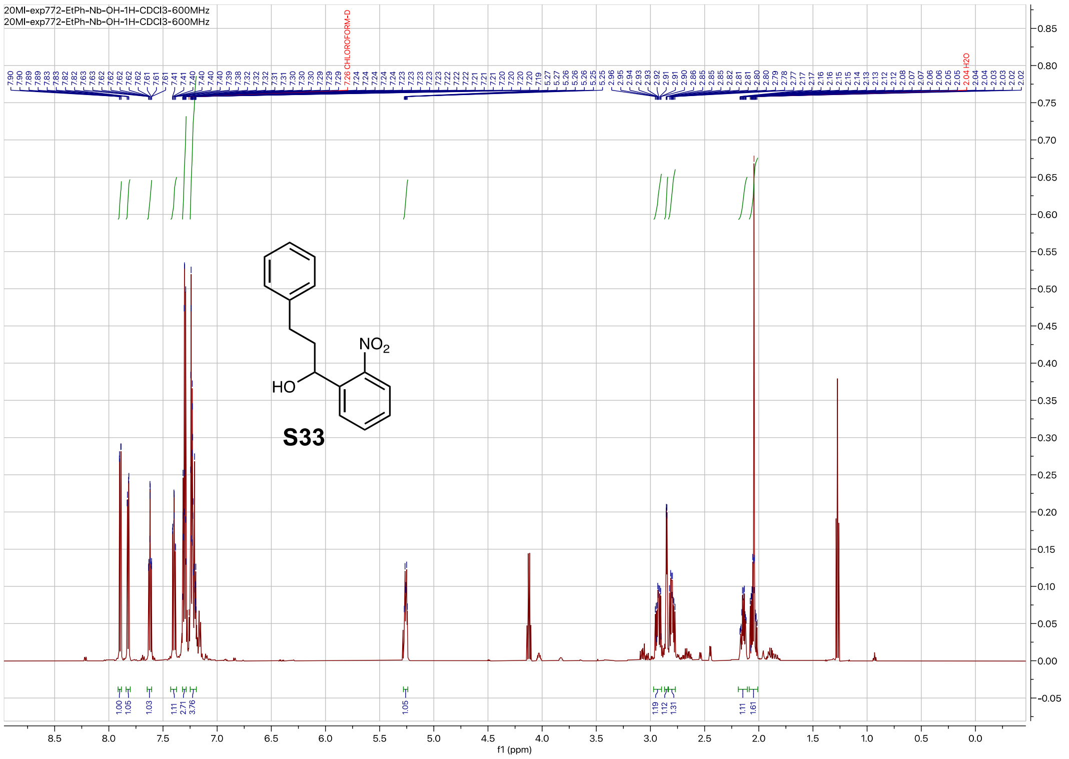

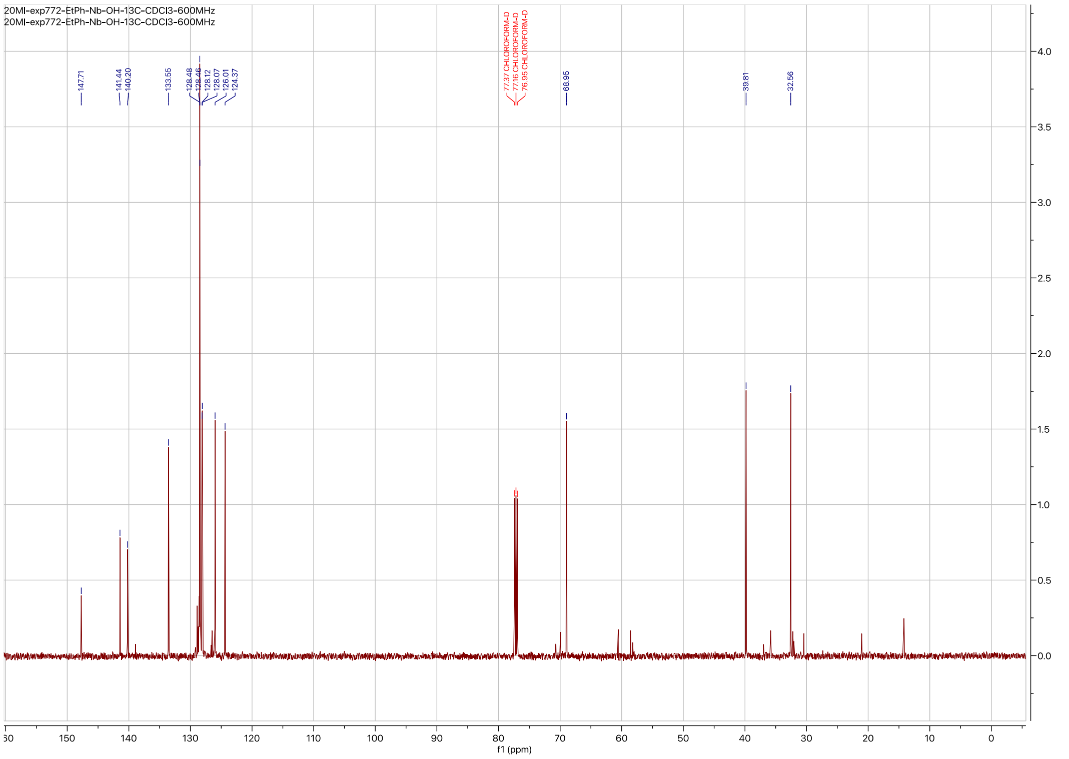

^{1}H and $^{13}C\{^{1}H\}$ NMR Spectra of Compound **S34**

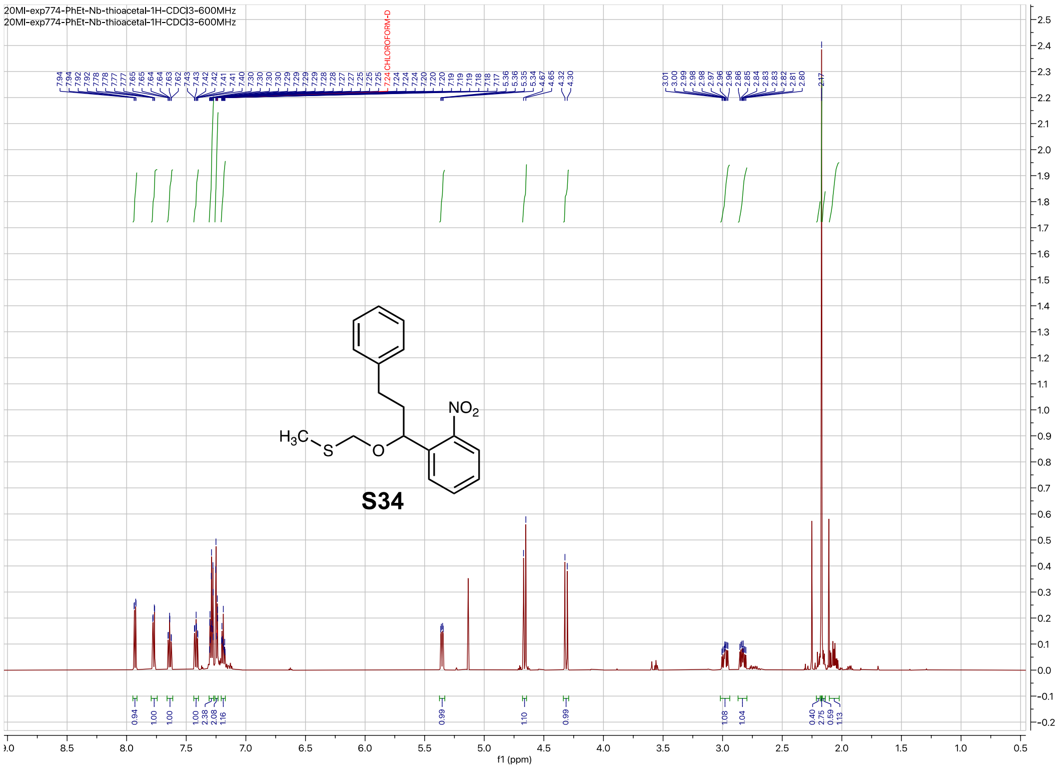

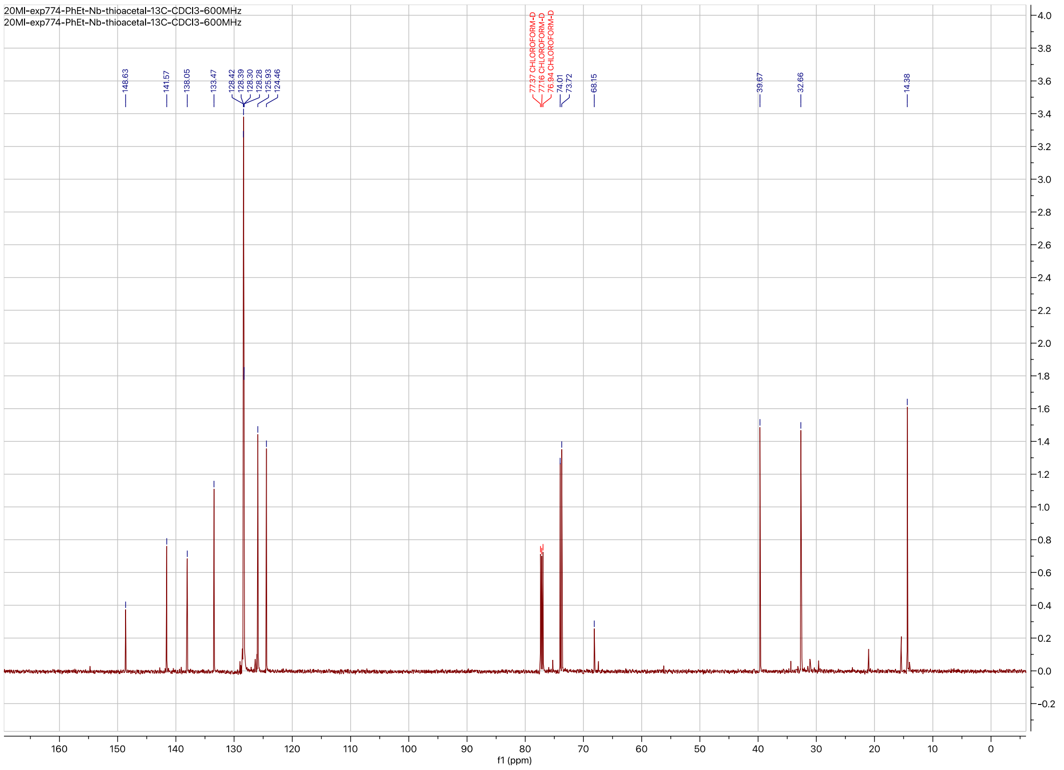

^{1}H and $^{13}C\{^{1}H\}$ NMR Spectra of Compound **S35**

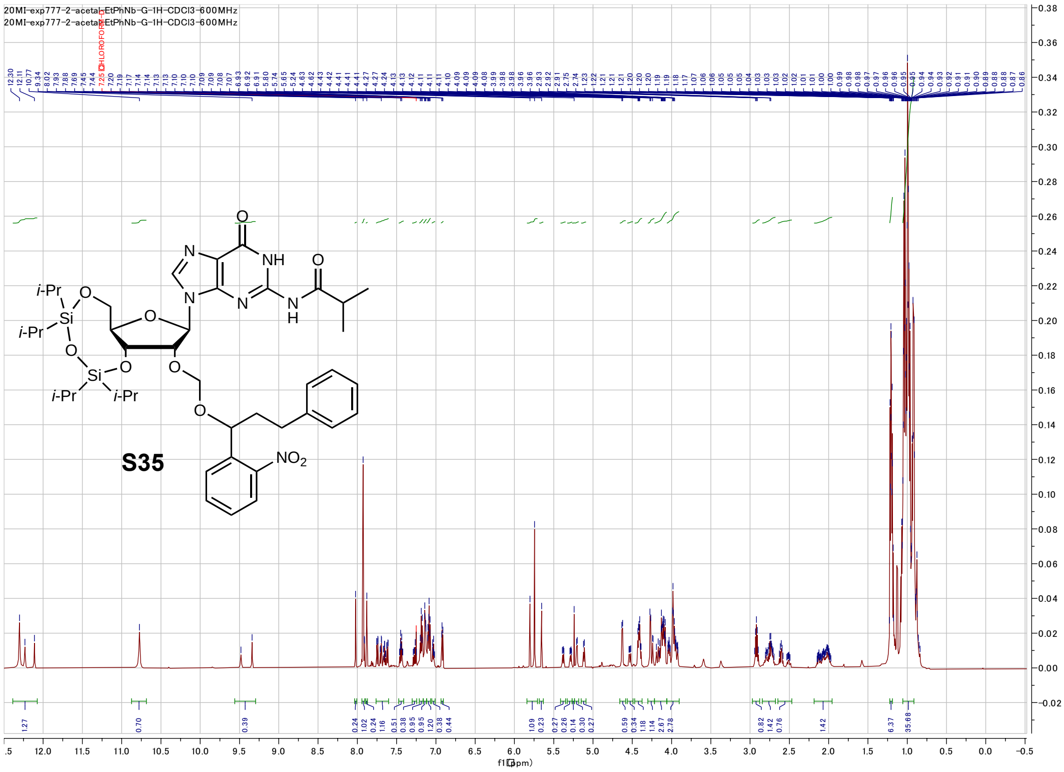

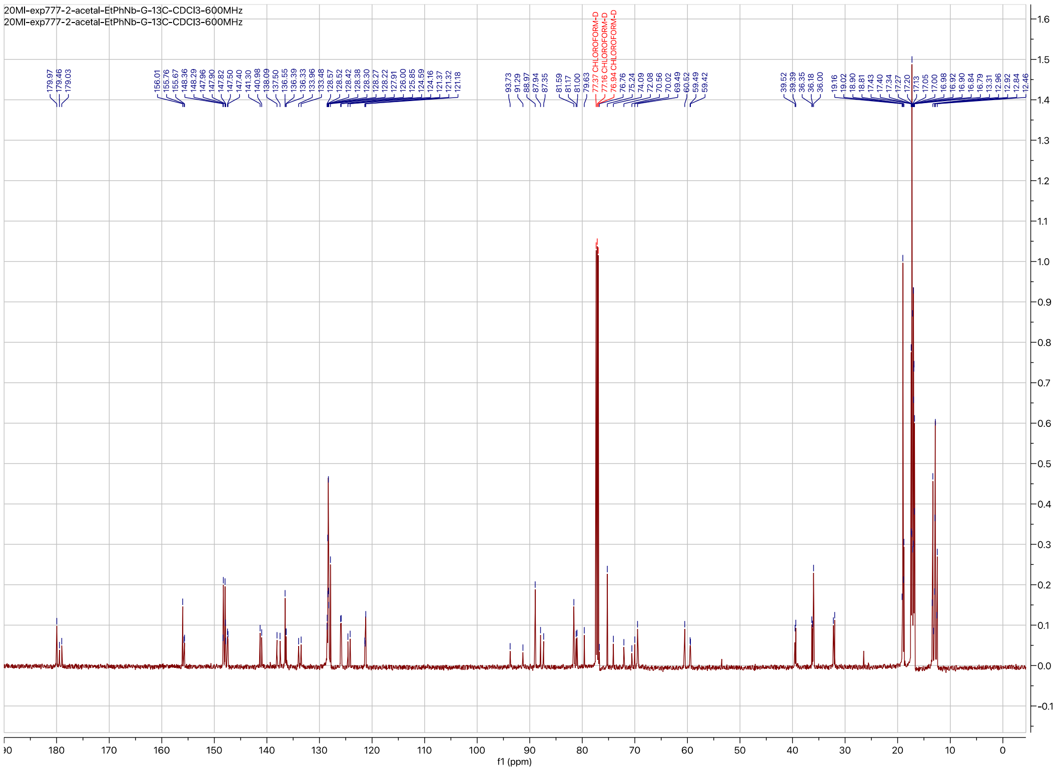

^{1}H and $^{13}C\{^{1}H\}$ NMR Spectra of Compound **S36**

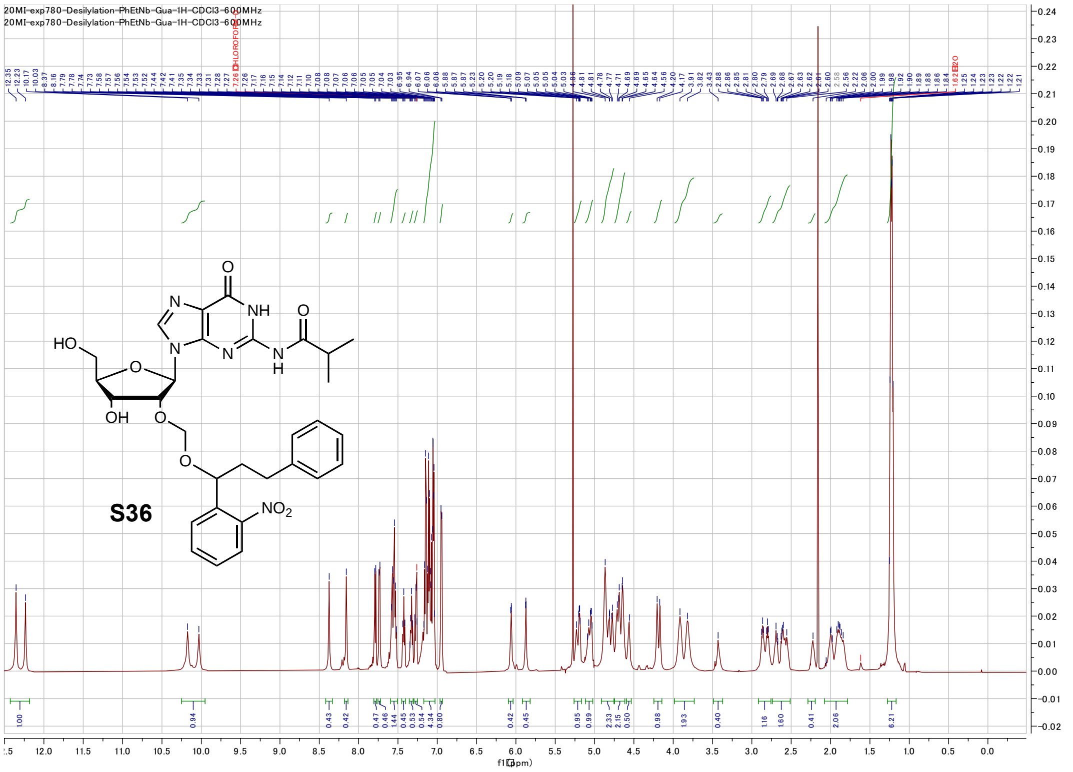

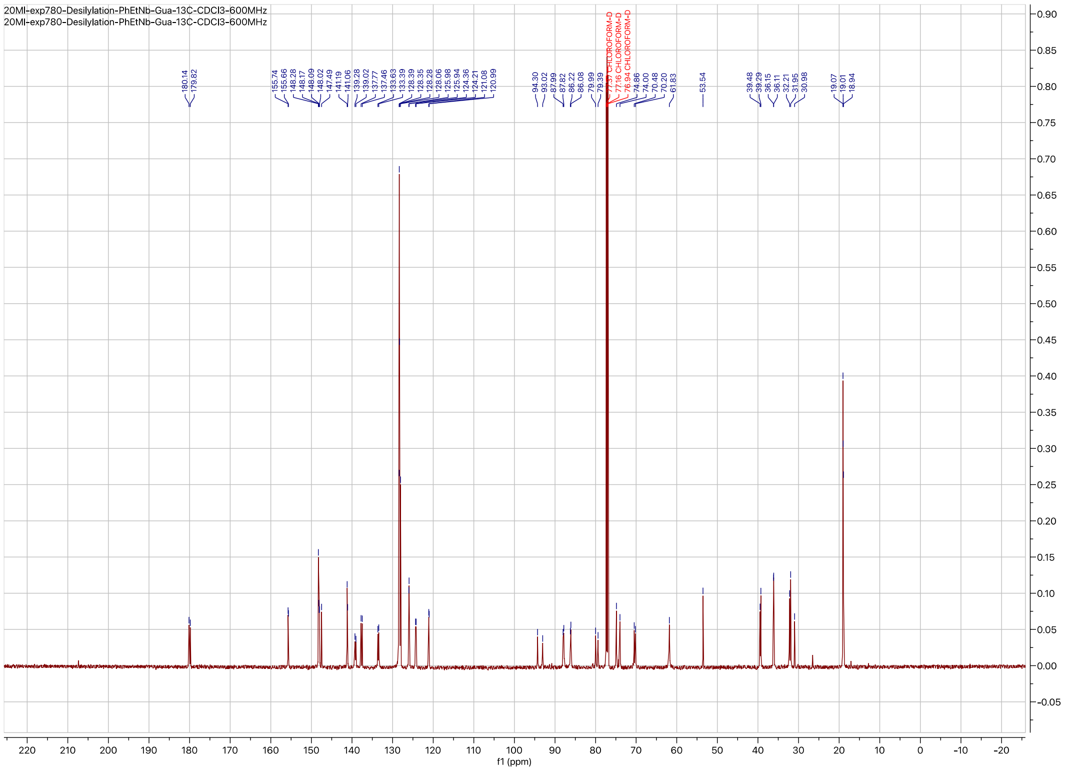

^{1}H and ^{13}C{^{1}H} NMR Spectra of Compound **S37**

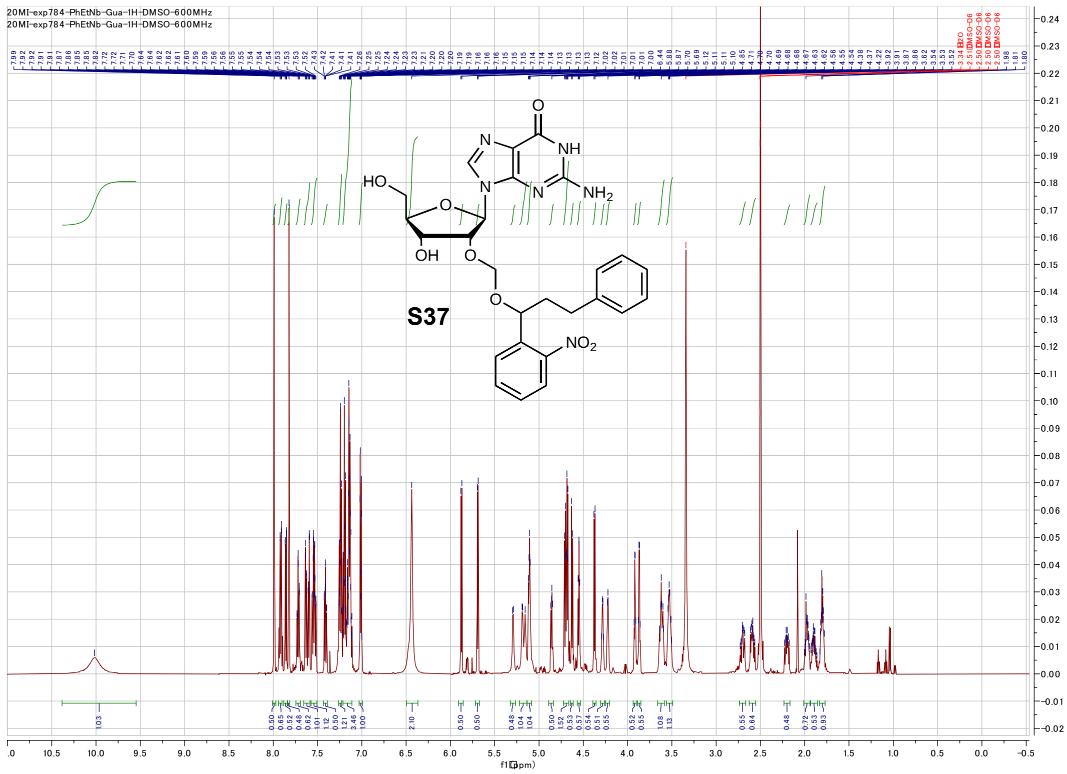

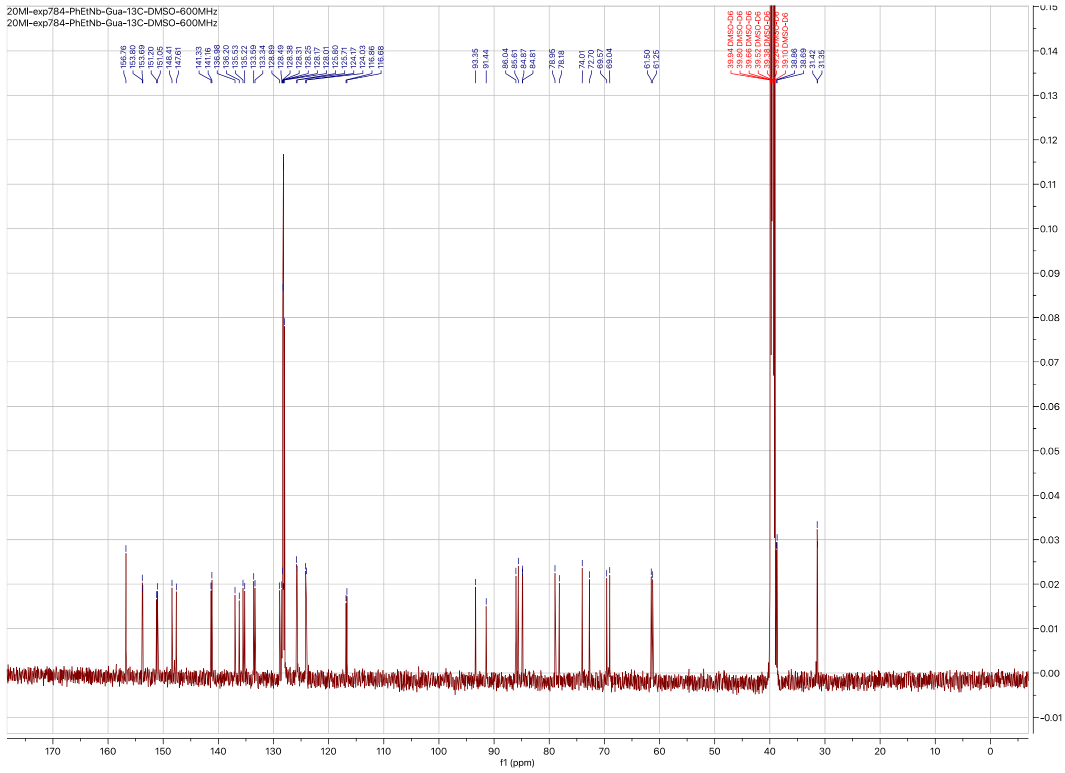

^{1}H and $^{13}C\{^{1}H\}$ NMR Spectra of Compound **S38**

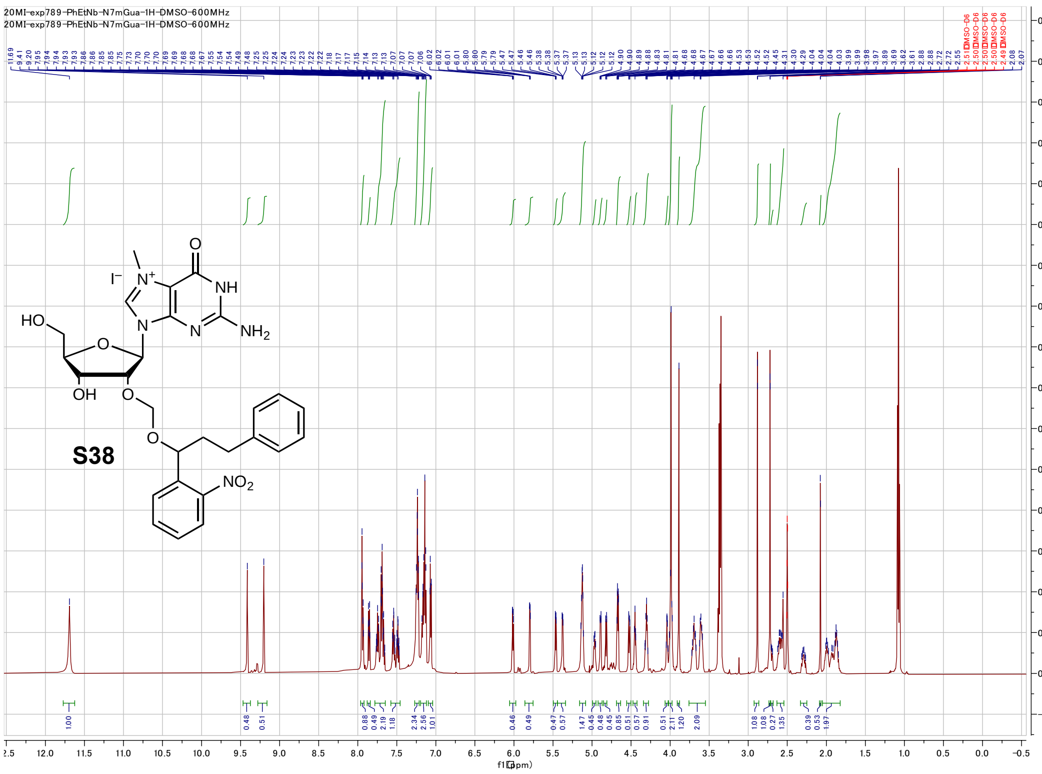

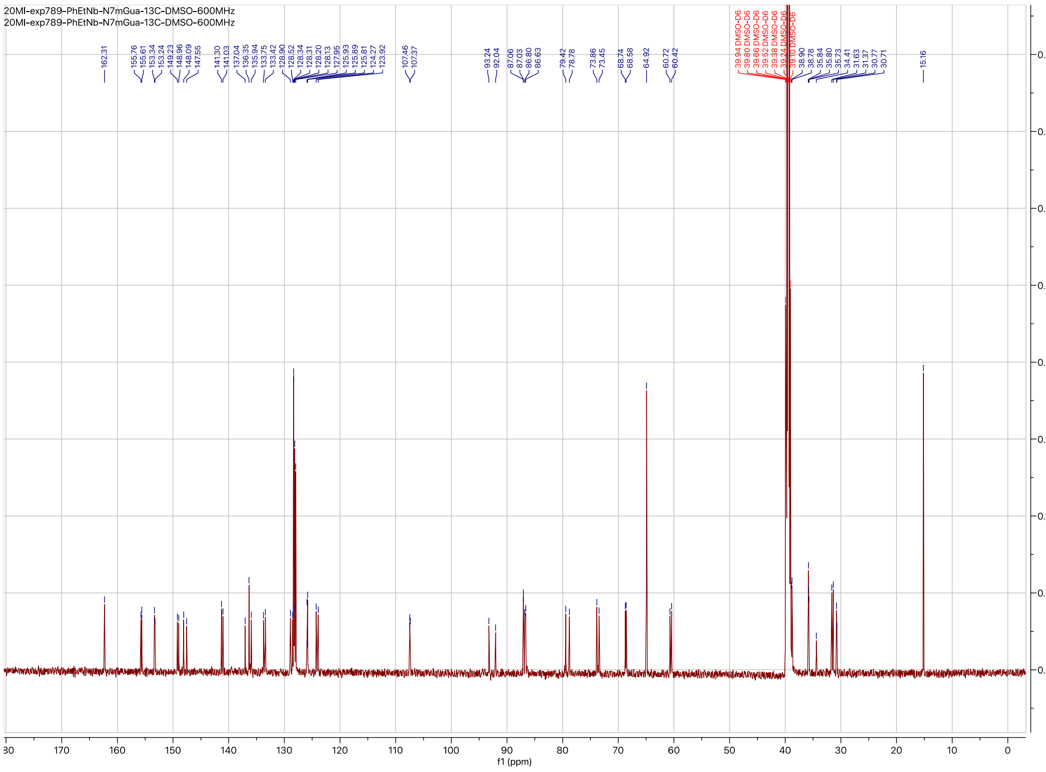

^{1}H and $^{13}C\{^{1}H\}$ NMR Spectra of Compound **S39**

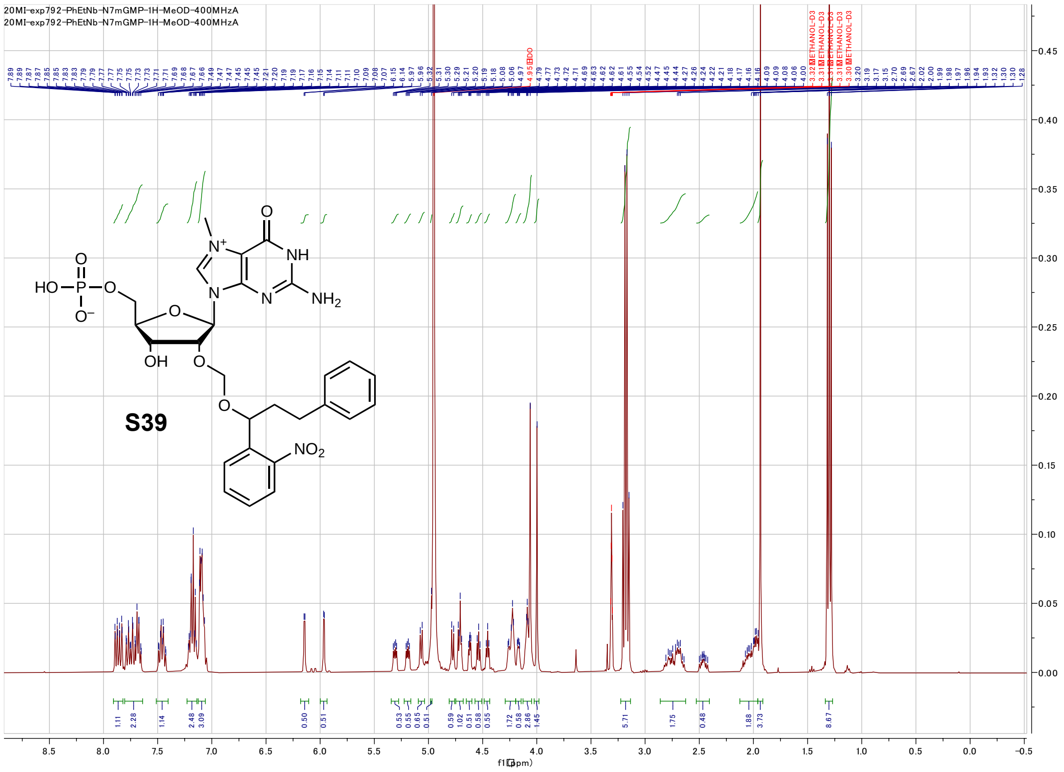

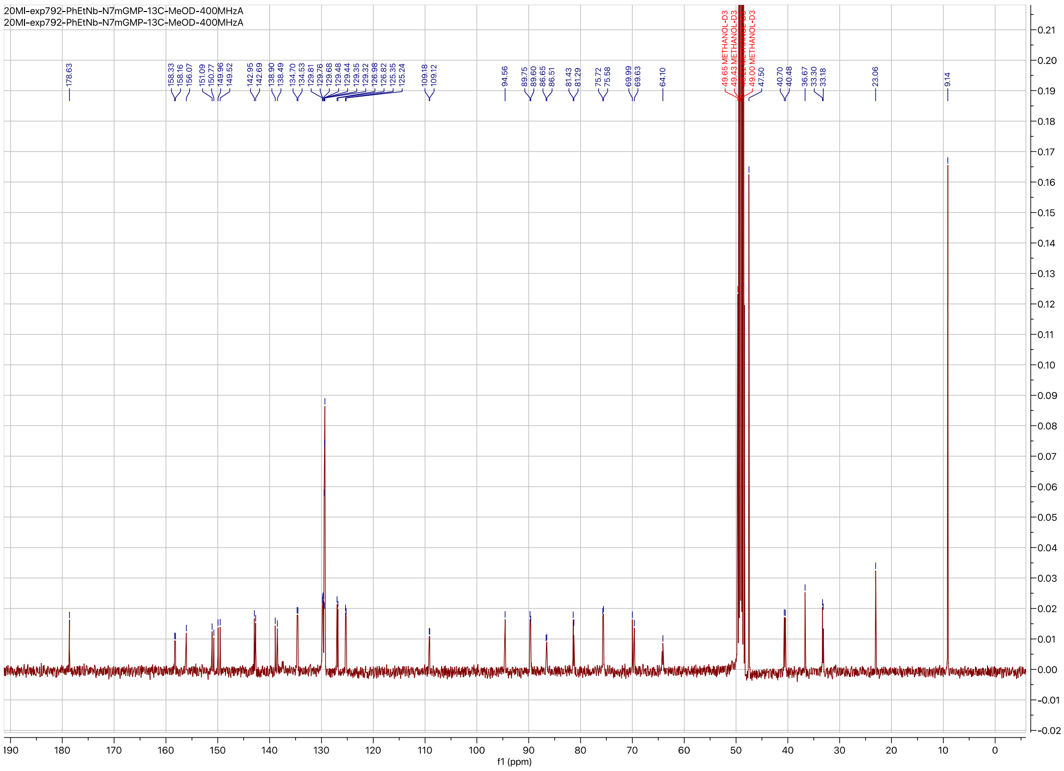

$^{31}P\{^1H\}$ NMR Spectrum of Compound **S39**

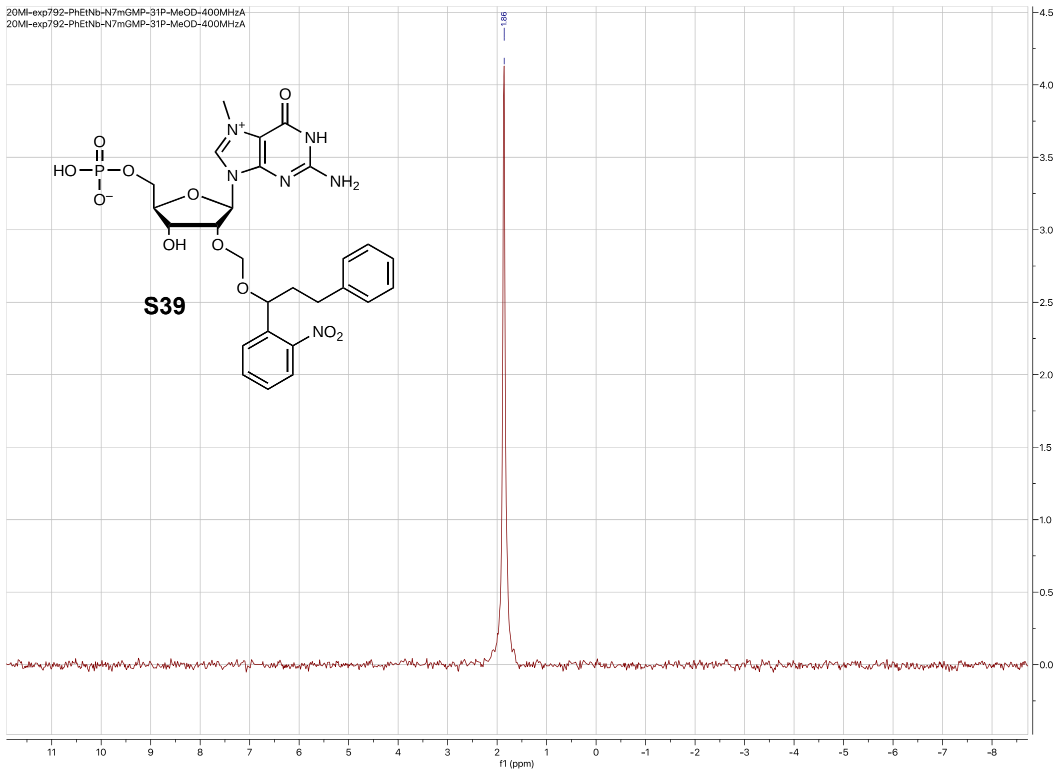

^{1}H and ^{13}C{^{1}H} NMR Spectra of Compound **23**

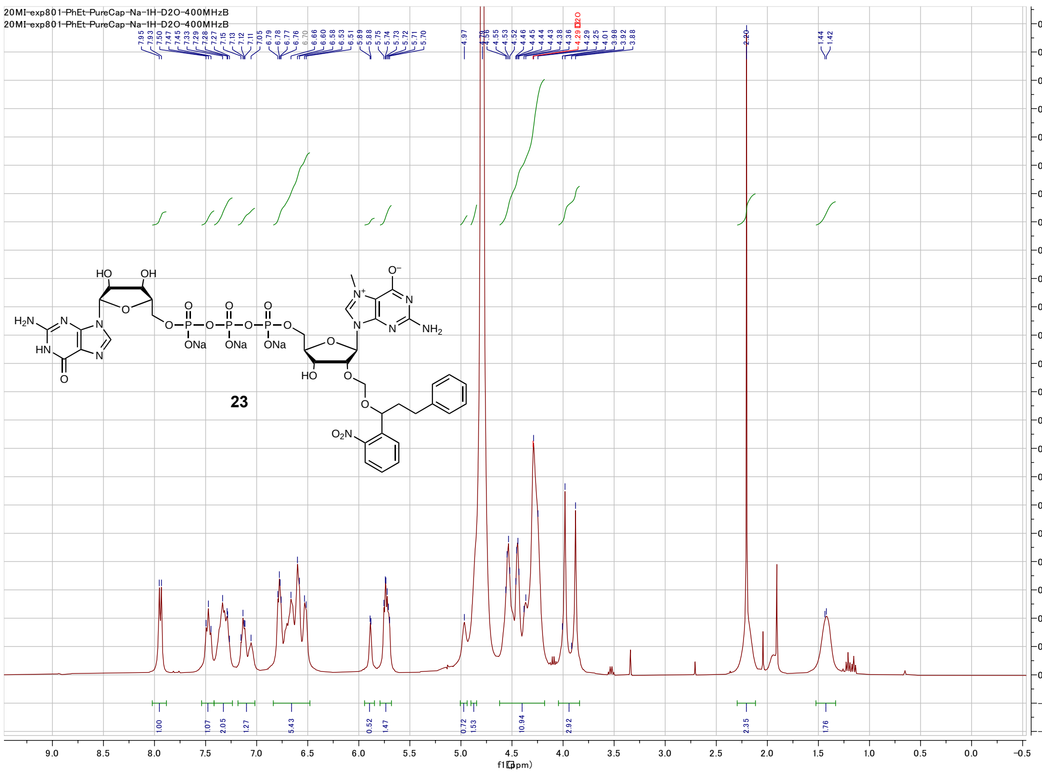

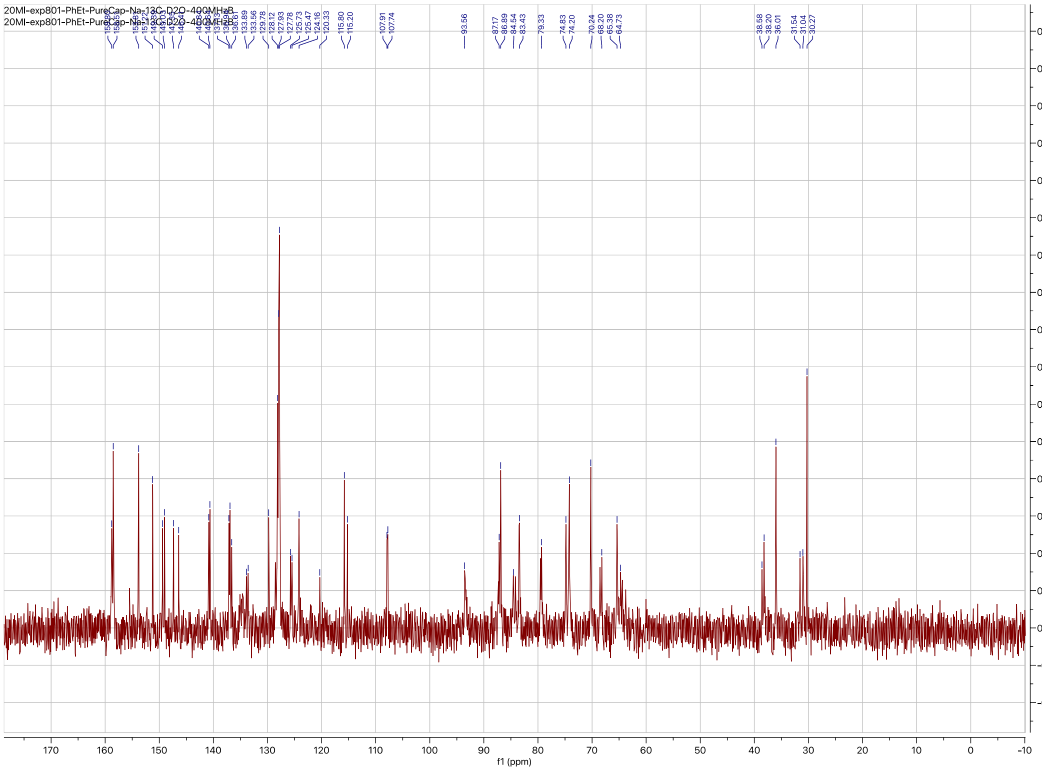

$^{31}P\{^{1}H\}$ NMR Spectrum of Compound **23**

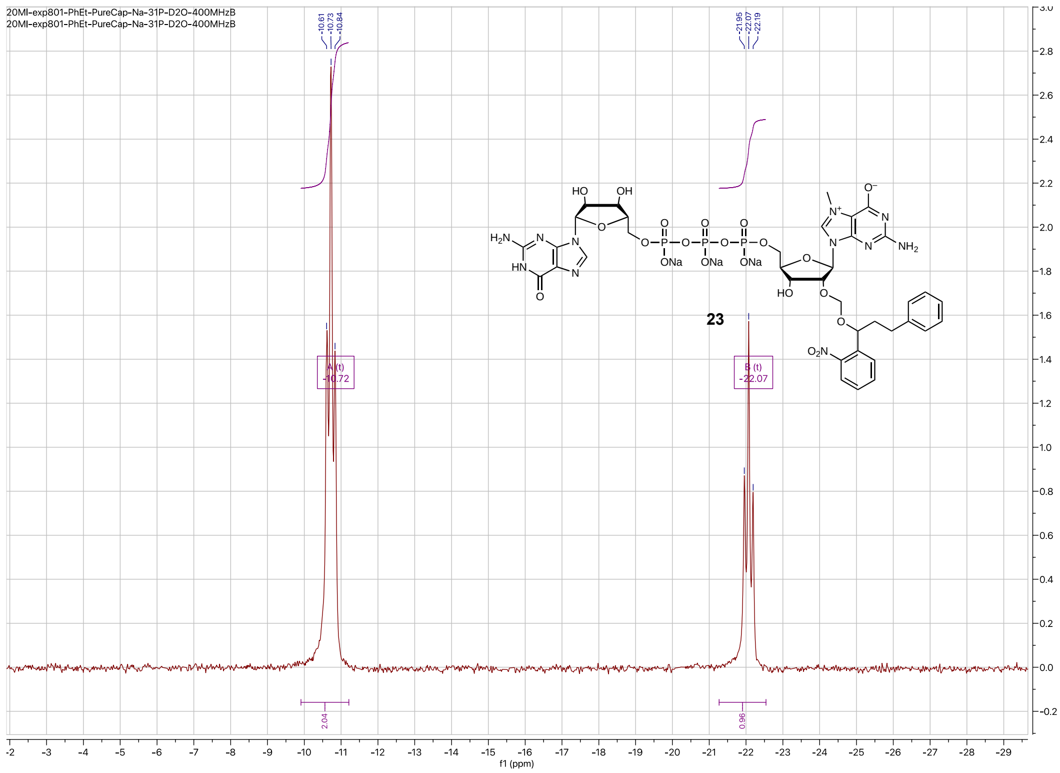

^1H and $^{13}C\{^1H\}$ NMR Spectra of Compound **S40**

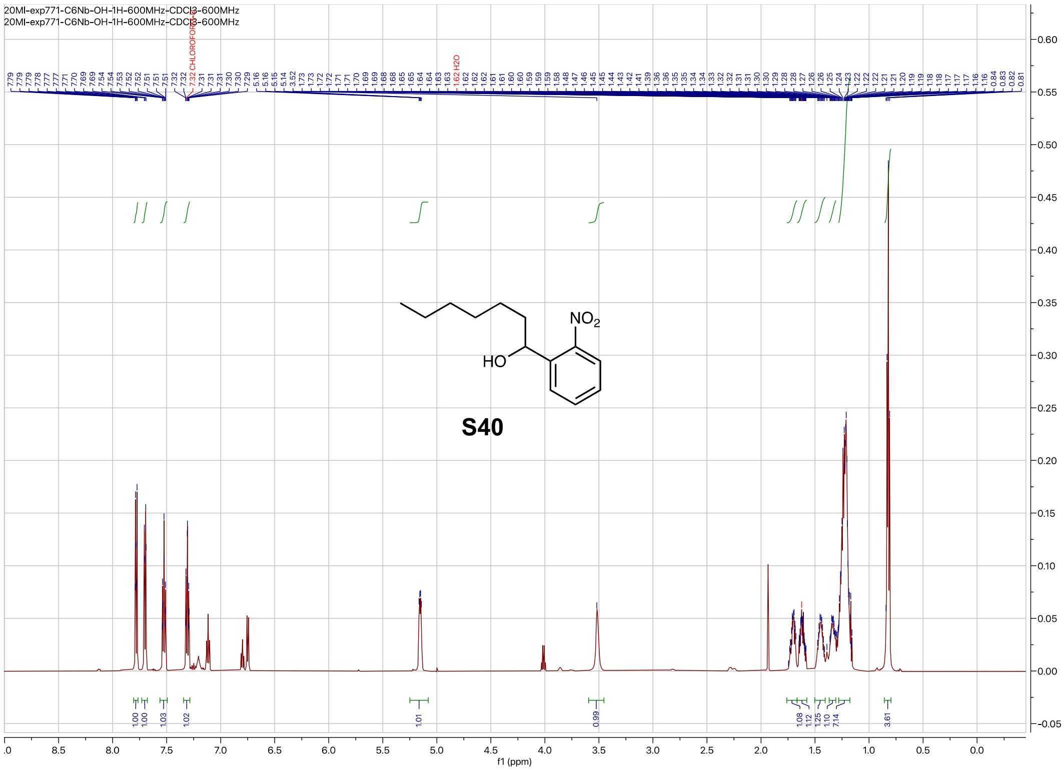

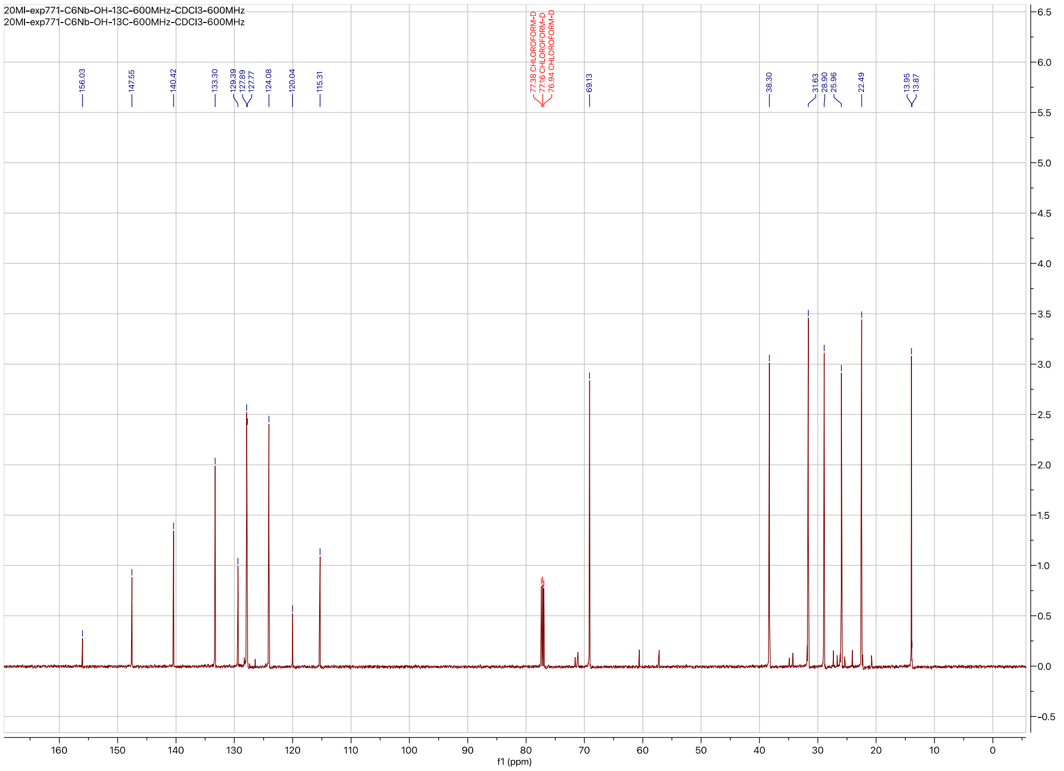

^{1}H and $^{13}C\{^{1}H\}$ NMR Spectra of Compound **S41**

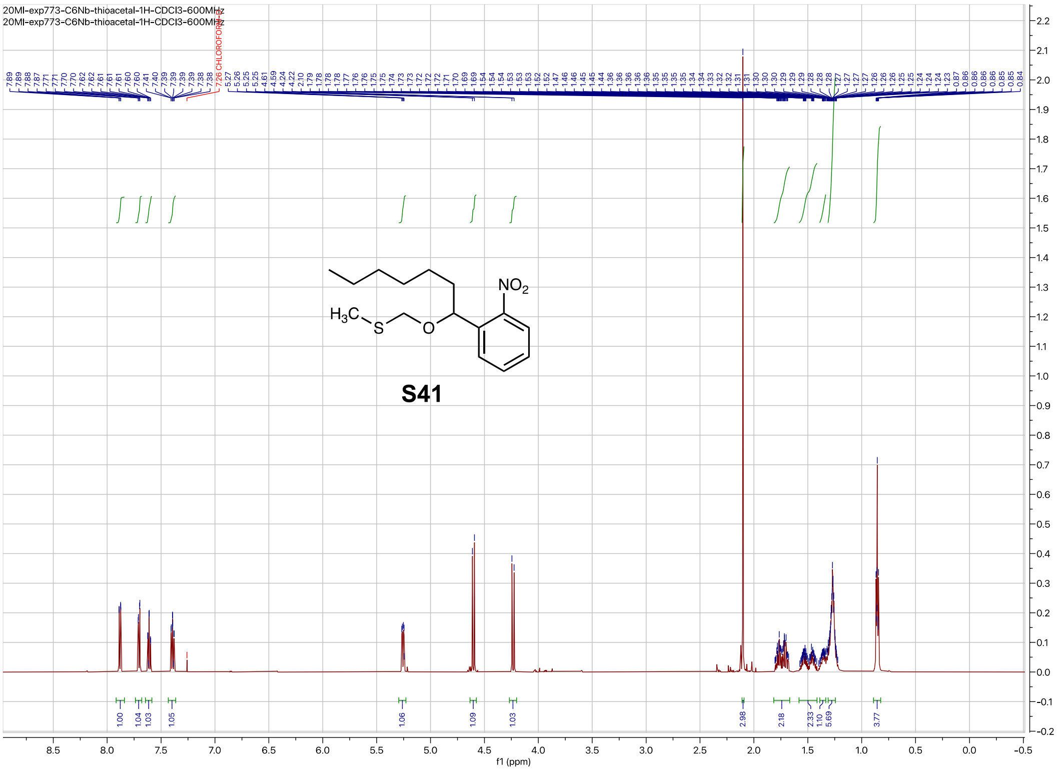

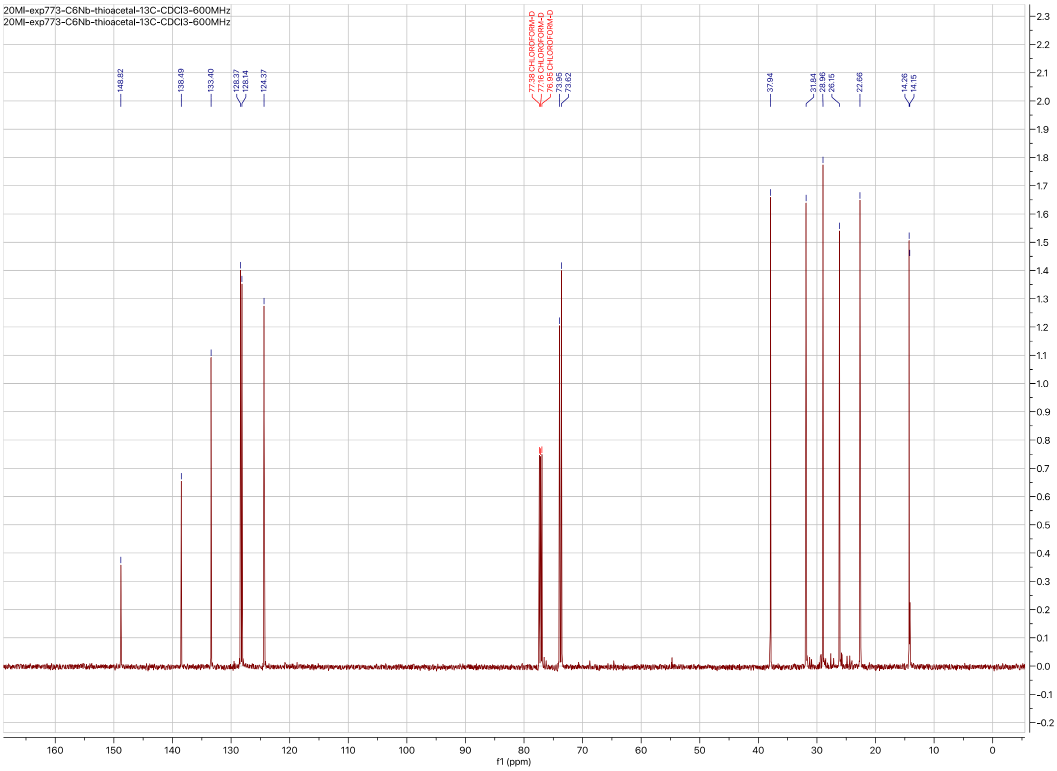

^{1}H and $^{13}C\{^{1}H\}$ NMR Spectra of Compound **S42**

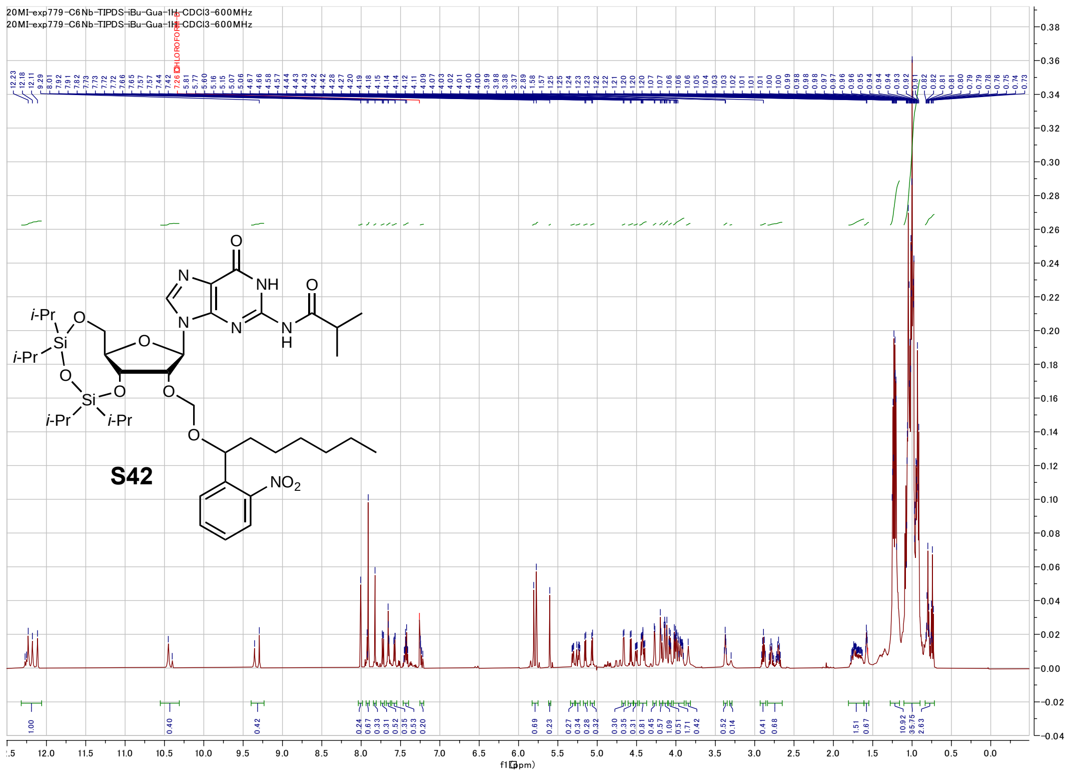

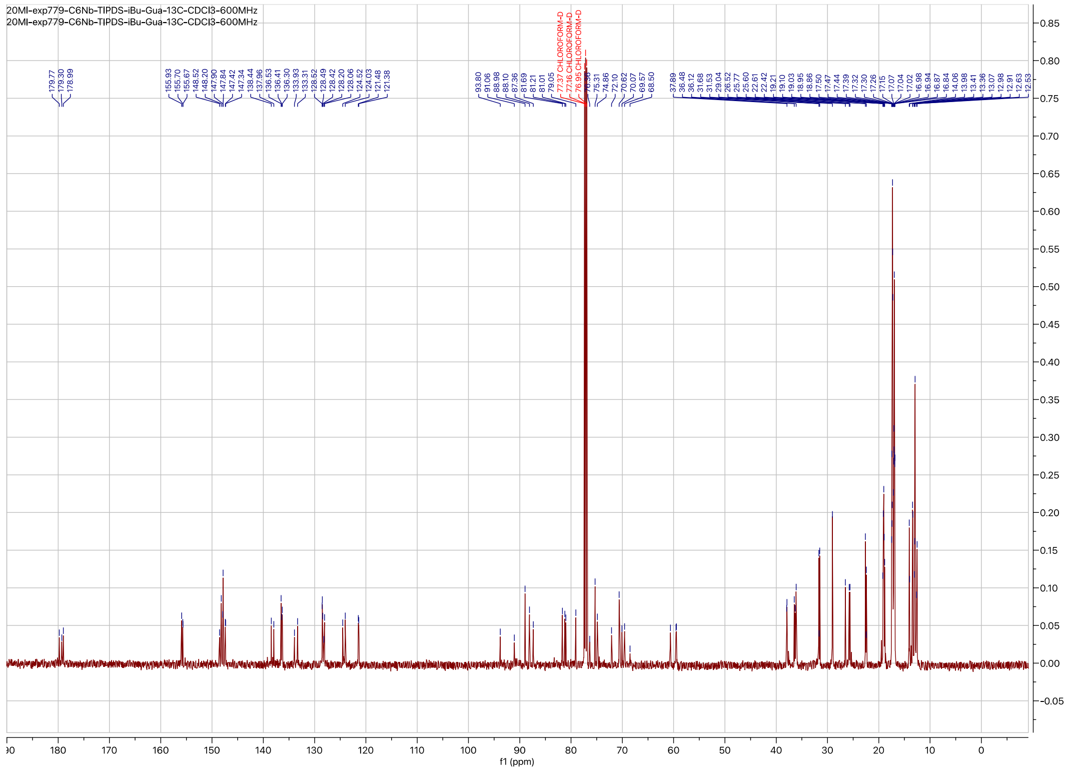

^{1}H and $^{13}C\{^{1}H\}$ NMR Spectra of Compound **S43**

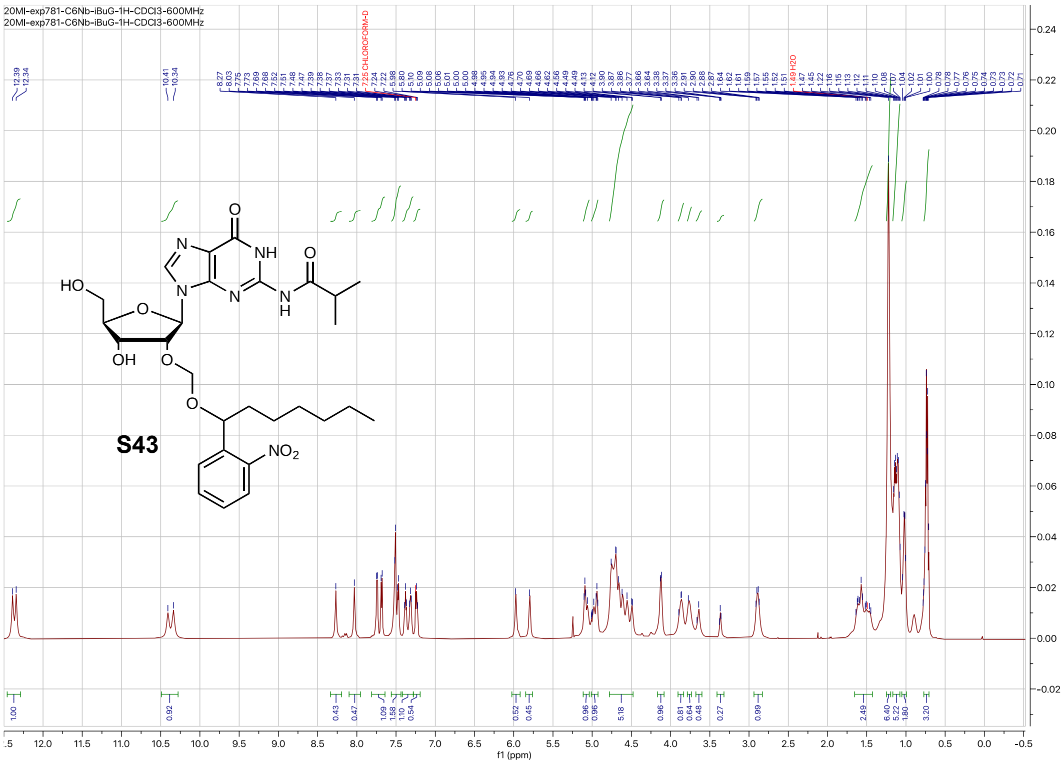

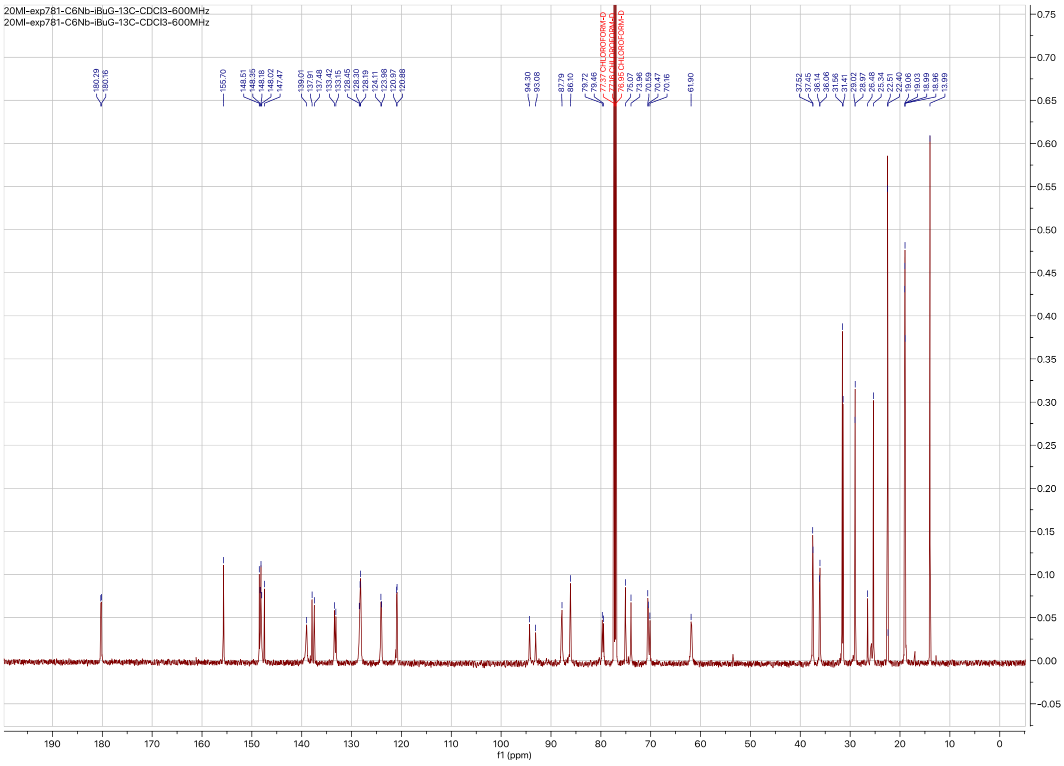

^{1}H and ^{13}C{^{1}H} NMR Spectra of Compound **S44**

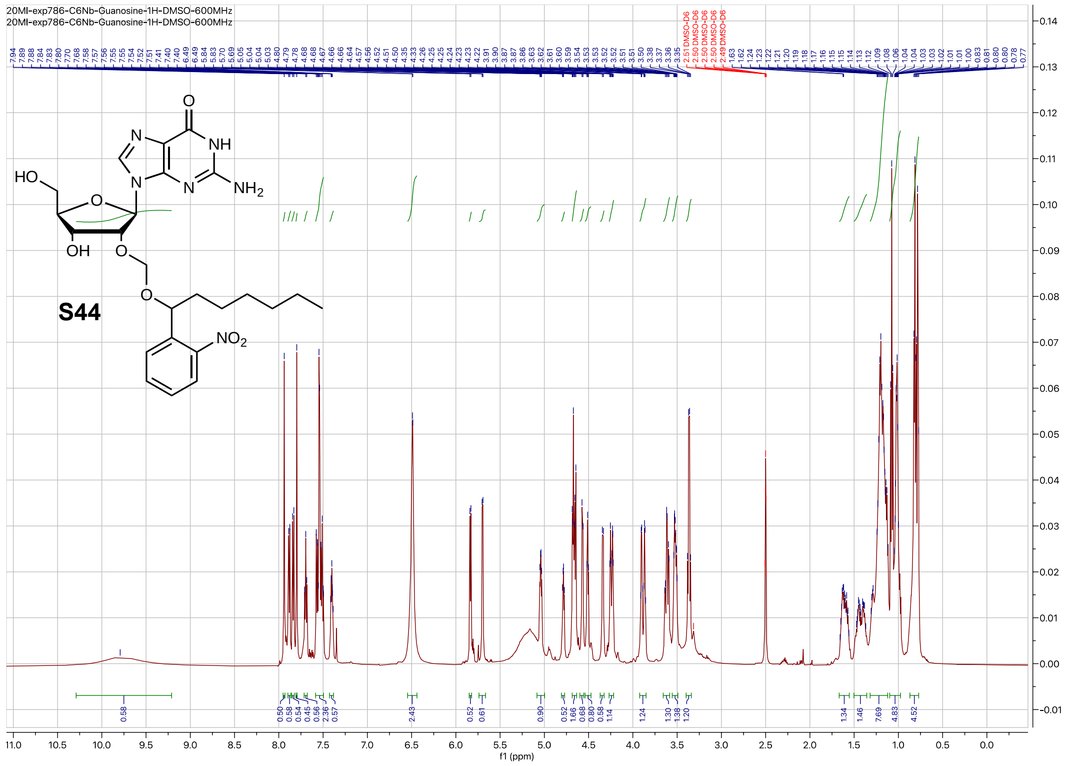

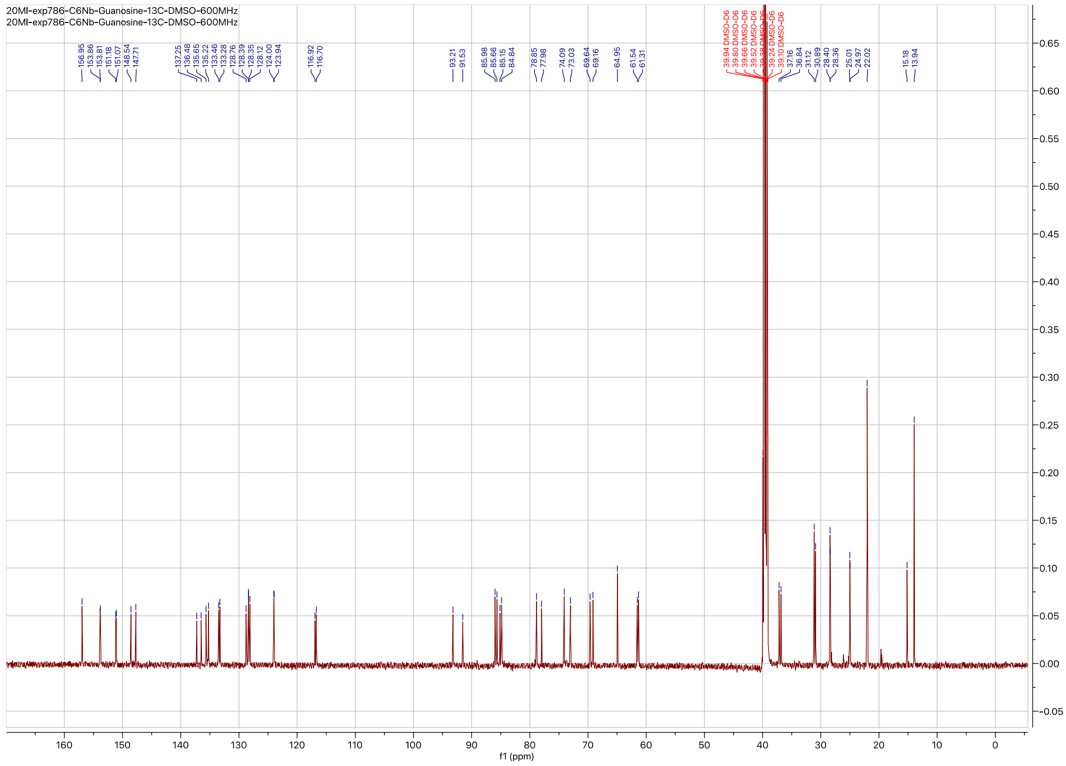

^{1}H and ^{13}C{^{1}H} NMR Spectra of Compound **S45**

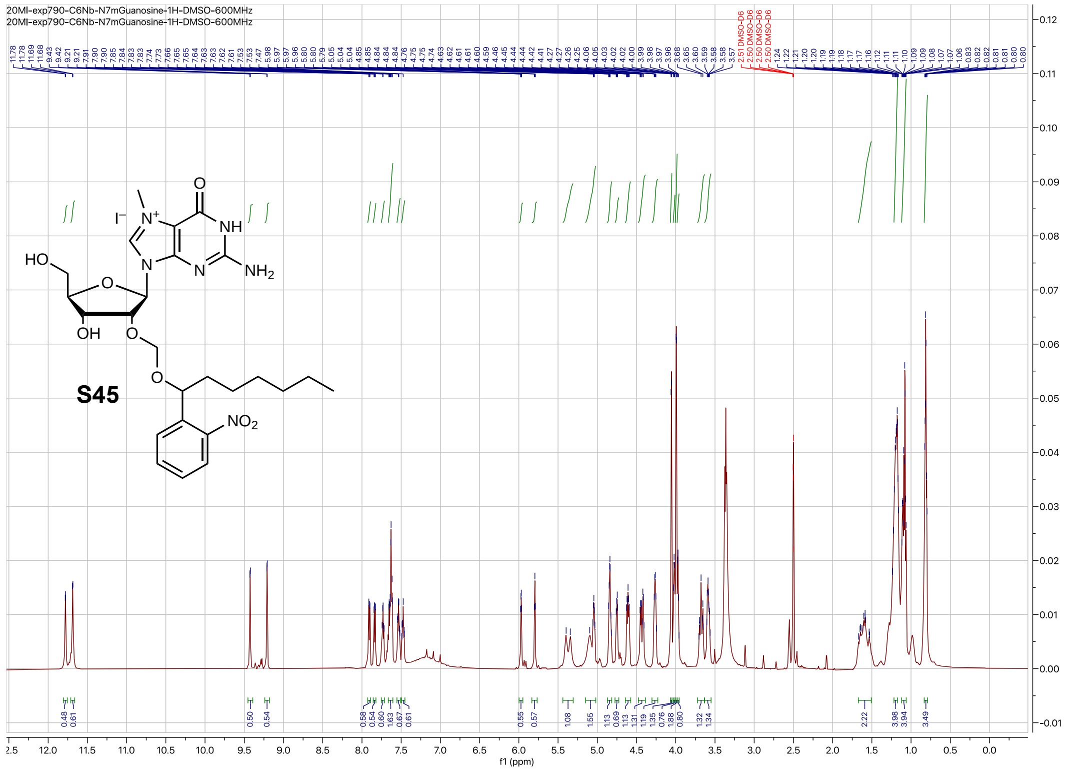

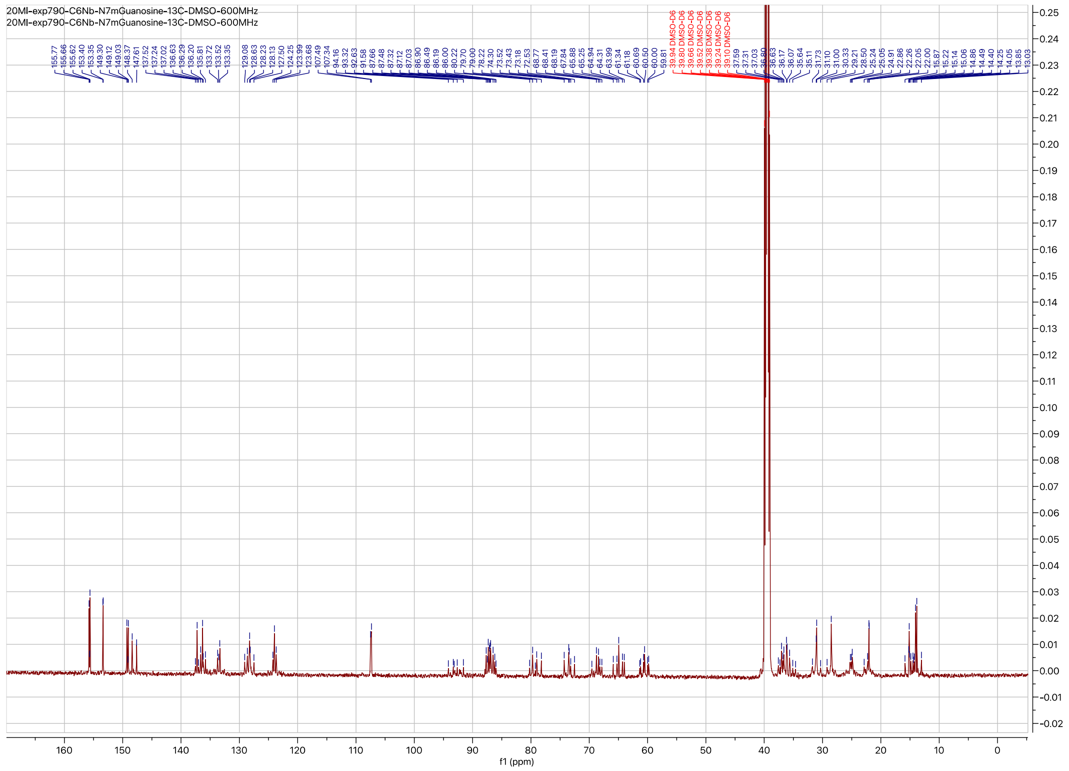

^{1}H and ^{13}C{^{1}H} NMR Spectra of Compound **S46**

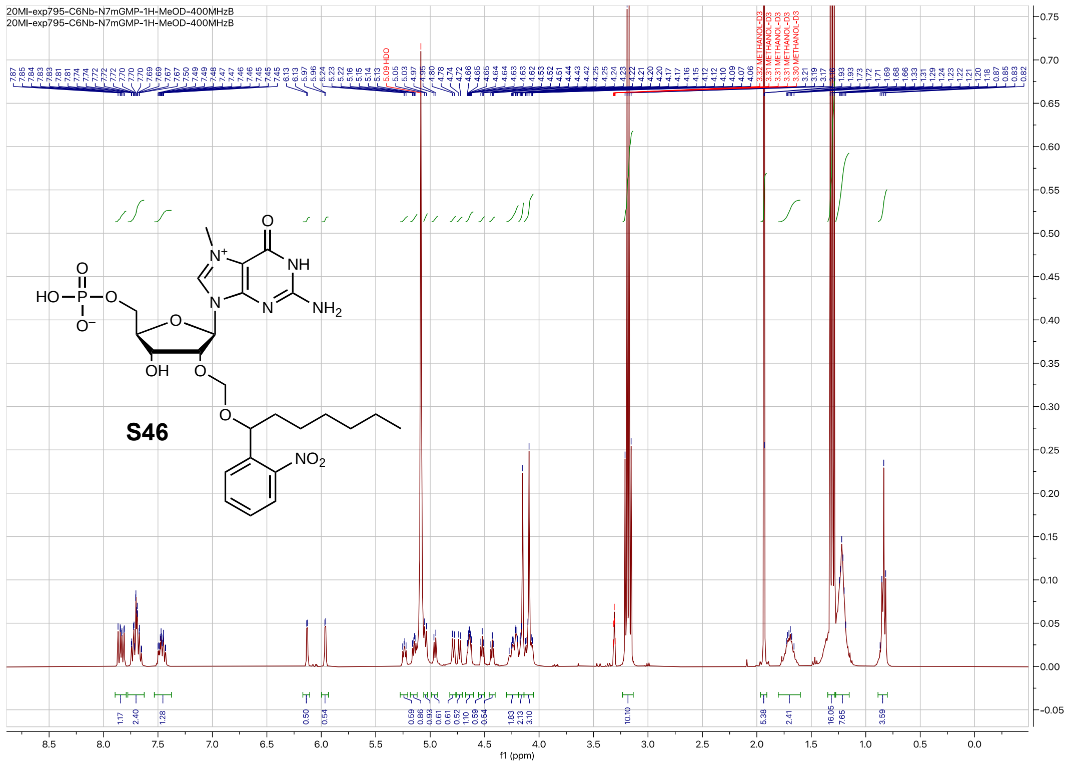

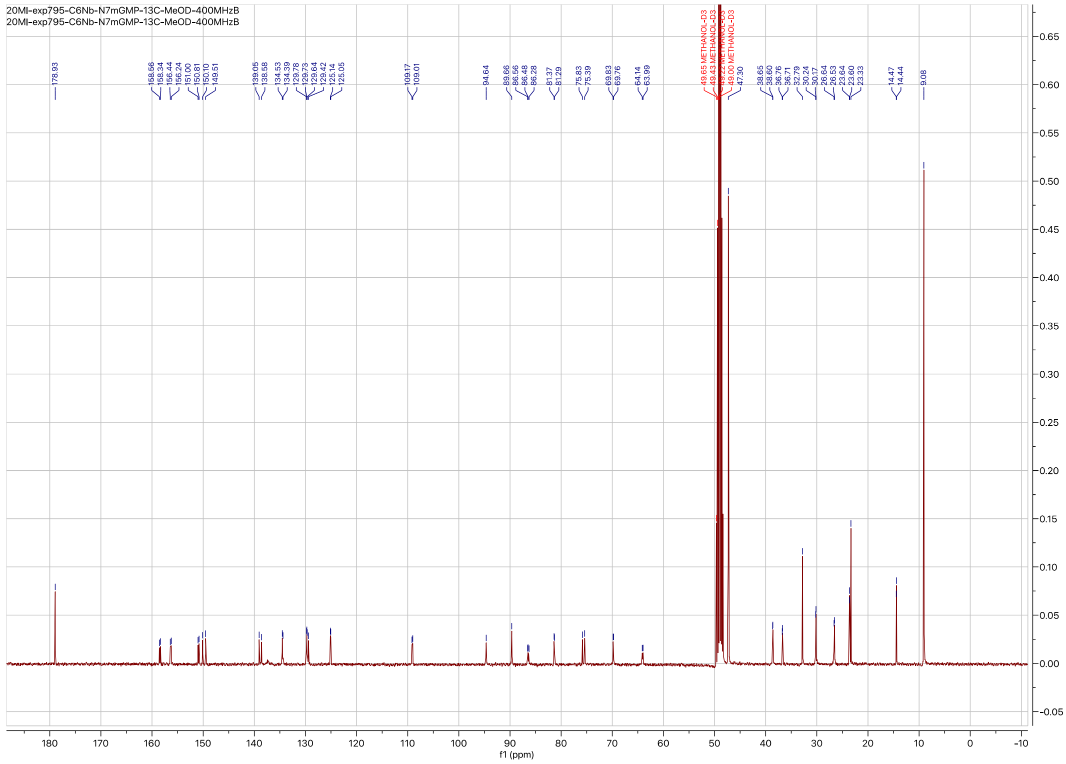

$^{31}P\{^1H\}$ NMR Spectrum of Compound **S46**

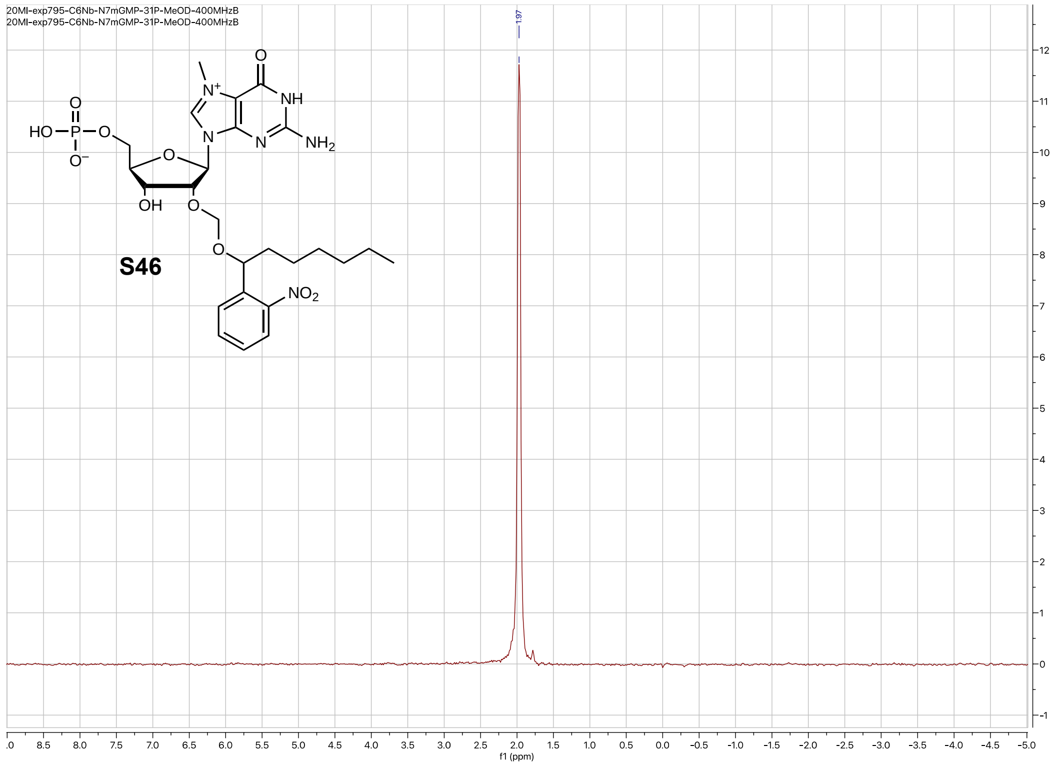

^1H and $^{13}C\{^1H\}$ NMR Spectra of Compound **24**

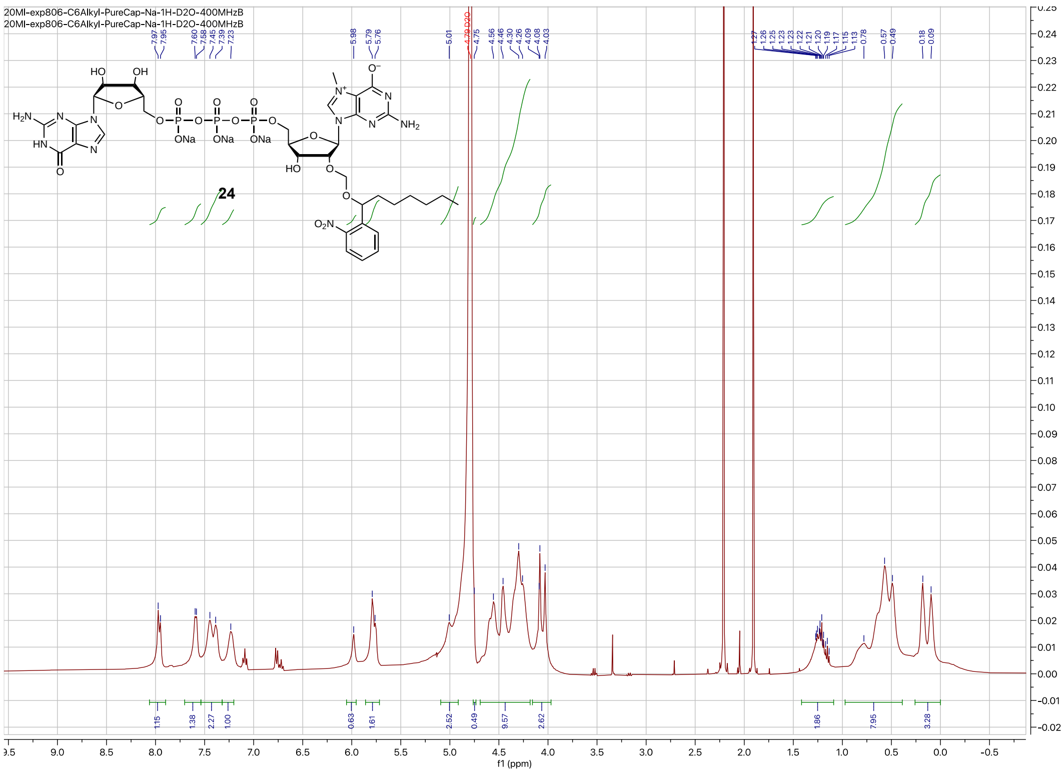

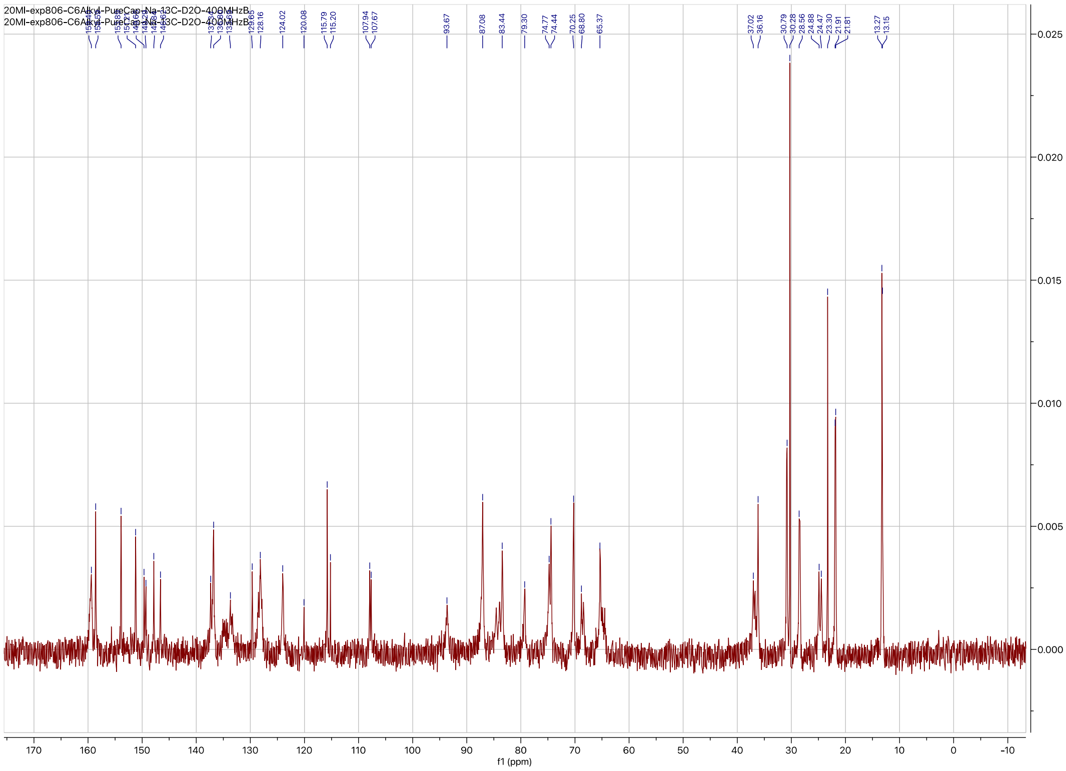

$^{31}P\{^1H\}$ NMR Spectrum of Compound **24**

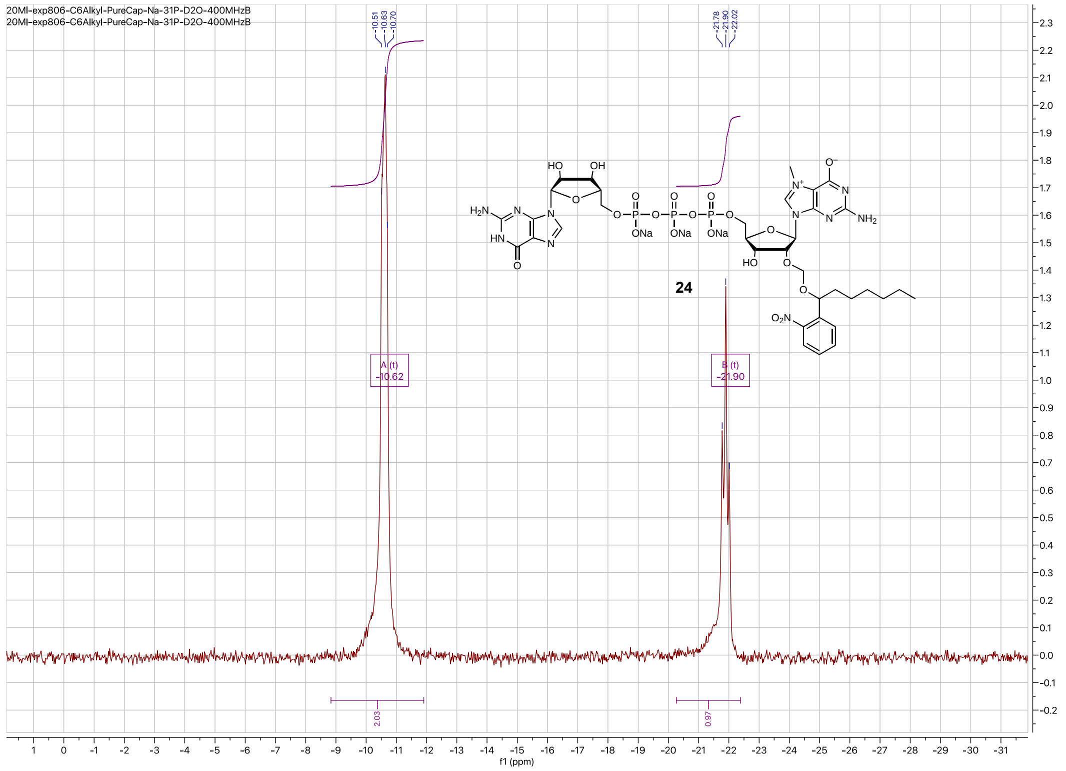

^{1}H and $^{13}C\{^{1}H\}$ NMR Spectra of Compound **S47**

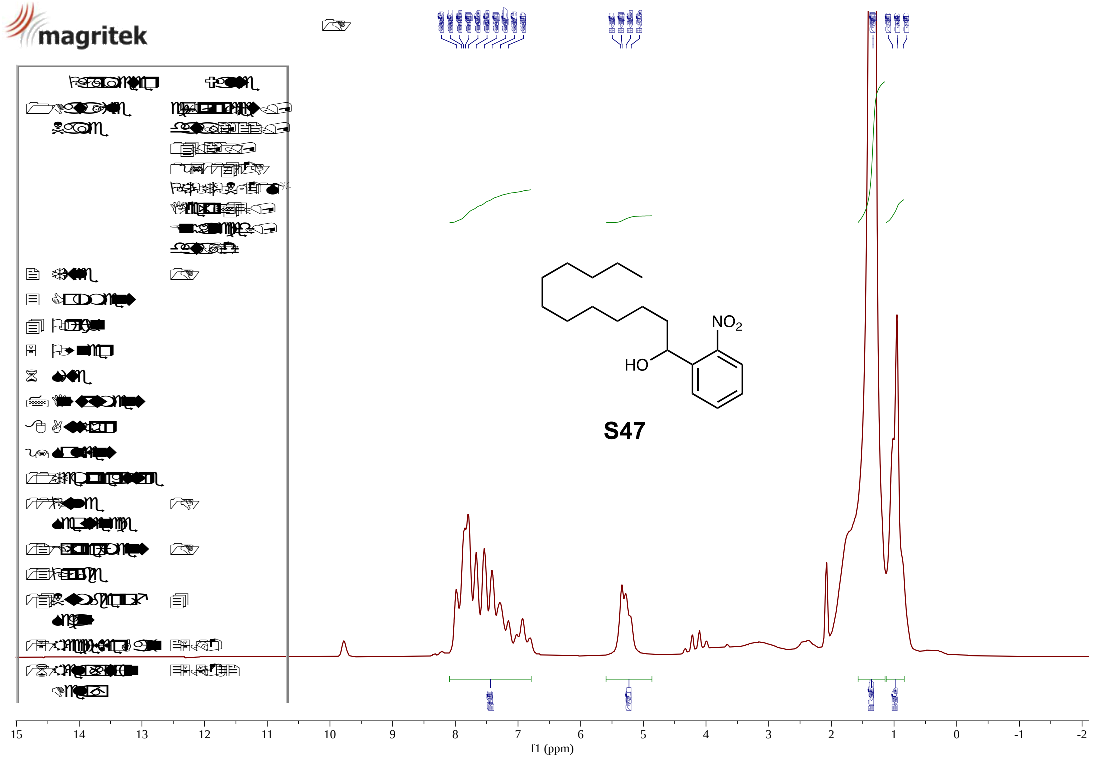

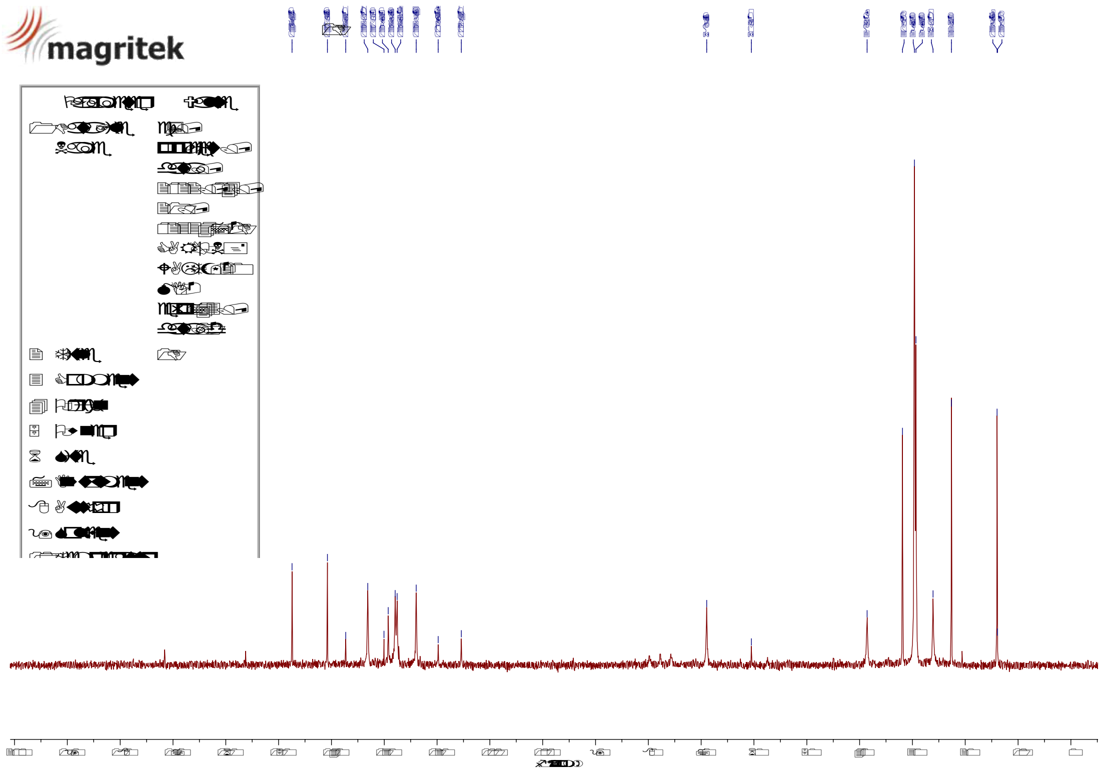

^{1}H and ^{13}C{^{1}H} NMR Spectra of Compound **S48**

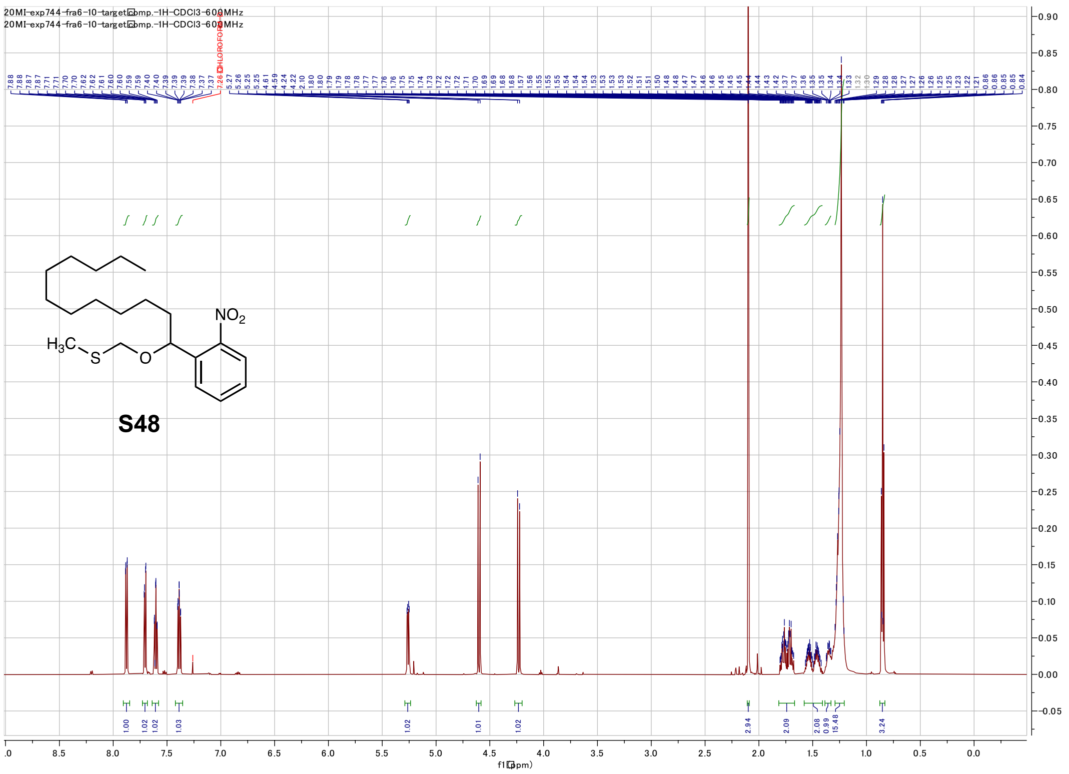

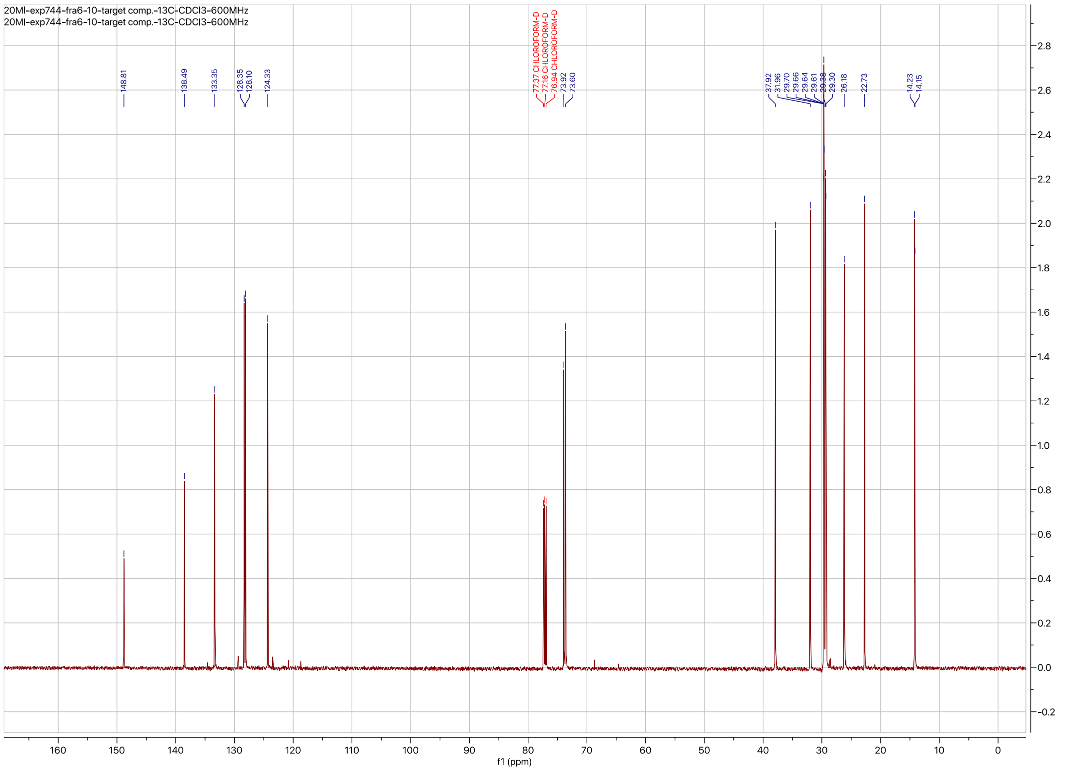

^{1}H and $^{13}C\{^{1}H\}$ NMR Spectra of Compound **S49**

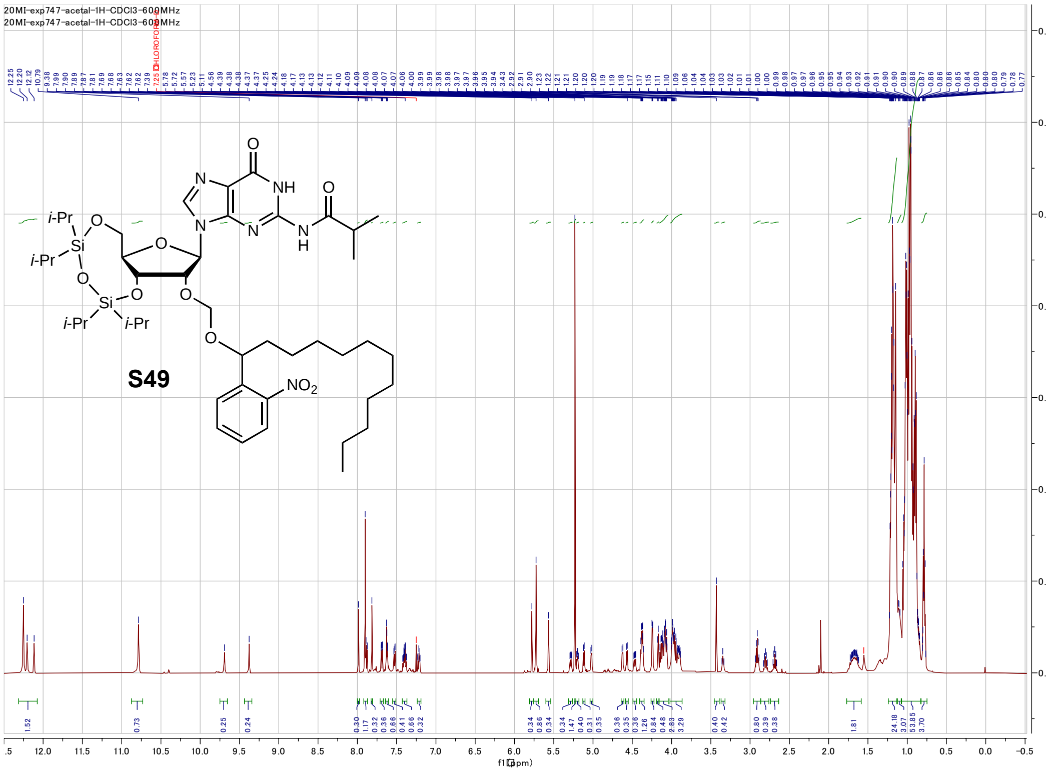

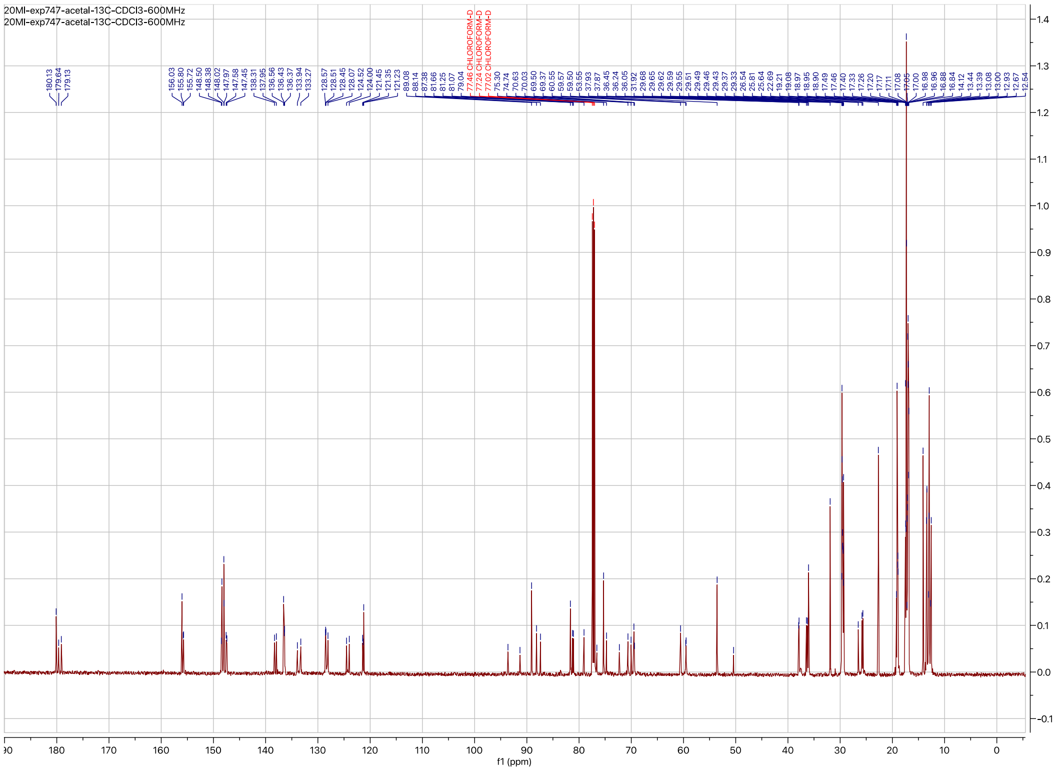

^{1}H and $^{13}C\{^{1}H\}$ NMR Spectra of Compound **S50**

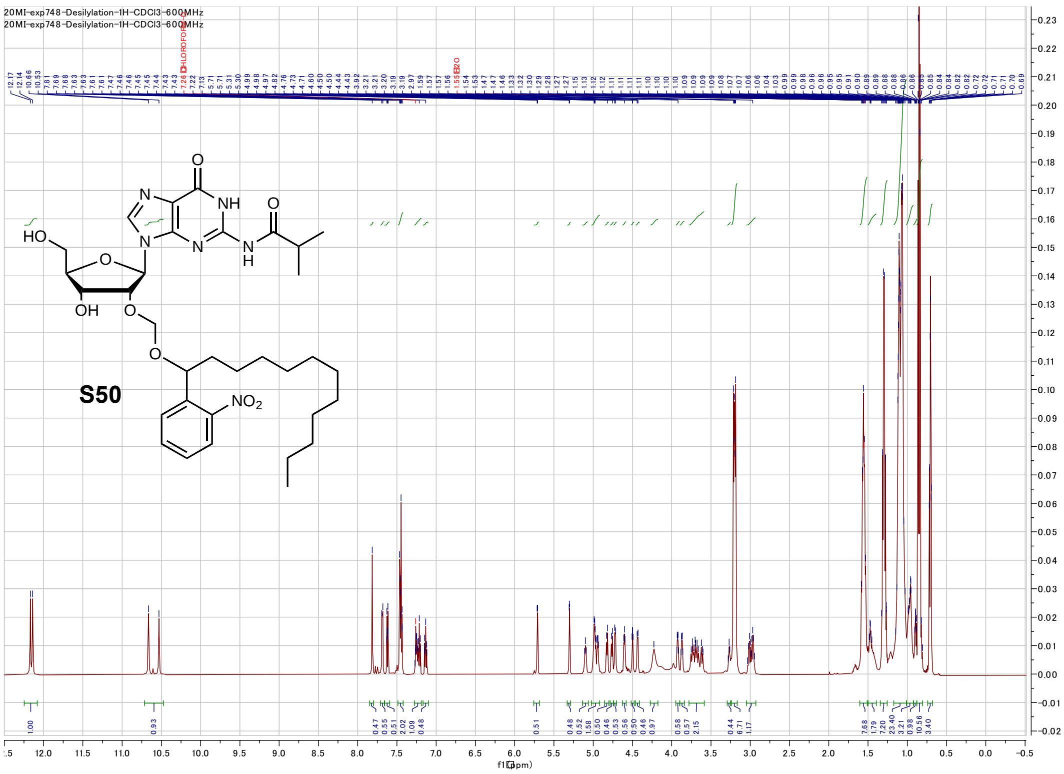

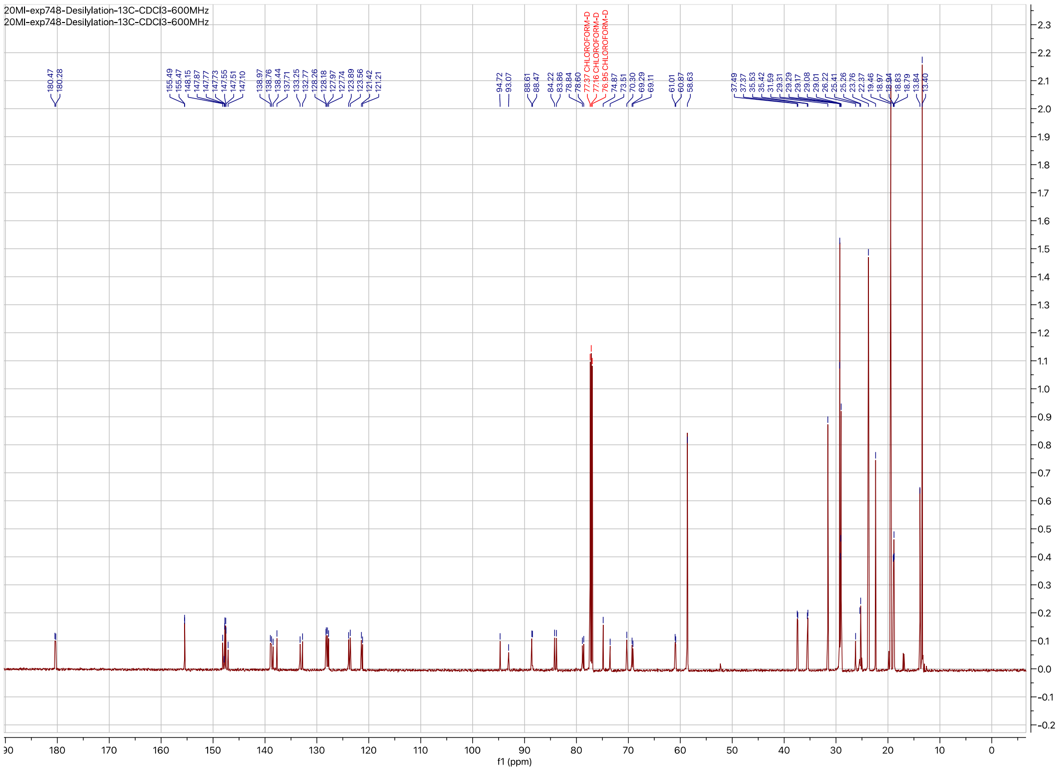

^{1}H and $^{13}C\{^{1}H\}$ NMR Spectra of Compound **S51**

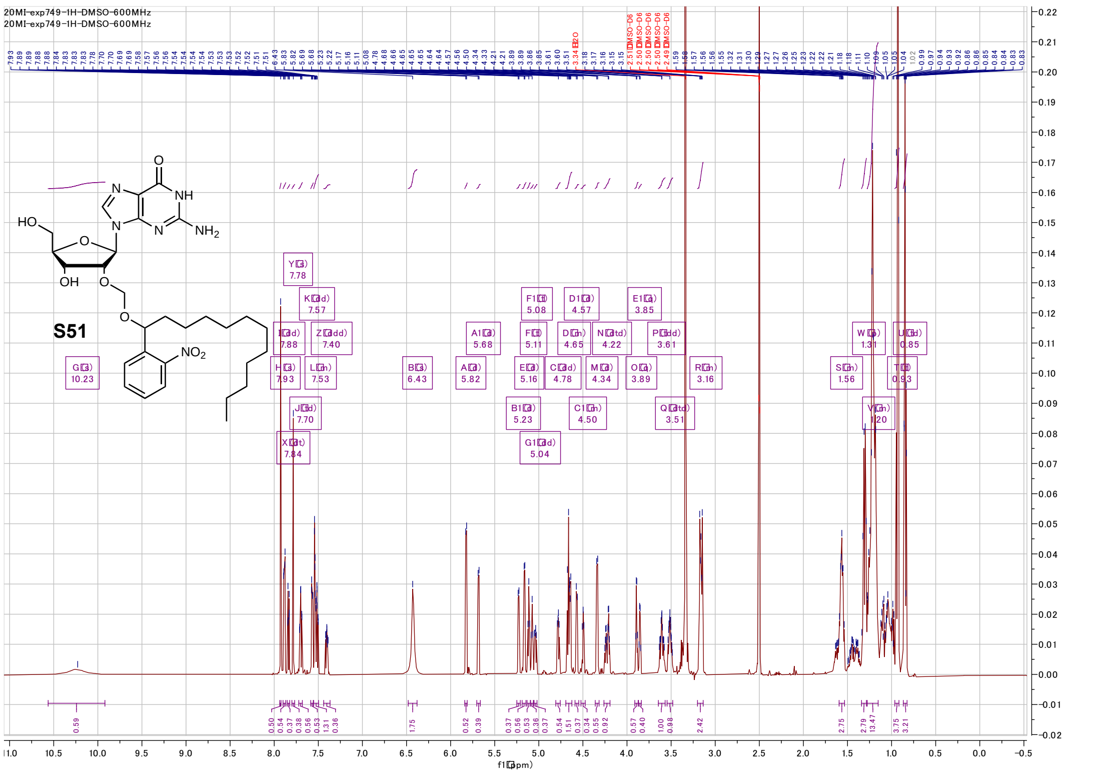

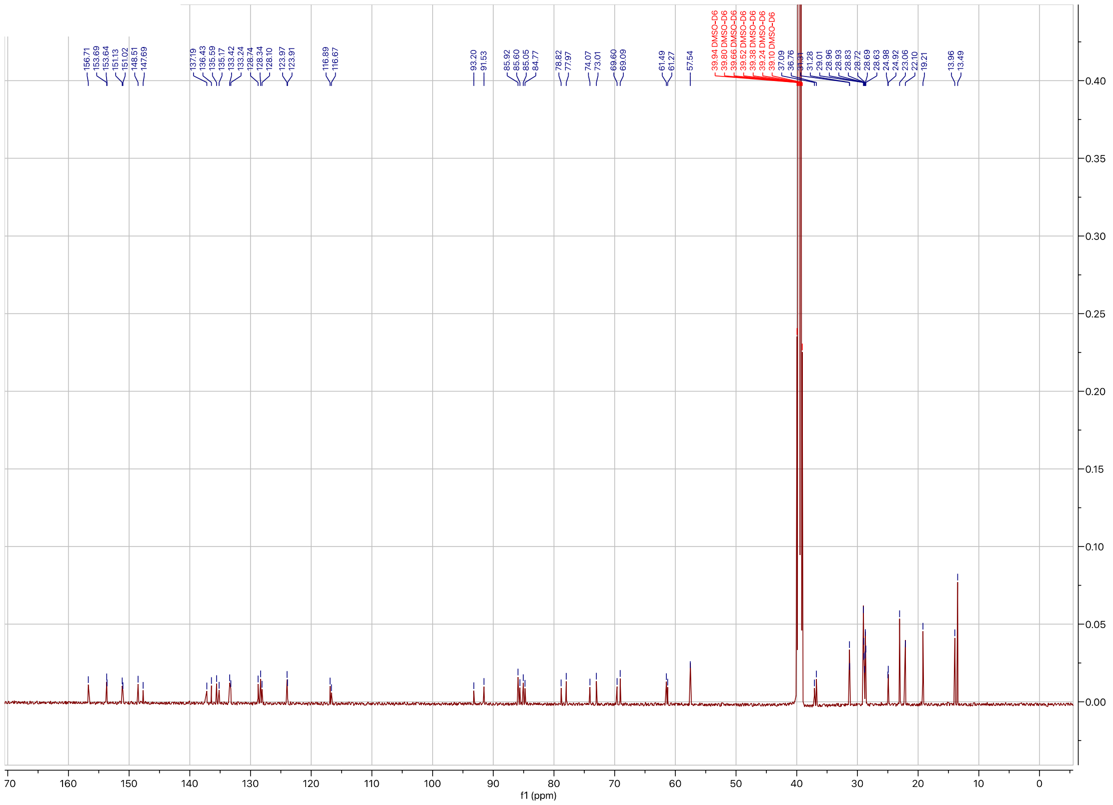

^{1}H and $^{13}C\{^{1}H\}$ NMR Spectra of Compound **S52**

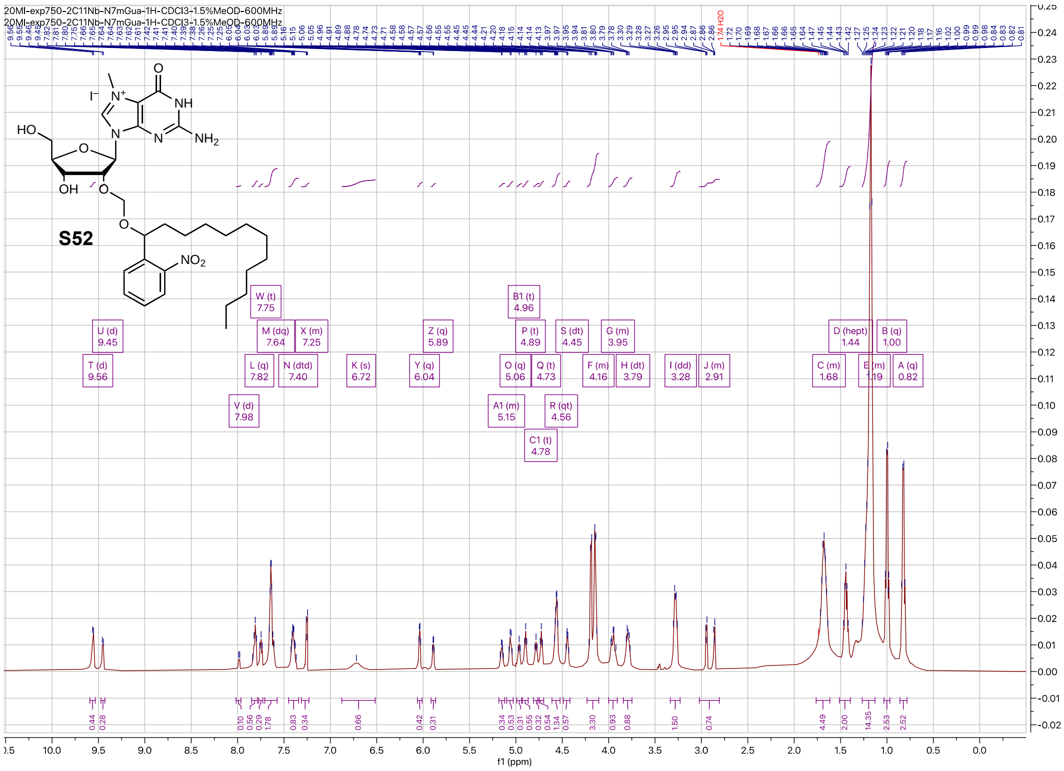

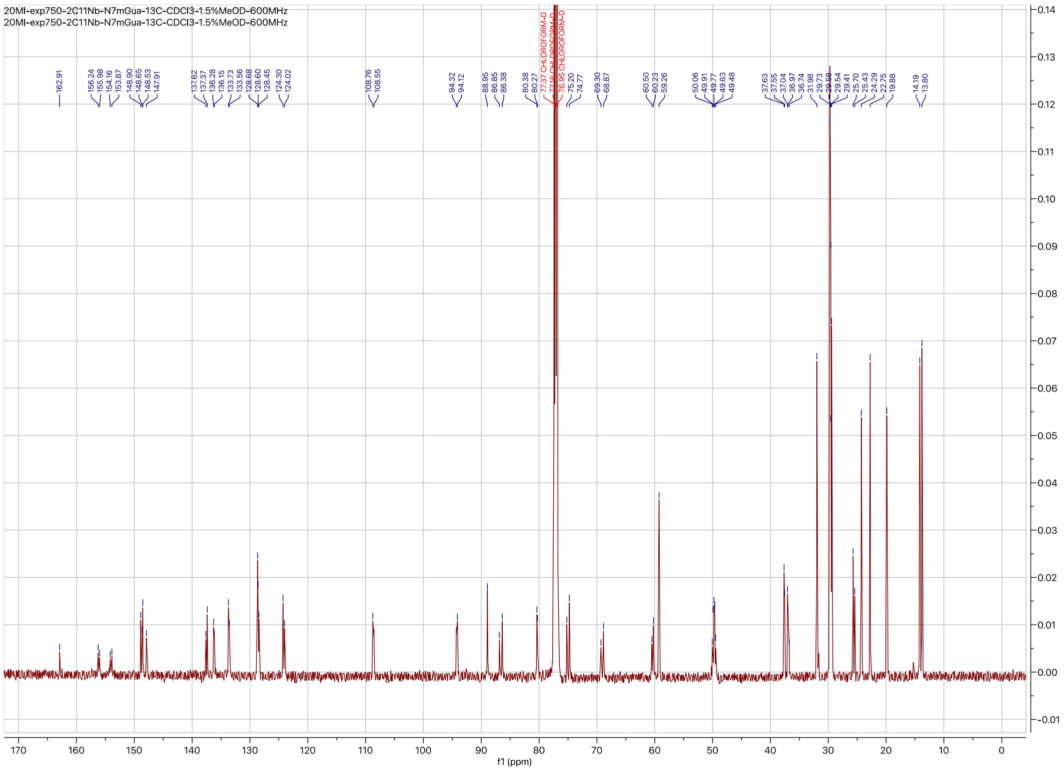

^{1}H and $^{13}C\{^{1}H\}$ NMR Spectra of Compound **S53**

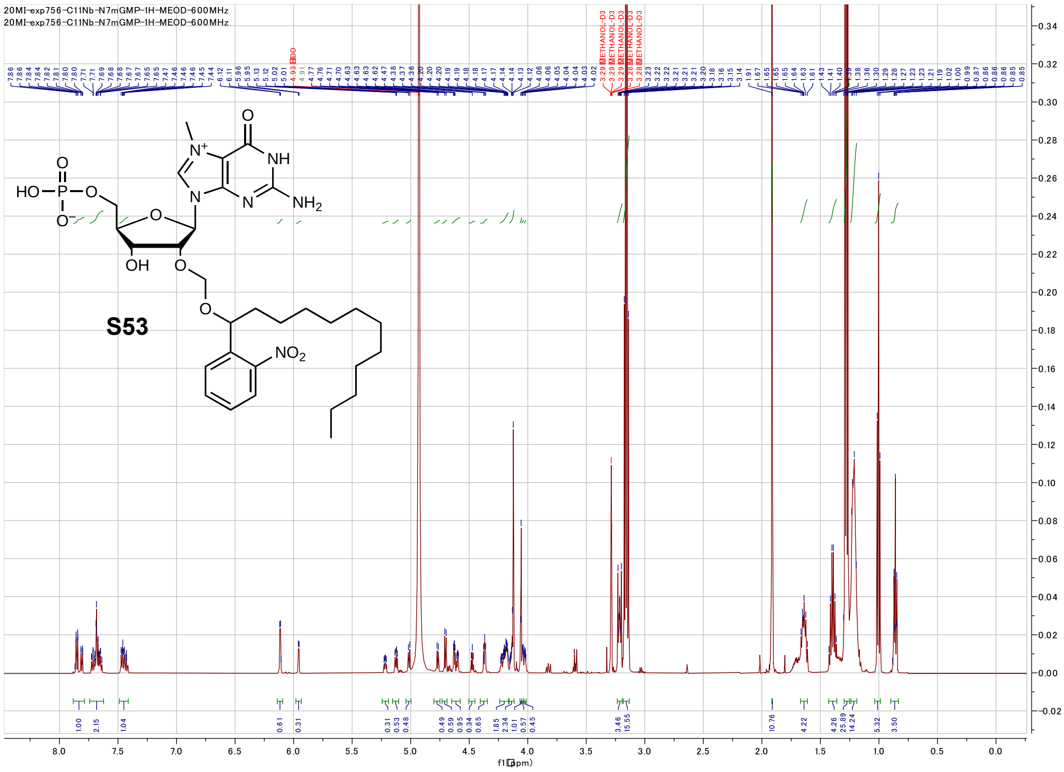

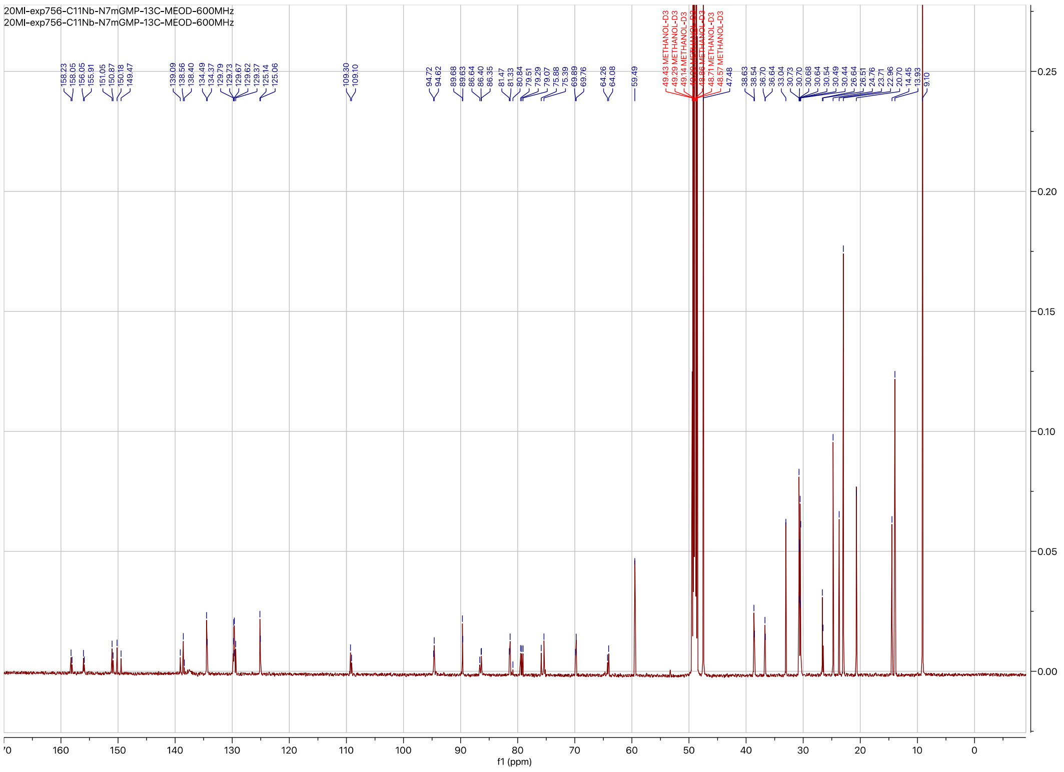

$^{31}P\{^1H\}$ NMR Spectrum of Compound **S53**

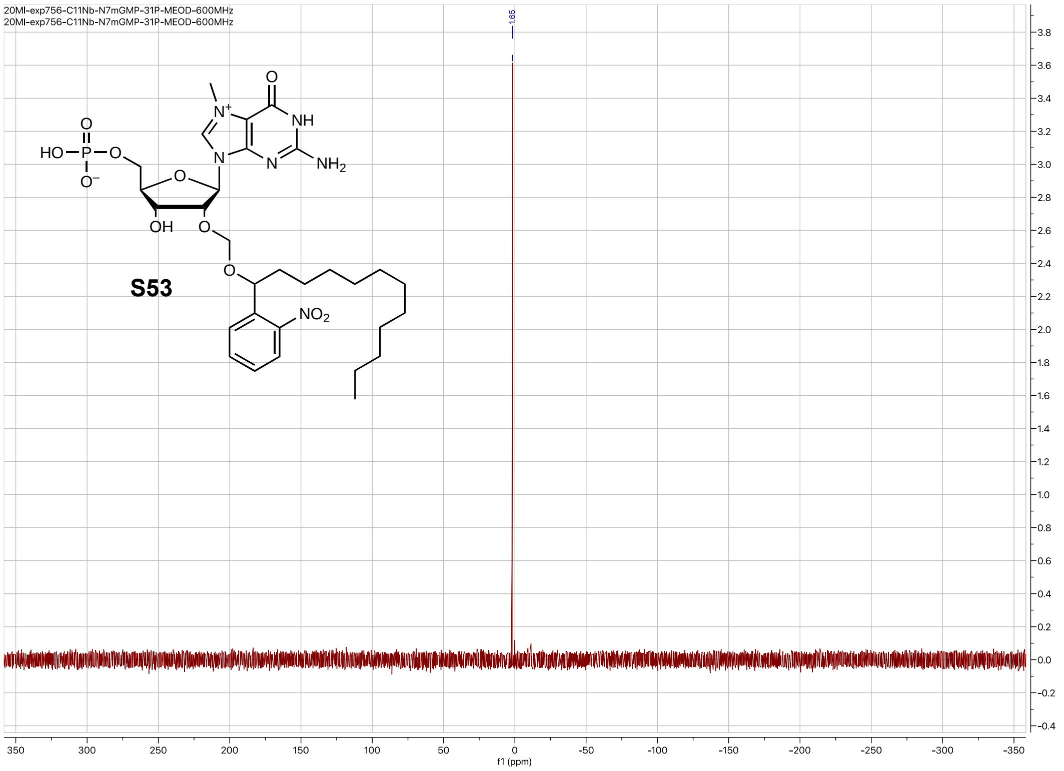

^{1}H and $^{13}C\{^{1}H\}$ NMR Spectra of Compound **25**

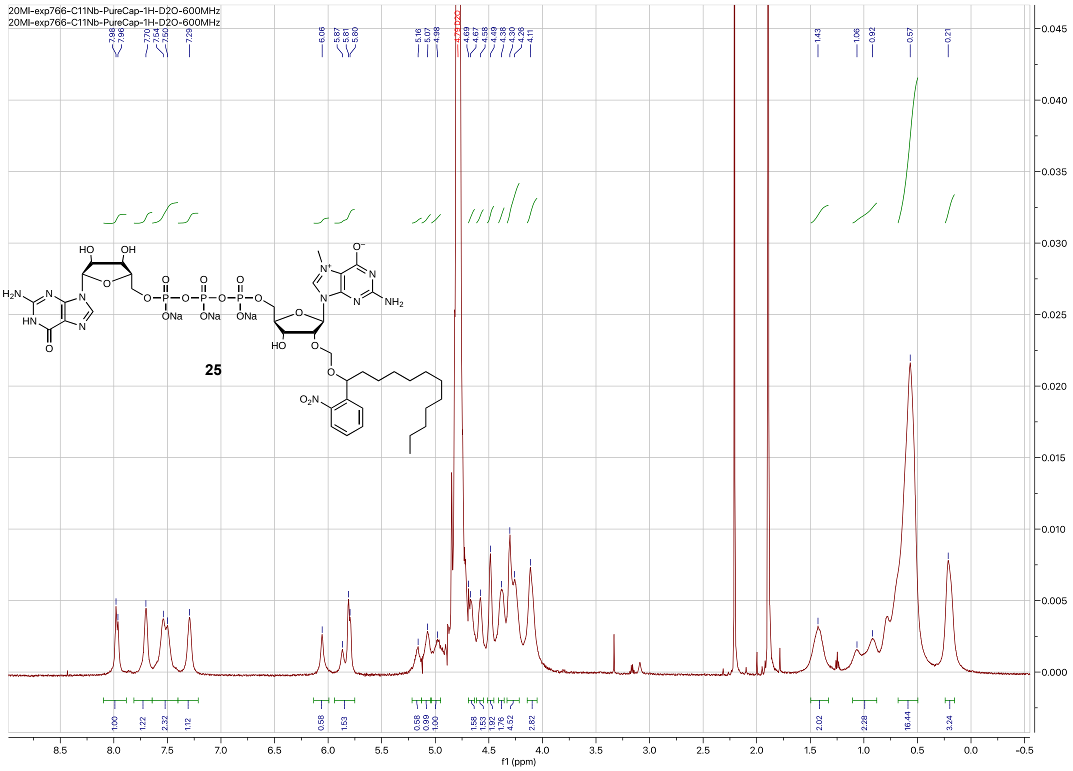

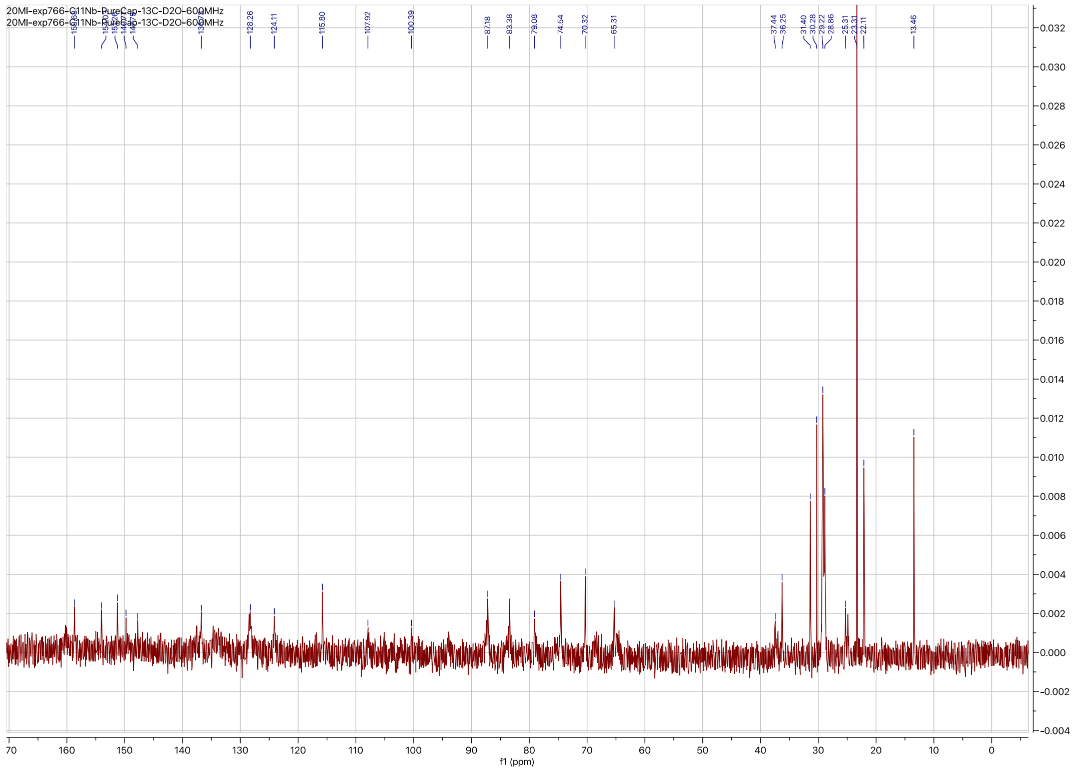

$^{31}P\{^{1}H\}$ NMR Spectrum of Compound **S25**

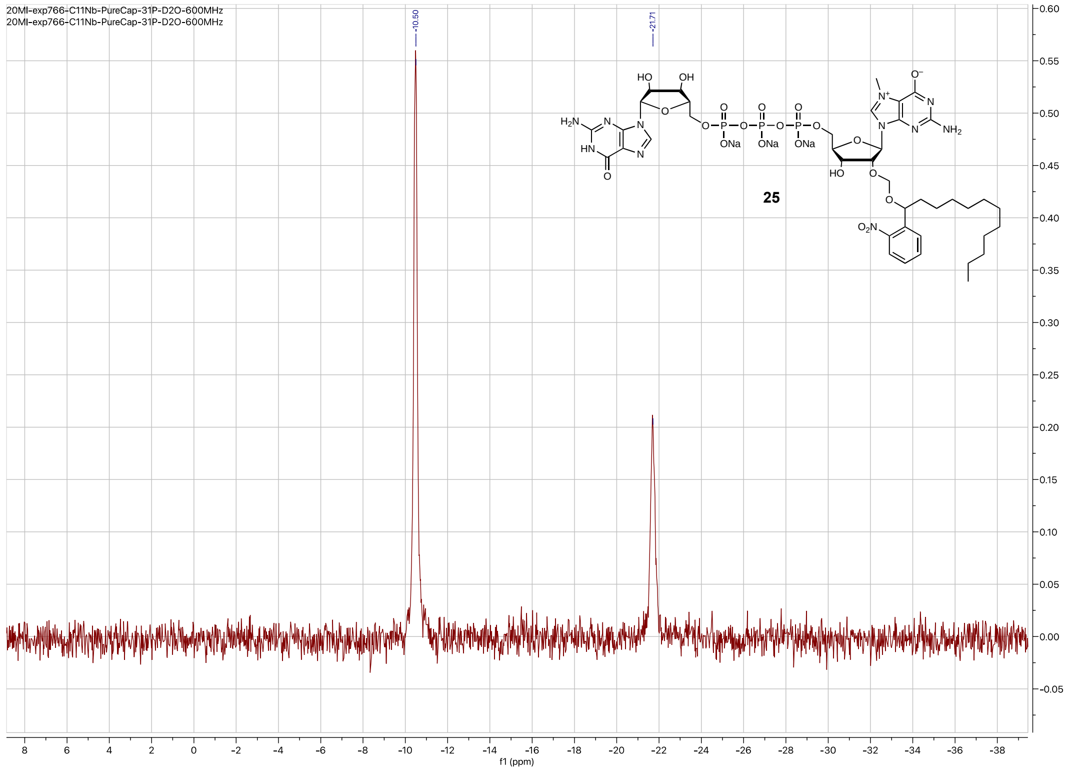

4. LC Profiles of Cap Analogs

HPLC Profile of Compound **1**

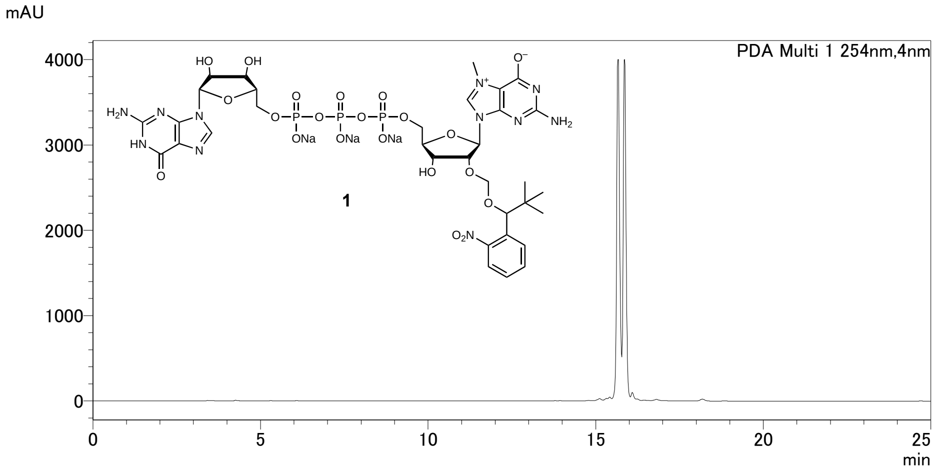

[Conditions] Column, YMC-Triart C8 (analytical, 4.6 mm I.D. × 25 cm); Solvent A, 50 mM TEAA buffer (pH 7.0) containing 5% CH_3CN; Solvent B, CH_3CN; linear gradient of Solvent B, 5–80% (25 min); flow rate, 1 mL/min; detection, 254 nm; column oven, 50 ºC.

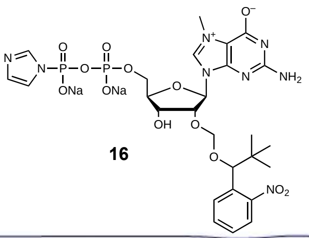

UPLC Profile of Compound **16**

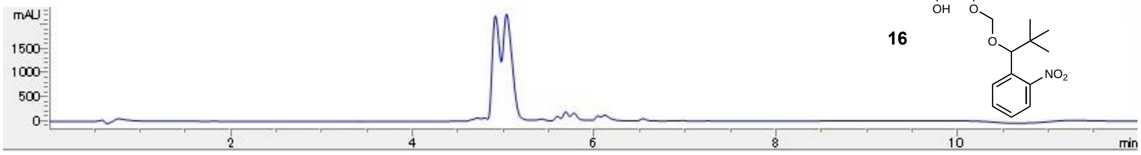

[Conditions] Column, ACQUITY UPLC BEH C18 Column (130 Å, 1.7 μm, 2.1 mm × 50 mm); Solvent A, 8.6 mM TEA / 100 mM HFIP; Solvent B, MeOH; linear gradient of Solvent B, 10 to 90% (7 min); flow rate, 0.3 mL/min; detected wavelength, 260 nm; column temp., 60 ºC.

HPLC Profile of Compound **2**

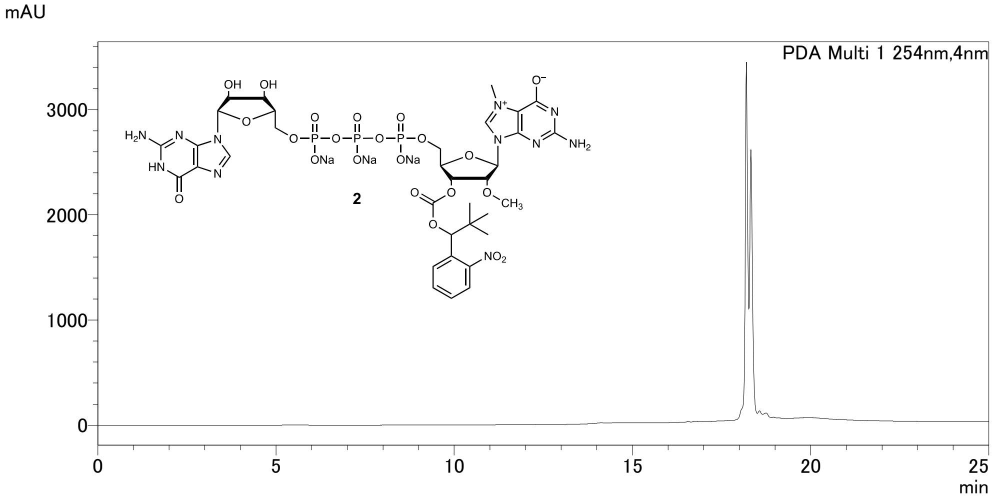

[Conditions] Column, YMC-Triart C8 (analytical, 4.6 mm I.D. × 25 cm); Solvent A, 0.1 M TEAB buffer (pH 7.9); Solvent B, CH_3CN; linear gradient of Solvent B, 5–80% (25 min); flow rate, 1 mL/min; detection, 254 nm; column oven, 50 ºC.

HPLC Profile of Compound **3**

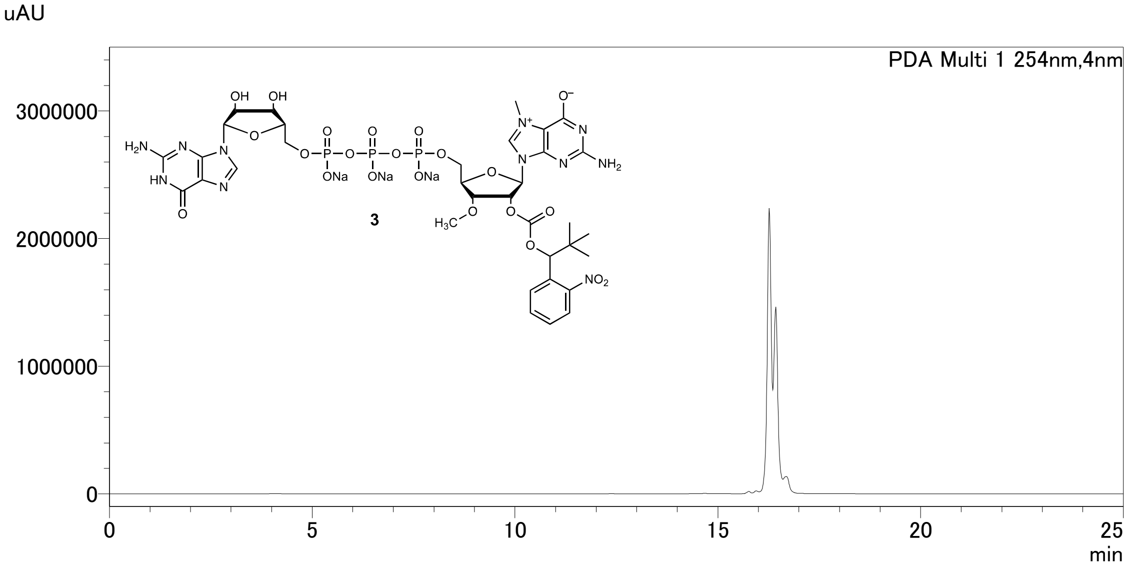

[Conditions] Column, YMC-Triart C8 (analytical, 4.6 mm I.D. × 25 cm); Solvent A, 50 mM TEAA buffer (pH 7.0) containing 5% CH_3CN; Solvent B, CH_3CN; linear gradient of Solvent B, 5–80% (25 min); flow rate, 1 mL/min; detection, 254 nm; column oven, 50 ºC.

HPLC Profile of Compound **4**

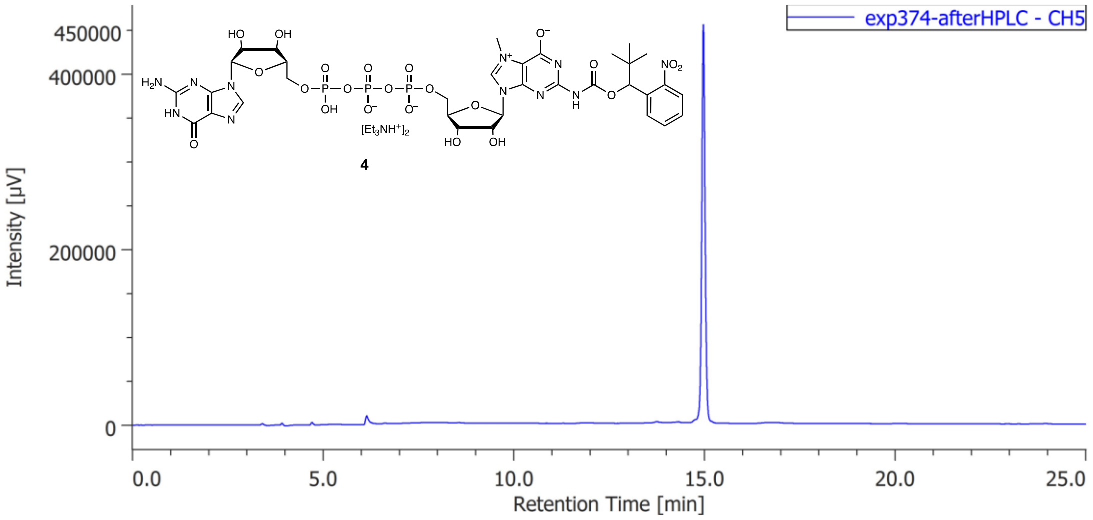

[Conditions] Column, YMC-Triart C8 (analytical, 4.6 mm I.D. × 25 cm); Solvent A, 50 mM TEAA buffer (pH 7.0) containing 5% CH_3CN; Solvent B, CH_3CN; linear gradient of Solvent B, 5–80% (25 min); flow rate: 1 mL/min; detection, 260 nm; column oven, 50 ºC.

HPLC Profile of Compound **5**

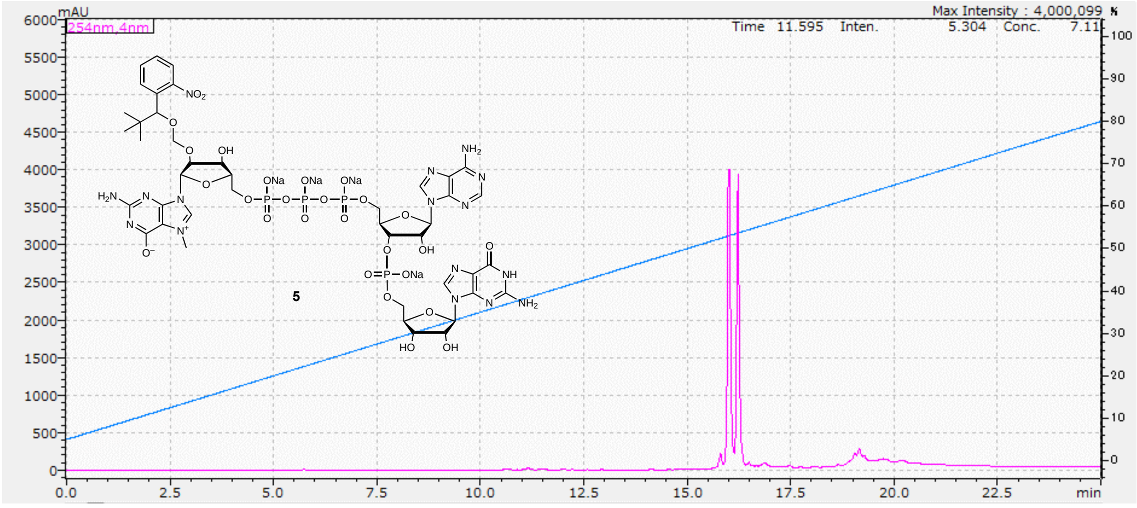

[Conditions] Column, YMC-Triart C8 (analytical, 4.6 mm I.D. × 25 cm; Solvent A, 0.1 M TEAB buffer (pH 7.9); Solvent B, CH_3CN; linear gradient of Solvent B, 5–80% (25 min); flow rate, 1 mL/min; detection, 254 nm; column oven, 50 ºC.

HPLC Profile of Compound **6**

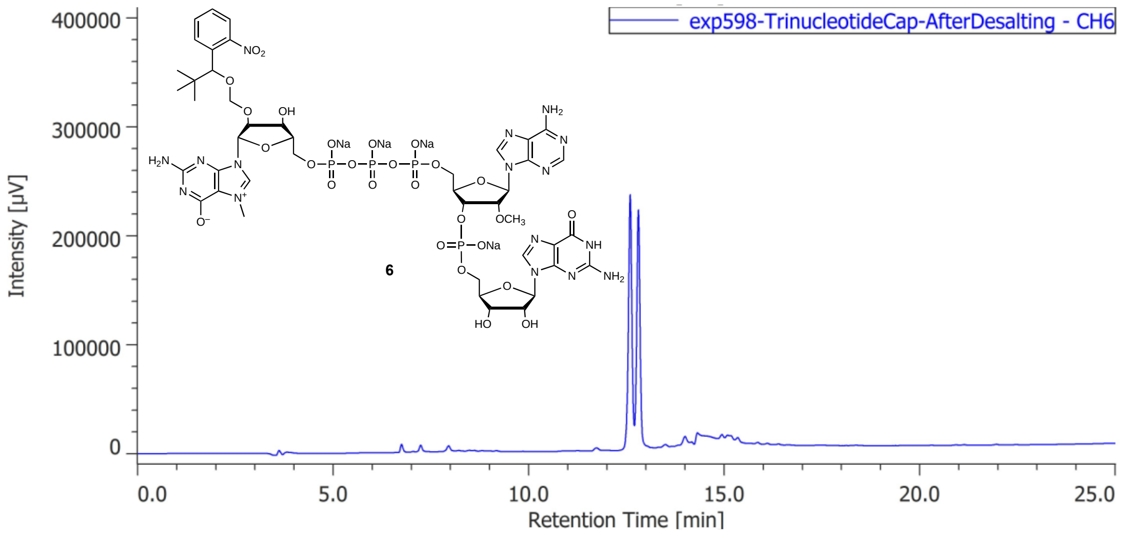

[Conditions] Column, YMC-Triart C8 (analytical, 4.6 mm I.D. × 25 cm); Solvent A, 0.1 M TEAB buffer (pH 7.9); Solvent B, CH_3CN; linear gradient of Solvent B, 5–80% (25 min); flow rate, 1 mL/min; detection, 260 nm; column oven, 50 ºC.

HPLC Profile of Compound **7**

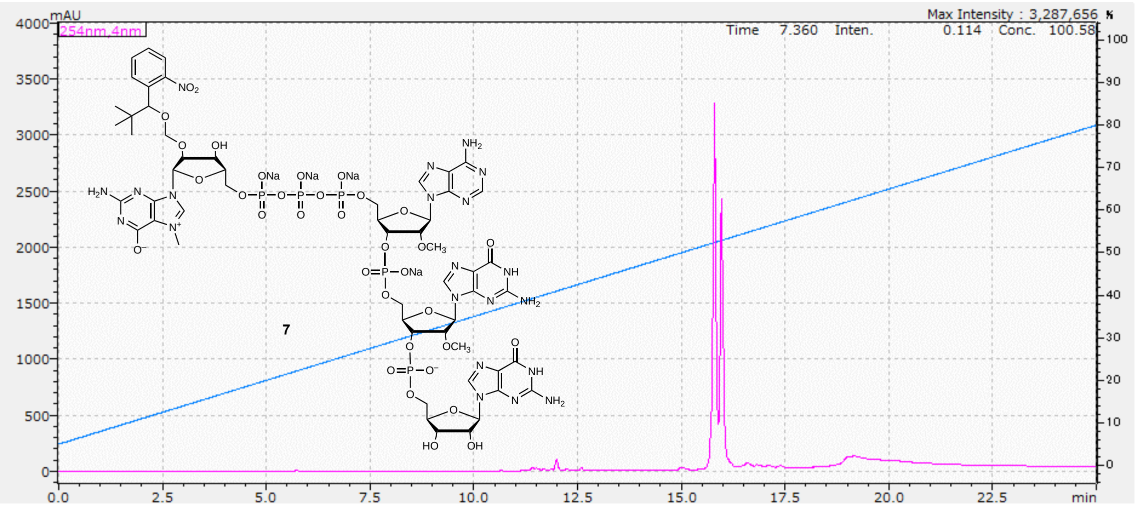

[Conditions] Column: YMC-Triart C8 (analytical, 4.6 mm I.D. × 25 cm); Solvent A, 0.1 M TEAB buffer (pH 7.9); Solvent B, CH_3CN; linear gradient of Solvent B, 5–80% (25 min); flow rate, 1 mL/min; detection, 254 nm; column oven, 50 ºC.

HPLC Profile of Compound **8**

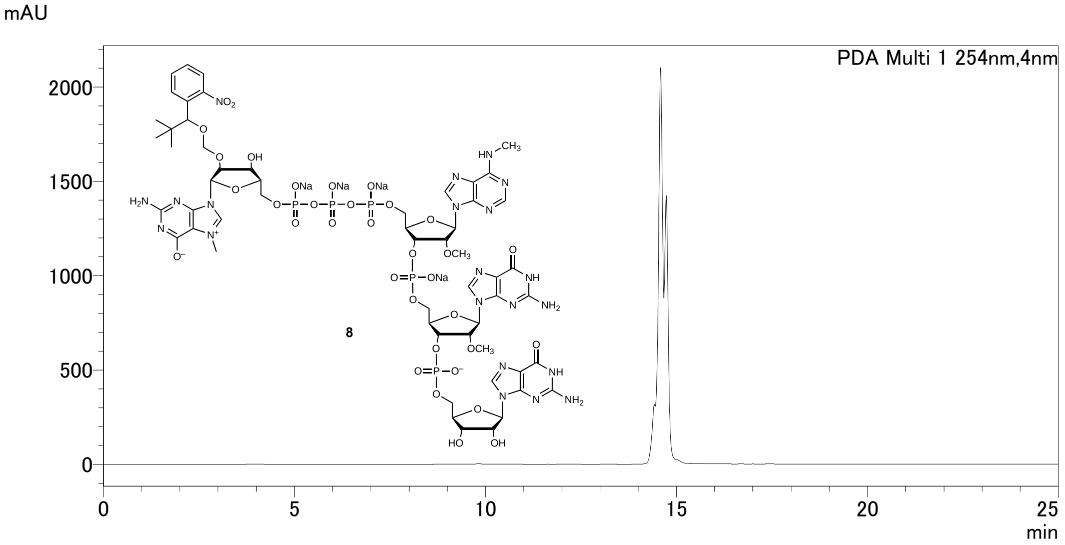

[Conditions] Column, YMC-Triart C8 (analytical, 4.6 mm I.D. × 25 cm); Solvent A, 0.1 M TEAB buffer (pH 7.9); Solvent B, CH_3CN; linear gradient of Solvent B, 5–80% (25 min); flow rate, 1 mL/min; detection, 254 nm; column oven, 50 ºC.

HPLC Profile of Compound **9**

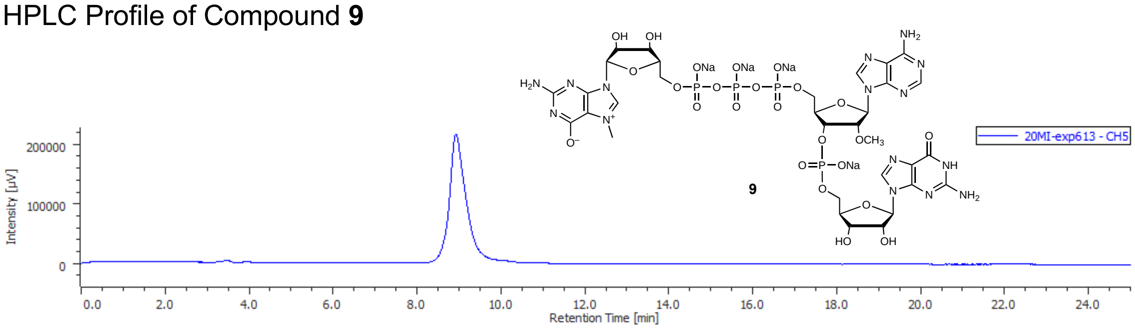

[Conditions] Column, YMC-Triart C8 (analytical, 4.6 mm I.D. × 25 cm); Solvent A, 0.1 M TEAB buffer (pH 7.9); Solvent B, CH_3CN; linear gradient of Solvent B, 5–80% (25 min); flow rate, 1 ml/min; detection, 260 nm; column oven, 50 ºC.

HPLC Profile of Compound **10**

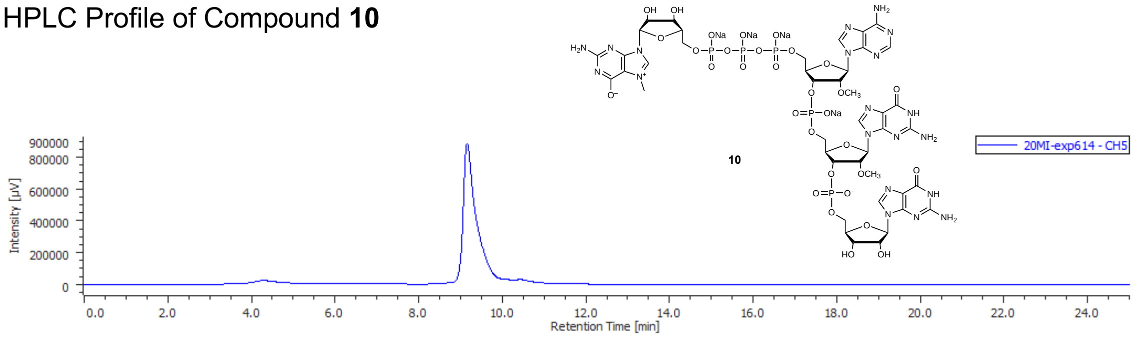

[Conditions] Column, YMC-Triart C8 (analytical, 4.6 mm I.D. × 25 cm); Solvent A, 0.1 M TEAB

buffer (pH 7.9); Solvent B, CH_3CN; linear gradient of Solvent B, 5–80% (25 min); flow rate, 1 mL/min; detection, 260 nm; column oven, 50 ºC.

HPLC Profiles of DiPure **1** (R = *tert*-Butyl), **23** (R = Phenylethyl), and **24** (R = *n*-Hexyl)

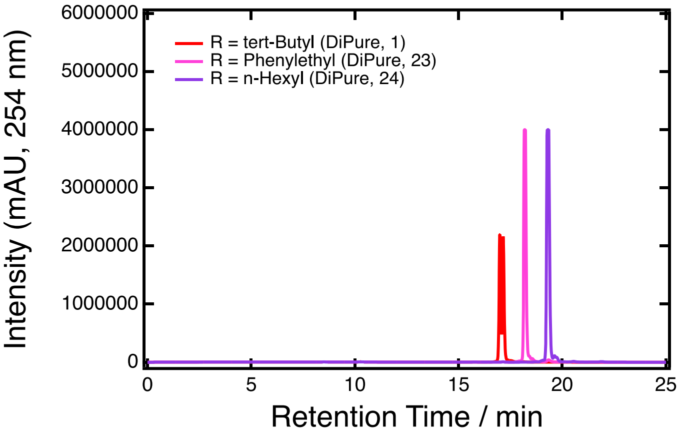

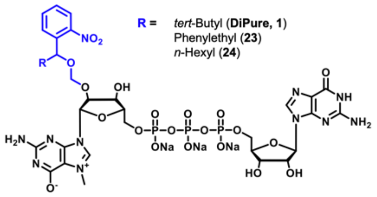

Modification	Retention Time
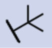	17.0 min
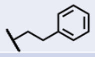	18.2 min
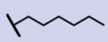	19.3 min

[Conditions] Column: YMC-Triart C8 (Analytical, 4.6 mm I.D. x 25 cm), Solvent A: 50 mM TEAA buffer (pH 6.0) contains 0.5% CH_3CN, Solvent B: CH_3CN, Linear Gradient 5–80%B (25 min), Flow Rate: 1 ml/min, Detection: 254 nm, Column oven: 50 ºC.

HPLC Profile of DiPure **25** (R = *n*-Undecyl)

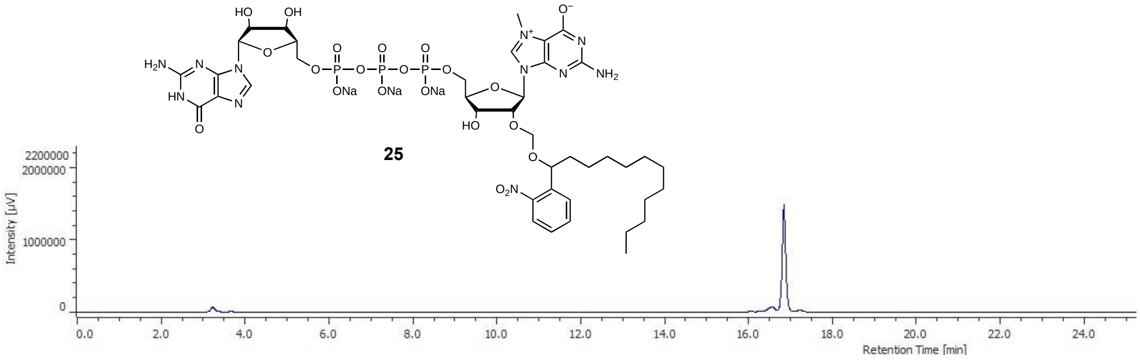

[Conditions] Column: YMC-Triart Bio C4 (Analytical, 4.6 mm I.D. x 25 cm), Solvent A: 50 mM TEAA buffer (pH 7.0) contains 5% CH_3CN, Solvent B: CH_3CN, Linear Gradient 5–80%B (25 min), Flow Rate: 1 ml/min, Detection: 260 nm, Column oven: 50 ºC

Supplementary Table 1. MALDI TOF MS data of 5' fragments originated from RP-HPLC purified capped Nluc mRNAs after cleavage by DNAzyme 10-23. Calculated and observed masses were shown. The cap analog used for the mRNA preparation is indicated in the table. AnP means it was dephosphorylated using Antarctic phosphatase after the RP-HPLC purification. The sequence shown as RNA is 5' -GCGCAUAUUAAGGUGACGCGUG-p (2',3' cyclic)

Sequence of RNA fragment	$[M+H]^+$ calcd	$[M+H]^+$ observed						
		**Cap(-)**	**ARCA**	**ARCA/AnP**	**DiPure**	**DiPure/N2**	**DiPure/3'OMe**	**DiPure/2'OMe**
**G-RNA**	7505.5			7506.0 (+0.5)				
**pp-G-RNA**	7665.4	7666.9 (+1.5)	7663.9 (-1.5)					
**ppp-G-RNA**	7745.4	7746.9 (+1.5)	7743.9 (-1.5)					
**m^7G-ppp-G-RNA**	8024.7				8025.2 (+0.5)	8026.3 (+1.6)		
**$m_2^{7,3'-O}$G-ppp-G-RNA**	8038.7		8038.7 (0)	8039.1 (+0.4)			8038.6 (-0.1)	
**$m_2^{7,2'-O}$G-ppp-G-RNA**	8038.7							8038.4 (-0.3)

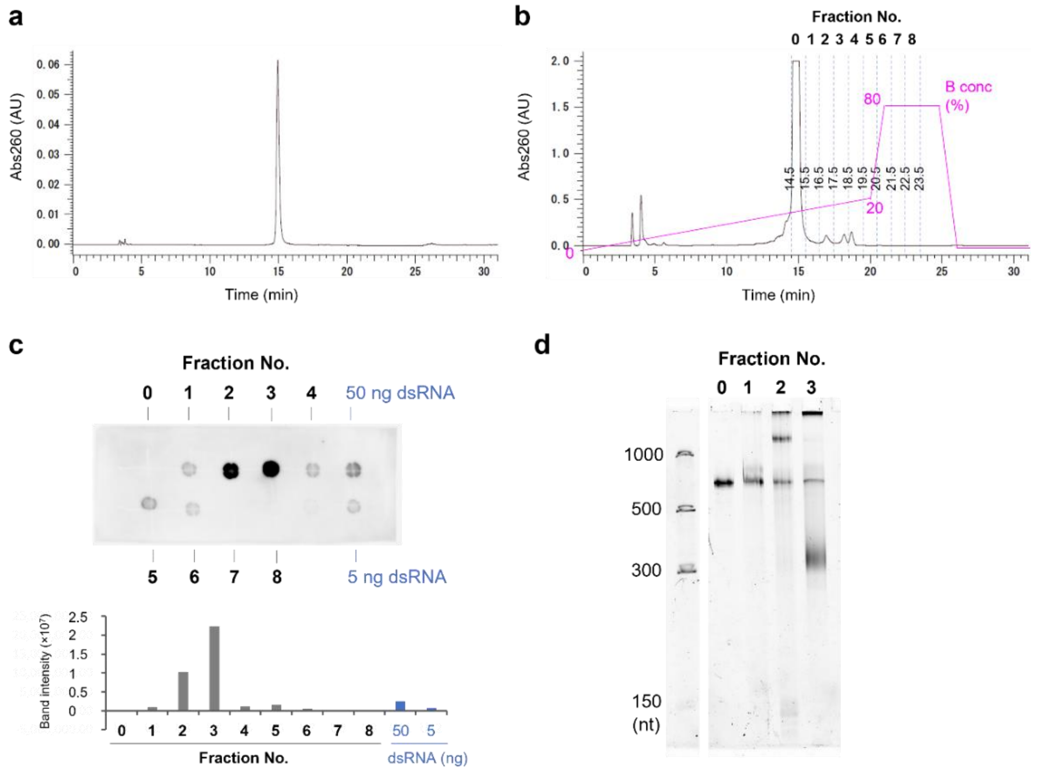

Supplementary Figure 15. Removal of double-stranded RNA (dsRNA) impurity from transcribed Nluc mRNA (650-nt) using reversed-phase HPLC. (**a, b**) Analytical (**a**) or preparative (**b**) RP-HPLC profiles of the crude RNA, using YMC Triart Bio C4 column (4.6 × 250 mm) using Solution_A [50 mM TEAA (pH 7), 5% acetonitrile] and Solution_B (acetonitrile). Solution_B content was increased from 0% to 20% over 20 minutes at a flow rate of 1 mL. The column temperature was maintained at 50 °C. (**b**) Eluted RNA was fractionated 1 min at a time, from 14.5 min to 23.5 min. (**c**) dsRNA detection by a dot blot assay using anti-dsRNA clone rJ2 antibody. An actual photograph of the blot and a graph of the signal quantification results were shown. The RNA fractions in (**b**) were concentrated, and 50 ng RNA was dotted. (**d**) 5% dPAGE analysis of fractionated RNAs (50 ng each). The gel was visualized by SYBR Green II staining. (**a**–**d**) Each experiment was repeated independently at least twice to obtain similar results. Source Data are provided with this paper.

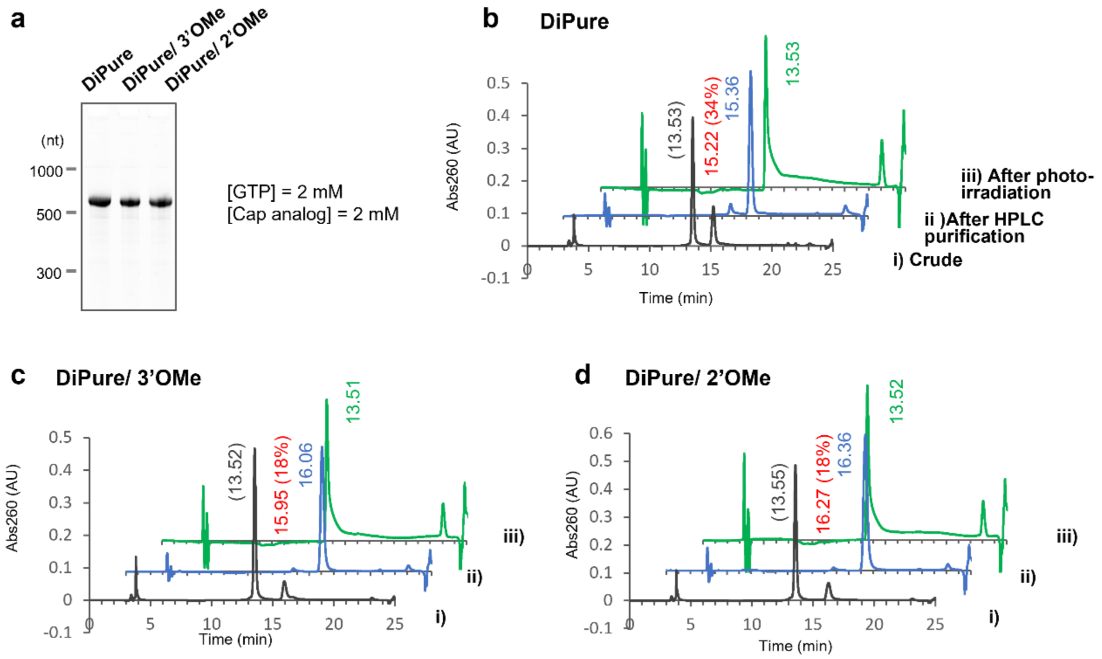

Supplementary Figure 16. IVT using dinuceotide PureCap cap analogs. Nluc mRNA (650-nt) was transcribed by T7 RNAP in the presence of cap analogs. (**a**) dPAGE analysis of the transcription reaction. (**b**-**d**) RP HPLC analysis of the RNA transcript. The transcript was analyzed as a crude mixture (**i**, black line) or after being purified by preparative HPLC (**ii**, blue line). Purified RNA was analyzed after deprotection by irradiating 365 nm light (**iii**, green line). The elution time (min) of the peak was noted nearby. A ratio (%) of capped mRNA calculated based on the peak area was listed in parentheses after the elution time. (**a**–**d**) Each experiment was repeated independently at least twice to obtain similar results. Source Data are provided with this paper.

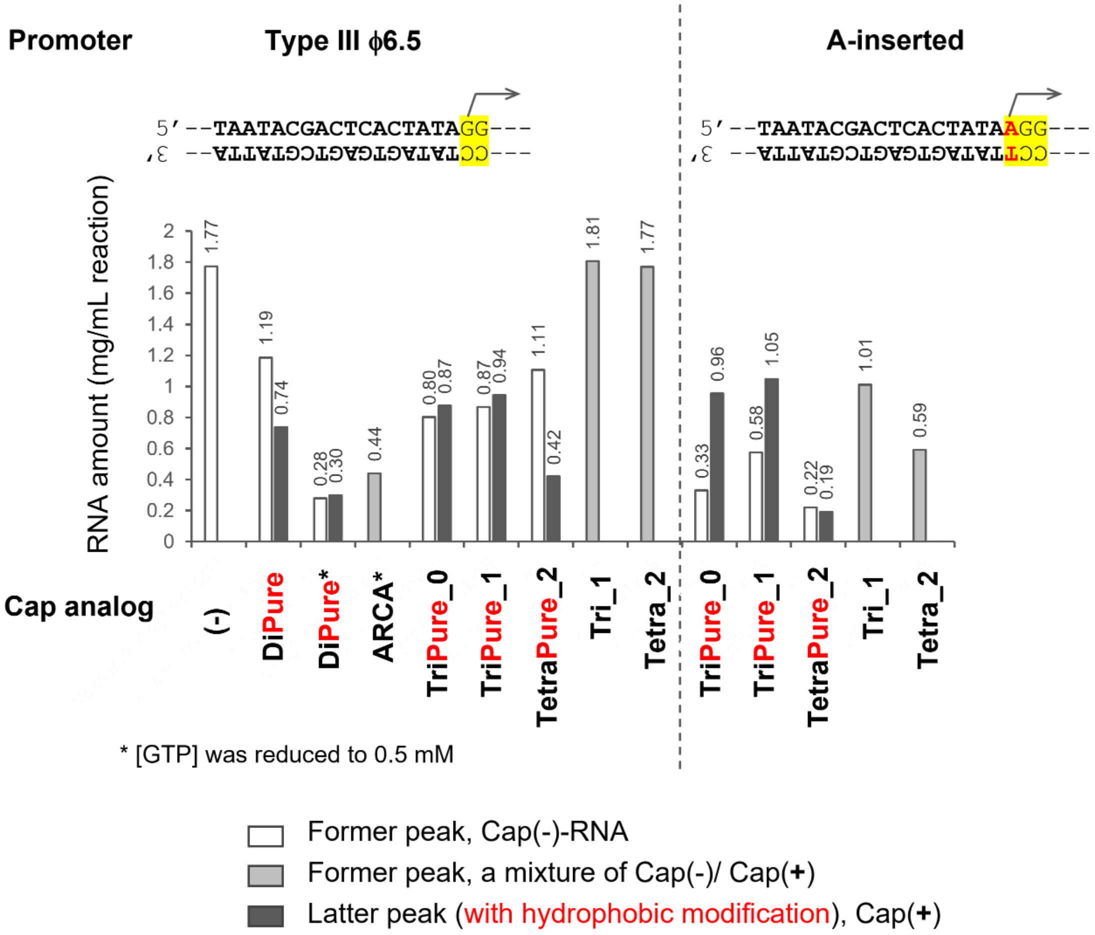

Supplementary Figure 17. RP-HPLC quantification of the capped/uncapped transcripts in the IVT reaction using PureCap analogs. 650-nt Nluc mRNA was transcribed from a DNA template containing a canonical Type III ϕ6.5 promoter or a modified promoter (“A-inserted”). The cap analog added to the reaction was indicated in the graph. Each experiment was repeated independently at least three times to obtain similar results.

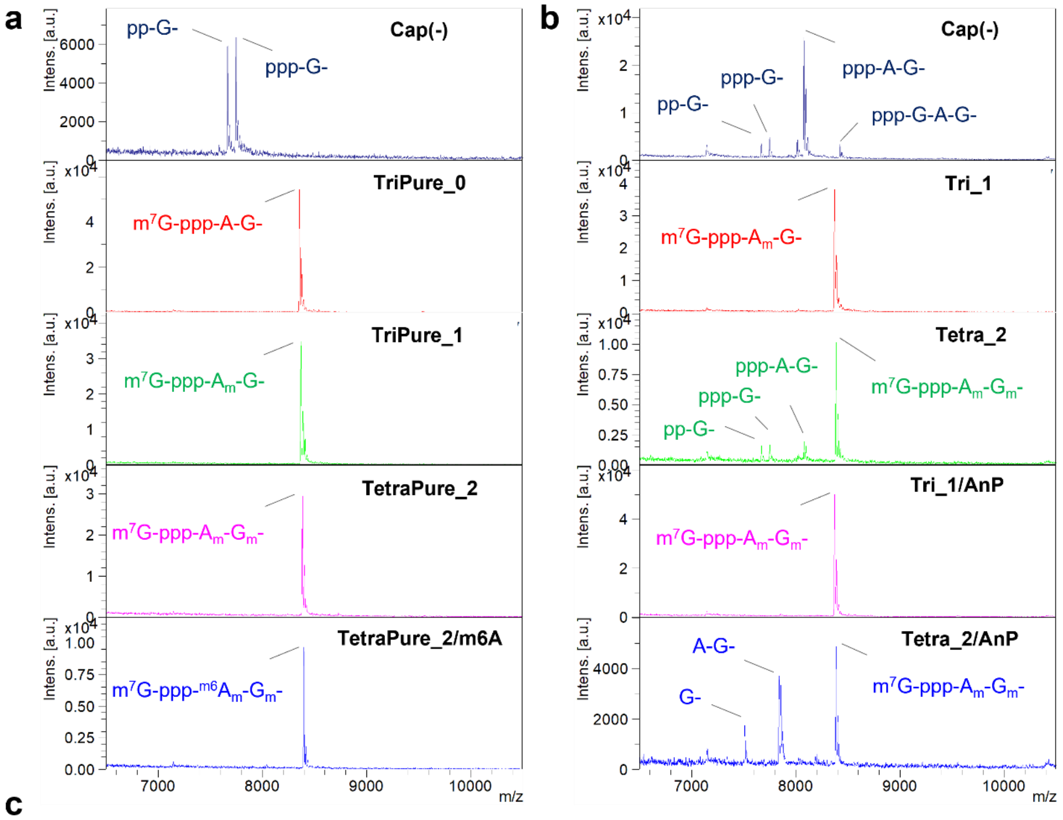

c

Sequence of RNA fragment	$[M+H]^+$ calcd	Promoter sequence									
		Type III phi6.5 (panel_**a**)					A-inserted (panel_**b**)				
		**Cap(-)**	**TriPure_0**	**TriPure_1**	**TetraPure_2**	**TetraPure_2/m6A**	**Cap(-)**	**Tri_1**	**Tetra_2**	**Tri_1/AnP**	**Tetra_2/AnP**
**G-RNA**	7505.5										7510.2 (+4.7)
**pp-G-RNA**	7665.4	7666.5 (+1.1)					7667.9 (+2.5)		7669.5 (+4.1)		
**ppp-G-RNA**	7745.4	7746.2 (+0.8)					7748.1 (+2.7)		7749.9 (+4.5)		
**A-G-RNA**	7834.7										7839.3 (+4.6)
**pp-A-G-RNA**	7994.7										
**ppp-A-G-RNA**	8074.7						8077.3 (+2.7)		8078.4 (+3.8)		
**ppp-G-A-G-RNA**	8419.9						8422.4 (+2.5)				
**m^7G-ppp-A-G-RNA**	8353.9		8356.6 (+2.7)								
**m^7G-ppp-A_m-G-RNA**	8367.9			8370.2 (+2.3)				8371.4 (+3.5)		8371.6 (+3.7)	
**m^7G-ppp-A_m-G_m-RNA**	8381.9				8384.8 (+2.9)				8385.8 (+3.9)		8386.6 (+4.7)
**m^7G-ppp-m^6A_m-G_m-RNA**	8395.9					8398.0 (+2.1)					

Supplementary Figure 18. MALDI-TOF MS analysis of 5' terminus of capped Nluc mRNAs prepared using tri/tetranucleotide cap analogs. The names of the cap analogs are shown in the figure; AnP means that the RNA was dephosphorylated using Antarctic phosphatase. Capped mRNAs prepared from a DNA template containing Type III ϕ6.5 promoter (**a**) or a modified T7 promoter named “A-inserted” (**b**) were analyzed. The 5' fragment was generated by cleavage with DNAzyme. (**c**) Calculated and observed masses shown in panels (**a**) and (**b**) are listed. The sequence shown as RNA is 5'-GCGCAUAUUAAGGUGACGCGUG-p (2',3' cyclic). All prominent peaks were accompanied by a peak with a molecular weight of +18, probably due to hydrolysis of the 2' and 3' cyclic monophosphate initially present at the 3' end of the RNA fragment. (**a**, **b**) Each experiment was repeated independently at least twice to obtain similar results.

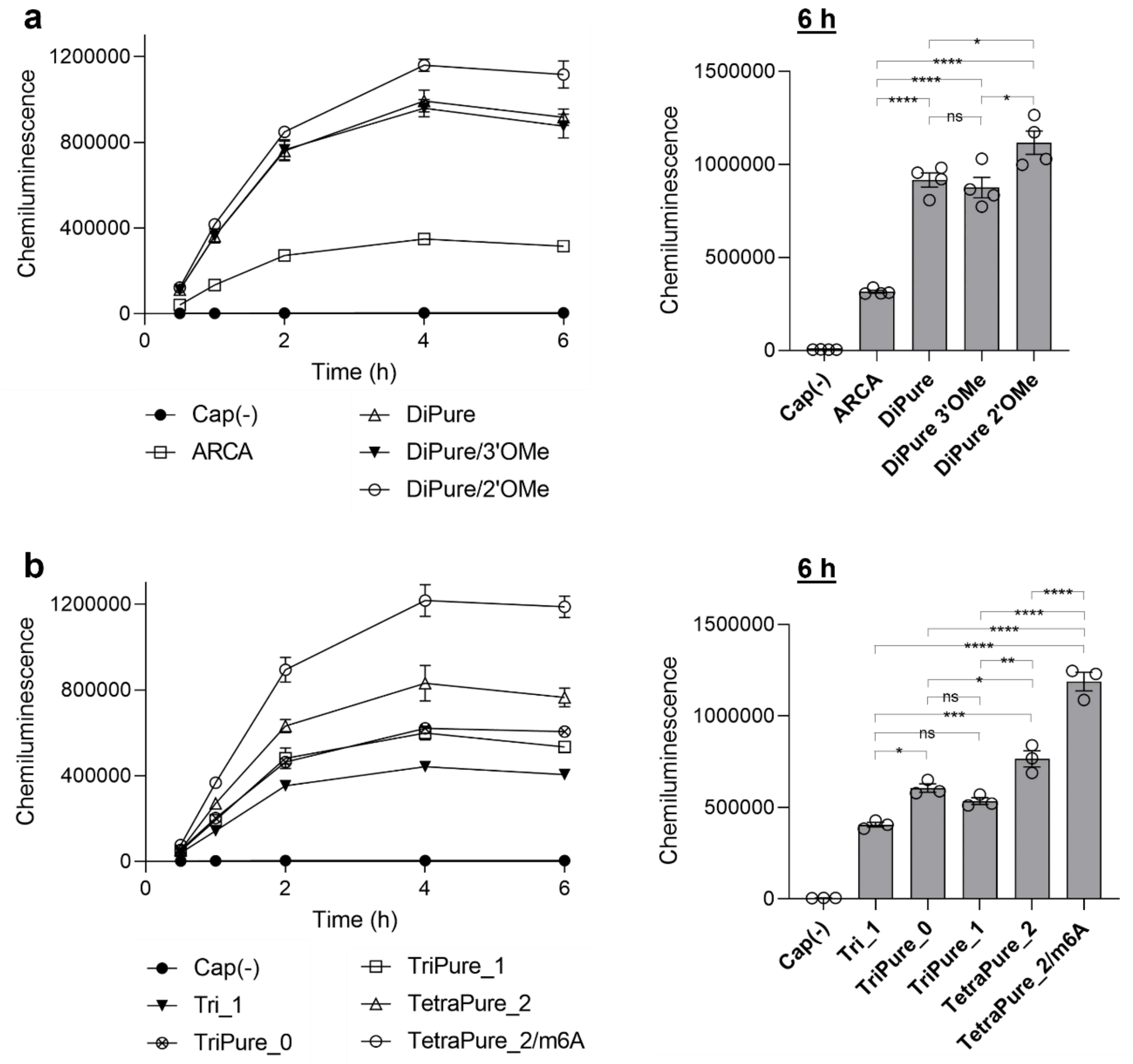

Supplementary Figure 19. Nluc mRNAs with different cap structures were compared in a HeLa S3-based cell-free translation system. Capped mRNAs prepared using dinucleotide (**a**) or tri/tetranucleotide cap analogs (**b**) were compared. Time-course of the expression profiles (left) and the extracted data at 6-h (right) were shown. Data are mean ± SEM for biological replicates (n = 3 or 4). One-way ANOVA test between capped mRNAs followed by the Tukey's test was marked as follows: ns, p > 0.05 (not significant); *, p < 0.05; **, p < 0.01; ***, p < 0.001, ****, p < 0.0001. The capping efficiency of these mRNAs, reproduced from Figs 3f and 5g, were as follows: ARCA, 56%; DiPure, >99%; DiPure/3'OMe, >99%; DiPure/2'OMe, >99%; Tri_1, 87%, TriPure_0, >99%; TriPure_1, >99 %; TetraPure_2, 98 %, TetraPure_2/m6A, 95 %. Each experiment was repeated independently twice to obtain similar results. Source Data are provided with this paper.

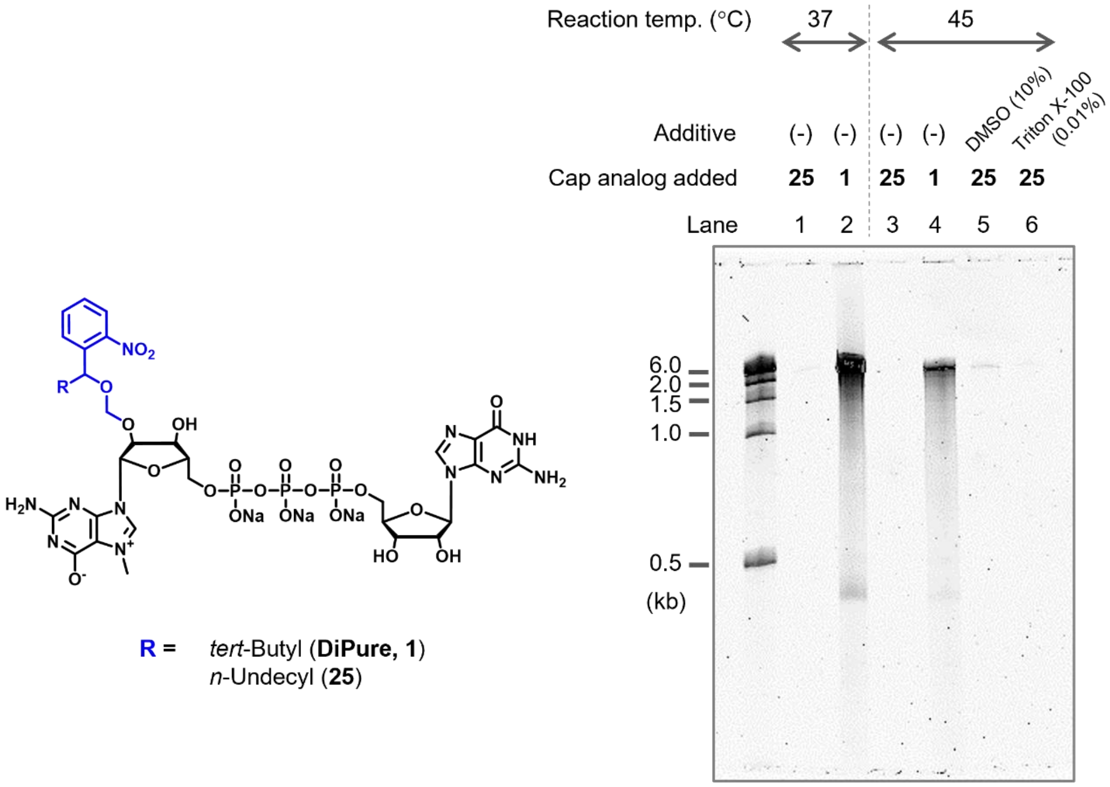

Supplementary Figure 20. Denaturing PAGE analysis of the IVT reaction with a PureCap analog **25**. 2.1 kb RNA was transcribed from a DNA template encoding firefly luciferase in the reaction mixture [5 ng/μL DNA, 2 mM ATP, 2 mM UTP, 2 mM GTP, 2 mM CTP, 2 mM PureCap analog (**1** or **25**), 40 mM Tris-HCl (pH 8.0), 8 mM $MgCl_2$, 2 mM spermidine, 5 mM DTT, 0.002 U/μL inorganic pyrophosphatase, 4.7 ng/μL T7 RNA polymerase]. The addition of DMSO (at a final concentration of 10 v/v%) and Triton X-100 (at a final concentration of 0.01 v/v%) was tested to see if the incorporation of the cap analog **25** was enhanced. After incubation of the reaction mixture at 37 ºC or 45 ºC for 1.5 h, DNase I was added to the mix at a final concentration of 0.1 units/μL and further incubated for 15 min. An aliquot was taken from the mixture, and they were analyzed by 5% denaturing PAGE containing 7.5 M urea. The gel was visualized by SYBR Green II staining. Source Data are provided with this paper.

5. Supplementary References

1. Litosh VA, Wu WD, Stupi BP, Wang JC, Morris SE, Hersh MN, et al. Improved nucleotide selectivity and termination of 3'-OH unblocked reversible terminators by molecular tuning of 2-nitrobenzyl alkylated HOMedU triphosphates. *Nucleic Acids Res* **39**, e39 (2011).
2. Featherston AL, Kwon Y, Pompeo MM, Engl OD, Leahy DK, Miller SJ. Catalytic asymmetric and stereodivergent oligonucleotide synthesis. *Science* **371**, 702-707 (2021).
3. Rydzik AM, Warminski M, Sikorski PJ, Baranowski MR, Walczak S, Kowalska J, et al. mRNA cap analogues substituted in the tetraphosphate chain with CX2: identification of O-to-CCl2 as the first bridging modification that confers resistance to decapping without impairing translation. *Nucleic Acids Res* **45**, 8661-8675 (2017).
4. Chaulk SG, MacMillan AM. Synthesis of oligo-RNAs with photocaged adenosine 2 '-hydroxyls. *Nat Protoc* **2**, 1052-1058 (2007).
5. Mukobata T, Ochi Y, Ito Y, Wada S, Urata H. Facile and efficient approach for the synthesis of N-2-dimethylaminomethylene-2 '-O-methylguanosine. *Bioorg Med Chem Lett* **20**, 129-131 (2010).
6. Agarwal V, Vondenhoff G, Gadakh B, Severinov K, Van Aerschot A, Nair SK. Exploring the substrate promiscuity of an antibiotic inactivating enzyme. *MedChemComm* **5**, 1567-1570 (2014).
7. Sikorski PJ, Warminski M, Kubacka D, Ratajczak T, Nowis D, Kowalska J, et al. The identity and methylation status of the first transcribed nucleotide in eukaryotic mRNA 5 ' cap modulates protein expression in living cells. *Nucleic Acids Res* **48**, 1607-1626 (2020).
