# Supplementary material for: Cap analogs with a hydrophobic photocleavable tag enable facile purification of fully capped mRNA with various cap structures
Source: Nat Commun. 2023 May 11;14:2657. doi: 10.1038/s41467-023-38244-8 (PMC10175277; doi:10.1038/s41467-023-38244-8)
Supplement: Supplementary file 3 — Reporting Summary [file 41467_2023_38244_MOESM3_ESM.pdf]

nature portfolio

Corresponding author(s): Hiroshi Abe

Last updated by author(s): 2023/4/20

Reporting Summary

Nature Portfolio wishes to improve the reproducibility of the work that we publish. This form provides structure for consistency and transparency in reporting. For further information on Nature Portfolio policies, see our Editorial Policies and the Editorial Policy Checklist.

Statistics

For all statistical analyses, confirm that the following items are present in the figure legend, table legend, main text, or Methods section.

n/a	Confirmed	
☐	☒	The exact sample size (*n*) for each experimental group/condition, given as a discrete number and unit of measurement
☐	☒	A statement on whether measurements were taken from distinct samples or whether the same sample was measured repeatedly
☐	☒	The statistical test(s) used AND whether they are one- or two-sided<br>*Only common tests should be described solely by name; describe more complex techniques in the Methods section.*
☒	☐	A description of all covariates tested
☒	☐	A description of any assumptions or corrections, such as tests of normality and adjustment for multiple comparisons
☐	☒	A full description of the statistical parameters including central tendency (e.g. means) or other basic estimates (e.g. regression coefficient) AND variation (e.g. standard deviation) or associated estimates of uncertainty (e.g. confidence intervals)
☐	☒	For null hypothesis testing, the test statistic (e.g. *F*, *t*, *r*) with confidence intervals, effect sizes, degrees of freedom and *P* value noted<br>*Give P values as exact values whenever suitable.*
☒	☐	For Bayesian analysis, information on the choice of priors and Markov chain Monte Carlo settings
☒	☐	For hierarchical and complex designs, identification of the appropriate level for tests and full reporting of outcomes
☒	☐	Estimates of effect sizes (e.g. Cohen's *d*, Pearson's *r*), indicating how they were calculated

Our web collection on statistics for biologists contains articles on many of the points above.

Software and code

Policy information about availability of computer code

Data collection	ImageLab Touch (BioRad ChemiDoc Touch imager), Chromaster (HItachi HPLC), LabSolutions (Shimadzu HPLC), ChromNAV Ver.2 (JASCO HPLC), ChemStation (Agilent UPLC), FlexControl (MALDI-TOF MS), JNM-ECS400 (JEOL NMR), JNM-ECA600 (JEOL NMR), MikroWin or ICE (Berthold plate reader TriStar2 or TriStar5, respectively), QTofControl 3.2 (Bruker Daltonics ESI QTOF)
Data analysis	Delta 5.2.1 or MestReNova v14.2.0-26256 (NMR data analysis), ChemDraw 20.0 (mass calc), ImageLab 6.1 (Gel imaging), FlexAnalysis 3.4 (MALDI-TOF MS), Microsoft Excel for Microsoft 365 MSO ver 2302, GraphPad Prism 9 and KaleidaGraph 5.0 (Statistical analysis), Compass DataAnalysis 5.1 (Bruker Daltonics ESI QTOF)

For manuscripts utilizing custom algorithms or software that are central to the research but not yet described in published literature, software must be made available to editors and reviewers. We strongly encourage code deposition in a community repository (e.g. GitHub). See the Nature Portfolio guidelines for submitting code & software for further information.

Data

Policy information about availability of data

All manuscripts must include a data availability statement. This statement should provide the following information, where applicable:

- Accession codes, unique identifiers, or web links for publicly available datasets
- A description of any restrictions on data availability
- For clinical datasets or third party data, please ensure that the statement adheres to our policy

The data supporting the findings of this study are available in this article, in the supplementary information file and in the source data files.

Human research participants

Policy information about studies involving human research participants and Sex and Gender in Research.

Reporting on sex and gender	Not applicable to this study.
Population characteristics	Not applicable to this study.
Recruitment	Not applicable to this study.
Ethics oversight	Not applicable to this study.

Note that full information on the approval of the study protocol must also be provided in the manuscript.

Field-specific reporting

Please select the one below that is the best fit for your research. If you are not sure, read the appropriate sections before making your selection.

☒ Life sciences ☐ Behavioural & social sciences ☐ Ecological, evolutionary & environmental sciences

For a reference copy of the document with all sections, see nature.com/documents/nr-reporting-summary-flat.pdf

Life sciences study design

All studies must disclose on these points even when the disclosure is negative.

Sample size	Biological test were performed in triplicates, quadruplicates or larger. No statistical method was used to predetermine the sample size.
Data exclusions	No data was excluded as outliers from the analyses.
Replication	Multiple independent experiments were performed to confirm the reproducibilities.
Randomization	Randomization was not relevant for this study, as we did not perform group comparisons.
Blinding	No blinding was performed in this study, as no group comparisons were performed and there was no specific reason to expect bias.

Reporting for specific materials, systems and methods

We require information from authors about some types of materials, experimental systems and methods used in many studies. Here, indicate whether each material, system or method listed is relevant to your study. If you are not sure if a list item applies to your research, read the appropriate section before selecting a response.

Materials & experimental systems

n/a	Involved in the study
☐	☒ Antibodies
☐	☒ Eukaryotic cell lines
☒	☐ Palaeontology and archaeology
☐	☒ Animals and other organisms
☒	☐ Clinical data
☒	☐ Dual use research of concern

Methods

n/a	Involved in the study
☒	☐ ChIP-seq
☒	☐ Flow cytometry
☒	☐ MRI-based neuroimaging

Antibodies

Antibodies used	anti-dsRNA clone J2 (Sigma-Aldrich, MABE1134: Jena Bioscience RNT-SCI-10010200), anti-mouse IgG-HRP (Sigma-Aldrich, A9044)
Validation	Anti-dsRNA monoclonal antibody J2 recognises double-stranded RNA (dsRNA) provided that the length of the helix is greater than or equal to 40 bp. dsRNA-recognition is independent of the sequence and nucleotide composition of the antigen. All naturally occurring dsRNAs investigated up to now (40-50 species) as well as poly(I)-poly(C) and poly(A)-poly(U) have been recognised by Anti-dsRNA monoclonal antibody J2 although in some assays its affinity to poly(I)-poly(C) is about 10 times lower than that to other dsRNA antigens. Species Origin: Mouse. Heavy Chain Isotype: IgG2a. Light Chain Isotype: kappa. Quality control: Purity/Identity, Reducing and Non-reducing SDS-PAGE; Activity, AN-ELISA (relative activity compared to reference J2).

Eukaryotic cell lines

Policy information about cell lines and Sex and Gender in Research

Cell line source(s)	RIKEN Cell Bank (HeLa, HeLa S3), ATCC (JAWS II), BPS Bioscience (NF-κB reporter (Luc)-HEK293 cells)
Authentication	The above sources comprehensively performs authentication and quality-control tests on all distribution lots of cell lines using morphology monitoring, STR profiling, PCR assays with species-specific primers.
Mycoplasma contamination	All cell line tested negative.
Commonly misidentified lines (See ICLAC register)	No commonly misidentified cell lines were used in the study.

Animals and other research organisms

Policy information about studies involving animals; ARRIVE guidelines recommended for reporting animal research, and Sex and Gender in Research

Laboratory animals	Balb/c mouse (female, 7-week-old, Charles River Laboratories Japan, Inc., Kanagawa, Japan) Housing conditions were maintained as follows: 12-h/12-h dark/light cycle, 23+/- 2 °C temperature and 40 to 60% humidity.
Wild animals	No wild animals were used in the study.
Reporting on sex	No analysis based on sex or gender was performed as it is not relevant to the study.
Field-collected samples	No field collected samples were used in the study.
Ethics oversight	The animal experiments were conducted under the approval of the animal care and use committees at Kyoto Prefectural University of Medicine (Kyoto, Japan) and Innovation Center of NanoMedicine (iCONM, Kawasaki, Japan).

Note that full information on the approval of the study protocol must also be provided in the manuscript.
